# Broad antibiosis activity of *Bacillus velezensis* and *Bacillus subtilis* is accounted for by a conserved capacity for lipopeptide biosynthesis

**DOI:** 10.3389/fmicb.2025.1636481

**Published:** 2025-08-29

**Authors:** Jahangir Alam, Oluwakemisola E. Olofintila, Francesco S. Moen, Zachary A. Noel, Mark R. Liles, Douglas C. Goodwin

**Affiliations:** ^1^Department of Chemistry and Biochemistry, Auburn University, Auburn, AL, United States; ^2^Department of Entomology and Plant Pathology, Auburn University, Auburn, AL, United States; ^3^Department of Biological Sciences, Auburn University, Auburn, AL, United States

**Keywords:** *Bacillus*, biocontrol, antibiosis, lipopeptide, *Phytophthora*, biosynthetic gene clusters

## Abstract

We evaluated 284 strains from 15 species across five genera in the Phylum Bacillota (*Bacillus*, *Priestia*, *Cytobacillus*, *Neobacillus*, and *Gottfriedia*) for antibiosis activity against the pathogenic oomycete, *Phytophthora nicotianae*. Fifty-eight strains were strong inhibitors, while 41 and 185 were weak and noninhibitors, respectively. Only *Bacillus* strains were strong inhibitors, and inhibitory metabolites were most frequently (55 of 58 strains) expressed from five species (*B. pumilus*, *B. safensis, B. altitudinis, B. velezensis*, and *B. subtilis*). Strongly inhibitory strains from *B. velezensis* (all) and *B. subtilis* (all but one) were also strong inhibitors of the fungal pathogens *Fusarium oxysporum*, *Fusarium graminearum*, and *Rhizoctonia solani*; therefore, these *Bacillus* strains were designated as *generalists*. The strong *P. nicotianae* inhibitors from *B. pumilus*, *B. safensis*, and *B. altitudinis* strains only weakly inhibited *R. solani and* did not inhibit *F. oxysporum* or *F. graminearum*; therefore, these strains were designated as *Oomycete specialists*. Lipopeptide-encoding biosynthetic gene clusters (BGCs) were prominently represented within the five bioactive generalist species and virtually absent from the 10 non-inhibitory species. Surfactin-encoding BGCs were observed across all specialists and generalists. *B. subtilis* strains also carried a fengycin BGC, and some *B. velezensis* strains were found to encode a novel iturin and fengycin BGC. Iturin (including bacillomycin L), fengycin, and surfactin were the most commonly observed lipopeptide BGCs among the most bioactive species, and many strains contained all three. Lipopeptides from strongly inhibitory *B. velezensis* JJ334 were isolated, identified, and characterized by LC-MS. Fengycin and bacillomycin L produced strong inhibition of oomycetes and fungi as compared to surfactin. Fengycin was the strongest inhibitor among lipopeptides evaluated. Six to thirteen derivatives of each lipopeptide were observed, varying primarily in fatty acid chain length.

## Introduction

Plant-pathogenic fungi and oomycetes are the cause of serious and intractable diseases that result in multiple billions of dollars in annual crop losses (Bebber and Gurr, 2015). Crop-destroying representative species from the oomycete *Phytophthora* are *P. infestans*, *P. sojae*, *P. capsica*, and *P. nicotianae*. Among these, *P. nicotianae* is the causative agent of blank shank of tobacco and has a broad host range, including citrus, cotton, apple, cashew, pistachio, tobacco, and tomato (Lamour, 2013). Economically important plant-pathogenic fungi responsible for severe diseases against a wide range of plants are *Magnaporthe oryzae*, *Botrytis cinerea*, *Fusarium graminearum*, *F. oxysporum*, and *Rhizoctonia solani* (Dean et al., 2012; Casadevall, 2018). The agricultural impacts of climate change combined with the overuse of synthetic fungicides have simultaneously produced an increase in fungal diseases and fungicide resistance (Hahn, 2014; Casadevall, 2018; Almeida et al., 2019). Due to these concerns, biological agents have been considered as an alternative and promising strategy for disease control and crop management (Gerbore et al., 2014). Studies have shown that bacteria from multiple Bacillaceae genera (e.g., *Bacillus*), as well as *Streptomyces* and *Pseudomonas* can be effective biocontrol agents against various plant pathogens (Syed et al., 2018; Köhl et al., 2019). In particular, *Bacillus* species have shown strong bioactivity against plant pathogens (Fira et al., 2018; Jiang et al., 2018; Saleh et al., 2021).

Species from multiple Bacillaceae genera inhabiting the soil and plant rhizosphere are particularly well-suited as biological agents due to their adaptations to terrestrial environments and their evolution as plant-associated rhizobacteria (Fira et al., 2018). Accordingly, many strains have been described as plant growth-promoting rhizobacteria (PGPR) for their contributions to plant growth and disease biocontrol (Kloepper, 1978). Factors contributing to these aspects include abilities in plant-root colonization and production of allelochemicals such as siderophores, antimicrobials, biocidal volatiles, lytic, and detoxification enzymes (Syed et al., 2018). The most common antimicrobials produced by species from Bacillaceae genera (especially *Bacillus* strains) are peptides, polyketides, betalactones, fatty-acid derivatives, and lytic enzymes (Kaspar et al., 2019). These compounds are known as secondary (or specialized) metabolites, often encoded by biosynthetic gene clusters (BGCs).

The most common bioactive secondary metabolites produced these organisms are non-ribosomal and ribosomal-peptides and polyketide-derived macrolides (Kaspar et al., 2019). The non-ribosomal peptides (NRP) are produced by multimodular BGCs called non-ribosomal peptide synthetases (NRPS) that accept proteinogenic or modified amino acids as substrates (Marahiel and Essen, 2009). The most extensively studied *Bacillus* NRPs are cyclic lipopeptides (e.g., surfactin, fengycin, iturin, etc.,), and siderophores (e.g., bacillibactin) (Kaspar et al., 2019). In particular, *Bacillus* lipopeptides are known to be strongly antagonistic against plant pathogenic fungi and oomycetes (Ongena and Jacques, 2008). Structurally, these lipopeptides are small peptides (5–12 amino acids) consisting of a cyclic lactone ring with a linked β-amino or β-hydroxy fatty acid of variable carbon chain length. The bioactivity of each lipopeptide varies significantly, ranging from broad to narrow-spectrum antifungal or antibacterial activity, which may depend on the chemical properties of constituent amino acids as well as fatty acid chain length and branching (Ongena and Jacques, 2008). The common modes of action of these lipopeptides are cell lysis, cell membrane leakage, inhibition of enzymes, and inhibition of protein synthesis of target pathogens (Kiesewalter et al., 2021).

Past studies have demonstrated excellent anti-infective activities of several *Bacillus* species against plant pathogens in correlation with their abilities to produce single or multiple secondary metabolites (Wang et al., 2020; Moreno-Velandia et al., 2021; Xie et al., 2021; Gu et al., 2017; Zhang and Sun, 2018). Strains from *B. velezensis* and *B. subtilis* are the two most extensively studied *Bacillus* species with activity against plant pathogens; for example, several studies demonstrated that *B. velezensis* FZB42 and SQR9 produce multiple antimicrobial secondary metabolites that collectively exert strong antibiosis activity against fungi and oomycetes (Li et al., 2014; Wu et al., 2014; Fan et al., 2018). However, the bioactivity of the *Bacillus* strain and derived natural products are mostly studied either in a single strain or a few strains within the same species. Consequently, a comparative overview of the conserved roles of such natural products in correlation with their diversity and extent of expression in diverse *Bacillus* species is missing from the literature. In particular, the breadth of *Bacillus* antibiosis activity across various plant pathogens such as oomycetes and fungi in connection with the ability for secondary metabolite production is poorly understood.

To address this gap, we carried out a comparative evaluation of the antibiosis activity of 284 strains of species from a diverse set of Bacillaceae genera in connection with their ability to produce antimicrobial secondary metabolites. Antibiosis screening of these organisms against a plant-pathogenic oomycete, *P. nicotianae*, identified 58 (20%) strongly inhibitory strains. These were further evaluated for broad-spectrum inhibitory properties against three plant pathogenic fungi *F. oxysporum*, *F. graminearum*, and *R. solani*. Genome analyses of these strains showed a strikingly strong conservation of three lipopeptide BGCs (iturin/bacillomycin L, fengycin, and surfactin) among a subset of strongly inhibitory *Bacillus* species. Bacillomycin L, fengycin, and surfactin were produced, extracted, and isolated from a representative strongly inhibitory strain, *B. velezensis* JJ334. Characteristic chemical properties and antibiosis activity of each purified lipopeptide were further evaluated using UV-vis absorption spectroscopy, liquid chromatography, high-resolution mass spectrometry, and plate-based antibiosis assays.

## Materials and methods

### Strains, chemicals, and culture conditions

In the present study, 284 PGPR strains from five Bacillaceae genera (*Bacillus*, *Priestia*, *Cytobacillus*, *Neobacillus*, and *Gottfriedia*) were obtained from the Plant-Associated Microbial collection that was assembled by Prof. Kloepper (1978) at Auburn University from various plant rhizospheres. Each of these PGPR strains were cryopreserved at −80 °C and were grown on Tryptic Soy Agar (TSA) for isolated colonies prior to evaluation as potential biological control agents. For genomic analysis, Illumina-generated draft genome sequences were analyzed, trimmed, and assembled using CLC Genomic Workbench^1^. Genome quality was further evaluated by CheckM v1.1.3. (Parks et al., 2015), and 284 genome sequences with returned genome completeness of greater than 70% were included in this study. A total of 29 (out of 284) *Bacillus* strains with the greatest biocontrol potential were further sequenced at Nanopore for single-contig complete genome sequence. As originally collected from 1989 through 2014, all of these PGPR strains were classified as belonging to the *Bacillus* genus. Accordingly, the taxonomy of all 284 strains was initially evaluated by the top hit of average nucleotide identity (ANI) of whole-genome sequence at Microbial Genomes Atlas (MiGA) webserver (Rodriguez-R et al., 2018). Upon NCBI database deposition, strain identities were confirmed and annotated by the NCBI RefSeq database (Goldfarb et al., 2024), and all strain information was made publicly available at BioProject Accession: PRJNA1078443 and ID: 1078443. A spreadsheet summarizing the information for each strain evaluated in this study (including each NCBI BioProject Strain Accession Number) can be found in Supplementary Table 1. As cited in Supplementary Table 1, 27 of the strains have been mentioned or investigated in previous publications. For convenience, those references are listed here as well (Ran et al., 2012; Rao et al., 2014; Hossain et al., 2015, 2019; Nasrin et al., 2015; Ravu et al., 2015; Liu et al., 2016, 2017; Thurlow et al., 2019; Wang et al., 2019; Devkota et al., 2020; Afroj et al., 2021; Kumar et al., 2011; Olajide et al., 2022; da Silva et al., 2023; Yustiati et al., 2023; Romanenko et al., 2024; Sun and Shahrajabian, 2025). Notably, reclassification as proposed by Gupta et al. (2020), Patel and Gupta (2020) assigned clades within the *Bacillus* genus into multiple Bacillaceae genera. Accordingly, all 284 strains were taxonomically distributed into 15 species. Six species fell within non-*Bacillus* Bacillaceae including *Priestia megaterium* (65 strains), *Cytobacillus firmus* (5 strains), *Neobacillus dretensis* (17 strains), *N. vireti* (5 strains), *N. niacini* (5 strains), and *Gottfriedia acidiceleris* (6 strains). The remaining nine species were retained within the *Bacillus* genus: *B. pumilus* (17 strains), *B. safensis* (24 strains), *B. altitudinis* (14 strains), *B. velezensis* (50 strains), *B. subtilis* (6 strains), *B. toyonensis* (15 strains), *B. thuringiensis* (13 strains), and *B. pseudomycoides* (5 strains). Finally, “Bacillaceae (other)” representing a collection of 37 strains from less commonly observed species contributing < 5 strains each to the pool were evaluated. A phylogenetic tree was constructed using the 16S rRNA sequence of each type strain matching all 15 species obtained from EZbioCloud database (Yoon et al., 2017). For routine bacterial growth, the bacteria were cultured at 30 °C in TSA or tryptic soy broth (TSB) medium with shaking at 200 rpm.

### Antibiosis assay of strains against *P. nicotianae* and fungal pathogens

All strains were screened for their abilities to inhibit the growth of the root-associated plant-pathogenic oomycete, *P. nicotianae* in a plate-based assay. *P. nicotianae* was grown in a V8 agar medium (180 mL/L V8 juice, 2 g/L CaCO_3_, and 15 g/L Bacto agar) while the Bacillaceae strains were grown on TSA. Assay plates were prepared using the V8 agar medium in which bacterial colonies were transferred into a well (diameter = 10 mm) containing TSA at the edge of the plate and the *P. nicotianae* was transferred as a plug to the center of the plate. The growth inhibition of *P. nicotianae*’s hyphae due to the presence of Bacillaceae colonies was recorded after a 7–10 days incubation. The inhibitory responses of Bacillaceae strains were classified as strong, weak, and no inhibition based on the measurement of the zone of inhibition (ZOI) and morphological changes of the Bacillaceae strain and *P. nicotianae* being evaluated. A strong inhibition was assigned for a clear zone of inhibition (ZOI) of 5–15 mm with complete elimination of *P. nicotianae*’s hyphae in the interface of *Bacillus* colonies and *P. nicotianae*, while no inhibition was assigned when the *P. nicotianae*’s hyphae spread over the *Bacillus* colonies with no observable ZOI. Exhibition of a less clear ZOI of 2–7 mm with substantial reduction of *P. nicotianae*’s hyphae was assigned as weak inhibition. Fifty-eight *P. nicotianae*-inhibitory Bacillaceae strains (all from *Bacillus* species) were further evaluated for antibiosis against *F. oxysporum*, *F. graminearum*, and *R. solani*. Each organism was assayed and evaluated in the same condition as described above for *P. nicotianae*, and the resulting antibiosis response was similarly classified as strong, weak, and no inhibition based on ZOI and morphological changes of the organisms evaluated.

### Calculation of bioactivity index

Conservation of antibiosis activity expressed by various Bacillaceae species was calculated on the basis of the distribution of strong, weak, and no inhibition among strains within each species. A term “bioactivity index” accounting for such conservation of antibiosis activity was calculated using a weighted-average score of 1 for strong inhibition, 0.5 for weak inhibition, and 0 for no inhibition using the following equation:


B⁢i⁢o⁢a⁢c⁢t⁢i⁢v⁢i⁢t⁢y⁢I⁢n⁢d⁢e⁢x=

$$\frac{\begin{array}{c} \left(\#\mathrm{strong}\mathrm{inhibitor}\mathrm{strains}\times 1\right)+(\#\mathrm{weak}\mathrm{inhibitor}\mathrm{strains}\\ \times 0.5)+(\#\mathrm{noninhibitory}\mathrm{strains}\times 0) \end{array}}{\#\,\mathrm{of}\,\mathrm{total}\,\mathrm{strains}\,\mathrm{within}\,a\,\mathrm{species}}$$

As defined, bioactivity index of 1 indicates the highest expression of antibiosis activity, where all tested strains of a given species show strong antibiosis activity. Conversely, a 0 indicates no expression of antibiosis activity (i.e., no tested strains of a given species showed antibiosis activity).

### Genome mining and bioinformatics analyses of Bacillaceae strains

Genome sequences of all 284 Bacillaceae strains were analyzed by antiSMASH v.5 (antibiotics and secondary metabolite analysis shell) to predict biosynthetic gene clusters (BGCs) and secondary metabolites. Predicted BGCs were further dereplicated based on respective BGCs from single contig complete genome sequences to eliminate duplicated and/or fragmented BGCs. To infer conservation in sequence and putative function, predicted BGCs were grouped into networks of clusters based on sequence similarities using BiG-SCAPE v.0.0.0r (Biosynthetic Gene Similarity Clustering and Prospecting Engine) (Navarro-Muñoz et al., 2020). Finally, network distances generated by BiG-SCAPE analysis were visualized and annotated using Cytoscape 2.8 (Smoot et al., 2011).

### Extraction of secondary metabolites from bioactive *Bacillus* strains

Representative strains from the five bioactive *Bacillus* species were selected for producing secondary metabolites that may be responsible for antibiosis activity. In order to produce secondary metabolites, the *Bacillus* strains were grown in Landy medium (glucose, 20 g/L, yeast 1 g/L, L-glutamic acid 5 g/L, KCl 0.5 g/L, MgSO_4_ 0.5 g/L, KH_2_PO_4_ 1 g/L, L-phenylalanine 3 mg/L, MnSO_4_ 5 mg/L, FeSO_4_ 0.15 mg/L, CuSO_4_ 0.16 mg/L, pH 7.0) for 72 h at 30 °C with constant agitation (175 rpm). To pellet cells, liquid cultures were subjected to centrifugation at 6,000 × *g* for 40 min. The pH of harvested cell-free supernatant was adjusted to 2.0 by dropwise addition of concentrated HCl with constant stirring. Following overnight incubation at 4 °C, the precipitate was collected by centrifugation at 6,000 × *g* for 50 min. The precipitate was extracted twice using 100% MeOH. The pooled MeOH extract was dried under a constant flow of N_2_ (*g*), and the dried residue was redissolved in MeOH, filtered with a 0.2 μm Acrodisc syringe filter (Pall Corporation, Ann Arbor, MI), and stored at −20 °C until evaluated.

### Evaluation of antibiosis of total extracts against *P. nicotianae*

The antibiosis assay of the total extract of a representative strain from each of the five bioactive *Bacillus* species (i.e., *B. velezensis* JJ334, *B. subtilis* JM553, *B. pumilus*, *B. altitudinis*, and *B. safensis*) against *P. nicotianae* was carried out using a disk diffusion method (Wang et al., 2020). The assay was conducted using a V8 agar plate. Freshly grown *P. nicotianae* was transferred as a plug (diameter = 5 mm) to the center of the assay plate and allowed to grow for 72 h. A total of 10 μL of the total extract was added onto a sterilized filter disk (diameter = 6 mm) and then placed at the edge of the assay plate. The growth inhibition of *P. nicotianae* hyphae surrounding the filter disk was measured and recorded after 5–7 days of incubation at 25 °C. An example of antibiosis activity of total extracts of *B. velezensis* JJ334 against *P. nicotianae*, *F. oxysporum*, *F. graminearum*, and *R. solani* is shown in Supplementary Table 4.

### Evaluation of chemical properties of total extract by UV-vis absorption and LC-MS

UV-vis absorption spectra of *Bacillus* strain total extracts were evaluated for characteristic absorption features. To detect compounds at 220 nm, 20 μL of total extract were separated through a ZORBAX SB-C18 column (4.6 × 150 mm, 5 μm) for 60 min at a flow rate of 0.20 ml/min using an Agilent Infinity 1100 HPLC system (Santa Clara, CA). Solvent A (100% water) and B (100% acetonitrile), each containing 0.1% trifluoroacetate (v/v) were used with the following elution gradient for solvent B: 40% at 0 min, 55% at 15 min, 75% at 40 min, 100% at 60 min, and 75% at 77 min. To identify compounds in the total extract, 0.2 μL of the extract was separated by LC through an Acquity UPLC BEH C18 (2.1 × 50 mm, 1.7 μm) column and eluted onto a Thermo Fisher Exploris 120 orbitrap LC-MS (Milford, MA). The LC separation was run for 20 min with a column temperature of 40 °C and a flow rate of 0.20 mL/min. The mobile phase gradient was created using water (A) and acetonitrile (B), each containing 0.1% formic acid (v/v) such that solvent B was at 40% at 0 min and ramped to 100% at 14 min, and then back to 40% B at 16 min.

Ions were generated using both positive and negative ionization modes employing an electrospray ionization (ESI) source. In addition, fragment ions from the precursor ions were simultaneously produced in a high-stage MS*^n^* analyzer. The identity of compounds from the total extract were initially confirmed by the parent ions generated by both positive and negative ionization modes. The mass spectra of each fragmented ion generated by the MS*^n^* analyzer from the corresponding precursor ion produced in the positive ionization mode were used for the unambiguous identification of each compound. Further, characteristic fragment ions generated from lipopeptide core peptides were used as diagnostic ions for the identification of lipopeptide derivatives. For fengycin derivatives, two reporter fragment ions (A and B), generated by the cleavage of Orn2-Tyr3 (A) and Glu1-Orn2 (B) bonds from fengycin core peptide (Glu1-Orn2-Tyr3-Thr4-Glu5-Ala/Val6-Pro7-Gln8-Tyr9-Ile/Val10) were used as diagnostic ions for unambiguous identification (de Faria et al., 2011; Pathak et al., 2012; Volpon et al., 1999; Ait Kaki et al., 2020). For surfactin, the fragment ions generated by the cleavage of Glu1-Leu/Ile2 bond from the core peptide (Glu1-Leu/Ile2-Leu3-Val4-Asp5-Leu6-Leu/Ile7) and the remaining Glu1-fatty acid tail were used to determine the derivatives and length of fatty acid tail (Savadogo et al., 2011; Ma et al., 2016; Chen et al., 2017). Similarly, the fragment diagnostic ions generated by the cleavage of Asn-Tyr (278.11) and Asn-Tyr-Asn (392.15) fragments from the core peptide and fragmented fatty-acid tail were used for unequivocal identification of bacillomycin L derivatives (Dunlap et al., 2019).

### Isolation of bioactive secondary metabolites using HPLC

Agilent Infinity 1,100 LC system was used for isolating bioactive compounds from the total extract of *Bacillus* strains. A total of 100 μL of the total extract was injected and eluted through Zorbax (Santa Clara, CA) SB-C18 column (4.6 × 150 mm, 5 mm) for 60 min, at a flow rate of 0.20 mL/min. The mobile phases were water (A) and acetonitrile (B), each containing 0.1% trichloroacetic acid (v/v) with the following gradient for solvent B: 40% at 0 min, 55% at 15 min, 75% at 40 min, 100% at 60 min, and 75% at 77 min. The compounds were detected at 220, 275, 375, and 450 nm by a diode array detector coupled with a full-spectrum (220–500 nm) analysis. Fifteen to 20 fractions were collected, pooled and concentrated from five consecutive runs. The purity of compounds in each fraction was evaluated by UV-vis absorption as well as by MS analyses.

### Antibiosis evaluation of isolated lipopeptides and target pathogens

Purified bacillomycin L, fengycin, and surfactin were evaluated for bioactivity against *P. nicotianae*, and a representative fungus, *F. oxysporum* using a disk diffusion assay (Wang et al., 2020). The assay was conducted on a V8 agar plate wherein a freshly grown target pathogen was transferred as a plug (d = 5 mm) to the center of the assay plate and allowed to grow for 72 h. A total of 10 μL of the purified lipopeptide was added into a sterilized filter disk (d = 6 mm) and then transferred onto the edge of the assay plate. The growth inhibition of *P. nicotianae*’s hyphae surrounding the disk was measured and recorded after 5–7 days. In order to determine the inhibitory strength of purified bacillomycin L, fengycin, and surfactin against *P. nicotianae* and *F. oxysporum*, a quantitative bioassay was carried out using a 96-well microtiter-based plate assay as described by Noel et al. (2019) with minor modifications. Both *P. nicotianae* and *F. oxysporum* were grown in a diluted V8 medium (80 mL/L of V8 juice, 1 g/L of CaCO_3_, and 6 g /L Bacto agar). Freshly grown plugs of target pathogens were macerated by passing through a 22-gauge needle attached to a 10 mL syringe and further homogenized by vortexing for 2 min. A total of 20 μL of homogenized culture macerate, 160 μL of diluted V8 broth (80 mL/L of V8 juice and 0.5 g/L CaCO_3_), and 20 μL of lipopeptide with desired concentration were then loaded into a 96-well microtiter plate using wide-orifice tips. Plates were incubated for 2 days at 25 °C and the growth of the target organism was determined spectrophotometrically at 600 nm by a microtiter plate reader (Biotek Instruments, Highland Park, VT).

## Results

### Concentration of *P. nicotianae* antibiosis activity among five *Bacillus* species

**FIGURE 1:**
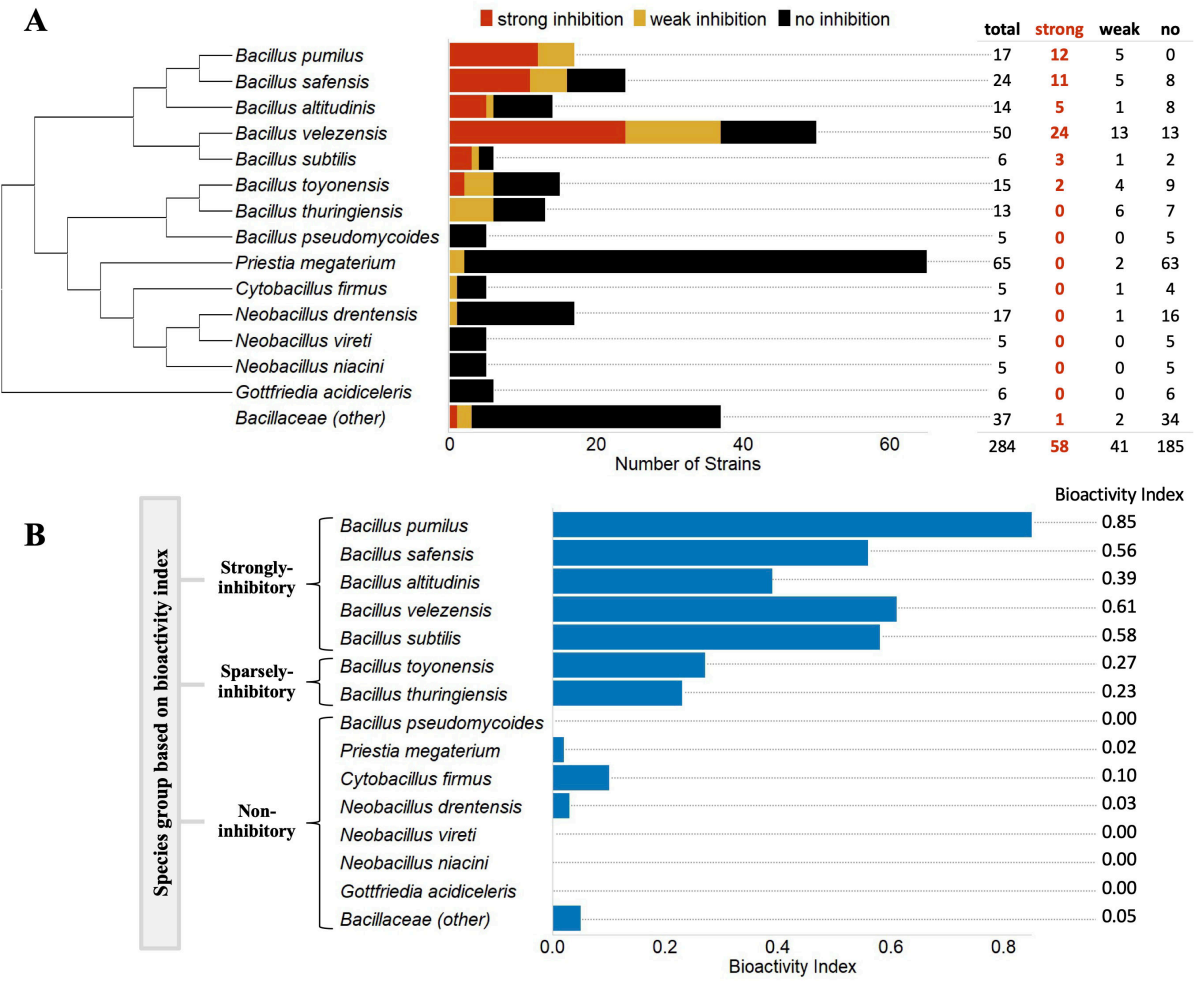
Antibiosis activity Bacillaceae species against *P. nicotianae*. The antibiosis activity of 284 strains across 14 species (eight *Bacillus* and six non-*Bacillus* Bacillaceae) against the plant-pathogenic oomycete, *P. nicotianae* is summarized **(A)**. Antagonism was classified as strong, weak, or no inhibition. Species denoted as “Bacillaceae (other)” contained strains from less-commonly observed species, each contributing fewer than five strains. A description of all strains evaluated is available in Supplementary Table 1. The degree to which antibiosis activity against *P. nicotianae* was expressed across strains from a given species was expressed as a bioactivity index (BI) **(B)**. Scores for BI range from 0 to 1, where 0 would indicate that no strains within a species demonstrated any antibiosis activity, and 1 would indicate that all strains within a species demonstrated strong antibiosis activity against *P. nicotianae* (see section “Materials and methods”). Five species were classified as strongly inhibitory based on BI values from 0.39 to 0.85, two were classed as sparsely inhibitory with values from 0.23 to 0.27, and the rest were regarded as non-inhibitory with BI values less than or equal to 0.10.

A library of 284 PGPR strains representing 15 species across five Bacillaceae genera was evaluated for antibiosis activity against a root-associated plant-pathogenic oomycete, *P. nicotianae*. Antagonism against *P. nicotianae* was classified as strong, weak, or non-inhibitory based on measurements of zones of inhibition (ZOI) as well as evaluation of morphological changes to *P. nicotianae* and the respective Bacillaceae strain. Defining characteristics of these levels of inhibition are given in section “Materials and methods,” and a representative antibiosis assay plate illustrating all three levels of inhibition was also documented (Supplementary Figure 1). Fifty-eight (20%) of the 284 strains exhibited strong inhibition, while 41 strains were weak inhibitors, and 185 showed no pathogen inhibition. All 58 of the strongly inhibitory strains belonged to *Bacillus* species. Across the 103 strains from the other four Bacillaceae genera (*Priestia*, *Cytobacillus*, *Neobacillus*, and *Gottfriedia*) none showed strong inhibition, and only four showed weak inhibition of *P. nicotianae*. Further, 55 out of the 58 strongly inhibitory strains belonged to five *Bacillus* species: *B. pumilus*, *B. safensis, B. altitudinis, B. velezensis*, and *B. subtilis* (Figure 1A). Interestingly, these five species are more closely phylogenetically related to one another as compared to the other species (Figure 1A), suggesting that the common factors contributing to antibiosis activity may be phylogenetically conserved. For each of these species, at least 40% of strains tested exhibited some level of inhibition (strong or weak): *B. velezensis* (74%), *B. pumilus* (100%), *B. safensis* (67%), *B. subtilis* (67%), and *B. altitudinis* (43%). Accounting for the overall percentage of inhibitory strains as well as the relative contribution of strong vs. weak vs. non-inhibitory strains, a bioactivity index (ranging from 0 to 1) was calculated (see section “Materials and methods”); these five species returned values of 0.39 (*B. altitudinis*), 0.58 (*B. subtilis*), 0.56 (*B. safensis*), 0.61 (*B. velezensis*), and 0.85 (*B. pumilus*) (Figure 1B). Inhibition was sparsely distributed among *B. toyonensis*, and *B. thuringiensis* strains, generating bioactivity indices of 0.27, and 0.23, respectively. Finally, all other species tested showed bioactivity indices ≤0.10: *B. pseudomycoides* (0)*, P. megaterium* (0.02)*, C. firmus* (0.1), *N. drentensis* (0.03)*, N. vireti* (0)*, N. niacin*i (0), and *G. acidiceler* (0), and *Bacillus* (other) (0.05) (Figure 1B).

### *P. nicotianae*-inhibitory *Bacillus* species divided into specialists and generalists

**FIGURE 2:**
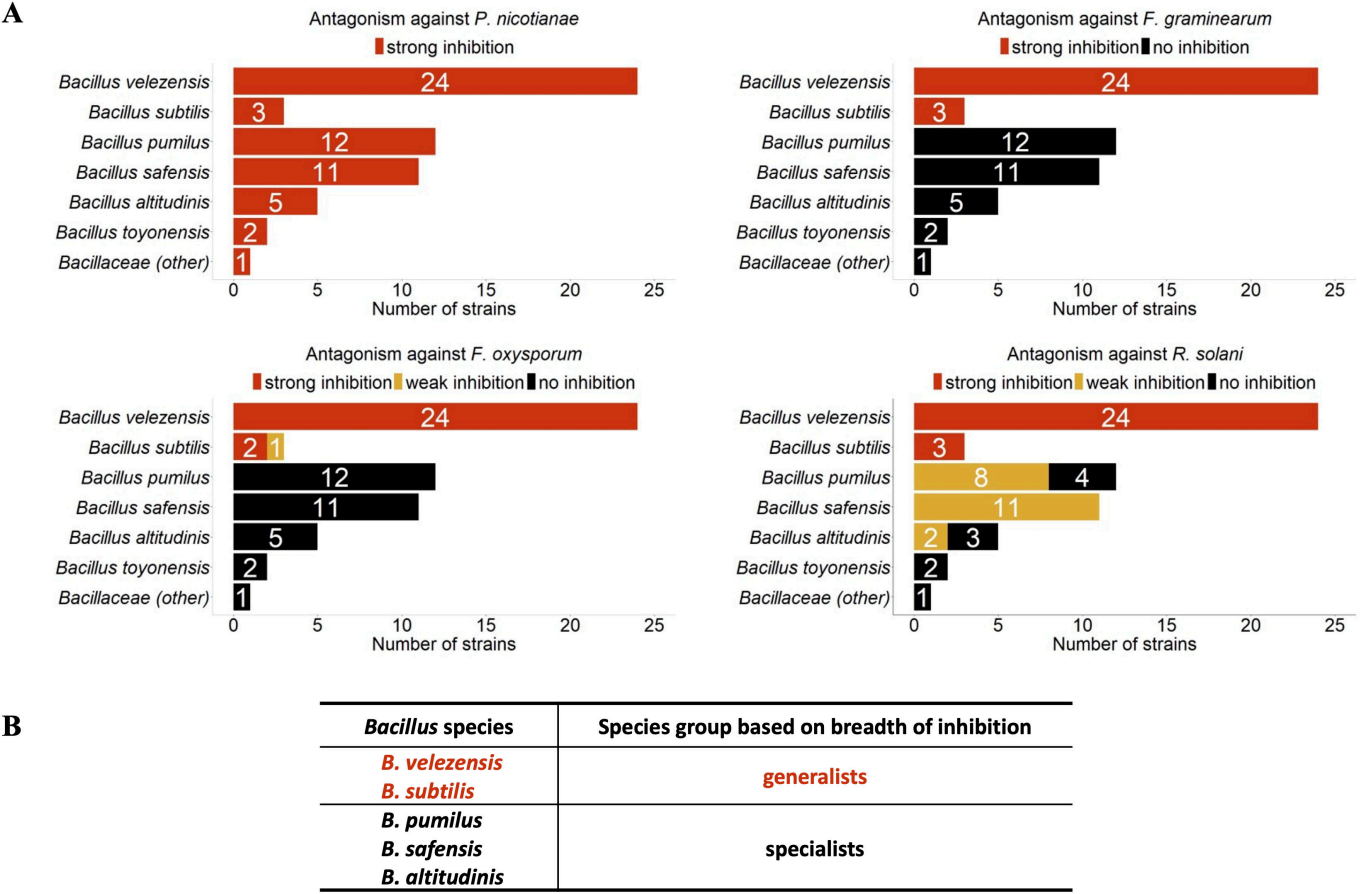
Antibiosis activity of 58 *P. nicotianae*-active Bacillus species. Antibiosis response of *P. nicotianae*-inhibitory *Bacillus* strains are shown against three root-associated plant-pathogenic fungi, *F. graminearum, F. oxysporum*, and *R. solani* **(A)**. Based on the breadth of their inhibition against plant pathogens, the five strongly *P. nicotianae*-inhibitory *Bacillus* species were further subdivided into *generalists* and *specialists* **(B)**. The single Bacillaceae (other) strain reported is *Bacillus sp. JJ1609*.

The 58 strong inhibitors of *P. nicotianae* were further evaluated for antibiosis activity against three fungal pathogens: *F. graminearum, F. oxysporum*, and *R. solani.* Interestingly, strains from *B. velezensis* and *B. subtilis* exhibited strong antibiosis activity against all three fungal pathogens whereas strains from *B. pumilus*, *B. safensis*, *B. altitudinis*, *B. toyonensis*, and *Bacillus (other)* exhibited either weak inhibition or were non-inhibitory (Figure 2). Specifically, all strains from *B. pumilus*, *B. safensis*, and *B. altitudinis* exhibited no inhibition against both *F. oxysporum* and *F. graminearum*. Further, only weak inhibition was observed against *R. solani*. As shown in Figure 1A, *B. velezensis* and *B. subtilis* are phylogenetically closely related; similarly, *B. pumilus*, *B. safensis*, and *B. altitudinis* are closely related to one another. Due to their ability to exert strong antagonism against *P. nicotianae* and all three pathogenic fungi, we classified *B. velezensis* and *B. subtilis* as generalists for their broad-spectrum bioactivity against fungal and oomycete pathogens; conversely, we classified *B. pumilus*, *B. safensis*, and *B. altitudinis* as oomycete-inhibiting specialists.

### Number, type, and distribution of BGCs are distinct between strongly inhibitory and non-inhibitory *Bacillus* species

We surmised that strains strongly inhibitory against *P. nicotianae* were likely to possess a conserved capacity for biosynthesis of secondary metabolites which would account for their antibiosis activity, and these BGCs would be absent from the non-inhibitory species. In order to evaluate this hypothesis, draft genome sequences of 284 *Bacillus* strains were analyzed for the presence of predicted BGCs using antiSMASH (v.5) (Blin et al., 2019). A total of 2,446 BGCs (average eight per strain) were predicted across the 284 genomes, including 1,279 known and 1,167 BGCs that do not have a known natural product. Based on the structural and chemical properties of predicted secondary metabolites, BGCs were grouped into six functional classes: NRPS, PKS, a hybrid of PKS and NRPS (PK-NRPS), ribosomally-synthesized and post-translationally modified peptides (RiPP), terpenes, and BGCs outside these five classes (other). Information regarding *Bacillus* species, strain IDs, anti-*P. nicotianae* bioactivity, and predicted BGCs (with corresponding secondary metabolites) are available in Supplementary Table 2.

*Bacillus* species affiliated with the strong inhibitory group generally tended to have a higher number of BGCs per strain (8.9–13.2) with *B. velezensis* carrying the largest number of BGCs per strain. By comparison, the sparsely inhibitory species group contained 9.0–10.1 BGCs per strain, and the non-inhibitory species group contained 4.2–8.4. Consistent with these observations, the correlation between the overall number of BGCs and bioactivity was strong (Pearson *r* = 0.80; *p* = 0.0001).

**FIGURE 3:**
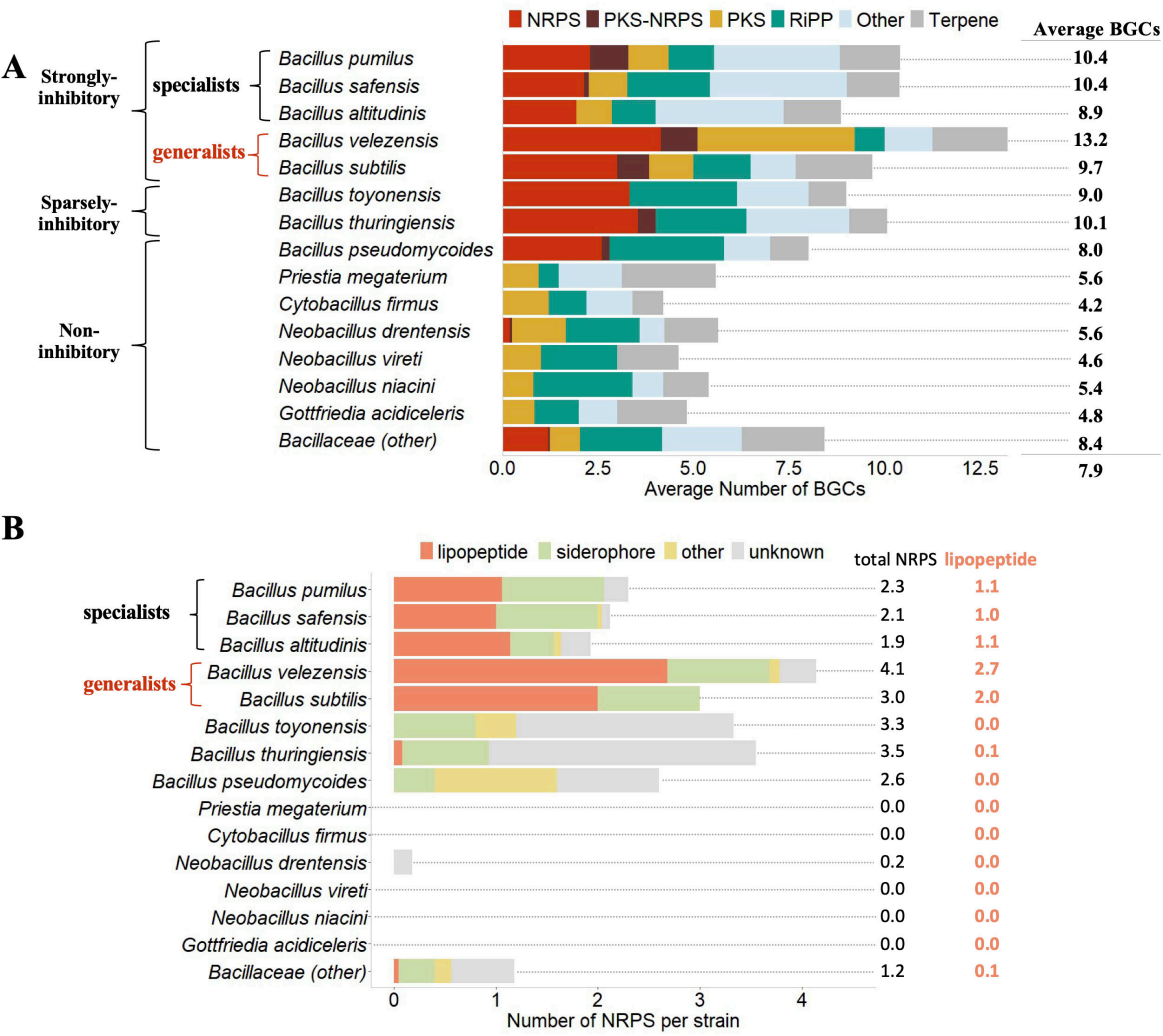
Bacillaceae biosynthetic gene cluster (BGC) diversity as related to strength and breadth of antibiosis activity. The average numbers of BGCs by type on a per strain basis among the species tested are shown **(A)**. All BGCs were classified into six functional groups based on the structural and chemical properties of the secondary metabolites predicted by antiSMASH. Species grouped into three classes based on the bioactivity index are designated next to the species, and species groupings into “generalist” vs. “specialist” based on the breadth of inhibition against various organisms also are indicated (see section “Materials and methods”). The distribution of specific types of non-ribosomal peptide synthetases (NRPS) BGCs across the Bacillaceae species tested are also shown **(B)**. The NRPS BGCs were subdivided based on structural and chemical properties of the predicted secondary metabolite: lipopeptide, siderophore, other, and unknown.

There was a striking distinction in the representation of NRPS clusters between strains from *Bacillus* species which averaged 2.67 clusters per strain versus species of the other Bacillaceae which were essentially devoid of them (average = 0.03 NRPS/strain) (Figure 3A). With respect to inhibition, a distinction was also observed between the strong-inhibition generalists (*B. velezensis* and *B. subtilis*) with an average 3.0–4.1 per strain, while the strong-inhibition specialists (*B. pumilus*, *B. safensis*, and *B. altitudinis*) only carried 1.9–2.3 NRPS per strain. As a contrast, PKS, RiPP, and terpene BGCs were evenly distributed across all of the Bacillaceae species evaluated, regardless of the level or breadth of inhibition exhibited. Accordingly, correlations between NRPS content and bioactivity were relatively strong (Pearson *r* = 0.63, *p* = 0.0069) while those between RiPPs and Terpenes were quite poor (Pearson *r* = −0.24, *p* = 0.3640; Pearson *r* = 0.01, *p* = 0.9620, respectively). The PKS and other BGCs occupied the middle, showing moderate correlations with bioactivity index (Pearson *r* = 0.42, *p* = 0.0953, and Pearson *r* = 0.58, *p* = 0.0153, respectively). Interestingly, the tightest correlation parameters between bioactivity index and BGC type were observed for the PKS-NRPS clusters (Pearson *r* = 0.79, *p* = 0.0002); however, one species from the strong inhibitory group (*B. altitudinis*) and one from the sparsely inhibitory group (*B. toyonensis*) carried no PKS-NRPS BGCs.

In order to compare their distribution among the bioactive *Bacillus* species based on their structural and chemical properties, NRPS clusters were further divided into four subtypes: lipopeptides, siderophores, other, and unknown. All five species belonging to the strongly inhibitory group had at least one and up to 2.7 lipopeptide BGCs per strain on average (Figure 3B). In contrast, lipopeptides were nearly completely absent from all other Bacillaceae species tested (i.e., the sparingly inhibitory and non-inhibitory species groups). Indeed, none were detected in any of the non-*Bacillus* Bacillaceae, *B. pseudomycoides*, or *B. toynensis* strains. Across the *B. thuringiensis* and *Bacillus* (other) strains tested the average number of lipopeptide clusters was 0.1 per strain. Siderophores were relatively common among species from strongly and sparsely inhibitory groups, while NRPS classified as “other” were almost completely absent from inhibitory species, and the distribution of unknown NRPS was sporadic. These data suggest that lipopeptides, almost exclusively produced by strains strongly antagonistic to *P. nicotianae*, may be substantial contributors to strong antibiosis activity. Interestingly, species from the generalist group contained two to three lipopeptides per strain while species from the specialist group contained only one lipopeptide per strain. This indicates that a diverse set of lipopeptides may produce a synergistic effect that contributes to the broad-spectrum antibiosis activity exhibited by generalists.

### Specific lipopeptide BGCs are highly conserved among generalists versus specialists

**FIGURE 4:**
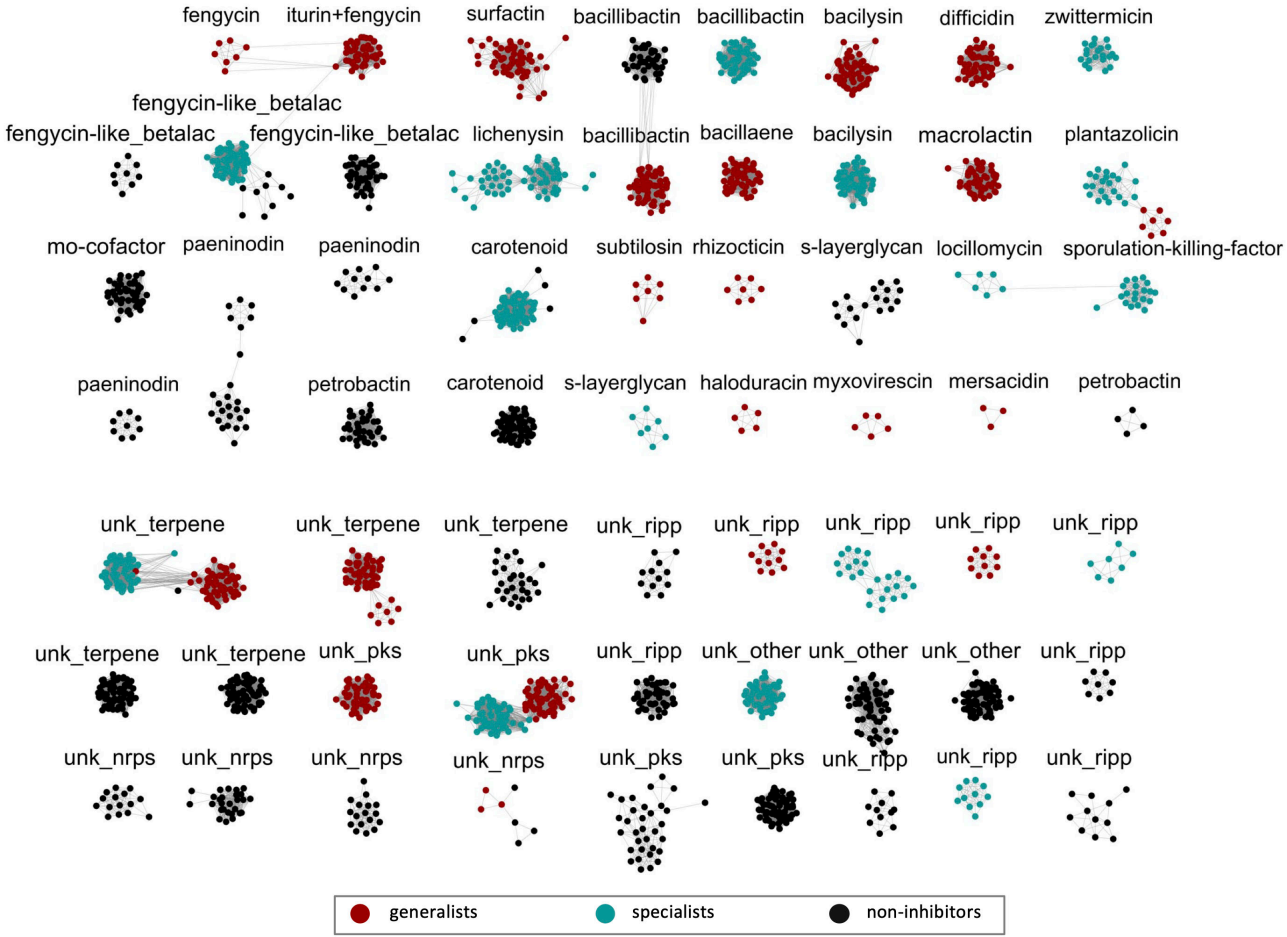
Similarity analysis of Bacillaceae biosynthetic gene clusters (BGCs). All 2,446 BGCs identified were grouped based on the distance matrices of gene cluster estimated by BiG-SCAPE (v.0.0.0r) (Navarro-Muñoz et al., 2020) analysis. Clusters containing three or more nodes (2,055 BGCs) are shown here and each cluster is labeled according to the secondary metabolite predicted by antiSMASH v.5 (Blin et al., 2019). An image including all 2,446 BGCs can be found in Supplementary Figure 3. The color of nodes is according to the breadth of observed antibiosis activity (generalist – red; specialist – teal; non-inhibitor – black) for the strain containing the BGC identified. The diversity of BGCs connected with fengycin and surfactin/lichenysin production are located in the top-left corner of the figure. Abbreviations for cluster labels are as follows: fengycin-like betalactone (fengycin-like_betalac), molybdenum cofactor (mo-cofactor), unknown RiPP (unk_ripp), unknown NRPS (unk_nrps), unknown PKS (unk_pks), and unknown Other BGC (unk_other).

**FIGURE 5:**
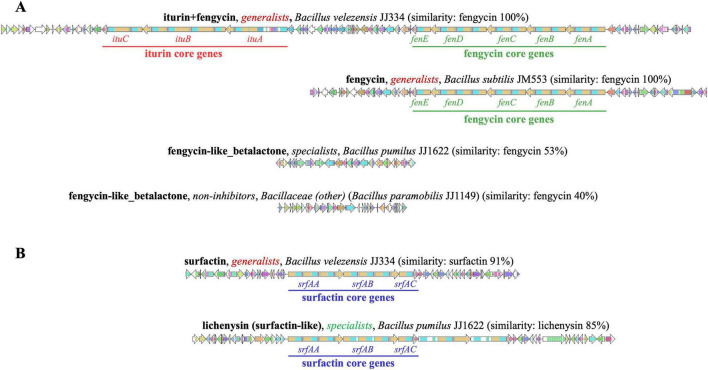
Comparison of lipopeptide biosynthetic gene cluster (BGC) gene organization. Representative BGCs from four distinct types of clusters identified as having fengycin similarity are shown **(A)**. These four include an iturin and fengycin tandem cluster identified exclusively in *B. velezensis* strains, a fengycin-only cluster identified exclusively in *B. subtilis* strains, and two fengycin-like betalactone BGCs, neither of which contain core genes for the production of a fengycin metabolite. One type is observed in specialists, and the other is observed in non-inhibitors. Representative BGCs from two distinct types of clusters identified as having surfactin (or lichenysin) similarity are shown **(B)**. Both types contain core genes for the production of surfactin or a surfactin-like lipopeptide. The BGC identified exclusively in generalists contains only the core-gene modules for the production of a surfactin-like heptapeptide. The BGC identified exclusively in specialists contains two additional core-gene modules.

Biosynthetic gene clusters from all 284 *Bacillus* strains were further analyzed (BiG-SCAPE v.0.0.0r) (Navarro-Muñoz et al., 2020) for gene cluster similarities to determine the extent to which secondary metabolite biosynthesis is conserved. Similarity analysis showed that BGCs identified as encoding a common putative metabolite across generalists, specialists, and non-inhibitory strains tended to segregate into separate clusters corresponding to these groups (Figure 4). This was particularly striking among lipopeptide BGCs where the differences in the structure of the gene clusters were highly distinct between antibiosis-based species groupings. Only generalists carried BGCs with modules for synthesis of the fengycin core decapeptide (*fenA* – *fenE*). Interestingly, the fengycin BGC from *B. velezensis* also contained *ituA*, *ituB*, and *ituC*, the modules necessary for the synthesis of an iturin core heptapeptide (Figure 5A). Although *Bacillus* strains from specialist and non-inhibitory species also carried a gene cluster identified as having similarity to a fengycin BGC, none of these contained modules for core peptide synthesis, but only genes supporting the synthesis of a putative betalactone. Interestingly, the specific structures of this BGC from specialists on one hand and non-inhibitory *Bacillus* species on the other were distinct (Figure 5A). Similarly, generalist and specialist *Bacillus* species groups all carried a BGC for production of a surfactin-like lipopeptide (Figure 4); no such BGC was identified in any strains from non-inhibitory species. Invariably, generalists (*B. velezensis* and *B. subtilis*) carried a BGC identified as surfactin (Figure 4) characterized by three core genes with modules for the synthesis of a heptapeptide (*srfAA* – *srfAC*) (Figure 5B). A separate bi-lobed cluster was observed for specialists with high similarity scores (85%) for lichenysin, a surfactin-like lipopeptide (Figure 4). The typical structure for this BGC contained two core genes in addition to the three required for heptapeptide synthesis (Figure 5B). These data suggest that the two to three highly conserved lipopeptides produced by generalists may contribute to a broad antifungal/anti-oomycete activity while the single surfactin or surfactin-like BGC carried by specialists may only enable strong anti-oomycete activity. We have observed that some strains belonging to generalist or specialist species exhibited no (or weak) inhibition (see Figure 1A) even though they appear to carry lipopeptide BGCs. Although the present study does not address this issue, it is possible that these strains may ultimately be unable to produce one or more of these lipopeptides due to missing core genes, nonsense mutations, frameshift mutations, and/or altered gene regulation. Such phenomena have previously been observed and reported for lipopeptide production by among *B. subtilis* strains, some even co-isolated from the same soil samples (Kiesewalter et al., 2021).

It should also be noted that other clusters of BGCs that putatively generate metabolites with antimicrobial properties segregate along the lines of generalists versus specialists versus non-inhibitors as well. Many of these BGCs belong to NRPS, PKS-NRPS, or PKS classes. For example, unique bacillibactin BGCs were identified for each of the three groups, and unique bacilysin clusters were each observed for generalists and specialists while non-inhibitors appeared to lack such a cluster altogether. In a similar manner, a bacillaene BGC is found only in generalists, and zwittermicin BGCs were only observed in specialists. Notably, the PKS BGCs for macrolactin and difficidin were only observed in *B. velezensis*.

### Antibiosis generalists produce at least two out of three lipopeptides: iturin, fengycin, and surfactin

**FIGURE 6:**
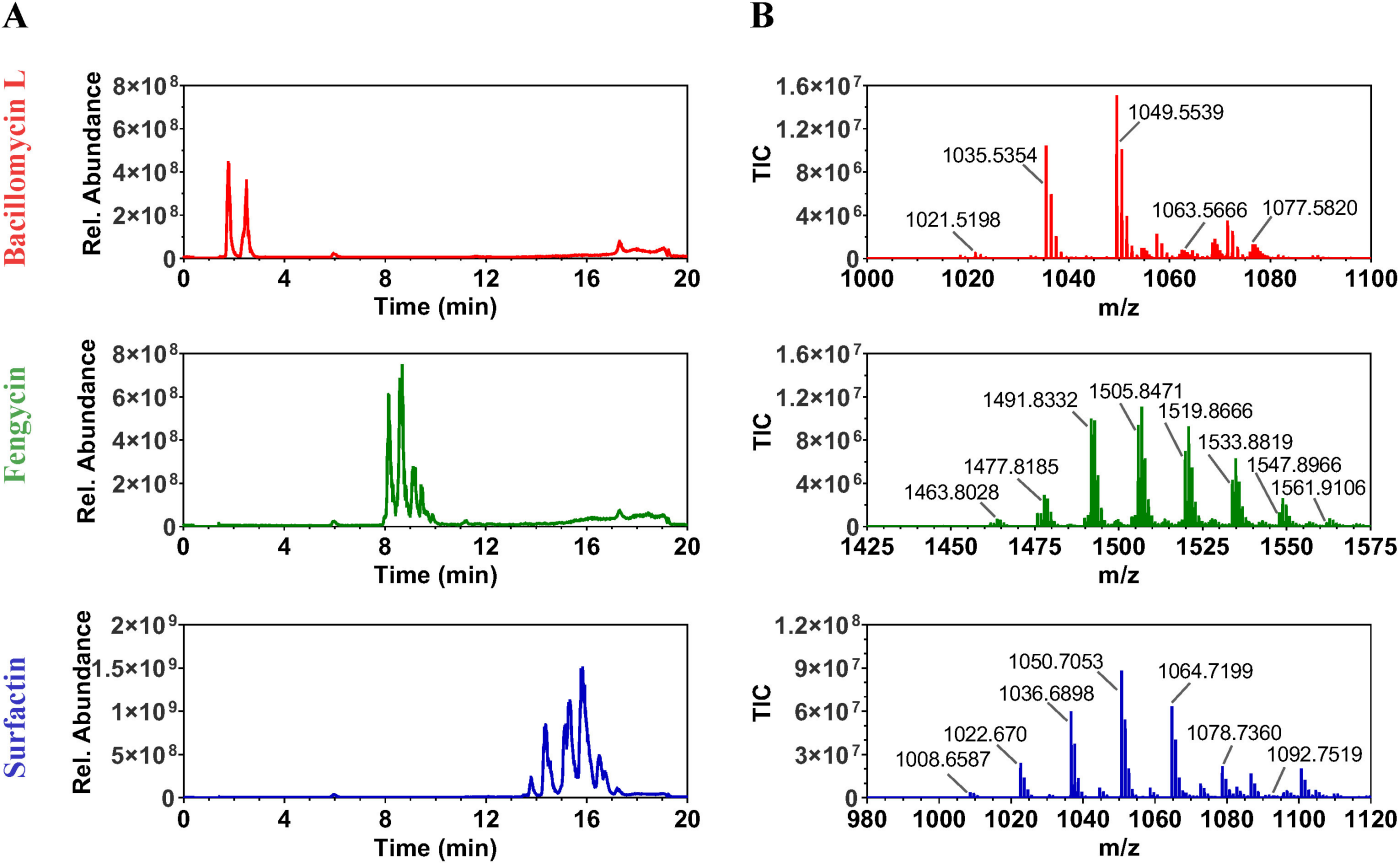
Chromatograms **(A)** and mass spectra **(B)** for bacillomycin L (an iturin), fengycin, and surfactin. Fractions containing each lipopeptide were obtained from acid-methanolic extracts of *B. velezensis* JJ334. Mass spectra were obtained in positive-ion mode, capturing [M+H]^+^ and/or [M+Na]^+^ ions of multiple derivatives of bacillomycin L, fengycin, and surfactin.

Lipopeptides were produced by and extracted from five strains, each representing a *Bacillus* species with strong *P. nicotianae* inhibitory activity, including, *B. velezensis* JJ334, *B. subtilis* JM553, *B. pumilus* JJ1622, *B. safensis* JJ1244, and *B. altitudinis* JJ1138. Each metabolite extract was evaluated by UV-vis and LC elution profile (see Supplementary Figure 4). Consistent with the structure and predicted products of its BGCs, mass spectrometric screening of *B. velezensis* strain JJ334 extracts showed the production of bacillomycin L (an iturin), fengycin, and surfactin. Fengycin and surfactin were identified in extracts of the *B. subtilis* strain, JM553. This also was consistent with the fengycin BGC observed across *B. subtilis* strains which contained core genes for only fengycin production but not an iturin. Interestingly, only surfactin was identified in *B. pumilus* JJ1622, *B. safensis* JJ1244, and *B. altitudinis* JJ1138 extracts. This was consistent with the production of surfactin-like (lichenysin) compound predicted by antiSMASH. Fractionation of *B. velezensis* JJ334 extracts by HPLC followed by LC-MS analyses of lipopeptide fractions showed that bacillomycin L derivatives eluted between 1.5 and 2.8 min., while fengycin and surfactin derivatives eluted from 8.0 to 10.0 min and 13.4 to 17.4 min, respectively (Figure 6A). Mass spectrometric analyses identified the presence of six derivatives of bacillomycin L, 10 of fengycin, and eight of surfactin in the purified lipopeptide fractions (Figure 6B).

Extracts corresponding to the purified fraction of each lipopeptide were analyzed by LC-MS*^n^* employing positive and negative ionization modes (see section “Materials and methods”). Each lipopeptide compound was confirmed by the fragmented ions generated by MS*^n^* analyses (see Supplementary Tables 3–5 and Supplementary Figures 6–8). Bacillomycin L (six) and surfactin (eight) derivatives were identified each varying by the carbon chain length of the fatty-acid side chains. For both lipopeptides, derivatives were detected in both positive and negative ion modes as protonated, deprotonated, double-protonated, and/or sodium-adduct forms (Table 1). Thirteen fengycin derivatives were identified as [M+H]^+^, [M–H], and [M+2H]^2+^ ions. Four were fengycin A and two were fengycin A2 where the amino acid at position six in the core peptide was Ala, and either Ile or Val occupied position 10, respectively. Three were fengycin B and three were fengycin B2 where the amino acid at position six in the core peptide was Val, and either Ile or Val occupied position 10, respectively. Here as well, derivatives within fengycin A, A2, B, and B2 varied by fatty acid carbon chain length or amino acid in the core peptide (Table 1). There were insufficient data to identify a 13 fengycin, but its ion m/z values were consistent with C21 fengycin B or C22 fengycin B2. To our knowledge, this diversity of production of lipopeptide derivatives from a single *Bacillus* strain has not previously been observed.

**TABLE 1:** Ions of bacillomycin L, fengycin, and surfactin detected by LC-MS^n^.

Lipopeptides^1^	Derivatives^2,3^	Molecular Form.	[M+H]^+^	[M–H]	[M+2H]^2+^	[M+Na]^+^
Bacillomycin L	C13	C_45_H_70_N_10_O_16_	1007.5044	1005.4879	504.2562	1029.4858
	C14	C_46_H_72_N_10_O_16_	1021.5198	1019.5049	511.2637	1043.5019
	C15	C_47_H_74_N_10_O_16_	1035.5354	1033.5203	518.2717	1057.5178
	C16	C_48_H_76_N_10_O_16_	1049.5539	1047.5366	525.2793	1071.5327
	C17	C_49_H_78_N_10_O_16_	1063.5666	1061.5507	532.2871	1085.5472
	C18	C_50_H_80_N_10_O_16_	1077.5820	1075.5669	539.295	1099.5629
Fengycin	C14 A^4^	C_70_H_106_N_12_O_20_	1435.7730	1433.7492	718.3893	–
	C15 A/C16 A2^5^	C_71_H_108_N_12_O_20_	1449.7878	1447.7728	725.3971	–
	C16 A/C17 A2	C_72_H_110_N_12_O_20_	1463.8028	1461.7891	732.4049	–
	C17 A	C_73_H_112_N_12_O_20_	1477.8185	1475.8048	739.4134	–
	C16 B^6^/C17 B2^7^	C_74_H_114_N_12_O_20_	1491.8332	1489.8163	746.4210	–
	C17 B	C_75_H_116_N_12_O_20_	1505.8471	1503.8320	753.4286	–
	C18 B	C_76_H_118_N_12_O_20_	1519.8666	1517.8474	760.4343	–
	C20 B2	C_77_H_120_N_12_O_20_	1533.8819	1531.8637	767.4439	–
	C21 B2	C_78_H_122_N_12_O_20_	1547.8966	1545.8792	774.4518	–
	Feng. ND^8^	C_79_H_124_N_12_O_20_	1561.9106	1559.8946	781.4584	–
Surfactin	C12	C_50_H_87_N_7_O_13_	994.6428	992.6281	497.8256	1016.6242
	C13	C_51_H_89_N_7_O_13_	1008.6587	1006.6429	504.8333	1030.6397
	C14	C_52_H_91_N_7_O_13_	1022.6740	1020.6577	511.8408	1044.6560
	C15	C_53_H_93_N_7_O_13_	1036.6898	1034.6730	518.8463	1058.6716
	C16	C_54_H_95_N_7_O_13_	1050.7053	1048.6884	525.8563	1072.6881
	C17	C_55_H_97_N_7_O_13_	1064.7199	1062.7056	532.8642	1086.7013
	C18	C_56_H_99_N_7_O_13_	1078.7360	1076.7212	539.8720	1100.7178
	C19	C_57_H_101_N_7_O_13_	1092.7519	1090.7370	546.8800	1114.7327

^1^Each lipopeptide was produced by and extracted from *B. velezensis* JJ334.

^2^MS data supporting lipopeptide structure assignments are available in Supplementary materials.

^3^Lipopeptide derivatives; C12–C21 refers to fatty acid carbon chain length

^4^Fengycin A core decapeptide sequence: Glu-*^D^*Orn-*^D^*Tyr-*^D^allo*Thr-Glu-*^D^*Ala-Pro-Gln-Tyr-Ile.

^5^Fengycin A2 core decapeptide sequence: Glu-*^D^*Orn-*^D^*Tyr-*^D^allo*Thr-Glu-*^D^*Ala-Pro-Gln-Tyr-Val.

^6^Fengycin B core decapeptide sequence: Glu-*^D^*Orn-*^D^*Tyr-*^D^allo*Thr-Glu-*^D^*Val-Pro-Gln-Tyr-Ile.

^7^Fengycin B2 core decapeptide sequence: Glu-*^D^*Orn-*^D^*Tyr-*^D^allo*Thr-Glu-*^D^*Val-Pro-Gln-Tyr-Val.

^8^Feng. ND: Fengycin not fully determined; *m/z* values are consistent with C21 B or C22 D, but MS^n^ data are inconclusive.

### Inhibitory capacity of bacillomycin L, fengycin, and surfactin against *P. nicotianae* and *F. oxysporum*

**FIGURE 7:**
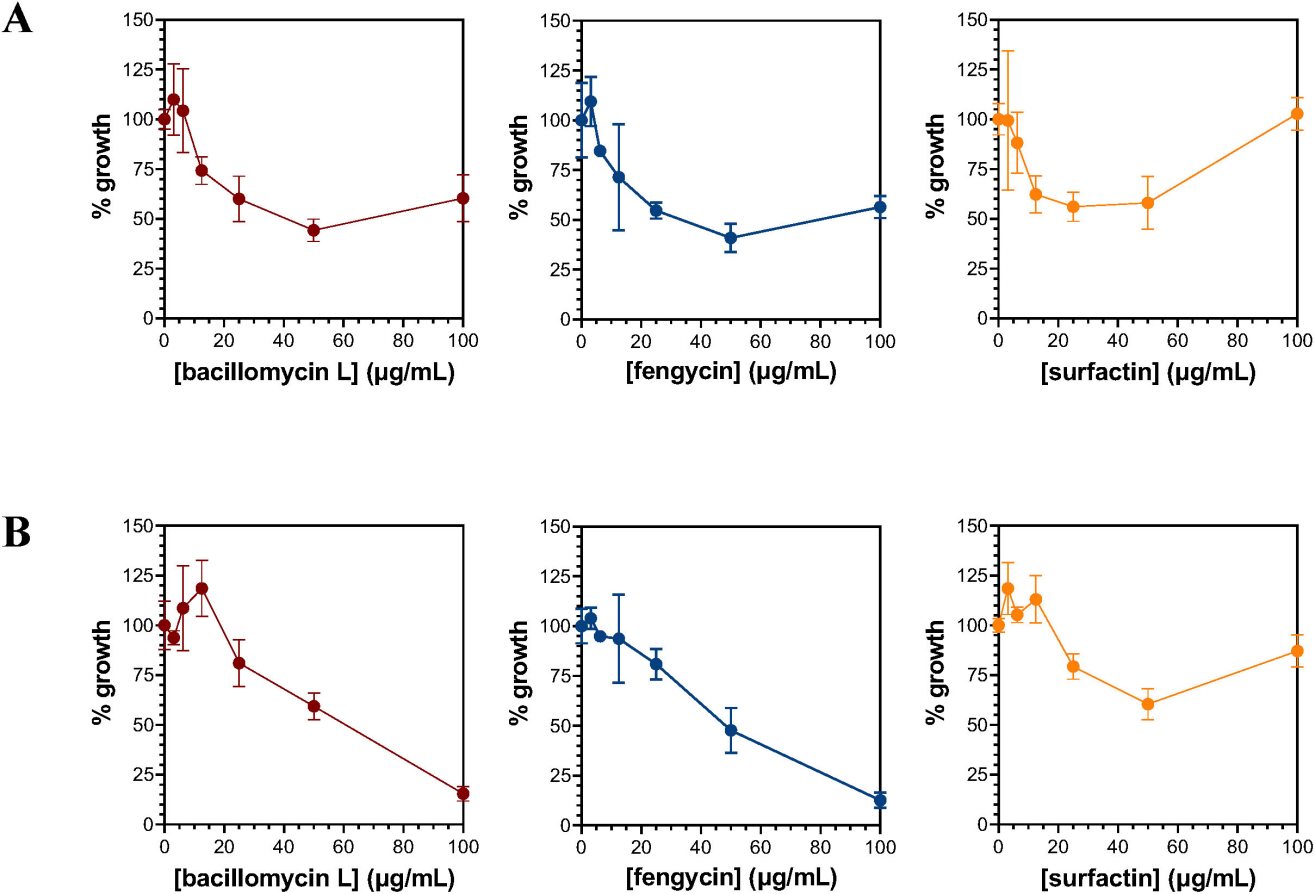
Inhibition of *P. nicotianae* **(A)** and *F. oxysporum* **(B)** by bacillomycin L, fengycin, and surfactin. Growth inhibition of *P. nicotianae* **(A)** and *F. oxysporum* **(B)** was evaluated using a microtiter plate-based assay. The bacillomycin L, fengycin, and surfactin utilized to evaluate inhibition were extracted and isolated from cultures of *B. velezensis* JJ334.

Fractions containing bacillomycin L, fengycin, and surfactin were evaluated for their ability to inhibit the growth of *P. nicotianae, F. oxysporum, F. graminearum*, and/or *R. solani* by a disk diffusion method. Interestingly, each lipopeptide fraction exhibited inhibition against all four target pathogens (Supplementary Figure 5). Considering the production of all three lipopeptides by *B. velezensis* strains Each lipopeptide was further evaluated for inhibition against *P. nicotianae* and *F. oxysporum* by a microtiter plate assay. The maximum inhibition of *P. nicotianae* growth produced by bacillomycin L and fengycin was observed at concentrations of 50 μg/mL each (Figure 7A), where 56% and 59% growth inhibition were detected, respectively. For surfactin, a similar level of *P. nicotianae* growth inhibition (∼40%) was observed at concentrations ranging from 12.5 to 50 mg/mL. Growth inhibition of *F. oxysporum* was also observed for all three lipopeptides (Figure 7B). Bacillomycin L and fengycin showed similar inhibitory potency against this organism, with significant growth inhibition observed at lipopeptide concentrations 25 mg/mL and producing 85% and 87% growth inhibition at 100 mg/mL, respectively. The maximum inhibitory effect of surfactin (∼40%) against *F. oxysporum* was observed at a concentration of 50 μg/mL.

## Discussion

Several studies have demonstrated that strains of *Bacillus* species can inhibit plant pathogens and enhance plant growth by producing various secondary metabolites (Ongena and Jacques, 2008; Fira et al., 2018; Kaspar et al., 2019; Wang et al., 2020; Kiesewalter et al., 2021). However, most studies have been conducted on a single strain or a few strains within a species against one to three pathogenic organisms. Studies of this kind naturally limit the scope of investigation on the conservation of antibiosis activity among species and the breadth of antibiosis against different pathogens. This also limits investigating whether the diversity and variation of secondary metabolites produced by different *Bacillus* species correlate with the observed bioactivity. To address this gap, we evaluated the antibiosis activity of 284 strains against *P. nicotianae* and compared this against the secondary metabolite-BGCs encoded in each genome to identify conserved factors contributing to activity. Strains strongly inhibitory against *P. nicotianae* were evaluated further for their ability to inhibit three fungal pathogens, *F. oxysporum*, *F. graminearum*, and *R. solani*.

Our results demonstrate that antibiosis against *P. nicotianae* is present primarily among five phylogenetically related species: *B. pumilus*, *B. safensis*, *B. altitudinis*, *B. velezensis*, and *B. subtilis*. *Bacillus* antibiosis conservation among strains within each species was expressed as a species-specific bioactivity index (ranging from 0 to 1) and compared against the BGCs carried by these species as determined from genome sequence analyses. This comparison showed a strong correlation between NRPS (especially lipopeptide) BGCs and the bioactivity index, suggesting that lipopeptide BGCs identified in strongly inhibitory *Bacillus* species are conserved factors that may account for a substantial portion of antibiosis activity against fungi and oomycetes. In terms of lipopeptide BGC content, there was a clear distinction between *B. velezensis* and *B. subtilis* strains on one hand and *B. pumilus*, *B. altitudinis*, and *B. safensis* strains on the other; on average, strains of the former encoded two to three times more lipopeptide BGCs than species from the latter group.

The breadth of antibiosis activity followed the same line of demarcation observed with lipopeptide BGC content. Indeed, further evaluation of strong *P. nicotianae* antagonists from *B. velezensis* and *B. subtilis* revealed that all but one *B. subtilis* strain exerted strong antibiosis activity against all three fungal pathogens evaluated (*F. graminearum, F. oxysporum*, and *R. solani*). Even the one *B. subtilis* strain (JM553) that was observed to be a strong inhibitor of *R. solani* and *F. graminearum* showed weak inhibition of *F. oxysporum*. Conversely, none of the *P. nicotianae*-antagonistic strains of *B. pumilus*, *B. safensis*, or *B. altitudinis* were able to inhibit either *Fusarium* species examined, and only weak inhibition was detected against *R. solani*.

The distribution of lipopeptide BGCs across these species showed distinct patterns that matched up well with the antibiosis phenotypes. The most prolific lipopeptide-producing species was *B. velezensis*, the strains of which consistently carry three such BGCs: an iturin, a fengycin, and a surfactin. Interestingly, the iturin and the fengycin core genes occupy the same BGC. Consistent with previous observations (Dunlap et al., 2019), our data indicate that bacillomycin L (e.g., *B. velezensis* JJ334) and iturin A (e.g., *B. velezensis* JJ951) are the two most common iturins generated by strains from this species. A broad panel of fengycins (i.e., multiple fatty acid derivatives of fengycin A, A2 B, and B2) are produced from the corresponding *B. velezensis* BGC. By comparison, *B. subtilis* strains reliably carry two lipopeptide BGCs, a fengycin-only cluster and a surfactin cluster. Finally, strains from all three of the specialist *Bacillus* species, *B. pumilus*, *B. altitudinis*, and *B. safensis*, encoded on average just one lipopeptide-generating BGC, and this BGC was predicted to be a lichenysin cluster. Though its overall structure is distinct from that of the closely related surfactin BGCs observed in our *B. velezensis* and *B. subtilis* strains, the amino-acid sequence of the core peptide predicted from the modules of the cluster’s first three core genes is Glu-Leu-*^D^*Leu-Val-Asp-*^D^*Leu-Ile. This is consistent with the surfactin-like group of lipopeptide derivatives observed from these species by LC-MS^n^ analyses. Taken together, these results suggest that production of surfactin alone may be sufficient to exert anti-*P. nicotianae* activity while antifungal activity requires production of at least fengycin in addition to surfactin. Indeed, Khan et al. have suggested that strong antifungal activity of *Bacillus* species is due to the synergistic actions of bacillomycin, fengycin, and surfactin (Khan et al., 2017).

The effectiveness of surfactin as a unilateral agent of antibiosis has varied in the literature, depending largely on the identity of the target organism(s). One study reported that surfactin directly contributed to the biological activity of *B. subtilis* GBL191 against the oomycete *Plasmopara viticola* (Li et al., 2019). In contrast, Wang et al. reported that surfactin alone did not inhibit the mycelial growth of the oomycete, *Phytophthora infestans* even at a concentration as high as 25 mg/mL (Wang et al., 2020). Another study showed that surfactin produced antibacterial activity against the Gram-negative bacterium, *Pseudomonas syringae* with a minimum inhibitory concentration of 25 mg/mL (Bais et al., 2004). Although the strong anti-*P. nicotianae* activity of *B. pumilus*, *B. safensis*, and *B. altitudinis* may not be fully explained solely by their ability to produce surfactin, we have observed that isolated surfactin is able to inhibit *P. nicotianae.* Although its maximum effect against the organism was observed at 50 mg/mL, it should be noted that very little difference in growth inhibition was observed between 12.5 and 50 mg/mL (see Figure 7A). These *Bacillus* species may produce other compounds with antagonistic activity such as betalactone, bacillibactin, and/or bacilysin along with surfactin to synergistically contribute to anti-*P. nicotianae* activity. Notably, betalactone and bacillibactin have been reported for anti-oomycete and antibacterial activity, respectively. Bacillysin has been well known for antibacterial activity until recently. Multiple investigators have demonstrated that bacilysin exhibits strong antibiosis activity against the oomycete *P. sojae* (Wu et al., 2014; Han et al., 2021). Future studies are anticipated to isolate these compounds and evaluate their antibiosis activity alone or collectively against *P. nicotianae*.

Bacillomycin L (an iturin) and fengycin are known as strongly antagonistic against plant-pathogenic fungi (Luo et al., 2015a; Jin et al., 2020; Zhang and Sun, 2018). Surfactin is known as a broad-spectrum antimicrobial that exhibits bioactivity against fungi, oomycetes, and bacteria (Ongena and Jacques, 2008; Zhao et al., 2017; Wang et al., 2020). We identified a total of six derivatives of bacillomycin L, ten derivatives of fengycin, and eight derivatives of surfactin from *B. velezensis* JJ334. We observed variation in the core peptide structure of fengycin, and we detected varying lengths of fatty acid side chains from 12 to 21 carbons, depending on the lipopeptide in question. Very few *Bacillus* species are known to produce all three lipopeptides (Ongena and Jacques, 2008), and when they are observed, identification of only a few derivatives (2–5) of each lipopeptide is typical (Luo et al., 2015b; Jasim et al., 2016; Zhang and Sun, 2018). Previous reports have suggested that the bioactivity of lipopeptides may increase along with the length of the fatty acid chain and the number of derivatives, since more derivatives may produce a synergistic effect that contributes to strong bioactivity (Zhao et al., 2017; Kaspar et al., 2019). In general, strong bioactivity was observed for bacillomycin L and fengycin whereas weak activity was observed for surfactin against oomycetes and fungi (Hu et al., 2007; Romero et al., 2007; Ongena and Jacques, 2008; Wang et al., 2020). What stands out in this study is that *B. velezensis* JJ334 produced a total of 24 lipopeptide derivatives with varying lengths of fatty acids, an impressive ability to produce that many lipopeptides from a single *B. velezensis* strain.

In summary, antibiosis activity of 284 PGPR *Bacillus* strains against *P. nicotianae* showed that antibiosis was highly associated among five species. Specifically, 55 out of 58 inhibitory strains identified came from *B. velezensis*, *B. subtilis*, *B. pumilus*, *B. safensis*, or *B. altitudinis*. Strains from closely related *B. velezensis* and *B. subtilis* also showed strong antifungal activity against multiple species. Conversely, the *B. pumilis*, *B. safensis*, and *B. altitudinis* strains capable of inhibiting *P. nicotianae* showed little to no ability to inhibit fungi. Consistent with genomic analysis, 2–3 lipopeptides (either fengycin and surfactin or bacillomycin/iturin, fengycin, and surfactin) were identified in the total extracts of *B. subtilis* and *B. velezensis*, respectively. Only surfactin was identified from *B. pumilus*, *B. safensis*, and *B. altitudinis*. All three lipopeptides showed antibiosis activity against both *P. nicotianae* and *F. oxysporum*, however, the most potent activities were observed for fengycin and bacillomycin L as compared to surfactin. The broad antibiosis activity of *B. velezensis* and *B. subtilis* is likely accounted for by their ability to produce fengycin (*B. subtilis*) and iturin + fengycin (*B. velezensis*) along with surfactin.

## Data Availability

All strain information was made publicly available at BioProject Accession: PRJNA1078443.

## Footnotes

1
https://digitalinsights.qiagen.com
