# Supplementary material for: Broad antibiosis activity of Bacillus velezensis and Bacillus subtilis is accounted for by a conserved capacity for lipopeptide biosynthesis
Source: Front Microbiol. 2025 Aug 29;16:1636481. doi: 10.3389/fmicb.2025.1636481 (PMC12426035; doi:10.3389/fmicb.2025.1636481)
Supplement: Supplementary file 2 [file Table_2.docx]

Supplementary Material

**Broad Antibiosis Activity of *Bacillus velezensis* and *Bacillus subtilis* is Accounted for by a Conserved Capacity for Lipopeptide Biosynthesis**

**Jahangir Alam,^1,4^ Oluwakemisola E. Olofintila,^2^ Francesco S. Moen,^3^ Zachary A. Noel,^2^ Mark R. Liles,^3^ Douglas C. Goodwin^1,*^**

^1^Department of Chemistry and Biochemistry, Auburn University, Auburn, AL 36849

^2^Department of Entomology and Plant Pathology, Auburn University, Auburn, AL 36849

^3^Department of Biological Sciences, Auburn University, Auburn, AL 36849

^4^Current address: Organon & Co., 727 Norristown Road, Building 4, Lower Gwynedd, PA, 19002

*** Correspondence:** Douglas C. Goodwin: goodwdc@auburn.edu

***Supplementary Material Contents***

***2-Supplementary Material Table S1 (Separate File)***

Table S1. Bacillaceae PGPR strains evaluated for biocontrol ability.

***3-Supplementary Material Figures S1 - S6 (This File)***

Figure S1. A representative assay plate showing antibiosis against *P. nicotianae*.

Figure S2. Correlations of bioactivity index and the number of BGCs from each of six major classes.

Figure S3. Clustering analysis of all 2,446 BGCs identified from the genomes of 284 Bacillaceae strains.

Figure S4. Plate-based evaluation of antibiosis exerted by total extracts of *B. velezensis* JJ334 against *P. nicotianae* and fungal pathogens.

Figure S5. Separation and spectral properties of total extracts from representative bioactive *Bacillus* species.

Figure S6. Plate-based evaluation of antibiosis exerted by isolated lipopeptides against *P. nicotianae* and fungal pathogens.

***4-Supplementary Material Table S2 (Separate File)***

Table S2. Detailed information on BGCs/secondary metabolites from Bacillaceae strains: Strain ID, BGCs, secondary metabolites, and inhibition against *Phytophthora nicotianae.*

***5-Supplementary Material Mass Spec (Separate File)***

Table S3 and Figure S7. Assignment of fengycin derivatives based on representative fragment ions generated by MS^2^ analyses.

Table S4 and Figure S8. Assignment of surfactin derivatives based on representative fragment ions generated by MS^2^ analyses.

Table S5 and Figure S9. Assignment of bacillomycin L derivatives based on representative fragment ions generated by MS^2^ analyses.


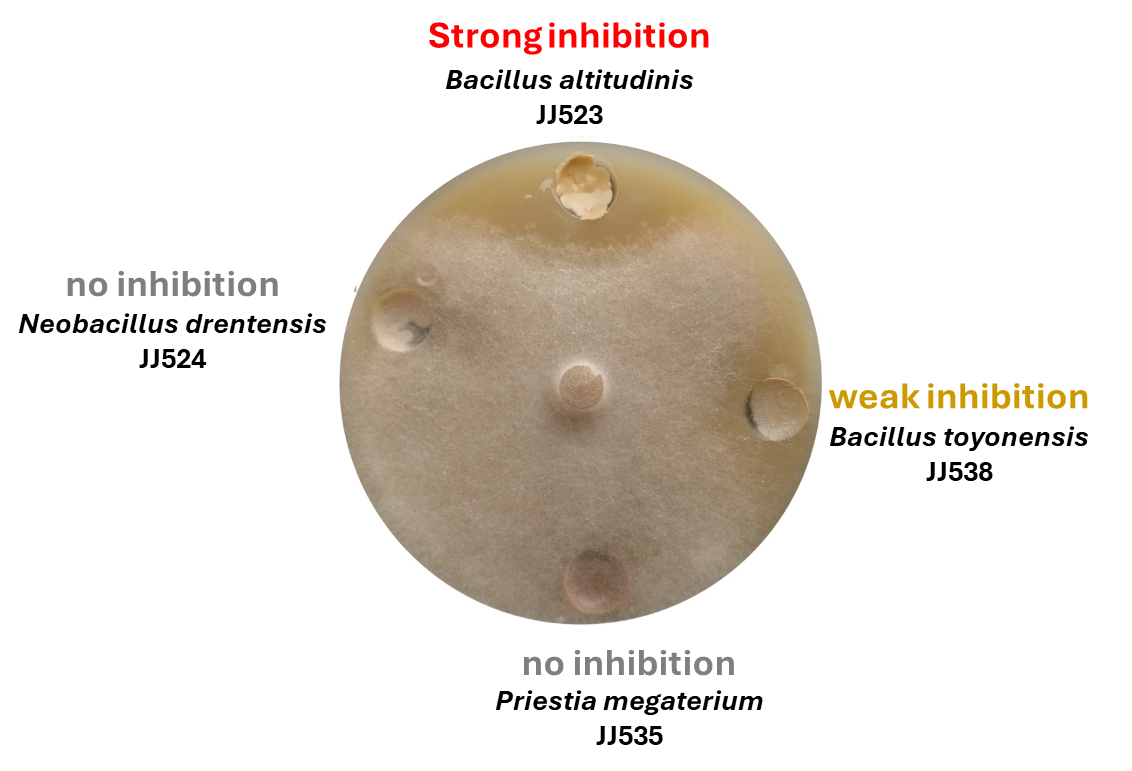


**Figure S1. Representative antibiosis assay plate showing strong, weak, and no inhibition of Bacillaceae species against the plant-pathogenic oomycete, *Phytophthora nicotianae*.**


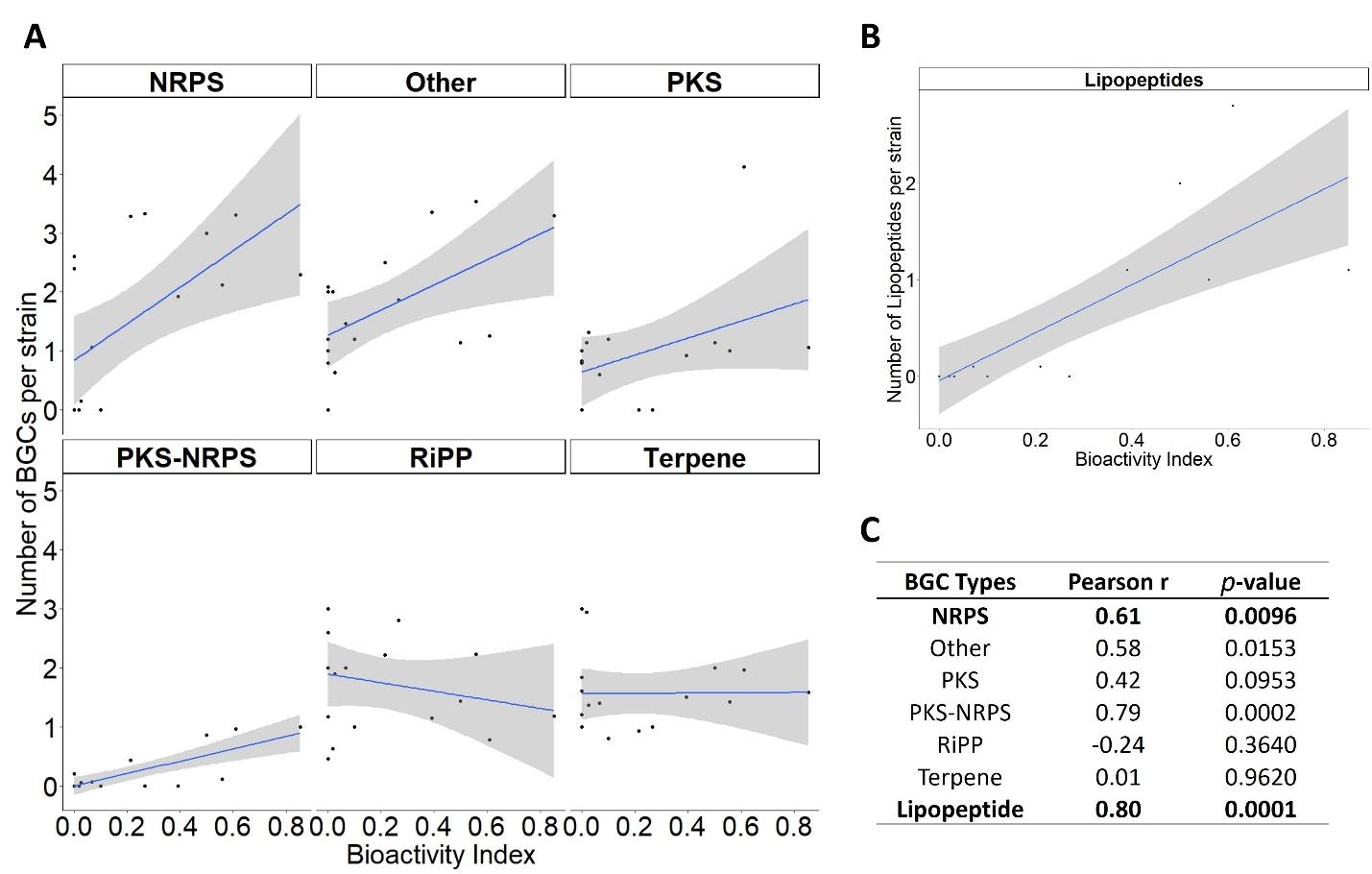


**Figure S2. Correlations of bioactivity index and the number of BGCs from each of six major classes** (A) and lipopeptide BGCs in particular (B). The average number of a given BGCs per strain within a given species were plotted against the bioactivity index expressed by that same species. The correlational parameters (Pearson r and *p*-value) for the six general BGC classes and specific lipopeptides are shown (C).


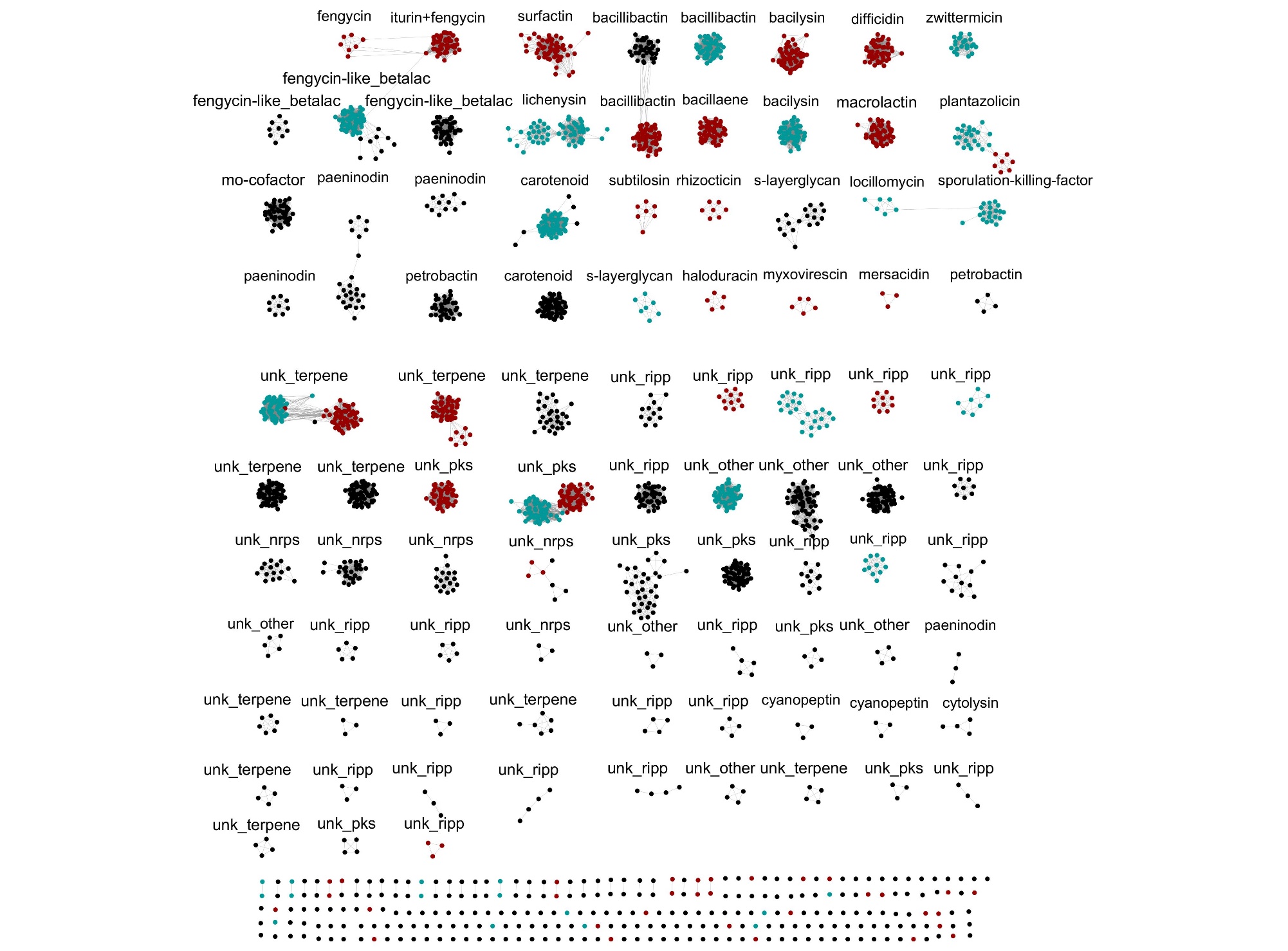


**Figure S3. Clustering analysis of all 2,446 BGCs identified from the genomes of 284 Bacillaceae strains.** Strains are color-coded according to their grouping among antibiosis generalists (red), antibiosis specialists (teal), or non-inhibitors (black).

**Figure S4. Plate-based evaluation of antibiosis exerted by total extracts of *B. velezensis* JJ334 against *P. nicotianae* and fungal pathogens.** Antibiosis activity of intact *B. velezensis* JJ334 against the four pathogens is shown across the top. Disks loaded with total extracts from *B. velezensis* JJ334 are shown across the middle. Extracts were generated according to the acid-methanolic procedure described in Materials and Methods. Disks loaded with methanol alone are shown across the bottom as a control.

**
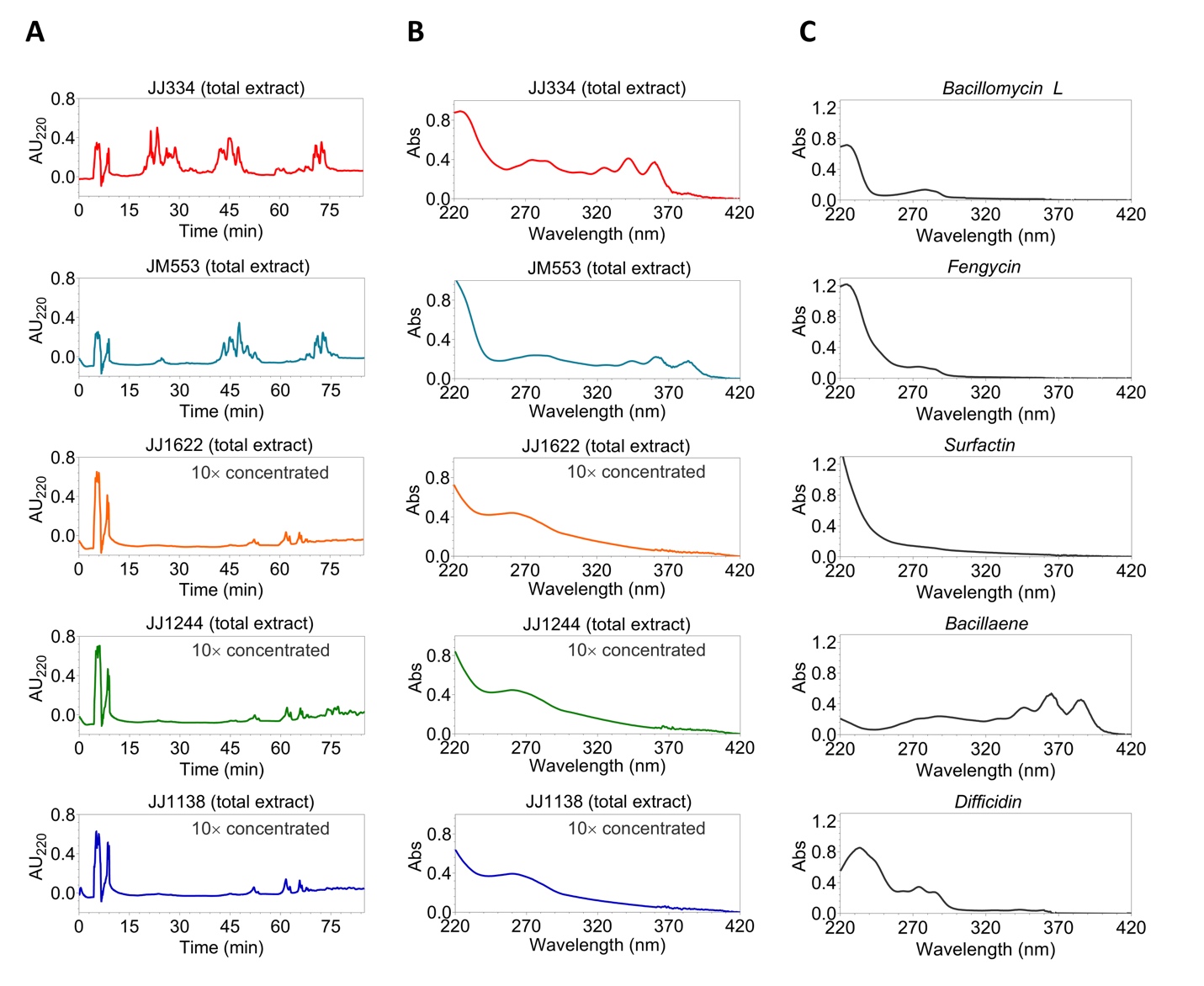
**

**Figure S5. Separation and spectral properties of total extracts from representative bioactive *Bacillus* species.** A typical LC chromatogram of total extraction of secondary metabolites produced by *B. velezensis* JJ334, *B. subtilis* JM553, *B. pumilus* JJ1622, *B. safensis* JJ1244, and *B. altitudinis* JJ1138. Elution of metabolites was monitored at 220 nm. The UV-vis absorption spectra of total extracts from cultures of each representative strain are shown (B). Diode array-captured spectra LC separations of five purified compounds: Bacillomycin L, fengycin, surfactin, bacillaene and difficidin.

**
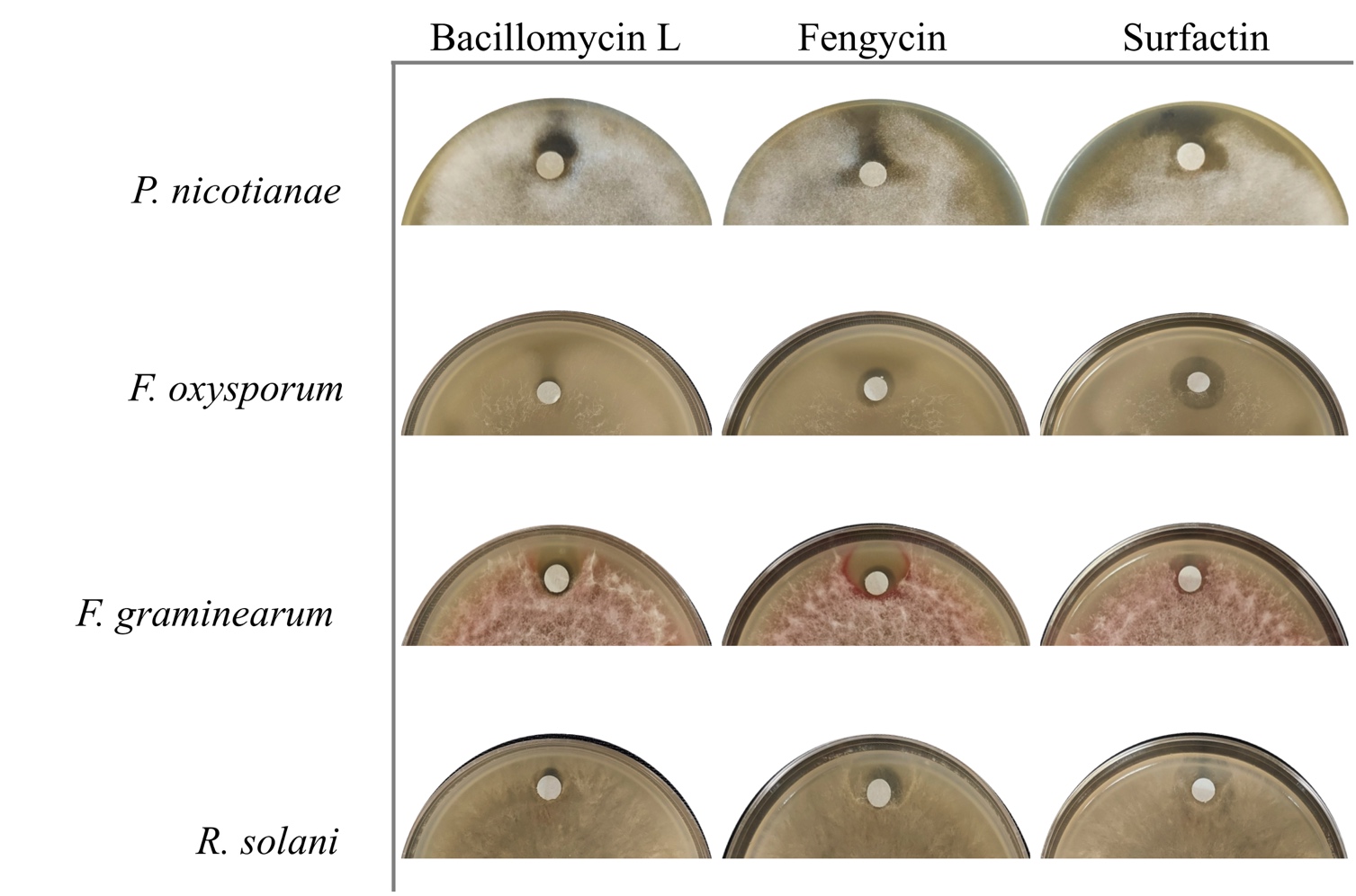
 Figure S6. Plate-based evaluation of antibiosis exerted by isolated lipopeptides against *P. nicotianae* and fungal pathogens.** A disk-diffusion assay was performed to observe antibiosis activity of bacillomycin L, fengycin, and surfactin against *P. nicotianae* and three fungal pathogens: *F. oxysporum*, *F. graminearum*, and *R. solani*.
