# Supplementary material for: Broad antibiosis activity of Bacillus velezensis and Bacillus subtilis is accounted for by a conserved capacity for lipopeptide biosynthesis
Source: Front Microbiol. 2025 Aug 29;16:1636481. doi: 10.3389/fmicb.2025.1636481 (PMC12426035; doi:10.3389/fmicb.2025.1636481)
Supplement: Supplementary file 3 [file Table_3.docx]

***​​Supplementary Material***

**​Broad Antibiosis Activity of *Bacillus velezensis* and *Bacillus subtilis* is Accounted for by a Conserved Capacity for Lipopeptide Biosynthesis**

**​Jahangir Alam,^1,4^ Oluwakemisola E. Olofintila,^2^ Francesco S. Moen,^3^ Zachary A. Noel,^2^ Mark R. Liles,^3^ Douglas C. Goodwin^1*^**

​^1^Department of Chemistry and Biochemistry, Auburn University, Auburn, AL 36849

​^2^Department of Entomology and Plant Pathology, Auburn University, Auburn, AL 36849

​^3^Department of Biological Sciences, Auburn University, Auburn, AL 36849

^4^Current address: Organon & Co., 727 Norristown Road, Bldg. 4, Lower Gwynedd PA 19002

**​* Correspondence:** Douglas C. Goodwin: goodwdc@auburn.edu

**​** ***Supplementary Material Contents***

***2-Supplementary Material Table S1 (Separate File)***

Table S1. Bacillaceae PGPR strains evaluated for biocontrol ability.

***3-Supplementary Material Figures S1 - S6 (Separate File)***

Figure S1. A representative assay plate showing antibiosis against *P. nicotianae*.

Figure S2. Correlations of bioactivity index and the number of BGCs from each of six major classes.

Figure S3. Clustering analysis of all 2,446 BGCs identified from the genomes of 284 Bacillaceae strains.

Figure S4. Plate-based evaluation of antibiosis exerted by total extracts of *B. velezensis* JJ334 against *P. nicotianae* and fungal pathogens.

Figure S5. Separation and spectral properties of total extracts from representative bioactive *Bacillus* species.

Figure S6. Plate-based evaluation of antibiosis exerted by isolated lipopeptides against *P. nicotianae* and fungal pathogens.

***4-Supplementary Material Table S2 (This File)***

Table S2. Detailed information on BGCs/secondary metabolites from Bacillaceae strains: Strain ID, BGCs, secondary metabolites, and inhibition against *Phytophthora nicotianae.*

***5-Supplementary Material Mass Spec (Separate File)***

Table S3 and Figure S7. Assignment of fengycin derivatives based on representative fragment ions generated by MS^2^ analyses.

Table S4 and Figure S8. Assignment of surfactin derivatives based on representative fragment ions generated by MS^2^ analyses.

Table S5 and Figure S9. Assignment of bacillomycin L derivatives based on representative fragment ions generated by MS^2^ analyses.

| **Strain ID** | **Bacillus Species** | **P. nicotianae Inhibition** | **Species Group** | **BGC Class** | **BGC Class (antiSMASH)** | **Secondary Metabolites** | **% Similarity** |
| --- | --- | --- | --- | --- | --- | --- | --- |
| AB01 | *Bacillus velezensis* | strong | Strong | NRPS | NRPS | surfactin | 82 |
| AB01 | *Bacillus velezensis* | strong | Strong | PKS | PKS-like | butirosin A | 7 |
| AB01 | *Bacillus velezensis* | strong | Strong | Terpene | terpene | unknown | ND |
| AB01 | *Bacillus velezensis* | strong | Strong | RiPP | lanthipeptide-class-ii | unknown | ND |
| AB01 | *Bacillus velezensis* | strong | Strong | PKS | transAT-PKS | macrolactin H | 100 |
| AB01 | *Bacillus velezensis* | strong | Strong | PKS-NRPS | transAT-PKS | bacillaene | 100 |
| AB01 | *Bacillus velezensis* | strong | Strong | NRPS | NRPS | fengycin | 100 |
| AB01 | *Bacillus velezensis* | strong | Strong | Terpene | terpene | unknown | ND |
| AB01 | *Bacillus velezensis* | strong | Strong | PKS | T3PKS | unknown | ND |
| AB01 | *Bacillus velezensis* | strong | Strong | PKS | transAT-PKS | difficidin | 100 |
| AB01 | *Bacillus velezensis* | strong | Strong | NRPS | NRPS | bacillibactin | 100 |
| AB01 | *Bacillus velezensis* | strong | Strong | Other | other | bacilysin | 100 |
| AB01 | *Bacillus velezensis* | strong | Strong | NRPS | Others | iturin/Bacilomycin L | 100 |
| AP150 | *Bacillus velezensis* | strong | Strong | PKS-NRPS | transAT-PKS-like | bacillaene | 100 |
| AP150 | *Bacillus velezensis* | strong | Strong | NRPS | NRPS | bacillibactin | 100 |
| AP150 | *Bacillus velezensis* | strong | Strong | Other | other | bacilysin | 100 |
| AP150 | *Bacillus velezensis* | strong | Strong | PKS | transAT-PKS-like | difficidin | 100 |
| AP150 | *Bacillus velezensis* | strong | Strong | NRPS | NRPS | fengycin | 80 |
| AP150 | *Bacillus velezensis* | strong | Strong | RiPP | lanthipeptide | haloduracin | 40 |
| AP150 | *Bacillus velezensis* | strong | Strong | PKS | transAT-PKS | macrolactin H | 100 |
| AP150 | *Bacillus velezensis* | strong | Strong | PKS | PKS-like | unknown | ND |
| AP150 | *Bacillus velezensis* | strong | Strong | Terpene | terpene | unknown | ND |
| AP150 | *Bacillus velezensis* | strong | Strong | Terpene | terpene | unknown | ND |
| AP150 | *Bacillus velezensis* | strong | Strong | NRPS | NRPS | unknown | ND |
| AP150 | *Bacillus velezensis* | strong | Strong | RiPP | LAP | plantazolicin | 41 |
| AP150 | *Bacillus velezensis* | strong | Strong | NRPS | NRPS | rhizocticin A | 6 |
| AP150 | *Bacillus velezensis* | strong | Strong | NRPS | NRPS | surfactin | 47 |
| AP150 | *Bacillus velezensis* | strong | Strong | PKS | T3PKS | unknown | ND |
| AP150 | *Bacillus velezensis* | strong | Strong | NRPS | Others | iturin/Bacilomycin L | 80 |
| AP183 | *Bacillus velezensis* | strong | Strong | PKS | T1PKS | aurantinin B | 25 |
| AP183 | *Bacillus velezensis* | strong | Strong | PKS-NRPS | transAT-PKS | bacillaene | 100 |
| AP183 | *Bacillus velezensis* | strong | Strong | NRPS | NRPS | bacillibactin | 100 |
| AP183 | *Bacillus velezensis* | strong | Strong | Other | other | bacilysin | 100 |
| AP183 | *Bacillus velezensis* | strong | Strong | PKS | PKS-like | butirosin A | 7 |
| AP183 | *Bacillus velezensis* | strong | Strong | PKS | transAT-PKS-like | difficidin | 100 |
| AP183 | *Bacillus velezensis* | strong | Strong | NRPS | NRPS | fengycin | 100 |
| AP183 | *Bacillus velezensis* | strong | Strong | PKS | transAT-PKS | macrolactin H | 100 |
| AP183 | *Bacillus velezensis* | strong | Strong | PKS | T3PKS | unknown | ND |
| AP183 | *Bacillus velezensis* | strong | Strong | Terpene | terpene | unknown | ND |
| AP183 | *Bacillus velezensis* | strong | Strong | Terpene | terpene | unknown | ND |
| AP183 | *Bacillus velezensis* | strong | Strong | NRPS | NRPS | surfactin | 86 |
| AP183 | *Bacillus velezensis* | strong | Strong | NRPS | Others | iturin/Bacilomycin L | 100 |
| AP189 | *Bacillus velezensis* | weak | Strong | PKS-NRPS | transAT-PKS | bacillaene | 100 |
| AP189 | *Bacillus velezensis* | weak | Strong | NRPS | NRPS | bacillibactin | 100 |
| AP189 | *Bacillus velezensis* | weak | Strong | Other | other | bacilysin | 100 |
| AP189 | *Bacillus velezensis* | weak | Strong | PKS | PKS-like | butirosin A | 7 |
| AP189 | *Bacillus velezensis* | weak | Strong | PKS | transAT-PKS-like | difficidin | 53 |
| AP189 | *Bacillus velezensis* | weak | Strong | NRPS | NRPS | fengycin | 86 |
| AP189 | *Bacillus velezensis* | weak | Strong | RiPP | lanthipeptide | kijanimicin | 4 |
| AP189 | *Bacillus velezensis* | weak | Strong | PKS | transAT-PKS | macrolactin H | 100 |
| AP189 | *Bacillus velezensis* | weak | Strong | PKS | T3PKS | unknown | ND |
| AP189 | *Bacillus velezensis* | weak | Strong | Terpene | terpene | unknown | ND |
| AP189 | *Bacillus velezensis* | weak | Strong | Terpene | terpene | unknown | ND |
| AP189 | *Bacillus velezensis* | weak | Strong | NRPS | NRPS | unknown | ND |
| AP189 | *Bacillus velezensis* | weak | Strong | NRPS | NRPS | rhizocticin A | 6 |
| AP189 | *Bacillus velezensis* | weak | Strong | NRPS | NRPS | surfactin | 47 |
| AP189 | *Bacillus velezensis* | weak | Strong | NRPS | Others | iturin/Bacilomycin L | 86 |
| AP194 | *Bacillus velezensis* | strong | Strong | PKS-NRPS | transAT-PKS | bacillaene | 100 |
| AP194 | *Bacillus velezensis* | strong | Strong | NRPS | NRPS | bacillibactin | 100 |
| AP194 | *Bacillus velezensis* | strong | Strong | Other | other | bacilysin | 100 |
| AP194 | *Bacillus velezensis* | strong | Strong | PKS | PKS-like | butirosin A | 7 |
| AP194 | *Bacillus velezensis* | strong | Strong | PKS | transAT-PKS-like | difficidin | 53 |
| AP194 | *Bacillus velezensis* | strong | Strong | NRPS | NRPS | fengycin | 80 |
| AP194 | *Bacillus velezensis* | strong | Strong | RiPP | lanthipeptide | kijanimicin | 4 |
| AP194 | *Bacillus velezensis* | strong | Strong | PKS | transAT-PKS | macrolactin H | 100 |
| AP194 | *Bacillus velezensis* | strong | Strong | PKS | T3PKS | unknown | ND |
| AP194 | *Bacillus velezensis* | strong | Strong | Terpene | terpene | unknown | ND |
| AP194 | *Bacillus velezensis* | strong | Strong | Terpene | terpene | unknown | ND |
| AP194 | *Bacillus velezensis* | strong | Strong | NRPS | NRPS | rhizocticin A | 16 |
| AP194 | *Bacillus velezensis* | strong | Strong | NRPS | NRPS | surfactin | 91 |
| AP194 | *Bacillus velezensis* | strong | Strong | NRPS | Others | iturin/Bacilomycin L | 80 |
| AP202 | *Bacillus velezensis* | strong | Strong | NRPS | NRPS | surfactin | 91 |
| AP202 | *Bacillus velezensis* | strong | Strong | RiPP | RRE-containing | plantazolicin | 91 |
| AP202 | *Bacillus velezensis* | strong | Strong | PKS | PKS-like | butirosin A | 7 |
| AP202 | *Bacillus velezensis* | strong | Strong | Terpene | terpene | unknown | ND |
| AP202 | *Bacillus velezensis* | strong | Strong | PKS | transAT-PKS | macrolactin H | 100 |
| AP202 | *Bacillus velezensis* | strong | Strong | PKS-NRPS | transAT-PKS | bacillaene | 100 |
| AP202 | *Bacillus velezensis* | strong | Strong | NRPS | NRPS | fengycin | 100 |
| AP202 | *Bacillus velezensis* | strong | Strong | Terpene | terpene | unknown | ND |
| AP202 | *Bacillus velezensis* | strong | Strong | PKS | T3PKS | unknown | ND |
| AP202 | *Bacillus velezensis* | strong | Strong | PKS | transAT-PKS | difficidin | 100 |
| AP202 | *Bacillus velezensis* | strong | Strong | NRPS | NRPS | bacillibactin | 100 |
| AP202 | *Bacillus velezensis* | strong | Strong | NRPS | NRPS | unknown | ND |
| AP202 | *Bacillus velezensis* | strong | Strong | Other | other | bacilysin | 100 |
| AP202 | *Bacillus velezensis* | strong | Strong | NRPS | Others | iturin/Bacilomycin L | 100 |
| AP214 | *Bacillus velezensis* | weak | Strong | PKS-NRPS | transAT-PKS-like | bacillaene | 100 |
| AP214 | *Bacillus velezensis* | weak | Strong | NRPS | NRPS | bacillibactin | 100 |
| AP214 | *Bacillus velezensis* | weak | Strong | Other | other | bacilysin | 100 |
| AP214 | *Bacillus velezensis* | weak | Strong | PKS | PKS-like | butirosin A | 7 |
| AP214 | *Bacillus velezensis* | weak | Strong | PKS | transAT-PKS | difficidin | 60 |
| AP214 | *Bacillus velezensis* | weak | Strong | NRPS | NRPS | fengycin | 80 |
| AP214 | *Bacillus velezensis* | weak | Strong | PKS | transAT-PKS | macrolactin H | 100 |
| AP214 | *Bacillus velezensis* | weak | Strong | PKS | T3PKS | unknown | ND |
| AP214 | *Bacillus velezensis* | weak | Strong | Terpene | terpene | unknown | ND |
| AP214 | *Bacillus velezensis* | weak | Strong | Terpene | terpene | unknown | ND |
| AP214 | *Bacillus velezensis* | weak | Strong | NRPS | NRPS | rhizocticin A | 22 |
| AP214 | *Bacillus velezensis* | weak | Strong | NRPS | NRPS | surfactin | 47 |
| AP214 | *Bacillus velezensis* | weak | Strong | NRPS | Others | iturin/Bacilomycin L | 80 |
| AP215 | *Bacillus velezensis* | weak | Strong | NRPS | NRPS | surfactin | 82 |
| AP215 | *Bacillus velezensis* | weak | Strong | PKS | PKS-like | butirosin A | 7 |
| AP215 | *Bacillus velezensis* | weak | Strong | Terpene | terpene | unknown | ND |
| AP215 | *Bacillus velezensis* | weak | Strong | RiPP | lanthipeptide-class-ii | unknown | ND |
| AP215 | *Bacillus velezensis* | weak | Strong | PKS | transAT-PKS | macrolactin H | 100 |
| AP215 | *Bacillus velezensis* | weak | Strong | PKS-NRPS | transAT-PKS | bacillaene | 100 |
| AP215 | *Bacillus velezensis* | weak | Strong | NRPS | NRPS | fengycin | 100 |
| AP215 | *Bacillus velezensis* | weak | Strong | Terpene | terpene | unknown | ND |
| AP215 | *Bacillus velezensis* | weak | Strong | PKS | T3PKS | unknown | ND |
| AP215 | *Bacillus velezensis* | weak | Strong | PKS | transAT-PKS | difficidin | 100 |
| AP215 | *Bacillus velezensis* | weak | Strong | NRPS | NRPS | bacillibactin | 100 |
| AP215 | *Bacillus velezensis* | weak | Strong | Other | other | bacilysin | 100 |
| AP215 | *Bacillus velezensis* | weak | Strong | NRPS | Others | iturin/Bacilomycin L | 100 |
| AP216 | *Bacillus velezensis* | weak | Strong | PKS | T3PKS | aurantinin B | 7 |
| AP216 | *Bacillus velezensis* | weak | Strong | PKS-NRPS | transAT-PKS | bacillaene | 100 |
| AP216 | *Bacillus velezensis* | weak | Strong | NRPS | bacteriocin | bacillibactin | 100 |
| AP216 | *Bacillus velezensis* | weak | Strong | Other | other | bacilysin | 100 |
| AP216 | *Bacillus velezensis* | weak | Strong | PKS | PKS-like | butirosin A | 7 |
| AP216 | *Bacillus velezensis* | weak | Strong | PKS | transAT-PKS-like | difficidin | 53 |
| AP216 | *Bacillus velezensis* | weak | Strong | PKS | transAT-PKS-like | elansolid A | 15 |
| AP216 | *Bacillus velezensis* | weak | Strong | NRPS | NRPS | fengycin | 86 |
| AP216 | *Bacillus velezensis* | weak | Strong | PKS | T1PKS | macrobrevin | 26 |
| AP216 | *Bacillus velezensis* | weak | Strong | PKS | transAT-PKS | macrolactin H | 100 |
| AP216 | *Bacillus velezensis* | weak | Strong | PKS | T3PKS | unknown | ND |
| AP216 | *Bacillus velezensis* | weak | Strong | Terpene | terpene | unknown | ND |
| AP216 | *Bacillus velezensis* | weak | Strong | Terpene | terpene | unknown | ND |
| AP216 | *Bacillus velezensis* | weak | Strong | PKS | transAT-PKS-like | unknown | ND |
| AP216 | *Bacillus velezensis* | weak | Strong | NRPS | NRPS | surfactin | 86 |
| AP216 | *Bacillus velezensis* | weak | Strong | NRPS | Others | iturin/Bacilomycin L | 86 |
| AP297 | *Bacillus velezensis* | no | Strong | PKS-NRPS | transAT-PKS-like | bacillaene | 100 |
| AP297 | *Bacillus velezensis* | no | Strong | NRPS | bacteriocin | bacillibactin | 100 |
| AP297 | *Bacillus velezensis* | no | Strong | Other | other | bacilysin | 100 |
| AP297 | *Bacillus velezensis* | no | Strong | PKS | PKS-like | butirosin A | 7 |
| AP297 | *Bacillus velezensis* | no | Strong | PKS | transAT-PKS-like | difficidin | 53 |
| AP297 | *Bacillus velezensis* | no | Strong | Other | betalactone | fengycin | 86 |
| AP297 | *Bacillus velezensis* | no | Strong | PKS | transAT-PKS | macrolactin H | 100 |
| AP297 | *Bacillus velezensis* | no | Strong | PKS | T3PKS | unknown | ND |
| AP297 | *Bacillus velezensis* | no | Strong | RiPP | thiopeptide | unknown | ND |
| AP297 | *Bacillus velezensis* | no | Strong | Terpene | terpene | unknown | ND |
| AP297 | *Bacillus velezensis* | no | Strong | Terpene | terpene | unknown | ND |
| AP297 | *Bacillus velezensis* | no | Strong | NRPS | NRPS | surfactin | 47 |
| AP45 | *Bacillus velezensis* | strong | Strong | NRPS | NRPS | surfactin | 86 |
| AP45 | *Bacillus velezensis* | strong | Strong | PKS | T1PKS | myxovirescin A | 21 |
| AP45 | *Bacillus velezensis* | strong | Strong | PKS | PKS-like | butirosin A | 7 |
| AP45 | *Bacillus velezensis* | strong | Strong | Terpene | terpene | unknown | ND |
| AP45 | *Bacillus velezensis* | strong | Strong | PKS | transAT-PKS | macrolactin H | 100 |
| AP45 | *Bacillus velezensis* | strong | Strong | PKS-NRPS | transAT-PKS | bacillaene | 100 |
| AP45 | *Bacillus velezensis* | strong | Strong | NRPS | NRPS | fengycin | 100 |
| AP45 | *Bacillus velezensis* | strong | Strong | Terpene | terpene | unknown | ND |
| AP45 | *Bacillus velezensis* | strong | Strong | PKS | T3PKS | unknown | ND |
| AP45 | *Bacillus velezensis* | strong | Strong | PKS | transAT-PKS | difficidin | 100 |
| AP45 | *Bacillus velezensis* | strong | Strong | NRPS | NRPS | bacillibactin | 100 |
| AP45 | *Bacillus velezensis* | strong | Strong | Other | other | bacilysin | 100 |
| AP45 | *Bacillus velezensis* | strong | Strong | NRPS | Others | iturin/Bacilomycin L | 100 |
| AP46 | *Bacillus velezensis* | strong | Strong | NRPS | NRPS | rhizomide A | 22 |
| AP46 | *Bacillus velezensis* | strong | Strong | NRPS | NRPS | surfactin | 91 |
| AP46 | *Bacillus velezensis* | strong | Strong | Other | cyclic-lactone-autoinducer | kijanimicin | 4 |
| AP46 | *Bacillus velezensis* | strong | Strong | PKS | PKS-like | butirosin A | 7 |
| AP46 | *Bacillus velezensis* | strong | Strong | Terpene | terpene | unknown | ND |
| AP46 | *Bacillus velezensis* | strong | Strong | PKS | transAT-PKS | macrolactin H | 100 |
| AP46 | *Bacillus velezensis* | strong | Strong | PKS-NRPS | transAT-PKS | bacillaene | 100 |
| AP46 | *Bacillus velezensis* | strong | Strong | NRPS | NRPS | fengycin | 100 |
| AP46 | *Bacillus velezensis* | strong | Strong | Terpene | terpene | micrococcin P | 8 |
| AP46 | *Bacillus velezensis* | strong | Strong | PKS | T3PKS | unknown | ND |
| AP46 | *Bacillus velezensis* | strong | Strong | PKS | transAT-PKS | difficidin | 100 |
| AP46 | *Bacillus velezensis* | strong | Strong | NRPS | NRPS | bacillibactin | 100 |
| AP46 | *Bacillus velezensis* | strong | Strong | Other | other | bacilysin | 100 |
| AP46 | *Bacillus velezensis* | strong | Strong | NRPS | Others | iturin/Bacilomycin L | 100 |
| AP50 | *Bacillus (other)* | no | No | NRPS | NRPS | bacillibactin | 46 |
| AP50 | *Bacillus (other)* | no | No | NRPS | NRPS | bogorol A | 22 |
| AP50 | *Bacillus (other)* | no | No | Other | betalactone | fengycin | 40 |
| AP50 | *Bacillus (other)* | no | No | Terpene | terpene | molybdenum cofactor | 17 |
| AP50 | *Bacillus (other)* | no | No | NRPS | NRPS | unknown | ND |
| AP50 | *Bacillus (other)* | no | No | RiPP | LAP | unknown | ND |
| AP50 | *Bacillus (other)* | no | No | NRPS | NRPS-like | unknown | ND |
| AP50 | *Bacillus (other)* | no | No | NRPS | NRPS | unknown | ND |
| AP50 | *Bacillus (other)* | no | No | Other | siderophore | petrobactin | 100 |
| AP50 | *Bacillus (other)* | no | No | RiPP | lanthipeptide | pseudomycoicidin | 100 |
| AP50 | *Bacillus (other)* | no | No | PKS-NRPS | T1PKS | zwittermicin A | 55 |
| AP52 | *Bacillus velezensis* | strong | Strong | RiPP | thiopeptide | unknown | ND |
| AP52 | *Bacillus velezensis* | strong | Strong | NRPS | NRPS | surfactin | 91 |
| AP52 | *Bacillus velezensis* | strong | Strong | PKS | PKS-like | butirosin A | 7 |
| AP52 | *Bacillus velezensis* | strong | Strong | Terpene | terpene | unknown | ND |
| AP52 | *Bacillus velezensis* | strong | Strong | PKS | transAT-PKS | macrolactin H | 100 |
| AP52 | *Bacillus velezensis* | strong | Strong | PKS-NRPS | transAT-PKS | bacillaene | 100 |
| AP52 | *Bacillus velezensis* | strong | Strong | NRPS | NRPS | fengycin | 100 |
| AP52 | *Bacillus velezensis* | strong | Strong | Terpene | terpene | unknown | ND |
| AP52 | *Bacillus velezensis* | strong | Strong | PKS | T3PKS | unknown | ND |
| AP52 | *Bacillus velezensis* | strong | Strong | PKS | transAT-PKS | difficidin | 100 |
| AP52 | *Bacillus velezensis* | strong | Strong | NRPS | NRPS | bacillibactin | 100 |
| AP52 | *Bacillus velezensis* | strong | Strong | Other | other | bacilysin | 100 |
| AP52 | *Bacillus velezensis* | strong | Strong | NRPS | Others | iturin/Bacilomycin L | 100 |
| AP78 | *Bacillus velezensis* | strong | Strong | PKS-NRPS | transAT-PKS-like | bacillaene | 100 |
| AP78 | *Bacillus velezensis* | strong | Strong | NRPS | bacteriocin | bacillibactin | 100 |
| AP78 | *Bacillus velezensis* | strong | Strong | Other | other | bacilysin | 100 |
| AP78 | *Bacillus velezensis* | strong | Strong | PKS | PKS-like | butirosin A | 7 |
| AP78 | *Bacillus velezensis* | strong | Strong | PKS | transAT-PKS-like | difficidin | 100 |
| AP78 | *Bacillus velezensis* | strong | Strong | Other | betalactone | fengycin | 86 |
| AP78 | *Bacillus velezensis* | strong | Strong | PKS | transAT-PKS | macrolactin H | 100 |
| AP78 | *Bacillus velezensis* | strong | Strong | Terpene | terpene | unknown | ND |
| AP78 | *Bacillus velezensis* | strong | Strong | PKS | T3PKS | unknown | ND |
| AP78 | *Bacillus velezensis* | strong | Strong | Terpene | terpene | unknown | ND |
| AP78 | *Bacillus velezensis* | strong | Strong | RiPP | thiopeptide | unknown | ND |
| AP78 | *Bacillus velezensis* | strong | Strong | NRPS | NRPS | surfactin | 91 |
| AP81 | *Bacillus velezensis* | strong | Strong | NRPS | NRPS | surfactin | 86 |
| AP81 | *Bacillus velezensis* | strong | Strong | PKS | T1PKS | myxovirescin A | 17 |
| AP81 | *Bacillus velezensis* | strong | Strong | PKS | PKS-like | butirosin A | 7 |
| AP81 | *Bacillus velezensis* | strong | Strong | Terpene | terpene | unknown | ND |
| AP81 | *Bacillus velezensis* | strong | Strong | PKS | transAT-PKS | macrolactin H | 100 |
| AP81 | *Bacillus velezensis* | strong | Strong | PKS-NRPS | transAT-PKS | bacillaene | 100 |
| AP81 | *Bacillus velezensis* | strong | Strong | NRPS | NRPS | fengycin | 100 |
| AP81 | *Bacillus velezensis* | strong | Strong | Terpene | terpene | unknown | ND |
| AP81 | *Bacillus velezensis* | strong | Strong | PKS | T3PKS | unknown | ND |
| AP81 | *Bacillus velezensis* | strong | Strong | PKS | transAT-PKS | difficidin | 100 |
| AP81 | *Bacillus velezensis* | strong | Strong | NRPS | NRPS | bacillibactin | 100 |
| AP81 | *Bacillus velezensis* | strong | Strong | Other | other | bacilysin | 100 |
| AP81 | *Bacillus velezensis* | strong | Strong | NRPS | Others | iturin/Bacilomycin L | 100 |
| JJ1003 | *Priestia megaterium* | no | No | Terpene | terpene | carotenoid | 50 |
| JJ1003 | *Priestia megaterium* | no | No | Other | phosphonate | unknown | ND |
| JJ1003 | *Priestia megaterium* | no | No | Terpene | terpene | unknown | ND |
| JJ1003 | *Priestia megaterium* | no | No | PKS | T3PKS | unknown | ND |
| JJ1003 | *Priestia megaterium* | no | No | Other | siderophore | unknown | ND |
| JJ1003 | *Priestia megaterium* | no | No | Other | siderophore | unknown | ND |
| JJ1019 | *Bacillus (other)* | no | No | Terpene | terpene | carotenoid | 50 |
| JJ1019 | *Bacillus (other)* | no | No | Other | phosphonate | unknown | ND |
| JJ1019 | *Bacillus (other)* | no | No | Terpene | terpene | unknown | ND |
| JJ1019 | *Bacillus (other)* | no | No | PKS | T3PKS | unknown | ND |
| JJ1019 | *Bacillus (other)* | no | No | Other | siderophore | unknown | ND |
| JJ1019 | *Bacillus (other)* | no | No | Other | siderophore | unknown | ND |
| JJ1019 | *Bacillus (other)* | no | No | Terpene | terpene | surfactin | 13 |
| JJ1027 | *Gottfriedia acidiceleris* | no | No | RiPP | LAP | unknown | ND |
| JJ1027 | *Gottfriedia acidiceleris* | no | No | Terpene | terpene | unknown | ND |
| JJ1027 | *Gottfriedia acidiceleris* | no | No | Terpene | terpene | unknown | ND |
| JJ1027 | *Gottfriedia acidiceleris* | no | No | Other | siderophore | unknown | ND |
| JJ1027 | *Gottfriedia acidiceleris* | no | No | PKS | T3PKS | unknown | ND |
| JJ1031 | *Gottfriedia acidiceleris* | no | No | Other | betalactone | unknown | ND |
| JJ1031 | *Gottfriedia acidiceleris* | no | No | Terpene | terpene | unknown | ND |
| JJ1031 | *Gottfriedia acidiceleris* | no | No | Terpene | terpene | unknown | ND |
| JJ1031 | *Gottfriedia acidiceleris* | no | No | Other | siderophore | unknown | ND |
| JJ1031 | *Gottfriedia acidiceleris* | no | No | RiPP | LAP | unknown | ND |
| JJ1041 | *Bacillus safensis* | strong | Strong | NRPS | NRPS | lichenysin | 85 |
| JJ1041 | *Bacillus safensis* | strong | Strong | PKS-NRPS | NRPS | zwittermicin A | 18 |
| JJ1041 | *Bacillus safensis* | strong | Strong | RiPP | RRE-containing | unknown | ND |
| JJ1041 | *Bacillus safensis* | strong | Strong | Terpene | terpene | carotenoid | 50 |
| JJ1041 | *Bacillus safensis* | strong | Strong | RiPP | RRE-containing | plantazolicin | 91 |
| JJ1041 | *Bacillus safensis* | strong | Strong | Other | betalactone | fengycin | 53 |
| JJ1041 | *Bacillus safensis* | strong | Strong | Terpene | terpene | unknown | ND |
| JJ1041 | *Bacillus safensis* | strong | Strong | PKS | T3PKS | unknown | ND |
| JJ1041 | *Bacillus safensis* | strong | Strong | Other | betalactone | bottromycin A | 6 |
| JJ1041 | *Bacillus safensis* | strong | Strong | RiPP | RiPP-like | unknown | ND |
| JJ1041 | *Bacillus safensis* | strong | Strong | Other | other | bacilysin | 85 |
| JJ1041 | *Bacillus safensis* | strong | Strong | NRPS | NRPS | bacillibactin | 53 |
| JJ1043 | *Bacillus velezensis* | strong | Strong | NRPS | NRPS | surfactin | 91 |
| JJ1043 | *Bacillus velezensis* | strong | Strong | PKS | PKS-like | butirosin A | 7 |
| JJ1043 | *Bacillus velezensis* | strong | Strong | Terpene | terpene | unknown | ND |
| JJ1043 | *Bacillus velezensis* | strong | Strong | PKS | transAT-PKS | macrolactin H | 100 |
| JJ1043 | *Bacillus velezensis* | strong | Strong | PKS-NRPS | transAT-PKS | bacillaene | 100 |
| JJ1043 | *Bacillus velezensis* | strong | Strong | NRPS | NRPS | fengycin | 100 |
| JJ1043 | *Bacillus velezensis* | strong | Strong | Terpene | terpene | unknown | ND |
| JJ1043 | *Bacillus velezensis* | strong | Strong | PKS | T3PKS | unknown | ND |
| JJ1043 | *Bacillus velezensis* | strong | Strong | PKS | transAT-PKS | difficidin | 100 |
| JJ1043 | *Bacillus velezensis* | strong | Strong | NRPS | NRPS | bacillibactin | 100 |
| JJ1043 | *Bacillus velezensis* | strong | Strong | NRPS | NRPS | unknown | ND |
| JJ1043 | *Bacillus velezensis* | strong | Strong | Other | other | bacilysin | 100 |
| JJ1043 | *Bacillus velezensis* | strong | Strong | NRPS | Others | iturin/Bacilomycin L | 100 |
| JJ1048 | *Bacillus (other)* | no | No | NRPS | NRPS | bacillibactin | 46 |
| JJ1048 | *Bacillus (other)* | no | No | Other | betalactone | fengycin | 40 |
| JJ1048 | *Bacillus (other)* | no | No | NRPS | NRPS | unknown | ND |
| JJ1048 | *Bacillus (other)* | no | No | Terpene | terpene | unknown | ND |
| JJ1048 | *Bacillus (other)* | no | No | RiPP | LAP | unknown | ND |
| JJ1048 | *Bacillus (other)* | no | No | RiPP | lassopeptide | unknown | ND |
| JJ1048 | *Bacillus (other)* | no | No | RiPP | bacteriocin | unknown | ND |
| JJ1048 | *Bacillus (other)* | no | No | RiPP | lassopeptide | paeninodin | 100 |
| JJ1048 | *Bacillus (other)* | no | No | Other | siderophore | petrobactin | 100 |
| JJ1051 | *Bacillus velezensis* | weak | Strong | PKS-NRPS | transAT-PKS-like | bacillaene | 35 |
| JJ1051 | *Bacillus velezensis* | weak | Strong | NRPS | NRPS | bacillibactin | 100 |
| JJ1051 | *Bacillus velezensis* | weak | Strong | Other | other | bacilysin | 100 |
| JJ1051 | *Bacillus velezensis* | weak | Strong | PKS | PKS-like | butirosin A | 7 |
| JJ1051 | *Bacillus velezensis* | weak | Strong | PKS | transAT-PKS-like | difficidin | 53 |
| JJ1051 | *Bacillus velezensis* | weak | Strong | NRPS | NRPS | fengycin | 80 |
| JJ1051 | *Bacillus velezensis* | weak | Strong | PKS | transAT-PKS | macrolactin H | 80 |
| JJ1051 | *Bacillus velezensis* | weak | Strong | Terpene | terpene | unknown | ND |
| JJ1051 | *Bacillus velezensis* | weak | Strong | Terpene | terpene | unknown | ND |
| JJ1051 | *Bacillus velezensis* | weak | Strong | PKS | T3PKS | unknown | ND |
| JJ1051 | *Bacillus velezensis* | weak | Strong | NRPS | NRPS | unknown | ND |
| JJ1051 | *Bacillus velezensis* | weak | Strong | NRPS | NRPS | surfactin | 39 |
| JJ1051 | *Bacillus velezensis* | weak | Strong | NRPS | Others | iturin/Bacilomycin L | 80 |
| JJ1054 | *Bacillus thuringiensis* | no | Sparse | NRPS | NRPS | bacillibactin | 46 |
| JJ1054 | *Bacillus thuringiensis* | no | Sparse | Other | betalactone | fengycin | 40 |
| JJ1054 | *Bacillus thuringiensis* | no | Sparse | Terpene | terpene | molybdenum cofactor | 17 |
| JJ1054 | *Bacillus thuringiensis* | no | Sparse | NRPS | NRPS | unknown | ND |
| JJ1054 | *Bacillus thuringiensis* | no | Sparse | NRPS | NRPS | unknown | ND |
| JJ1054 | *Bacillus thuringiensis* | no | Sparse | NRPS | NRPS-like | unknown | ND |
| JJ1054 | *Bacillus thuringiensis* | no | Sparse | RiPP | LAP | unknown | ND |
| JJ1054 | *Bacillus thuringiensis* | no | Sparse | RiPP | sactipeptide | unknown | ND |
| JJ1054 | *Bacillus thuringiensis* | no | Sparse | Other | siderophore | petrobactin | 100 |
| JJ1054 | *Bacillus thuringiensis* | no | Sparse | Other | ladderane | S-layerglycan | 26 |
| JJ1071 | *Bacillus velezensis* | strong | Strong | PKS-NRPS | transAT-PKS | bacillaene | 100 |
| JJ1071 | *Bacillus velezensis* | strong | Strong | NRPS | NRPS | bacillibactin | 100 |
| JJ1071 | *Bacillus velezensis* | strong | Strong | Other | other | bacilysin | 100 |
| JJ1071 | *Bacillus velezensis* | strong | Strong | PKS | PKS-like | butirosin A | 7 |
| JJ1071 | *Bacillus velezensis* | strong | Strong | PKS | transAT-PKS-like | difficidin | 100 |
| JJ1071 | *Bacillus velezensis* | strong | Strong | NRPS | NRPS | fengycin | 100 |
| JJ1071 | *Bacillus velezensis* | strong | Strong | PKS | transAT-PKS | macrolactin H | 100 |
| JJ1071 | *Bacillus velezensis* | strong | Strong | PKS | T3PKS | unknown | ND |
| JJ1071 | *Bacillus velezensis* | strong | Strong | RiPP | lanthipeptide | unknown | ND |
| JJ1071 | *Bacillus velezensis* | strong | Strong | Terpene | terpene | unknown | ND |
| JJ1071 | *Bacillus velezensis* | strong | Strong | Terpene | terpene | unknown | ND |
| JJ1071 | *Bacillus velezensis* | strong | Strong | NRPS | NRPS | surfactin | 82 |
| JJ1071 | *Bacillus velezensis* | strong | Strong | NRPS | Others | iturin/Bacilomycin L | 100 |
| JJ1072 | *Priestia megaterium* | no | No | Terpene | terpene | carotenoid | 50 |
| JJ1072 | *Priestia megaterium* | no | No | Terpene | terpene | locillomycin | 14 |
| JJ1072 | *Priestia megaterium* | no | No | Other | phosphonate | unknown | ND |
| JJ1072 | *Priestia megaterium* | no | No | PKS | T3PKS | unknown | ND |
| JJ1072 | *Priestia megaterium* | no | No | Terpene | terpene | unknown | ND |
| JJ1072 | *Priestia megaterium* | no | No | Other | siderophore | unknown | ND |
| JJ1076 | *Bacillus safensis* | weak | Strong | NRPS | NRPS | bacillibactin | 53 |
| JJ1076 | *Bacillus safensis* | weak | Strong | Other | other | bacilysin | 85 |
| JJ1076 | *Bacillus safensis* | weak | Strong | Terpene | terpene | carotenoid | 50 |
| JJ1076 | *Bacillus safensis* | weak | Strong | Other | betalactone | fengycin | 53 |
| JJ1076 | *Bacillus safensis* | weak | Strong | NRPS | NRPS | lichenysin | 85 |
| JJ1076 | *Bacillus safensis* | weak | Strong | Other | betalactone | unknown | ND |
| JJ1076 | *Bacillus safensis* | weak | Strong | Terpene | terpene | unknown | ND |
| JJ1076 | *Bacillus safensis* | weak | Strong | PKS | T3PKS | unknown | ND |
| JJ1076 | *Bacillus safensis* | weak | Strong | RiPP | LAP | plantazolicin | 25 |
| JJ1077 | *Cytobacillus firmus* | no | Sparse | PKS | T3PKS | unknown | ND |
| JJ1077 | *Cytobacillus firmus* | no | Sparse | RiPP | lanthipeptide | unknown | ND |
| JJ1077 | *Cytobacillus firmus* | no | Sparse | Terpene | terpene | unknown | ND |
| JJ1077 | *Cytobacillus firmus* | no | Sparse | Other | siderophore | petrobactin | 33 |
| JJ1079 | *Priestia megaterium* | no | No | Terpene | terpene | carotenoid | 50 |
| JJ1079 | *Priestia megaterium* | no | No | Terpene | terpene | unknown | ND |
| JJ1079 | *Priestia megaterium* | no | No | Other | siderophore | unknown | ND |
| JJ1079 | *Priestia megaterium* | no | No | Other | phosphonate | unknown | ND |
| JJ1079 | *Priestia megaterium* | no | No | Terpene | terpene | surfactin | 13 |
| JJ1083 | *Bacillus thuringiensis* | weak | Sparse | NRPS | NRPS | bacillibactin | 46 |
| JJ1083 | *Bacillus thuringiensis* | weak | Sparse | Other | betalactone | fengycin | 40 |
| JJ1083 | *Bacillus thuringiensis* | weak | Sparse | Other | furan | methylenomycin A | 9 |
| JJ1083 | *Bacillus thuringiensis* | weak | Sparse | Terpene | terpene | molybdenum cofactor | 17 |
| JJ1083 | *Bacillus thuringiensis* | weak | Sparse | NRPS | NRPS-like | unknown | ND |
| JJ1083 | *Bacillus thuringiensis* | weak | Sparse | NRPS | NRPS | unknown | ND |
| JJ1083 | *Bacillus thuringiensis* | weak | Sparse | NRPS | NRPS | unknown | ND |
| JJ1083 | *Bacillus thuringiensis* | weak | Sparse | RiPP | LAP | unknown | ND |
| JJ1083 | *Bacillus thuringiensis* | weak | Sparse | RiPP | bacteriocin | unknown | ND |
| JJ1083 | *Bacillus thuringiensis* | weak | Sparse | Other | siderophore | petrobactin | 100 |
| JJ1083 | *Bacillus thuringiensis* | weak | Sparse | Other | ladderane | S-layerglycan | 26 |
| JJ1083 | *Bacillus thuringiensis* | weak | Sparse | PKS-NRPS | T1PKS | zwittermicin A | 66 |
| JJ1088 | *Bacillus velezensis* | strong | Strong | PKS-NRPS | transAT-PKS | bacillaene | 100 |
| JJ1088 | *Bacillus velezensis* | strong | Strong | NRPS | bacteriocin | bacillibactin | 100 |
| JJ1088 | *Bacillus velezensis* | strong | Strong | Other | other | bacilysin | 100 |
| JJ1088 | *Bacillus velezensis* | strong | Strong | PKS | PKS-like | butirosin A | 7 |
| JJ1088 | *Bacillus velezensis* | strong | Strong | PKS | transAT-PKS-like | difficidin | 100 |
| JJ1088 | *Bacillus velezensis* | strong | Strong | NRPS | NRPS | fengycin | 80 |
| JJ1088 | *Bacillus velezensis* | strong | Strong | PKS | transAT-PKS | macrolactin H | 100 |
| JJ1088 | *Bacillus velezensis* | strong | Strong | PKS | T3PKS | unknown | ND |
| JJ1088 | *Bacillus velezensis* | strong | Strong | RiPP | lanthipeptide | unknown | ND |
| JJ1088 | *Bacillus velezensis* | strong | Strong | Terpene | terpene | unknown | ND |
| JJ1088 | *Bacillus velezensis* | strong | Strong | Terpene | terpene | unknown | ND |
| JJ1088 | *Bacillus velezensis* | strong | Strong | NRPS | NRPS | surfactin | 82 |
| JJ1088 | *Bacillus velezensis* | strong | Strong | NRPS | Others | iturin/Bacilomycin L | 80 |
| JJ1089 | *Cytobacillus firmus* | weak | Sparse | PKS | T3PKS | unknown | ND |
| JJ1089 | *Cytobacillus firmus* | weak | Sparse | RiPP | lanthipeptide | unknown | ND |
| JJ1089 | *Cytobacillus firmus* | weak | Sparse | Terpene | terpene | unknown | ND |
| JJ1089 | *Cytobacillus firmus* | weak | Sparse | Other | siderophore | petrobactin | 33 |
| JJ1098 | *Bacillus thuringiensis* | no | Sparse | NRPS | NRPS | bacillibactin | 46 |
| JJ1098 | *Bacillus thuringiensis* | no | Sparse | Other | betalactone | fengycin | 40 |
| JJ1098 | *Bacillus thuringiensis* | no | Sparse | Terpene | terpene | molybdenum cofactor | 17 |
| JJ1098 | *Bacillus thuringiensis* | no | Sparse | NRPS | NRPS | unknown | ND |
| JJ1098 | *Bacillus thuringiensis* | no | Sparse | RiPP | LAP | unknown | ND |
| JJ1098 | *Bacillus thuringiensis* | no | Sparse | NRPS | NRPS | unknown | ND |
| JJ1098 | *Bacillus thuringiensis* | no | Sparse | RiPP | bacteriocin | unknown | ND |
| JJ1098 | *Bacillus thuringiensis* | no | Sparse | Other | furan | unknown | ND |
| JJ1098 | *Bacillus thuringiensis* | no | Sparse | Other | siderophore | petrobactin | 100 |
| JJ1098 | *Bacillus thuringiensis* | no | Sparse | Other | ladderane | S-layerglycan | 20 |
| JJ1098 | *Bacillus thuringiensis* | no | Sparse | PKS-NRPS | NRPS | zwittermicin A | 59 |
| JJ1104 | *Cytobacillus firmus* | no | Sparse | PKS | T3PKS | unknown | ND |
| JJ1104 | *Cytobacillus firmus* | no | Sparse | Terpene | terpene | unknown | ND |
| JJ1104 | *Cytobacillus firmus* | no | Sparse | Other | siderophore | petrobactin | 33 |
| JJ1104 | *Cytobacillus firmus* | no | Sparse | RiPP | lanthipeptide | rhizocticin A | 6 |
| JJ1114 | *Bacillus (other)* | no | No | Terpene | terpene | carotenoid | 50 |
| JJ1114 | *Bacillus (other)* | no | No | Other | phosphonate | unknown | ND |
| JJ1114 | *Bacillus (other)* | no | No | PKS | T3PKS | unknown | ND |
| JJ1114 | *Bacillus (other)* | no | No | Terpene | terpene | unknown | ND |
| JJ1114 | *Bacillus (other)* | no | No | Other | siderophore | unknown | ND |
| JJ1114 | *Bacillus (other)* | no | No | RiPP | LAP | unknown | ND |
| JJ1114 | *Bacillus (other)* | no | No | Terpene | terpene | surfactin | 13 |
| JJ1116 | *Bacillus (other)* | no | No | Terpene | terpene | carotenoid | 50 |
| JJ1116 | *Bacillus (other)* | no | No | Other | phosphonate | unknown | ND |
| JJ1116 | *Bacillus (other)* | no | No | PKS | T3PKS | unknown | ND |
| JJ1116 | *Bacillus (other)* | no | No | RiPP | lanthipeptide | unknown | ND |
| JJ1116 | *Bacillus (other)* | no | No | Terpene | terpene | unknown | ND |
| JJ1116 | *Bacillus (other)* | no | No | Other | siderophore | unknown | ND |
| JJ1116 | *Bacillus (other)* | no | No | Terpene | terpene | surfactin | 13 |
| JJ1122 | *Bacillus (other)* | no | No | PKS | T3PKS | unknown | ND |
| JJ1122 | *Bacillus (other)* | no | No | RiPP | LAP | unknown | ND |
| JJ1122 | *Bacillus (other)* | no | No | RiPP | lassopeptide | paeninodin | 80 |
| JJ1127 | *Bacillus (other)* | no | No | Other | betalactone | fengycin | 40 |
| JJ1127 | *Bacillus (other)* | no | No | NRPS | NRPS-like | unknown | ND |
| JJ1127 | *Bacillus (other)* | no | No | RiPP | LAP | unknown | ND |
| JJ1127 | *Bacillus (other)* | no | No | Terpene | terpene | unknown | ND |
| JJ1127 | *Bacillus (other)* | no | No | Other | siderophore | unknown | ND |
| JJ1127 | *Bacillus (other)* | no | No | RiPP | bacteriocin | unknown | ND |
| JJ1138 | *Bacillus altitudinis* | strong | Sparse | NRPS | NRPS-like | locillomycin | 21 |
| JJ1138 | *Bacillus altitudinis* | strong | Sparse | NRPS | NRPS | lichenysin | 85 |
| JJ1138 | *Bacillus altitudinis* | strong | Sparse | RiPP | RRE-containing | unknown | ND |
| JJ1138 | *Bacillus altitudinis* | strong | Sparse | Terpene | terpene | carotenoid | 50 |
| JJ1138 | *Bacillus altitudinis* | strong | Sparse | Other | betalactone | fengycin | 53 |
| JJ1138 | *Bacillus altitudinis* | strong | Sparse | Terpene | terpene | unknown | ND |
| JJ1138 | *Bacillus altitudinis* | strong | Sparse | PKS | T3PKS | unknown | ND |
| JJ1138 | *Bacillus altitudinis* | strong | Sparse | RiPP | RiPP-like | unknown | ND |
| JJ1138 | *Bacillus altitudinis* | strong | Sparse | Other | betalactone | unknown | ND |
| JJ1138 | *Bacillus altitudinis* | strong | Sparse | NRPS | NRPS | bacillibactin | 53 |
| JJ1142 | *Priestia megaterium* | no | No | Terpene | terpene | carotenoid | 50 |
| JJ1142 | *Priestia megaterium* | no | No | Other | phosphonate | unknown | ND |
| JJ1142 | *Priestia megaterium* | no | No | PKS | T3PKS | unknown | ND |
| JJ1142 | *Priestia megaterium* | no | No | Other | siderophore | unknown | ND |
| JJ1142 | *Priestia megaterium* | no | No | Terpene | terpene | unknown | ND |
| JJ1142 | *Priestia megaterium* | no | No | Other | siderophore | unknown | ND |
| JJ1142 | *Priestia megaterium* | no | No | Terpene | terpene | surfactin | 8 |
| JJ1144 | *Bacillus safensis* | strong | Strong | NRPS | NRPS | bacillibactin | 53 |
| JJ1144 | *Bacillus safensis* | strong | Strong | Other | other | bacilysin | 85 |
| JJ1144 | *Bacillus safensis* | strong | Strong | Other | other | carotenoid | 50 |
| JJ1144 | *Bacillus safensis* | strong | Strong | Other | betalactone | fengycin | 53 |
| JJ1144 | *Bacillus safensis* | strong | Strong | NRPS | NRPS | lichenysin | 85 |
| JJ1144 | *Bacillus safensis* | strong | Strong | PKS | T3PKS | unknown | ND |
| JJ1144 | *Bacillus safensis* | strong | Strong | Other | betalactone | unknown | ND |
| JJ1144 | *Bacillus safensis* | strong | Strong | Terpene | terpene | unknown | ND |
| JJ1144 | *Bacillus safensis* | strong | Strong | RiPP | bacteriocin | unknown | ND |
| JJ1144 | *Bacillus safensis* | strong | Strong | RiPP | LAP | plantazolicin | 91 |
| JJ1144 | *Bacillus safensis* | strong | Strong | PKS-NRPS | NRPS | zwittermicin A | 18 |
| JJ1146 | *Neobacillus vireti* | no | No | RiPP | LAP | lankacidin C | 13 |
| JJ1146 | *Neobacillus vireti* | no | No | PKS | T3PKS | unknown | ND |
| JJ1146 | *Neobacillus vireti* | no | No | Terpene | terpene | unknown | ND |
| JJ1146 | *Neobacillus vireti* | no | No | RiPP | lassopeptide | paeninodin | 100 |
| JJ1149 | *Bacillus (other)* | no | No | RiPP | LAP | unknown | ND |
| JJ1149 | *Bacillus (other)* | no | No | NRPS | NRPS | bacillibactin | 46 |
| JJ1149 | *Bacillus (other)* | no | No | Other | betalactone | fengycin | 40 |
| JJ1149 | *Bacillus (other)* | no | No | RiPP | RiPP-like | unknown | ND |
| JJ1149 | *Bacillus (other)* | no | No | RiPP | RiPP-like | unknown | ND |
| JJ1149 | *Bacillus (other)* | no | No | Terpene | terpene | molybdenum cofactor | 17 |
| JJ1149 | *Bacillus (other)* | no | No | RiPP | lassopeptide | paeninodin | 100 |
| JJ1149 | *Bacillus (other)* | no | No | RiPP | lanthipeptide-class-ii | cerecidin | 76 |
| JJ1157 | *Priestia megaterium* | no | No | PKS | T3PKS | unknown | ND |
| JJ1157 | *Priestia megaterium* | no | No | Terpene | terpene | unknown | ND |
| JJ1157 | *Priestia megaterium* | no | No | Other | phosphonate | unknown | ND |
| JJ1157 | *Priestia megaterium* | no | No | Other | siderophore | unknown | ND |
| JJ1157 | *Priestia megaterium* | no | No | Terpene | terpene | unknown | ND |
| JJ1157 | *Priestia megaterium* | no | No | Terpene | terpene | surfactin | 13 |
| JJ1159 | *Bacillus (other)* | no | No | Other | betalactone | fengycin | 46 |
| JJ1159 | *Bacillus (other)* | no | No | NRPS | NRPS | koranimine | 87 |
| JJ1159 | *Bacillus (other)* | no | No | NRPS | NRPS | meilingmycin | 2 |
| JJ1159 | *Bacillus (other)* | no | No | RiPP | LAP | unknown | ND |
| JJ1159 | *Bacillus (other)* | no | No | Terpene | terpene | unknown | ND |
| JJ1159 | *Bacillus (other)* | no | No | PKS | T3PKS | unknown | ND |
| JJ1159 | *Bacillus (other)* | no | No | Other | siderophore | unknown | ND |
| JJ1159 | *Bacillus (other)* | no | No | Terpene | terpene | unknown | ND |
| JJ1163 | *Bacillus altitudinis* | no | Sparse | Other | other | bacilysin | 85 |
| JJ1163 | *Bacillus altitudinis* | no | Sparse | Other | other | carotenoid | 50 |
| JJ1163 | *Bacillus altitudinis* | no | Sparse | Other | betalactone | fengycin | 53 |
| JJ1163 | *Bacillus altitudinis* | no | Sparse | NRPS | NRPS | lichenysin | 71 |
| JJ1163 | *Bacillus altitudinis* | no | Sparse | PKS | T3PKS | unknown | ND |
| JJ1163 | *Bacillus altitudinis* | no | Sparse | Terpene | terpene | unknown | ND |
| JJ1163 | *Bacillus altitudinis* | no | Sparse | RiPP | bacteriocin | unknown | ND |
| JJ1174 | *Bacillus altitudinis* | no | Sparse | Other | other | bacilysin | 85 |
| JJ1174 | *Bacillus altitudinis* | no | Sparse | Other | other | carotenoid | 50 |
| JJ1174 | *Bacillus altitudinis* | no | Sparse | Other | betalactone | fengycin | 53 |
| JJ1174 | *Bacillus altitudinis* | no | Sparse | NRPS | NRPS | lichenysin | 50 |
| JJ1174 | *Bacillus altitudinis* | no | Sparse | PKS | T3PKS | unknown | ND |
| JJ1174 | *Bacillus altitudinis* | no | Sparse | Terpene | terpene | unknown | ND |
| JJ1174 | *Bacillus altitudinis* | no | Sparse | RiPP | bacteriocin | unknown | ND |
| JJ1174 | *Bacillus altitudinis* | no | Sparse | RiPP | bacteriocin | unknown | ND |
| JJ1183 | *Bacillus altitudinis* | no | Sparse | Other | other | bacilysin | 71 |
| JJ1183 | *Bacillus altitudinis* | no | Sparse | Terpene | terpene | carotenoid | 50 |
| JJ1183 | *Bacillus altitudinis* | no | Sparse | Other | betalactone | fengycin | 53 |
| JJ1183 | *Bacillus altitudinis* | no | Sparse | NRPS | NRPS | lichenysin | 28 |
| JJ1183 | *Bacillus altitudinis* | no | Sparse | Terpene | terpene | unknown | ND |
| JJ1183 | *Bacillus altitudinis* | no | Sparse | Other | siderophore | unknown | ND |
| JJ1197 | *Bacillus thuringiensis* | no | Sparse | Other | betalactone | fengycin | 40 |
| JJ1197 | *Bacillus thuringiensis* | no | Sparse | RiPP | LAP | unknown | ND |
| JJ1197 | *Bacillus thuringiensis* | no | Sparse | NRPS | NRPS | unknown | ND |
| JJ1197 | *Bacillus thuringiensis* | no | Sparse | Terpene | terpene | unknown | ND |
| JJ1197 | *Bacillus thuringiensis* | no | Sparse | Other | siderophore | unknown | ND |
| JJ1197 | *Bacillus thuringiensis* | no | Sparse | RiPP | lassopeptide | paeninodin | 100 |
| JJ1201 | *Bacillus (other)* | no | No | Terpene | terpene | carotenoid | 50 |
| JJ1201 | *Bacillus (other)* | no | No | PKS | T3PKS | unknown | ND |
| JJ1201 | *Bacillus (other)* | no | No | Other | phosphonate | unknown | ND |
| JJ1201 | *Bacillus (other)* | no | No | Other | siderophore | unknown | ND |
| JJ1201 | *Bacillus (other)* | no | No | Terpene | terpene | unknown | ND |
| JJ1201 | *Bacillus (other)* | no | No | Terpene | terpene | surfactin | 13 |
| JJ1202 | *Priestia megaterium* | no | No | Terpene | terpene | carotenoid | 50 |
| JJ1202 | *Priestia megaterium* | no | No | PKS | T3PKS | unknown | ND |
| JJ1202 | *Priestia megaterium* | no | No | Other | phosphonate | unknown | ND |
| JJ1202 | *Priestia megaterium* | no | No | Terpene | terpene | unknown | ND |
| JJ1202 | *Priestia megaterium* | no | No | Other | siderophore | unknown | ND |
| JJ1202 | *Priestia megaterium* | no | No | RiPP | bacteriocin | unknown | ND |
| JJ1202 | *Priestia megaterium* | no | No | Terpene | terpene | surfactin | 13 |
| JJ1204 | *Priestia megaterium* | no | No | Terpene | terpene | carotenoid | 50 |
| JJ1204 | *Priestia megaterium* | no | No | PKS | T3PKS | unknown | ND |
| JJ1204 | *Priestia megaterium* | no | No | Other | phosphonate | unknown | ND |
| JJ1204 | *Priestia megaterium* | no | No | Terpene | terpene | unknown | ND |
| JJ1204 | *Priestia megaterium* | no | No | Other | siderophore | unknown | ND |
| JJ1204 | *Priestia megaterium* | no | No | Terpene | terpene | surfactin | 13 |
| JJ1209 | *Bacillus pumilus* | strong | Strong | NRPS | NRPS | bacillibactin | 53 |
| JJ1209 | *Bacillus pumilus* | strong | Strong | Other | other | bacilysin | 85 |
| JJ1209 | *Bacillus pumilus* | strong | Strong | Terpene | terpene | carotenoid | 50 |
| JJ1209 | *Bacillus pumilus* | strong | Strong | Other | betalactone | fengycin | 46 |
| JJ1209 | *Bacillus pumilus* | strong | Strong | NRPS | NRPS | lichenysin | 50 |
| JJ1209 | *Bacillus pumilus* | strong | Strong | PKS | T3PKS | unknown | ND |
| JJ1209 | *Bacillus pumilus* | strong | Strong | Terpene | terpene | unknown | ND |
| JJ1209 | *Bacillus pumilus* | strong | Strong | RiPP | head_to_tail | sporulation killing factor | 85 |
| JJ1209 | *Bacillus pumilus* | strong | Strong | PKS-NRPS | T1PKS | zwittermicin A | 18 |
| JJ1211 | *Priestia megaterium* | no | No | Terpene | terpene | carotenoid | 50 |
| JJ1211 | *Priestia megaterium* | no | No | Terpene | terpene | unknown | ND |
| JJ1211 | *Priestia megaterium* | no | No | Other | siderophore | unknown | ND |
| JJ1211 | *Priestia megaterium* | no | No | PKS | T3PKS | unknown | ND |
| JJ1211 | *Priestia megaterium* | no | No | RiPP | lanthipeptide | unknown | ND |
| JJ1211 | *Priestia megaterium* | no | No | Terpene | terpene | unknown | ND |
| JJ1215 | *Priestia megaterium* | no | No | Terpene | terpene | carotenoid | 50 |
| JJ1215 | *Priestia megaterium* | no | No | Terpene | terpene | unknown | ND |
| JJ1215 | *Priestia megaterium* | no | No | PKS | T3PKS | unknown | ND |
| JJ1215 | *Priestia megaterium* | no | No | Other | siderophore | unknown | ND |
| JJ1215 | *Priestia megaterium* | no | No | Terpene | terpene | unknown | ND |
| JJ1216 | *Bacillus thuringiensis* | no | Sparse | NRPS | NRPS-like | unknown | ND |
| JJ1216 | *Bacillus thuringiensis* | no | Sparse | RiPP | LAP | unknown | ND |
| JJ1216 | *Bacillus thuringiensis* | no | Sparse | Other | siderophore | petrobactin | 100 |
| JJ1216 | *Bacillus thuringiensis* | no | Sparse | NRPS | NRPS | bacillibactin | 46 |
| JJ1216 | *Bacillus thuringiensis* | no | Sparse | NRPS | NRPS | unknown | ND |
| JJ1216 | *Bacillus thuringiensis* | no | Sparse | Other | betalactone | fengycin | 40 |
| JJ1216 | *Bacillus thuringiensis* | no | Sparse | RiPP | RiPP-like | unknown | ND |
| JJ1216 | *Bacillus thuringiensis* | no | Sparse | RiPP | RiPP-like | unknown | ND |
| JJ1216 | *Bacillus thuringiensis* | no | Sparse | NRPS | NRPS | unknown | ND |
| JJ1216 | *Bacillus thuringiensis* | no | Sparse | Terpene | terpene | molybdenum cofactor | 17 |
| JJ1216 | *Bacillus thuringiensis* | no | Sparse | NRPS | NRPS | unknown | ND |
| JJ1216 | *Bacillus thuringiensis* | no | Sparse | RiPP | ranthipeptide | unknown | ND |
| JJ1216 | *Bacillus thuringiensis* | no | Sparse | PKS-NRPS | ladderane | zwittermicin A | 81 |
| JJ1216 | *Bacillus thuringiensis* | no | Sparse | RiPP | RiPP-like | unknown | ND |
| JJ1217 | *Priestia megaterium* | no | No | Terpene | terpene | carotenoid | 50 |
| JJ1217 | *Priestia megaterium* | no | No | Terpene | terpene | locillomycin | 14 |
| JJ1217 | *Priestia megaterium* | no | No | Other | phosphonate | unknown | ND |
| JJ1217 | *Priestia megaterium* | no | No | PKS | T3PKS | unknown | ND |
| JJ1217 | *Priestia megaterium* | no | No | Terpene | terpene | unknown | ND |
| JJ1217 | *Priestia megaterium* | no | No | Other | siderophore | unknown | ND |
| JJ1217 | *Priestia megaterium* | no | No | RiPP | bacteriocin | unknown | ND |
| JJ1224 | *Bacillus (other)* | no | No | Terpene | terpene | carotenoid | 50 |
| JJ1224 | *Bacillus (other)* | no | No | PKS | T3PKS | unknown | ND |
| JJ1224 | *Bacillus (other)* | no | No | Terpene | terpene | unknown | ND |
| JJ1224 | *Bacillus (other)* | no | No | Other | siderophore | unknown | ND |
| JJ1224 | *Bacillus (other)* | no | No | Other | phosphonate | unknown | ND |
| JJ1224 | *Bacillus (other)* | no | No | Terpene | terpene | surfactin | 8 |
| JJ1231 | *Priestia megaterium* | no | No | Terpene | terpene | carotenoid | 50 |
| JJ1231 | *Priestia megaterium* | no | No | Other | phosphonate | unknown | ND |
| JJ1231 | *Priestia megaterium* | no | No | PKS | T3PKS | unknown | ND |
| JJ1231 | *Priestia megaterium* | no | No | Terpene | terpene | unknown | ND |
| JJ1231 | *Priestia megaterium* | no | No | Other | siderophore | unknown | ND |
| JJ1231 | *Priestia megaterium* | no | No | RiPP | lanthipeptide | unknown | ND |
| JJ1231 | *Priestia megaterium* | no | No | Terpene | terpene | surfactin | 13 |
| JJ1232 | *Neobacillus drentensis* | no | No | PKS | T3PKS | unknown | ND |
| JJ1232 | *Neobacillus drentensis* | no | No | RiPP | LAP | unknown | ND |
| JJ1232 | *Neobacillus drentensis* | no | No | Terpene | terpene | unknown | ND |
| JJ1232 | *Neobacillus drentensis* | no | No | RiPP | lassopeptide | paeninodin | 100 |
| JJ1235 | *Priestia megaterium* | no | No | Terpene | terpene | unknown | ND |
| JJ1235 | *Priestia megaterium* | no | No | Other | siderophore | unknown | ND |
| JJ1235 | *Priestia megaterium* | no | No | Terpene | terpene | unknown | ND |
| JJ1235 | *Priestia megaterium* | no | No | PKS | T3PKS | unknown | ND |
| JJ1235 | *Priestia megaterium* | no | No | Other | phosphonate | unknown | ND |
| JJ1237 | *Bacillus thuringiensis* | weak | Sparse | NRPS | NRPS | bacillibactin | 46 |
| JJ1237 | *Bacillus thuringiensis* | weak | Sparse | Other | betalactone | fengycin | 40 |
| JJ1237 | *Bacillus thuringiensis* | weak | Sparse | Terpene | terpene | molybdenum cofactor | 17 |
| JJ1237 | *Bacillus thuringiensis* | weak | Sparse | NRPS | NRPS | unknown | ND |
| JJ1237 | *Bacillus thuringiensis* | weak | Sparse | NRPS | NRPS-like | unknown | ND |
| JJ1237 | *Bacillus thuringiensis* | weak | Sparse | RiPP | LAP | unknown | ND |
| JJ1237 | *Bacillus thuringiensis* | weak | Sparse | NRPS | NRPS | unknown | ND |
| JJ1237 | *Bacillus thuringiensis* | weak | Sparse | RiPP | bacteriocin | unknown | ND |
| JJ1237 | *Bacillus thuringiensis* | weak | Sparse | Other | siderophore | petrobactin | 100 |
| JJ1237 | *Bacillus thuringiensis* | weak | Sparse | Other | ladderane | S-layerglycan | 20 |
| JJ1237 | *Bacillus thuringiensis* | weak | Sparse | PKS-NRPS | T1PKS | zwittermicin A | 51 |
| JJ1241 | *Bacillus altitudinis* | no | Sparse | Other | other | bacilysin | 85 |
| JJ1241 | *Bacillus altitudinis* | no | Sparse | Other | other | carotenoid | 50 |
| JJ1241 | *Bacillus altitudinis* | no | Sparse | Other | betalactone | fengycin | 53 |
| JJ1241 | *Bacillus altitudinis* | no | Sparse | NRPS | NRPS | lichenysin | 85 |
| JJ1241 | *Bacillus altitudinis* | no | Sparse | Other | betalactone | unknown | ND |
| JJ1241 | *Bacillus altitudinis* | no | Sparse | Terpene | terpene | unknown | ND |
| JJ1241 | *Bacillus altitudinis* | no | Sparse | PKS | T3PKS | unknown | ND |
| JJ1241 | *Bacillus altitudinis* | no | Sparse | RiPP | bacteriocin | unknown | ND |
| JJ1244 | *Bacillus safensis* | strong | Strong | NRPS | NRPS | lichenysin | 92 |
| JJ1244 | *Bacillus safensis* | strong | Strong | RiPP | RRE-containing | unknown | ND |
| JJ1244 | *Bacillus safensis* | strong | Strong | Terpene | terpene | carotenoid | 50 |
| JJ1244 | *Bacillus safensis* | strong | Strong | RiPP | RRE-containing | plantazolicin | 91 |
| JJ1244 | *Bacillus safensis* | strong | Strong | Other | betalactone | fengycin | 53 |
| JJ1244 | *Bacillus safensis* | strong | Strong | Terpene | terpene | unknown | ND |
| JJ1244 | *Bacillus safensis* | strong | Strong | PKS | T3PKS | unknown | ND |
| JJ1244 | *Bacillus safensis* | strong | Strong | RiPP | RiPP-like | unknown | ND |
| JJ1244 | *Bacillus safensis* | strong | Strong | Other | betalactone | bottromycin A | 6 |
| JJ1244 | *Bacillus safensis* | strong | Strong | Other | other | bacilysin | 85 |
| JJ1244 | *Bacillus safensis* | strong | Strong | NRPS | NRPS | bacillibactin | 53 |
| JJ1248 | *Bacillus subtilis* | no | Strong | PKS | transAT-PKS-like | aurantinin B | 17 |
| JJ1248 | *Bacillus subtilis* | no | Strong | NRPS | NRPS | bacillibactin | 100 |
| JJ1248 | *Bacillus subtilis* | no | Strong | Other | other | bacilysin | 100 |
| JJ1248 | *Bacillus subtilis* | no | Strong | NRPS | NRPS | fengycin | 73 |
| JJ1248 | *Bacillus subtilis* | no | Strong | PKS | T3PKS | unknown | ND |
| JJ1248 | *Bacillus subtilis* | no | Strong | Terpene | terpene | unknown | ND |
| JJ1248 | *Bacillus subtilis* | no | Strong | Terpene | terpene | unknown | ND |
| JJ1248 | *Bacillus subtilis* | no | Strong | RiPP | sactipeptide | sporulation killing factor | 100 |
| JJ1248 | *Bacillus subtilis* | no | Strong | RiPP | sactipeptide | subtilosin A | 100 |
| JJ1248 | *Bacillus subtilis* | no | Strong | NRPS | NRPS | surfactin | 82 |
| JJ1250 | *Priestia megaterium* | no | No | Terpene | terpene | carotenoid | 50 |
| JJ1250 | *Priestia megaterium* | no | No | Other | phosphonate | unknown | ND |
| JJ1250 | *Priestia megaterium* | no | No | Terpene | terpene | unknown | ND |
| JJ1250 | *Priestia megaterium* | no | No | PKS | T3PKS | unknown | ND |
| JJ1250 | *Priestia megaterium* | no | No | Other | siderophore | unknown | ND |
| JJ1250 | *Priestia megaterium* | no | No | Terpene | terpene | unknown | ND |
| JJ1253 | *Bacillus thuringiensis* | no | Sparse | NRPS | NRPS | bacillibactin | 46 |
| JJ1253 | *Bacillus thuringiensis* | no | Sparse | Other | betalactone | fengycin | 40 |
| JJ1253 | *Bacillus thuringiensis* | no | Sparse | Terpene | terpene | molybdenum cofactor | 17 |
| JJ1253 | *Bacillus thuringiensis* | no | Sparse | NRPS | NRPS | unknown | ND |
| JJ1253 | *Bacillus thuringiensis* | no | Sparse | NRPS | NRPS | unknown | ND |
| JJ1253 | *Bacillus thuringiensis* | no | Sparse | RiPP | LAP | unknown | ND |
| JJ1253 | *Bacillus thuringiensis* | no | Sparse | RiPP | bacteriocin | unknown | ND |
| JJ1253 | *Bacillus thuringiensis* | no | Sparse | Other | siderophore | petrobactin | 100 |
| JJ1255 | *Bacillus altitudinis* | strong | Sparse | NRPS | NRPS | bacillibactin | 53 |
| JJ1255 | *Bacillus altitudinis* | strong | Sparse | Other | other | bacilysin | 85 |
| JJ1255 | *Bacillus altitudinis* | strong | Sparse | Terpene | terpene | carotenoid | 50 |
| JJ1255 | *Bacillus altitudinis* | strong | Sparse | Other | betalactone | fengycin | 53 |
| JJ1255 | *Bacillus altitudinis* | strong | Sparse | NRPS | NRPS | lichenysin | 42 |
| JJ1255 | *Bacillus altitudinis* | strong | Sparse | NRPS | NRPS-like | locillomycin | 21 |
| JJ1255 | *Bacillus altitudinis* | strong | Sparse | Other | betalactone | unknown | ND |
| JJ1255 | *Bacillus altitudinis* | strong | Sparse | PKS | T3PKS | unknown | ND |
| JJ1255 | *Bacillus altitudinis* | strong | Sparse | Terpene | terpene | unknown | ND |
| JJ1255 | *Bacillus altitudinis* | strong | Sparse | Other | siderophore | unknown | ND |
| JJ1255 | *Bacillus altitudinis* | strong | Sparse | RiPP | bacteriocin | unknown | ND |
| JJ1260 | *Priestia megaterium* | no | No | Terpene | terpene | carotenoid | 50 |
| JJ1260 | *Priestia megaterium* | no | No | PKS | T3PKS | unknown | ND |
| JJ1260 | *Priestia megaterium* | no | No | Terpene | terpene | unknown | ND |
| JJ1260 | *Priestia megaterium* | no | No | Other | siderophore | unknown | ND |
| JJ1260 | *Priestia megaterium* | no | No | Other | phosphonate | unknown | ND |
| JJ1260 | *Priestia megaterium* | no | No | Terpene | terpene | surfactin | 13 |
| JJ1262 | *Neobacillus drentensis* | no | No | Terpene | terpene | carotenoid | 33 |
| JJ1262 | *Neobacillus drentensis* | no | No | PKS | T3PKS | unknown | ND |
| JJ1262 | *Neobacillus drentensis* | no | No | Terpene | terpene | unknown | ND |
| JJ1262 | *Neobacillus drentensis* | no | No | RiPP | LAP | unknown | ND |
| JJ1262 | *Neobacillus drentensis* | no | No | RiPP | lassopeptide | paeninodin | 100 |
| JJ1265 | *Bacillus thuringiensis* | no | Sparse | NRPS | NRPS | bacillibactin | 46 |
| JJ1265 | *Bacillus thuringiensis* | no | Sparse | Other | betalactone | fengycin | 40 |
| JJ1265 | *Bacillus thuringiensis* | no | Sparse | Terpene | terpene | molybdenum cofactor | 17 |
| JJ1265 | *Bacillus thuringiensis* | no | Sparse | NRPS | NRPS | unknown | ND |
| JJ1265 | *Bacillus thuringiensis* | no | Sparse | RiPP | LAP | unknown | ND |
| JJ1265 | *Bacillus thuringiensis* | no | Sparse | NRPS | NRPS | unknown | ND |
| JJ1265 | *Bacillus thuringiensis* | no | Sparse | RiPP | bacteriocin | unknown | ND |
| JJ1265 | *Bacillus thuringiensis* | no | Sparse | Other | siderophore | petrobactin | 100 |
| JJ1265 | *Bacillus thuringiensis* | no | Sparse | Other | ladderane | S-layerglycan | 26 |
| JJ1267 | *Priestia megaterium* | no | No | Terpene | terpene | carotenoid | 50 |
| JJ1267 | *Priestia megaterium* | no | No | PKS | T3PKS | unknown | ND |
| JJ1267 | *Priestia megaterium* | no | No | Terpene | terpene | unknown | ND |
| JJ1267 | *Priestia megaterium* | no | No | Other | siderophore | unknown | ND |
| JJ1267 | *Priestia megaterium* | no | No | RiPP | lassopeptide | paeninodin | 80 |
| JJ1267 | *Priestia megaterium* | no | No | Terpene | terpene | surfactin | 13 |
| JJ1272 | *Neobacillus drentensis* | no | No | Other | betalactone | fengycin | 40 |
| JJ1272 | *Neobacillus drentensis* | no | No | RiPP | LAP | unknown | ND |
| JJ1272 | *Neobacillus drentensis* | no | No | Terpene | terpene | unknown | ND |
| JJ1272 | *Neobacillus drentensis* | no | No | PKS | T3PKS | unknown | ND |
| JJ1272 | *Neobacillus drentensis* | no | No | RiPP | bacteriocin | unknown | ND |
| JJ1273 | *Neobacillus niacini* | no | No | Terpene | terpene | unknown | ND |
| JJ1273 | *Neobacillus niacini* | no | No | PKS | T3PKS | unknown | ND |
| JJ1273 | *Neobacillus niacini* | no | No | RiPP | bacteriocin | unknown | ND |
| JJ1273 | *Neobacillus niacini* | no | No | Terpene | terpene | unknown | ND |
| JJ1273 | *Neobacillus niacini* | no | No | RiPP | lassopeptide | paeninodin | 60 |
| JJ1276 | *Neobacillus drentensis* | no | No | Other | phosphonate | unknown | ND |
| JJ1276 | *Neobacillus drentensis* | no | No | Other | betalactone | unknown | ND |
| JJ1276 | *Neobacillus drentensis* | no | No | RiPP | LAP | unknown | ND |
| JJ1276 | *Neobacillus drentensis* | no | No | PKS | T3PKS | unknown | ND |
| JJ1276 | *Neobacillus drentensis* | no | No | Terpene | terpene | unknown | ND |
| JJ1284 | *Bacillus velezensis* | strong | Strong | NRPS | NRPS | surfactin | 91 |
| JJ1284 | *Bacillus velezensis* | strong | Strong | RiPP | RRE-containing | plantazolicin | 91 |
| JJ1284 | *Bacillus velezensis* | strong | Strong | PKS | PKS-like | butirosin A | 7 |
| JJ1284 | *Bacillus velezensis* | strong | Strong | Terpene | terpene | unknown | ND |
| JJ1284 | *Bacillus velezensis* | strong | Strong | PKS | transAT-PKS | macrolactin H | 100 |
| JJ1284 | *Bacillus velezensis* | strong | Strong | PKS-NRPS | transAT-PKS | bacillaene | 100 |
| JJ1284 | *Bacillus velezensis* | strong | Strong | NRPS | NRPS | fengycin | 100 |
| JJ1284 | *Bacillus velezensis* | strong | Strong | Terpene | terpene | unknown | ND |
| JJ1284 | *Bacillus velezensis* | strong | Strong | PKS | T3PKS | unknown | ND |
| JJ1284 | *Bacillus velezensis* | strong | Strong | PKS | transAT-PKS | difficidin | 100 |
| JJ1284 | *Bacillus velezensis* | strong | Strong | NRPS | NRPS | bacillibactin | 100 |
| JJ1284 | *Bacillus velezensis* | strong | Strong | Other | other | bacilysin | 100 |
| JJ1284 | *Bacillus velezensis* | strong | Strong | RiPP | lanthipeptide-class-ii | mersacidin | 100 |
| JJ1284 | *Bacillus velezensis* | strong | Strong | NRPS | Others | iturin/Bacilomycin L | 100 |
| JJ1295 | *Bacillus velezensis* | strong | Strong | PKS-NRPS | transAT-PKS-like | bacillaene | 100 |
| JJ1295 | *Bacillus velezensis* | strong | Strong | NRPS | NRPS | bacillibactin | 100 |
| JJ1295 | *Bacillus velezensis* | strong | Strong | Other | other | bacilysin | 100 |
| JJ1295 | *Bacillus velezensis* | strong | Strong | PKS | PKS-like | butirosin A | 7 |
| JJ1295 | *Bacillus velezensis* | strong | Strong | PKS | transAT-PKS-like | difficidin | 100 |
| JJ1295 | *Bacillus velezensis* | strong | Strong | Other | betalactone | fengycin | 86 |
| JJ1295 | *Bacillus velezensis* | strong | Strong | PKS | transAT-PKS | macrolactin H | 100 |
| JJ1295 | *Bacillus velezensis* | strong | Strong | PKS | T3PKS | unknown | ND |
| JJ1295 | *Bacillus velezensis* | strong | Strong | RiPP | lanthipeptide | unknown | ND |
| JJ1295 | *Bacillus velezensis* | strong | Strong | Terpene | terpene | unknown | ND |
| JJ1295 | *Bacillus velezensis* | strong | Strong | Terpene | terpene | unknown | ND |
| JJ1295 | *Bacillus velezensis* | strong | Strong | NRPS | NRPS | surfactin | 39 |
| JJ1345 | *Bacillus pumilus* | strong | Strong | NRPS | NRPS | bacillibactin | 53 |
| JJ1345 | *Bacillus pumilus* | strong | Strong | Other | other | bacilysin | 85 |
| JJ1345 | *Bacillus pumilus* | strong | Strong | Other | other | carotenoid | 50 |
| JJ1345 | *Bacillus pumilus* | strong | Strong | Other | betalactone | fengycin | 53 |
| JJ1345 | *Bacillus pumilus* | strong | Strong | NRPS | NRPS | lichenysin | 50 |
| JJ1345 | *Bacillus pumilus* | strong | Strong | PKS | T3PKS | unknown | ND |
| JJ1345 | *Bacillus pumilus* | strong | Strong | Other | betalactone | unknown | ND |
| JJ1345 | *Bacillus pumilus* | strong | Strong | Terpene | terpene | unknown | ND |
| JJ1345 | *Bacillus pumilus* | strong | Strong | NRPS | NRPS | xenocoumacin | 14 |
| JJ1345 | *Bacillus pumilus* | strong | Strong | PKS-NRPS | NRPS | zwittermicin A | 14 |
| JJ1368 | *Bacillus pumilus* | strong | Strong | RiPP | RiPP-like | unknown | ND |
| JJ1368 | *Bacillus pumilus* | strong | Strong | Other | other | bacilysin | 85 |
| JJ1368 | *Bacillus pumilus* | strong | Strong | NRPS | NRPS | bacillibactin | 53 |
| JJ1368 | *Bacillus pumilus* | strong | Strong | NRPS | NRPS | lichenysin | 85 |
| JJ1368 | *Bacillus pumilus* | strong | Strong | PKS-NRPS | NRPS | zwittermicin A | 18 |
| JJ1368 | *Bacillus pumilus* | strong | Strong | RiPP | RRE-containing | unknown | ND |
| JJ1368 | *Bacillus pumilus* | strong | Strong | Terpene | terpene | carotenoid | 50 |
| JJ1368 | *Bacillus pumilus* | strong | Strong | Other | betalactone | fengycin | 53 |
| JJ1368 | *Bacillus pumilus* | strong | Strong | Terpene | terpene | unknown | ND |
| JJ1368 | *Bacillus pumilus* | strong | Strong | PKS | T3PKS | unknown | ND |
| JJ1368 | *Bacillus pumilus* | strong | Strong | Other | betalactone | unknown | ND |
| JJ1389 | *Priestia megaterium* | no | No | Terpene | terpene | carotenoid | 50 |
| JJ1389 | *Priestia megaterium* | no | No | PKS | T3PKS | unknown | ND |
| JJ1389 | *Priestia megaterium* | no | No | Terpene | terpene | unknown | ND |
| JJ1389 | *Priestia megaterium* | no | No | Other | siderophore | unknown | ND |
| JJ1389 | *Priestia megaterium* | no | No | Other | phosphonate | unknown | ND |
| JJ1389 | *Priestia megaterium* | no | No | RiPP | bacteriocin | unknown | ND |
| JJ1389 | *Priestia megaterium* | no | No | Terpene | terpene | surfactin | 13 |
| JJ1392 | *Bacillus (other)* | no | No | Terpene | terpene | carotenoid | 50 |
| JJ1392 | *Bacillus (other)* | no | No | Other | siderophore | unknown | ND |
| JJ1392 | *Bacillus (other)* | no | No | Terpene | terpene | unknown | ND |
| JJ1392 | *Bacillus (other)* | no | No | PKS | T3PKS | unknown | ND |
| JJ1392 | *Bacillus (other)* | no | No | Other | phosphonate | unknown | ND |
| JJ1392 | *Bacillus (other)* | no | No | Terpene | terpene | surfactin | 8 |
| JJ1394 | *Bacillus (other)* | weak | No | Other | betalactone | fengycin | 46 |
| JJ1394 | *Bacillus (other)* | weak | No | NRPS | NRPS | koranimine | 25 |
| JJ1394 | *Bacillus (other)* | weak | No | RiPP | LAP | unknown | ND |
| JJ1394 | *Bacillus (other)* | weak | No | Other | siderophore | unknown | ND |
| JJ1394 | *Bacillus (other)* | weak | No | Terpene | terpene | unknown | ND |
| JJ1394 | *Bacillus (other)* | weak | No | Terpene | terpene | unknown | ND |
| JJ1394 | *Bacillus (other)* | weak | No | Other | phosphonate | unknown | ND |
| JJ1399 | *Priestia megaterium* | no | No | Terpene | terpene | carotenoid | 50 |
| JJ1399 | *Priestia megaterium* | no | No | PKS | T3PKS | unknown | ND |
| JJ1399 | *Priestia megaterium* | no | No | Other | phosphonate | unknown | ND |
| JJ1399 | *Priestia megaterium* | no | No | Terpene | terpene | unknown | ND |
| JJ1399 | *Priestia megaterium* | no | No | Other | siderophore | unknown | ND |
| JJ1399 | *Priestia megaterium* | no | No | Terpene | terpene | surfactin | 13 |
| JJ1403 | *Bacillus subtilis* | weak | Strong | PKS-NRPS | transAT-PKS | bacillaene | 100 |
| JJ1403 | *Bacillus subtilis* | weak | Strong | NRPS | NRPS | bacillibactin | 100 |
| JJ1403 | *Bacillus subtilis* | weak | Strong | Other | other | bacilysin | 100 |
| JJ1403 | *Bacillus subtilis* | weak | Strong | NRPS | NRPS | fengycin | 86 |
| JJ1403 | *Bacillus subtilis* | weak | Strong | PKS | T3PKS | unknown | ND |
| JJ1403 | *Bacillus subtilis* | weak | Strong | Terpene | terpene | unknown | ND |
| JJ1403 | *Bacillus subtilis* | weak | Strong | Terpene | terpene | unknown | ND |
| JJ1403 | *Bacillus subtilis* | weak | Strong | RiPP | lanthipeptide | subtilin | 100 |
| JJ1403 | *Bacillus subtilis* | weak | Strong | RiPP | head_to_tail | subtilosin A | 100 |
| JJ1403 | *Bacillus subtilis* | weak | Strong | NRPS | NRPS | surfactin | 82 |
| JJ1406 | *Priestia megaterium* | no | No | Terpene | terpene | carotenoid | 50 |
| JJ1406 | *Priestia megaterium* | no | No | PKS | T3PKS | unknown | ND |
| JJ1406 | *Priestia megaterium* | no | No | Terpene | terpene | unknown | ND |
| JJ1406 | *Priestia megaterium* | no | No | Other | siderophore | unknown | ND |
| JJ1406 | *Priestia megaterium* | no | No | Other | phosphonate | unknown | ND |
| JJ1406 | *Priestia megaterium* | no | No | Terpene | terpene | surfactin | 13 |
| JJ1413 | *Priestia megaterium* | no | No | Terpene | terpene | carotenoid | 50 |
| JJ1413 | *Priestia megaterium* | no | No | PKS | T3PKS | unknown | ND |
| JJ1413 | *Priestia megaterium* | no | No | Other | phosphonate | unknown | ND |
| JJ1413 | *Priestia megaterium* | no | No | Terpene | terpene | unknown | ND |
| JJ1413 | *Priestia megaterium* | no | No | Other | siderophore | unknown | ND |
| JJ1413 | *Priestia megaterium* | no | No | RiPP | lanthipeptide | unknown | ND |
| JJ1413 | *Priestia megaterium* | no | No | Terpene | terpene | surfactin | 13 |
| JJ1417 | *Neobacillus drentensis* | no | No | Other | betalactone | fengycin | 40 |
| JJ1417 | *Neobacillus drentensis* | no | No | PKS | T3PKS | unknown | ND |
| JJ1417 | *Neobacillus drentensis* | no | No | RiPP | LAP | unknown | ND |
| JJ1417 | *Neobacillus drentensis* | no | No | Terpene | terpene | unknown | ND |
| JJ1417 | *Neobacillus drentensis* | no | No | Terpene | terpene | unknown | ND |
| JJ1417 | *Neobacillus drentensis* | no | No | RiPP | bacteriocin | unknown | ND |
| JJ1419 | *Bacillus toyonensis* | no | Sparse | NRPS | NRPS | bacillibactin | 46 |
| JJ1419 | *Bacillus toyonensis* | no | Sparse | NRPS | NRPS | cyanopeptin | 50 |
| JJ1419 | *Bacillus toyonensis* | no | Sparse | Other | betalactone | fengycin | 40 |
| JJ1419 | *Bacillus toyonensis* | no | Sparse | Terpene | terpene | molybdenum cofactor | 17 |
| JJ1419 | *Bacillus toyonensis* | no | Sparse | NRPS | NRPS | unknown | ND |
| JJ1419 | *Bacillus toyonensis* | no | Sparse | RiPP | LAP | unknown | ND |
| JJ1419 | *Bacillus toyonensis* | no | Sparse | NRPS | NRPS | unknown | ND |
| JJ1419 | *Bacillus toyonensis* | no | Sparse | RiPP | bacteriocin | unknown | ND |
| JJ1419 | *Bacillus toyonensis* | no | Sparse | RiPP | lassopeptide | paeninodin | 80 |
| JJ1419 | *Bacillus toyonensis* | no | Sparse | Other | siderophore | petrobactin | 100 |
| JJ1426 | *Bacillus (other)* | no | No | PKS | T3PKS | unknown | ND |
| JJ1426 | *Bacillus (other)* | no | No | Terpene | terpene | unknown | ND |
| JJ1426 | *Bacillus (other)* | no | No | RiPP | LAP | unknown | ND |
| JJ1426 | *Bacillus (other)* | no | No | NRPS | NRPS | unknown | ND |
| JJ1428 | *Priestia megaterium* | no | No | Terpene | terpene | carotenoid | 50 |
| JJ1428 | *Priestia megaterium* | no | No | Terpene | terpene | unknown | ND |
| JJ1428 | *Priestia megaterium* | no | No | PKS | T3PKS | unknown | ND |
| JJ1428 | *Priestia megaterium* | no | No | Other | siderophore | unknown | ND |
| JJ1428 | *Priestia megaterium* | no | No | Other | phosphonate | unknown | ND |
| JJ1428 | *Priestia megaterium* | no | No | Terpene | terpene | surfactin | 13 |
| JJ1434 | *Priestia megaterium* | no | No | Terpene | terpene | carotenoid | 50 |
| JJ1434 | *Priestia megaterium* | no | No | PKS | T3PKS | unknown | ND |
| JJ1434 | *Priestia megaterium* | no | No | Terpene | terpene | unknown | ND |
| JJ1434 | *Priestia megaterium* | no | No | Other | siderophore | unknown | ND |
| JJ1434 | *Priestia megaterium* | no | No | Other | phosphonate | unknown | ND |
| JJ1434 | *Priestia megaterium* | no | No | Terpene | terpene | surfactin | 13 |
| JJ1435 | *Neobacillus drentensis* | no | No | PKS | T3PKS | unknown | ND |
| JJ1435 | *Neobacillus drentensis* | no | No | Other | phosphonate | unknown | ND |
| JJ1435 | *Neobacillus drentensis* | no | No | NRPS | NRPS | unknown | ND |
| JJ1435 | *Neobacillus drentensis* | no | No | RiPP | LAP | unknown | ND |
| JJ1435 | *Neobacillus drentensis* | no | No | Terpene | terpene | unknown | ND |
| JJ1440 | *Priestia megaterium* | no | No | PKS | transAT-PKS-like | calyculin A | 8 |
| JJ1440 | *Priestia megaterium* | no | No | Terpene | terpene | carotenoid | 50 |
| JJ1440 | *Priestia megaterium* | no | No | PKS | PKS-like | elansolid A | 35 |
| JJ1440 | *Priestia megaterium* | no | No | PKS | T3PKS | unknown | ND |
| JJ1440 | *Priestia megaterium* | no | No | Other | phosphonate | unknown | ND |
| JJ1440 | *Priestia megaterium* | no | No | Terpene | terpene | unknown | ND |
| JJ1440 | *Priestia megaterium* | no | No | Other | siderophore | unknown | ND |
| JJ1440 | *Priestia megaterium* | no | No | RiPP | bacteriocin | unknown | ND |
| JJ1440 | *Priestia megaterium* | no | No | PKS | transAT-PKS-like | scytophycin | 27 |
| JJ1440 | *Priestia megaterium* | no | No | Terpene | terpene | surfactin | 13 |
| JJ1441 | *Bacillus toyonensis* | no | Sparse | NRPS | NRPS | bacillibactin | 46 |
| JJ1441 | *Bacillus toyonensis* | no | Sparse | Other | betalactone | fengycin | 33 |
| JJ1441 | *Bacillus toyonensis* | no | Sparse | Terpene | terpene | molybdenum cofactor | 17 |
| JJ1441 | *Bacillus toyonensis* | no | Sparse | NRPS | NRPS | unknown | ND |
| JJ1441 | *Bacillus toyonensis* | no | Sparse | NRPS | NRPS | unknown | ND |
| JJ1441 | *Bacillus toyonensis* | no | Sparse | RiPP | LAP | unknown | ND |
| JJ1441 | *Bacillus toyonensis* | no | Sparse | NRPS | NRPS-like | unknown | ND |
| JJ1441 | *Bacillus toyonensis* | no | Sparse | RiPP | lassopeptide | paeninodin | 100 |
| JJ1441 | *Bacillus toyonensis* | no | Sparse | Other | siderophore | petrobactin | 100 |
| JJ1446 | *Gottfriedia acidiceleris* | no | No | PKS | T3PKS | unknown | ND |
| JJ1446 | *Gottfriedia acidiceleris* | no | No | Terpene | terpene | unknown | ND |
| JJ1446 | *Gottfriedia acidiceleris* | no | No | Terpene | terpene | unknown | ND |
| JJ1446 | *Gottfriedia acidiceleris* | no | No | Other | siderophore | unknown | ND |
| JJ1446 | *Gottfriedia acidiceleris* | no | No | RiPP | LAP | unknown | ND |
| JJ1473 | *Bacillus toyonensis* | weak | Sparse | NRPS | NRPS | bacillibactin | 46 |
| JJ1473 | *Bacillus toyonensis* | weak | Sparse | RiPP | LAP | unknown | ND |
| JJ1473 | *Bacillus toyonensis* | weak | Sparse | NRPS | NRPS-like | unknown | ND |
| JJ1473 | *Bacillus toyonensis* | weak | Sparse | Terpene | terpene | unknown | ND |
| JJ1473 | *Bacillus toyonensis* | weak | Sparse | Other | betalactone | unknown | ND |
| JJ1473 | *Bacillus toyonensis* | weak | Sparse | RiPP | lassopeptide | unknown | ND |
| JJ1473 | *Bacillus toyonensis* | weak | Sparse | Other | siderophore | petrobactin | 100 |
| JJ1474 | *Bacillus (other)* | no | No | Other | betalactone | fengycin | 40 |
| JJ1474 | *Bacillus (other)* | no | No | PKS | T3PKS | unknown | ND |
| JJ1474 | *Bacillus (other)* | no | No | RiPP | LAP | unknown | ND |
| JJ1474 | *Bacillus (other)* | no | No | Other | phosphonate | unknown | ND |
| JJ1474 | *Bacillus (other)* | no | No | Terpene | terpene | unknown | ND |
| JJ1474 | *Bacillus (other)* | no | No | Terpene | terpene | unknown | ND |
| JJ1493 | *Bacillus toyonensis* | strong | Sparse | NRPS | NRPS | unknown | ND |
| JJ1493 | *Bacillus toyonensis* | strong | Sparse | Terpene | terpene | unknown | ND |
| JJ1493 | *Bacillus toyonensis* | strong | Sparse | RiPP | bacteriocin | unknown | ND |
| JJ1493 | *Bacillus toyonensis* | strong | Sparse | Other | betalactone | unknown | ND |
| JJ1493 | *Bacillus toyonensis* | strong | Sparse | NRPS | NRPS | nostopeptolide A | 37 |
| JJ1493 | *Bacillus toyonensis* | strong | Sparse | Other | siderophore | petrobactin | 83 |
| JJ1499 | *Neobacillus drentensis* | no | No | Other | betalactone | fengycin | 40 |
| JJ1499 | *Neobacillus drentensis* | no | No | PKS | T3PKS | unknown | ND |
| JJ1499 | *Neobacillus drentensis* | no | No | Terpene | terpene | unknown | ND |
| JJ1499 | *Neobacillus drentensis* | no | No | Terpene | terpene | unknown | ND |
| JJ1499 | *Neobacillus drentensis* | no | No | RiPP | LAP | unknown | ND |
| JJ1499 | *Neobacillus drentensis* | no | No | RiPP | bacteriocin | unknown | ND |
| JJ1502 | *Gottfriedia acidiceleris* | no | No | PKS | T3PKS | unknown | ND |
| JJ1502 | *Gottfriedia acidiceleris* | no | No | Other | betalactone | unknown | ND |
| JJ1502 | *Gottfriedia acidiceleris* | no | No | Terpene | terpene | unknown | ND |
| JJ1502 | *Gottfriedia acidiceleris* | no | No | Terpene | terpene | unknown | ND |
| JJ1502 | *Gottfriedia acidiceleris* | no | No | RiPP | LAP | unknown | ND |
| JJ1503 | *Bacillus (other)* | no | No | Other | betalactone | fengycin | 40 |
| JJ1503 | *Bacillus (other)* | no | No | PKS | T3PKS | unknown | ND |
| JJ1503 | *Bacillus (other)* | no | No | Other | phosphonate | unknown | ND |
| JJ1503 | *Bacillus (other)* | no | No | RiPP | bacteriocin | unknown | ND |
| JJ1503 | *Bacillus (other)* | no | No | Terpene | terpene | unknown | ND |
| JJ1503 | *Bacillus (other)* | no | No | Terpene | terpene | unknown | ND |
| JJ1510 | *Gottfriedia acidiceleris* | no | No | RiPP | LAP | unknown | ND |
| JJ1510 | *Gottfriedia acidiceleris* | no | No | Terpene | terpene | unknown | ND |
| JJ1510 | *Gottfriedia acidiceleris* | no | No | Terpene | terpene | unknown | ND |
| JJ1510 | *Gottfriedia acidiceleris* | no | No | PKS | T3PKS | unknown | ND |
| JJ1510 | *Gottfriedia acidiceleris* | no | No | RiPP | bacteriocin | unknown | ND |
| JJ1521 | *Bacillus (other)* | no | No | RiPP | LAP | unknown | ND |
| JJ1521 | *Bacillus (other)* | no | No | Terpene | terpene | unknown | ND |
| JJ1521 | *Bacillus (other)* | no | No | PKS | T3PKS | unknown | ND |
| JJ1521 | *Bacillus (other)* | no | No | Terpene | terpene | unknown | ND |
| JJ1523 | *Neobacillus drentensis* | no | No | PKS-NRPS | transAT-PKS-like | bacillaene | 42 |
| JJ1523 | *Neobacillus drentensis* | no | No | NRPS | NRPS-like | butirosin A | 7 |
| JJ1523 | *Neobacillus drentensis* | no | No | RiPP | lanthipeptide | lichenicidin C | 100 |
| JJ1523 | *Neobacillus drentensis* | no | No | NRPS | NRPS-like | unknown | ND |
| JJ1523 | *Neobacillus drentensis* | no | No | PKS | transAT-PKS-like | unknown | ND |
| JJ1523 | *Neobacillus drentensis* | no | No | Terpene | terpene | unknown | ND |
| JJ1523 | *Neobacillus drentensis* | no | No | PKS | transAT-PKS-like | unknown | ND |
| JJ1523 | *Neobacillus drentensis* | no | No | RiPP | bacteriocin | unknown | ND |
| JJ1523 | *Neobacillus drentensis* | no | No | PKS | transAT-PKS-like | oxazolepoxidomycin A | 26 |
| JJ1523 | *Neobacillus drentensis* | no | No | RiPP | lassopeptide | paeninodin | 100 |
| JJ1523 | *Neobacillus drentensis* | no | No | PKS | PKS-like | phormidolide | 8 |
| JJ1532 | *Bacillus (other)* | no | No | Other | betalactone | fengycin | 33 |
| JJ1532 | *Bacillus (other)* | no | No | PKS | T3PKS | unknown | ND |
| JJ1532 | *Bacillus (other)* | no | No | Other | phosphonate | unknown | ND |
| JJ1532 | *Bacillus (other)* | no | No | RiPP | LAP | unknown | ND |
| JJ1532 | *Bacillus (other)* | no | No | Terpene | terpene | unknown | ND |
| JJ1532 | *Bacillus (other)* | no | No | Terpene | terpene | unknown | ND |
| JJ1533 | *Bacillus (other)* | no | No | PKS | T3PKS | unknown | ND |
| JJ1533 | *Bacillus (other)* | no | No | RiPP | LAP | unknown | ND |
| JJ1533 | *Bacillus (other)* | no | No | Terpene | terpene | unknown | ND |
| JJ1533 | *Bacillus (other)* | no | No | Terpene | terpene | unknown | ND |
| JJ1533 | *Bacillus (other)* | no | No | RiPP | lassopeptide | paeninodin | 80 |
| JJ1535 | *Priestia megaterium* | no | No | Terpene | terpene | carotenoid | 50 |
| JJ1535 | *Priestia megaterium* | no | No | Other | phosphonate | unknown | ND |
| JJ1535 | *Priestia megaterium* | no | No | PKS | T3PKS | unknown | ND |
| JJ1535 | *Priestia megaterium* | no | No | Terpene | terpene | unknown | ND |
| JJ1535 | *Priestia megaterium* | no | No | Other | siderophore | unknown | ND |
| JJ1535 | *Priestia megaterium* | no | No | RiPP | bacteriocin | unknown | ND |
| JJ1535 | *Priestia megaterium* | no | No | Terpene | terpene | surfactin | 13 |
| JJ155 | *Cytobacillus firmus* | no | Sparse | PKS | T3PKS | unknown | ND |
| JJ155 | *Cytobacillus firmus* | no | Sparse | RiPP | lanthipeptide | unknown | ND |
| JJ155 | *Cytobacillus firmus* | no | Sparse | Terpene | terpene | unknown | ND |
| JJ155 | *Cytobacillus firmus* | no | Sparse | Other | siderophore | petrobactin | 33 |
| JJ1551 | *Neobacillus drentensis* | no | No | Other | betalactone | fengycin | 33 |
| JJ1551 | *Neobacillus drentensis* | no | No | PKS | T3PKS | unknown | ND |
| JJ1551 | *Neobacillus drentensis* | no | No | RiPP | LAP | unknown | ND |
| JJ1551 | *Neobacillus drentensis* | no | No | Terpene | terpene | unknown | ND |
| JJ1551 | *Neobacillus drentensis* | no | No | Terpene | terpene | unknown | ND |
| JJ1557 | *Neobacillus drentensis* | no | No | Terpene | terpene | carotenoid | 33 |
| JJ1557 | *Neobacillus drentensis* | no | No | PKS | T3PKS | unknown | ND |
| JJ1557 | *Neobacillus drentensis* | no | No | RiPP | LAP | unknown | ND |
| JJ1557 | *Neobacillus drentensis* | no | No | Terpene | terpene | unknown | ND |
| JJ1557 | *Neobacillus drentensis* | no | No | RiPP | lassopeptide | paeninodin | 80 |
| JJ1562 | *Bacillus (other)* | no | No | RiPP | LAP | kijanimicin | 4 |
| JJ1562 | *Bacillus (other)* | no | No | PKS | T3PKS | unknown | ND |
| JJ1562 | *Bacillus (other)* | no | No | RiPP | LAP | unknown | ND |
| JJ1562 | *Bacillus (other)* | no | No | Terpene | terpene | unknown | ND |
| JJ1562 | *Bacillus (other)* | no | No | Terpene | terpene | unknown | ND |
| JJ1566 | *Bacillus (other)* | no | No | PKS | T3PKS | unknown | ND |
| JJ1566 | *Bacillus (other)* | no | No | RiPP | LAP | unknown | ND |
| JJ1566 | *Bacillus (other)* | no | No | Terpene | terpene | unknown | ND |
| JJ1566 | *Bacillus (other)* | no | No | Terpene | terpene | unknown | ND |
| JJ1566 | *Bacillus (other)* | no | No | RiPP | lassopeptide | paeninodin | 100 |
| JJ1568 | *Neobacillus niacini* | no | No | RiPP | LAP | unknown | ND |
| JJ1568 | *Neobacillus niacini* | no | No | Other | betalactone | unknown | ND |
| JJ1568 | *Neobacillus niacini* | no | No | PKS | T3PKS | unknown | ND |
| JJ1568 | *Neobacillus niacini* | no | No | Terpene | terpene | unknown | ND |
| JJ1568 | *Neobacillus niacini* | no | No | RiPP | bacteriocin | unknown | ND |
| JJ157 | *Bacillus (other)* | no | No | Terpene | terpene | carotenoid | 50 |
| JJ157 | *Bacillus (other)* | no | No | RiPP | lanthipeptide | cytolysin | 40 |
| JJ157 | *Bacillus (other)* | no | No | PKS | T3PKS | unknown | ND |
| JJ157 | *Bacillus (other)* | no | No | Other | phosphonate | unknown | ND |
| JJ157 | *Bacillus (other)* | no | No | Other | siderophore | unknown | ND |
| JJ157 | *Bacillus (other)* | no | No | Terpene | terpene | unknown | ND |
| JJ157 | *Bacillus (other)* | no | No | Terpene | terpene | surfactin | 13 |
| JJ1571 | *Neobacillus niacini* | no | No | RiPP | LAP | unknown | ND |
| JJ1571 | *Neobacillus niacini* | no | No | RiPP | LAP | unknown | ND |
| JJ1571 | *Neobacillus niacini* | no | No | Terpene | terpene | unknown | ND |
| JJ1571 | *Neobacillus niacini* | no | No | RiPP | bacteriocin | unknown | ND |
| JJ1571 | *Neobacillus niacini* | no | No | RiPP | lassopeptide | paeninodin | 100 |
| JJ1576 | *Priestia megaterium* | no | No | Terpene | terpene | carotenoid | 50 |
| JJ1576 | *Priestia megaterium* | no | No | Other | phosphonate | unknown | ND |
| JJ1576 | *Priestia megaterium* | no | No | RiPP | lanthipeptide | unknown | ND |
| JJ1576 | *Priestia megaterium* | no | No | Terpene | terpene | unknown | ND |
| JJ1576 | *Priestia megaterium* | no | No | PKS | T3PKS | unknown | ND |
| JJ1576 | *Priestia megaterium* | no | No | Other | siderophore | unknown | ND |
| JJ1576 | *Priestia megaterium* | no | No | Terpene | terpene | surfactin | 13 |
| JJ158 | *Bacillus velezensis* | weak | Strong | PKS-NRPS | transAT-PKS | bacillaene | 100 |
| JJ158 | *Bacillus velezensis* | weak | Strong | NRPS | NRPS | bacillibactin | 100 |
| JJ158 | *Bacillus velezensis* | weak | Strong | Other | other | bacilysin | 100 |
| JJ158 | *Bacillus velezensis* | weak | Strong | PKS | PKS-like | butirosin A | 7 |
| JJ158 | *Bacillus velezensis* | weak | Strong | PKS | transAT-PKS-like | difficidin | 53 |
| JJ158 | *Bacillus velezensis* | weak | Strong | NRPS | NRPS | fengycin | 80 |
| JJ158 | *Bacillus velezensis* | weak | Strong | PKS | transAT-PKS | macrolactin H | 100 |
| JJ158 | *Bacillus velezensis* | weak | Strong | PKS | T3PKS | unknown | ND |
| JJ158 | *Bacillus velezensis* | weak | Strong | Terpene | terpene | unknown | ND |
| JJ158 | *Bacillus velezensis* | weak | Strong | Terpene | terpene | unknown | ND |
| JJ158 | *Bacillus velezensis* | weak | Strong | RiPP | LAP | plantazolicin | 91 |
| JJ158 | *Bacillus velezensis* | weak | Strong | NRPS | NRPS | surfactin | 47 |
| JJ158 | *Bacillus velezensis* | weak | Strong | NRPS | Others | iturin/Bacilomycin L | 80 |
| JJ1589 | *Bacillus altitudinis* | no | Sparse | Other | other | bacilysin | 85 |
| JJ1589 | *Bacillus altitudinis* | no | Sparse | Other | betalactone | bottromycin A | 6 |
| JJ1589 | *Bacillus altitudinis* | no | Sparse | Terpene | terpene | carotenoid | 50 |
| JJ1589 | *Bacillus altitudinis* | no | Sparse | Other | betalactone | fengycin | 53 |
| JJ1589 | *Bacillus altitudinis* | no | Sparse | NRPS | NRPS | lichenysin | 85 |
| JJ1589 | *Bacillus altitudinis* | no | Sparse | PKS | T3PKS | unknown | ND |
| JJ1589 | *Bacillus altitudinis* | no | Sparse | Terpene | terpene | unknown | ND |
| JJ1589 | *Bacillus altitudinis* | no | Sparse | RiPP | bacteriocin | unknown | ND |
| JJ1589 | *Bacillus altitudinis* | no | Sparse | RiPP | bacteriocin | unknown | ND |
| JJ1590 | *Bacillus thuringiensis* | weak | Sparse | NRPS | siderophore | fengycin | 40 |
| JJ1590 | *Bacillus thuringiensis* | weak | Sparse | Terpene | terpene | molybdenum cofactor | 17 |
| JJ1590 | *Bacillus thuringiensis* | weak | Sparse | NRPS | NRPS | unknown | ND |
| JJ1590 | *Bacillus thuringiensis* | weak | Sparse | NRPS | NRPS-like | unknown | ND |
| JJ1590 | *Bacillus thuringiensis* | weak | Sparse | NRPS | NRPS | unknown | ND |
| JJ1590 | *Bacillus thuringiensis* | weak | Sparse | RiPP | LAP | unknown | ND |
| JJ1590 | *Bacillus thuringiensis* | weak | Sparse | PKS-NRPS | NRPS | zwittermicin A | 44 |
| JJ1605 | *Bacillus safensis* | no | Strong | NRPS | NRPS | bacillibactin | 53 |
| JJ1605 | *Bacillus safensis* | no | Strong | Other | other | bacilysin | 85 |
| JJ1605 | *Bacillus safensis* | no | Strong | Other | other | carotenoid | 50 |
| JJ1605 | *Bacillus safensis* | no | Strong | Other | betalactone | fengycin | 53 |
| JJ1605 | *Bacillus safensis* | no | Strong | NRPS | NRPS | lichenysin | 28 |
| JJ1605 | *Bacillus safensis* | no | Strong | Other | betalactone | unknown | ND |
| JJ1605 | *Bacillus safensis* | no | Strong | Terpene | terpene | unknown | ND |
| JJ1605 | *Bacillus safensis* | no | Strong | PKS | T3PKS | unknown | ND |
| JJ1605 | *Bacillus safensis* | no | Strong | NRPS | NRPS | unknown | ND |
| JJ1605 | *Bacillus safensis* | no | Strong | RiPP | LAP | plantazolicin | 41 |
| JJ1605 | *Bacillus safensis* | no | Strong | RiPP | sactipeptide | sporulation killing factor | 85 |
| JJ1609 | *Bacillus (other)* | strong | No | PKS | T3PKS | unknown | ND |
| JJ1609 | *Bacillus (other)* | strong | No | RiPP | LAP | unknown | ND |
| JJ1609 | *Bacillus (other)* | strong | No | RiPP | lassopeptide | paeninodin | 80 |
| JJ1622 | *Bacillus pumilus* | strong | Strong | NRPS | NRPS | lichenysin | 85 |
| JJ1622 | *Bacillus pumilus* | strong | Strong | PKS-NRPS | NRPS | zwittermicin A | 18 |
| JJ1622 | *Bacillus pumilus* | strong | Strong | RiPP | RRE-containing | unknown | ND |
| JJ1622 | *Bacillus pumilus* | strong | Strong | Terpene | terpene | carotenoid | 50 |
| JJ1622 | *Bacillus pumilus* | strong | Strong | Other | betalactone | fengycin | 53 |
| JJ1622 | *Bacillus pumilus* | strong | Strong | Terpene | terpene | unknown | ND |
| JJ1622 | *Bacillus pumilus* | strong | Strong | PKS | T3PKS | unknown | ND |
| JJ1622 | *Bacillus pumilus* | strong | Strong | Other | betalactone | unknown | ND |
| JJ1622 | *Bacillus pumilus* | strong | Strong | RiPP | RiPP-like | unknown | ND |
| JJ1622 | *Bacillus pumilus* | strong | Strong | Other | other | bacilysin | 85 |
| JJ1622 | *Bacillus pumilus* | strong | Strong | NRPS | NRPS | bacillibactin | 53 |
| JJ1626 | *Bacillus safensis* | strong | Strong | NRPS | NRPS | bacillibactin | 53 |
| JJ1626 | *Bacillus safensis* | strong | Strong | Other | other | bacilysin | 85 |
| JJ1626 | *Bacillus safensis* | strong | Strong | Other | betalactone | bottromycin A | 6 |
| JJ1626 | *Bacillus safensis* | strong | Strong | Other | other | carotenoid | 50 |
| JJ1626 | *Bacillus safensis* | strong | Strong | NRPS | NRPS | lichenysin | 50 |
| JJ1626 | *Bacillus safensis* | strong | Strong | RiPP | bacteriocin | unknown | ND |
| JJ1626 | *Bacillus safensis* | strong | Strong | PKS | T3PKS | unknown | ND |
| JJ1626 | *Bacillus safensis* | strong | Strong | RiPP | LAP | plantazolicin | 50 |
| JJ1626 | *Bacillus safensis* | strong | Strong | Other | betalactone | fengycin | 53 |
| JJ1627 | *Bacillus (other)* | no | No | NRPS | NRPS | bacillibactin | 46 |
| JJ1627 | *Bacillus (other)* | no | No | NRPS | NRPS | bacitracin | 33 |
| JJ1627 | *Bacillus (other)* | no | No | Other | betalactone | fengycin | 40 |
| JJ1627 | *Bacillus (other)* | no | No | Terpene | terpene | molybdenum cofactor | 17 |
| JJ1627 | *Bacillus (other)* | no | No | RiPP | LAP | unknown | ND |
| JJ1627 | *Bacillus (other)* | no | No | NRPS | NRPS | unknown | ND |
| JJ1627 | *Bacillus (other)* | no | No | RiPP | bacteriocin | unknown | ND |
| JJ1627 | *Bacillus (other)* | no | No | Other | siderophore | petrobactin | 100 |
| JJ1634 | *Priestia megaterium* | no | No | Terpene | terpene | carotenoid | 50 |
| JJ1634 | *Priestia megaterium* | no | No | PKS | T3PKS | unknown | ND |
| JJ1634 | *Priestia megaterium* | no | No | Other | phosphonate | unknown | ND |
| JJ1634 | *Priestia megaterium* | no | No | Terpene | terpene | unknown | ND |
| JJ1634 | *Priestia megaterium* | no | No | RiPP | bacteriocin | unknown | ND |
| JJ1634 | *Priestia megaterium* | no | No | RiPP | lanthipeptide | unknown | ND |
| JJ1634 | *Priestia megaterium* | no | No | Terpene | terpene | surfactin | 13 |
| JJ1637 | *Bacillus toyonensis* | no | Sparse | NRPS | NRPS | bacillibactin | 46 |
| JJ1637 | *Bacillus toyonensis* | no | Sparse | Other | betalactone | fengycin | 40 |
| JJ1637 | *Bacillus toyonensis* | no | Sparse | Terpene | terpene | molybdenum cofactor | 17 |
| JJ1637 | *Bacillus toyonensis* | no | Sparse | NRPS | NRPS-like | unknown | ND |
| JJ1637 | *Bacillus toyonensis* | no | Sparse | RiPP | LAP | unknown | ND |
| JJ1637 | *Bacillus toyonensis* | no | Sparse | NRPS | NRPS | unknown | ND |
| JJ1637 | *Bacillus toyonensis* | no | Sparse | RiPP | bacteriocin | unknown | ND |
| JJ1637 | *Bacillus toyonensis* | no | Sparse | RiPP | lassopeptide | paeninodin | 80 |
| JJ1637 | *Bacillus toyonensis* | no | Sparse | Other | siderophore | petrobactin | 100 |
| JJ1637 | *Bacillus toyonensis* | no | Sparse | RiPP | lanthipeptide | thuricin | 83 |
| JJ1641 | *Bacillus (other)* | no | No | Terpene | terpene | carotenoid | 33 |
| JJ1641 | *Bacillus (other)* | no | No | PKS | transAT-PKS-like | locillomycin | 14 |
| JJ1641 | *Bacillus (other)* | no | No | Terpene | terpene | unknown | ND |
| JJ1641 | *Bacillus (other)* | no | No | NRPS | NRPS | unknown | ND |
| JJ1641 | *Bacillus (other)* | no | No | NRPS | NRPS | unknown | ND |
| JJ1641 | *Bacillus (other)* | no | No | RiPP | lassopeptide | paeninodin | 60 |
| JJ1651 | *Bacillus toyonensis* | weak | Sparse | NRPS | NRPS | bacillibactin | 15 |
| JJ1651 | *Bacillus toyonensis* | weak | Sparse | Other | betalactone | fengycin | 26 |
| JJ1651 | *Bacillus toyonensis* | weak | Sparse | Terpene | terpene | molybdenum cofactor | 17 |
| JJ1651 | *Bacillus toyonensis* | weak | Sparse | NRPS | NRPS | unknown | ND |
| JJ1651 | *Bacillus toyonensis* | weak | Sparse | RiPP | LAP | unknown | ND |
| JJ1651 | *Bacillus toyonensis* | weak | Sparse | NRPS | NRPS-like | unknown | ND |
| JJ1651 | *Bacillus toyonensis* | weak | Sparse | NRPS | NRPS | unknown | ND |
| JJ1651 | *Bacillus toyonensis* | weak | Sparse | Other | siderophore | petrobactin | 100 |
| JJ1657 | *Priestia megaterium* | no | No | Terpene | terpene | carotenoid | 50 |
| JJ1657 | *Priestia megaterium* | no | No | Other | phosphonate | unknown | ND |
| JJ1657 | *Priestia megaterium* | no | No | PKS | T3PKS | unknown | ND |
| JJ1657 | *Priestia megaterium* | no | No | Terpene | terpene | unknown | ND |
| JJ1657 | *Priestia megaterium* | no | No | RiPP | lanthipeptide | unknown | ND |
| JJ1657 | *Priestia megaterium* | no | No | Other | siderophore | unknown | ND |
| JJ1657 | *Priestia megaterium* | no | No | Terpene | terpene | surfactin | 13 |
| JJ1659 | *Bacillus toyonensis* | no | Sparse | NRPS | NRPS | bacillibactin | 46 |
| JJ1659 | *Bacillus toyonensis* | no | Sparse | NRPS | NRPS | cyanopeptin | 50 |
| JJ1659 | *Bacillus toyonensis* | no | Sparse | NRPS | NRPS | unknown | ND |
| JJ1659 | *Bacillus toyonensis* | no | Sparse | RiPP | LAP | unknown | ND |
| JJ1659 | *Bacillus toyonensis* | no | Sparse | Terpene | terpene | unknown | ND |
| JJ1659 | *Bacillus toyonensis* | no | Sparse | RiPP | bacteriocin | unknown | ND |
| JJ1659 | *Bacillus toyonensis* | no | Sparse | NRPS | NRPS | unknown | ND |
| JJ1659 | *Bacillus toyonensis* | no | Sparse | RiPP | lassopeptide | paeninodin | 80 |
| JJ1659 | *Bacillus toyonensis* | no | Sparse | Other | siderophore | petrobactin | 100 |
| JJ1659 | *Bacillus toyonensis* | no | Sparse | NRPS | NRPS | rhizomide A | 100 |
| JJ1663 | *Priestia megaterium* | no | No | Terpene | terpene | carotenoid | 50 |
| JJ1663 | *Priestia megaterium* | no | No | PKS | T3PKS | unknown | ND |
| JJ1663 | *Priestia megaterium* | no | No | Other | phosphonate | unknown | ND |
| JJ1663 | *Priestia megaterium* | no | No | Terpene | terpene | unknown | ND |
| JJ1663 | *Priestia megaterium* | no | No | Other | siderophore | unknown | ND |
| JJ1663 | *Priestia megaterium* | no | No | RiPP | lassopeptide | paeninodin | 60 |
| JJ1663 | *Priestia megaterium* | no | No | Terpene | terpene | surfactin | 13 |
| JJ1682 | *Priestia megaterium* | no | No | Terpene | terpene | carotenoid | 50 |
| JJ1682 | *Priestia megaterium* | no | No | PKS | transAT-PKS-like | difficidin | 13 |
| JJ1682 | *Priestia megaterium* | no | No | PKS | PKS-like | elansolid A | 30 |
| JJ1682 | *Priestia megaterium* | no | No | PKS | transAT-PKS-like | macrobrevin | 40 |
| JJ1682 | *Priestia megaterium* | no | No | PKS | T3PKS | unknown | ND |
| JJ1682 | *Priestia megaterium* | no | No | Terpene | terpene | unknown | ND |
| JJ1682 | *Priestia megaterium* | no | No | Other | siderophore | unknown | ND |
| JJ1682 | *Priestia megaterium* | no | No | Other | phosphonate | unknown | ND |
| JJ1682 | *Priestia megaterium* | no | No | RiPP | bacteriocin | unknown | ND |
| JJ1682 | *Priestia megaterium* | no | No | Terpene | terpene | surfactin | 13 |
| JJ1682 | *Priestia megaterium* | no | No | PKS | transAT-PKS-like | tetrounknownsin | 9 |
| JJ1685 | *Bacillus toyonensis* | no | Sparse | NRPS | NRPS | cyanopeptin | 50 |
| JJ1685 | *Bacillus toyonensis* | no | Sparse | NRPS | NRPS | unknown | ND |
| JJ1685 | *Bacillus toyonensis* | no | Sparse | RiPP | LAP | unknown | ND |
| JJ1685 | *Bacillus toyonensis* | no | Sparse | Other | betalactone | unknown | ND |
| JJ1685 | *Bacillus toyonensis* | no | Sparse | Terpene | terpene | unknown | ND |
| JJ1685 | *Bacillus toyonensis* | no | Sparse | Other | siderophore | unknown | ND |
| JJ1685 | *Bacillus toyonensis* | no | Sparse | RiPP | lassopeptide | paeninodin | 100 |
| JJ1691 | *Priestia megaterium* | no | No | Terpene | terpene | carotenoid | 50 |
| JJ1691 | *Priestia megaterium* | no | No | Other | phosphonate | unknown | ND |
| JJ1691 | *Priestia megaterium* | no | No | PKS | T3PKS | unknown | ND |
| JJ1691 | *Priestia megaterium* | no | No | Terpene | terpene | unknown | ND |
| JJ1691 | *Priestia megaterium* | no | No | Other | siderophore | unknown | ND |
| JJ1691 | *Priestia megaterium* | no | No | Terpene | terpene | surfactin | 13 |
| JJ1707 | *Bacillus (other)* | no | No | Terpene | terpene | carotenoid | 33 |
| JJ1707 | *Bacillus (other)* | no | No | NRPS | NRPS | unknown | ND |
| JJ1707 | *Bacillus (other)* | no | No | Terpene | terpene | unknown | ND |
| JJ1708 | *Priestia megaterium* | weak | No | Terpene | terpene | carotenoid | 50 |
| JJ1708 | *Priestia megaterium* | weak | No | PKS | T3PKS | unknown | ND |
| JJ1708 | *Priestia megaterium* | weak | No | Terpene | terpene | unknown | ND |
| JJ1708 | *Priestia megaterium* | weak | No | Other | siderophore | unknown | ND |
| JJ1708 | *Priestia megaterium* | weak | No | Other | phosphonate | unknown | ND |
| JJ1708 | *Priestia megaterium* | weak | No | Terpene | terpene | surfactin | 13 |
| JJ1712 | *Priestia megaterium* | no | No | Terpene | terpene | carotenoid | 50 |
| JJ1712 | *Priestia megaterium* | no | No | PKS | T3PKS | unknown | ND |
| JJ1712 | *Priestia megaterium* | no | No | Other | phosphonate | unknown | ND |
| JJ1712 | *Priestia megaterium* | no | No | Terpene | terpene | unknown | ND |
| JJ1712 | *Priestia megaterium* | no | No | Other | siderophore | unknown | ND |
| JJ1712 | *Priestia megaterium* | no | No | Terpene | terpene | surfactin | 13 |
| JJ1714 | *Bacillus thuringiensis* | weak | Sparse | NRPS | NRPS | bacillibactin | 46 |
| JJ1714 | *Bacillus thuringiensis* | weak | Sparse | Other | betalactone | fengycin | 40 |
| JJ1714 | *Bacillus thuringiensis* | weak | Sparse | Terpene | terpene | molybdenum cofactor | 17 |
| JJ1714 | *Bacillus thuringiensis* | weak | Sparse | NRPS | NRPS | unknown | ND |
| JJ1714 | *Bacillus thuringiensis* | weak | Sparse | RiPP | LAP | unknown | ND |
| JJ1714 | *Bacillus thuringiensis* | weak | Sparse | NRPS | NRPS | unknown | ND |
| JJ1714 | *Bacillus thuringiensis* | weak | Sparse | RiPP | sactipeptide | unknown | ND |
| JJ1714 | *Bacillus thuringiensis* | weak | Sparse | Other | siderophore | petrobactin | 100 |
| JJ1714 | *Bacillus thuringiensis* | weak | Sparse | NRPS | NRPS | polyoxypeptin | 5 |
| JJ1714 | *Bacillus thuringiensis* | weak | Sparse | Other | ladderane | S-layerglycan | 26 |
| JJ1714 | *Bacillus thuringiensis* | weak | Sparse | RiPP | bacteriocin | unknown | ND |
| JJ1714 | *Bacillus thuringiensis* | weak | Sparse | NRPS | NRPS-like | unknown | ND |
| JJ1728 | *Priestia megaterium* | no | No | Terpene | terpene | carotenoid | 50 |
| JJ1728 | *Priestia megaterium* | no | No | Other | phosphonate | unknown | ND |
| JJ1728 | *Priestia megaterium* | no | No | PKS | T3PKS | unknown | ND |
| JJ1728 | *Priestia megaterium* | no | No | Terpene | terpene | unknown | ND |
| JJ1728 | *Priestia megaterium* | no | No | Other | siderophore | unknown | ND |
| JJ1728 | *Priestia megaterium* | no | No | RiPP | lanthipeptide | unknown | ND |
| JJ1728 | *Priestia megaterium* | no | No | Terpene | terpene | surfactin | 8 |
| JJ1737 | *Neobacillus drentensis* | no | No | PKS | T3PKS | unknown | ND |
| JJ1737 | *Neobacillus drentensis* | no | No | Terpene | terpene | unknown | ND |
| JJ1737 | *Neobacillus drentensis* | no | No | RiPP | bacteriocin | unknown | ND |
| JJ1737 | *Neobacillus drentensis* | no | No | RiPP | lassopeptide | paeninodin | 80 |
| JJ1752 | *Bacillus safensis* | no | Strong | NRPS | NRPS | bacillibactin | 53 |
| JJ1752 | *Bacillus safensis* | no | Strong | Terpene | terpene | carotenoid | 50 |
| JJ1752 | *Bacillus safensis* | no | Strong | Other | betalactone | fengycin | 53 |
| JJ1752 | *Bacillus safensis* | no | Strong | NRPS | NRPS | lichenysin | 78 |
| JJ1752 | *Bacillus safensis* | no | Strong | PKS | T3PKS | unknown | ND |
| JJ1752 | *Bacillus safensis* | no | Strong | Other | betalactone | unknown | ND |
| JJ1752 | *Bacillus safensis* | no | Strong | NRPS | NRPS | unknown | ND |
| JJ1752 | *Bacillus safensis* | no | Strong | Terpene | terpene | unknown | ND |
| JJ1752 | *Bacillus safensis* | no | Strong | RiPP | bacteriocin | unknown | ND |
| JJ1752 | *Bacillus safensis* | no | Strong | NRPS | NRPS | paenilarvins | 75 |
| JJ1754 | *Bacillus (other)* | no | No | Terpene | terpene | carotenoid | 50 |
| JJ1754 | *Bacillus (other)* | no | No | Other | phosphonate | unknown | ND |
| JJ1754 | *Bacillus (other)* | no | No | PKS | T3PKS | unknown | ND |
| JJ1754 | *Bacillus (other)* | no | No | Terpene | terpene | unknown | ND |
| JJ1754 | *Bacillus (other)* | no | No | Other | siderophore | unknown | ND |
| JJ1754 | *Bacillus (other)* | no | No | Terpene | terpene | surfactin | 13 |
| JJ1764 | *Bacillus (other)* | no | No | RiPP | LAP | unknown | ND |
| JJ1764 | *Bacillus (other)* | no | No | RiPP | sactipeptide | unknown | ND |
| JJ1764 | *Bacillus (other)* | no | No | RiPP | bacteriocin | unknown | ND |
| JJ1764 | *Bacillus (other)* | no | No | RiPP | lassopeptide | paeninodin | 100 |
| JJ1770 | *Bacillus safensis* | strong | Strong | NRPS | NRPS | bacillibactin | 53 |
| JJ1770 | *Bacillus safensis* | strong | Strong | Other | other | bacilysin | 71 |
| JJ1770 | *Bacillus safensis* | strong | Strong | Other | other | carotenoid | 50 |
| JJ1770 | *Bacillus safensis* | strong | Strong | Other | betalactone | fengycin | 53 |
| JJ1770 | *Bacillus safensis* | strong | Strong | NRPS | NRPS | lichenysin | 28 |
| JJ1770 | *Bacillus safensis* | strong | Strong | PKS | T3PKS | unknown | ND |
| JJ1770 | *Bacillus safensis* | strong | Strong | Terpene | terpene | unknown | ND |
| JJ1770 | *Bacillus safensis* | strong | Strong | RiPP | LAP | plantazolicin | 33 |
| JJ1770 | *Bacillus safensis* | strong | Strong | Other | ladderane | S-layerglycan | 13 |
| JJ1770 | *Bacillus safensis* | strong | Strong | RiPP | head_to_tail | sporulation killing factor | 85 |
| JJ1773 | *Bacillus (other)* | no | No | Other | betalactone | fengycin | 33 |
| JJ1773 | *Bacillus (other)* | no | No | PKS | T3PKS | unknown | ND |
| JJ1773 | *Bacillus (other)* | no | No | Other | phosphonate | unknown | ND |
| JJ1773 | *Bacillus (other)* | no | No | Terpene | terpene | unknown | ND |
| JJ1773 | *Bacillus (other)* | no | No | RiPP | LAP | unknown | ND |
| JJ1773 | *Bacillus (other)* | no | No | Terpene | terpene | unknown | ND |
| JJ1773 | *Bacillus (other)* | no | No | RiPP | LAP | surfactin | 8 |
| JJ1779 | *Bacillus (other)* | no | No | Terpene | terpene | carotenoid | 50 |
| JJ1779 | *Bacillus (other)* | no | No | Other | phosphonate | unknown | ND |
| JJ1779 | *Bacillus (other)* | no | No | PKS | T3PKS | unknown | ND |
| JJ1779 | *Bacillus (other)* | no | No | Terpene | terpene | unknown | ND |
| JJ1779 | *Bacillus (other)* | no | No | Other | siderophore | unknown | ND |
| JJ1779 | *Bacillus (other)* | no | No | RiPP | lanthipeptide | unknown | ND |
| JJ1779 | *Bacillus (other)* | no | No | Terpene | terpene | surfactin | 8 |
| JJ1797 | *Priestia megaterium* | no | No | Terpene | terpene | carotenoid | 50 |
| JJ1797 | *Priestia megaterium* | no | No | PKS | T3PKS | unknown | ND |
| JJ1797 | *Priestia megaterium* | no | No | Other | phosphonate | unknown | ND |
| JJ1797 | *Priestia megaterium* | no | No | Terpene | terpene | unknown | ND |
| JJ1797 | *Priestia megaterium* | no | No | RiPP | lanthipeptide | unknown | ND |
| JJ1797 | *Priestia megaterium* | no | No | Other | siderophore | unknown | ND |
| JJ1797 | *Priestia megaterium* | no | No | Terpene | terpene | surfactin | 13 |
| JJ1805 | *Priestia megaterium* | no | No | Terpene | terpene | carotenoid | 50 |
| JJ1805 | *Priestia megaterium* | no | No | Other | ladderane | metatricycloene | 6 |
| JJ1805 | *Priestia megaterium* | no | No | PKS | T3PKS | unknown | ND |
| JJ1805 | *Priestia megaterium* | no | No | Other | phosphonate | unknown | ND |
| JJ1805 | *Priestia megaterium* | no | No | Terpene | terpene | unknown | ND |
| JJ1805 | *Priestia megaterium* | no | No | Other | siderophore | unknown | ND |
| JJ1805 | *Priestia megaterium* | no | No | Terpene | terpene | surfactin | 13 |
| JJ1806 | *Priestia megaterium* | no | No | Terpene | terpene | carotenoid | 50 |
| JJ1806 | *Priestia megaterium* | no | No | Other | phosphonate | unknown | ND |
| JJ1806 | *Priestia megaterium* | no | No | Terpene | terpene | unknown | ND |
| JJ1806 | *Priestia megaterium* | no | No | Other | siderophore | unknown | ND |
| JJ1806 | *Priestia megaterium* | no | No | PKS | T3PKS | unknown | ND |
| JJ1806 | *Priestia megaterium* | no | No | RiPP | lanthipeptide | unknown | ND |
| JJ1806 | *Priestia megaterium* | no | No | Terpene | terpene | surfactin | 13 |
| JJ1868 | *Bacillus safensis* | no | Strong | NRPS | NRPS | bacillibactin | 53 |
| JJ1868 | *Bacillus safensis* | no | Strong | Other | other | bacilysin | 71 |
| JJ1868 | *Bacillus safensis* | no | Strong | Other | other | carotenoid | 50 |
| JJ1868 | *Bacillus safensis* | no | Strong | Other | betalactone | fengycin | 53 |
| JJ1868 | *Bacillus safensis* | no | Strong | NRPS | NRPS | lichenysin | 50 |
| JJ1868 | *Bacillus safensis* | no | Strong | Other | betalactone | unknown | ND |
| JJ1868 | *Bacillus safensis* | no | Strong | Terpene | terpene | unknown | ND |
| JJ1868 | *Bacillus safensis* | no | Strong | PKS | T3PKS | unknown | ND |
| JJ1868 | *Bacillus safensis* | no | Strong | RiPP | bacteriocin | unknown | ND |
| JJ1868 | *Bacillus safensis* | no | Strong | RiPP | LAP | plantazolicin | 50 |
| JJ1868 | *Bacillus safensis* | no | Strong | Other | ladderane | S-layerglycan | 20 |
| JJ1868 | *Bacillus safensis* | no | Strong | RiPP | sactipeptide | sporulation killing factor | 85 |
| JJ1873 | *Bacillus toyonensis* | no | Sparse | NRPS | NRPS | bacillibactin | 38 |
| JJ1873 | *Bacillus toyonensis* | no | Sparse | RiPP | lanthipeptide | cerecidin | 70 |
| JJ1873 | *Bacillus toyonensis* | no | Sparse | RiPP | NRPS | micropeptin | 25 |
| JJ1873 | *Bacillus toyonensis* | no | Sparse | RiPP | LAP | unknown | ND |
| JJ1873 | *Bacillus toyonensis* | no | Sparse | NRPS | NRPS | unknown | ND |
| JJ1873 | *Bacillus toyonensis* | no | Sparse | Terpene | terpene | unknown | ND |
| JJ1873 | *Bacillus toyonensis* | no | Sparse | Other | betalactone | unknown | ND |
| JJ206 | *Bacillus safensis* | strong | Strong | NRPS | NRPS | bacillibactin | 53 |
| JJ206 | *Bacillus safensis* | strong | Strong | Other | other | bacilysin | 85 |
| JJ206 | *Bacillus safensis* | strong | Strong | Terpene | terpene | carotenoid | 50 |
| JJ206 | *Bacillus safensis* | strong | Strong | Other | betalactone | fengycin | 53 |
| JJ206 | *Bacillus safensis* | strong | Strong | NRPS | NRPS | lichenysin | 42 |
| JJ206 | *Bacillus safensis* | strong | Strong | PKS | T3PKS | unknown | ND |
| JJ206 | *Bacillus safensis* | strong | Strong | Other | betalactone | unknown | ND |
| JJ206 | *Bacillus safensis* | strong | Strong | Terpene | terpene | unknown | ND |
| JJ206 | *Bacillus safensis* | strong | Strong | RiPP | bacteriocin | unknown | ND |
| JJ208 | *Neobacillus drentensis* | no | No | RiPP | LAP | unknown | ND |
| JJ208 | *Neobacillus drentensis* | no | No | RiPP | bacteriocin | unknown | ND |
| JJ208 | *Neobacillus drentensis* | no | No | PKS | T3PKS | unknown | ND |
| JJ208 | *Neobacillus drentensis* | no | No | Terpene | terpene | unknown | ND |
| JJ208 | *Neobacillus drentensis* | no | No | Terpene | terpene | unknown | ND |
| JJ211 | *Neobacillus niacini* | no | No | PKS | T3PKS | unknown | ND |
| JJ211 | *Neobacillus niacini* | no | No | Other | betalactone | unknown | ND |
| JJ211 | *Neobacillus niacini* | no | No | Other | betalactone | unknown | ND |
| JJ211 | *Neobacillus niacini* | no | No | RiPP | LAP | unknown | ND |
| JJ211 | *Neobacillus niacini* | no | No | Terpene | terpene | unknown | ND |
| JJ211 | *Neobacillus niacini* | no | No | RiPP | lassopeptide | paeninodin | 100 |
| JJ213 | *Bacillus velezensis* | strong | Strong | NRPS | NRPS | surfactin | 91 |
| JJ213 | *Bacillus velezensis* | strong | Strong | Other | ladderane | unknown | ND |
| JJ213 | *Bacillus velezensis* | strong | Strong | PKS | PKS-like | butirosin A | 7 |
| JJ213 | *Bacillus velezensis* | strong | Strong | Terpene | terpene | unknown | ND |
| JJ213 | *Bacillus velezensis* | strong | Strong | RiPP | lanthipeptide-class-ii | unknown | ND |
| JJ213 | *Bacillus velezensis* | strong | Strong | PKS | transAT-PKS | macrolactin H | 100 |
| JJ213 | *Bacillus velezensis* | strong | Strong | PKS-NRPS | transAT-PKS | bacillaene | 100 |
| JJ213 | *Bacillus velezensis* | strong | Strong | NRPS | NRPS | fengycin | 100 |
| JJ213 | *Bacillus velezensis* | strong | Strong | Terpene | terpene | unknown | ND |
| JJ213 | *Bacillus velezensis* | strong | Strong | PKS | T3PKS | unknown | ND |
| JJ213 | *Bacillus velezensis* | strong | Strong | PKS | transAT-PKS | difficidin | 100 |
| JJ213 | *Bacillus velezensis* | strong | Strong | NRPS | NRPS | bacillibactin | 100 |
| JJ213 | *Bacillus velezensis* | strong | Strong | Other | other | bacilysin | 100 |
| JJ213 | *Bacillus velezensis* | strong | Strong | NRPS | Others | iturin/Bacilomycin L | 100 |
| JJ218 | *Bacillus thuringiensis* | no | Sparse | NRPS | NRPS | unknown | ND |
| JJ218 | *Bacillus thuringiensis* | no | Sparse | Terpene | terpene | molybdenum cofactor | 17 |
| JJ218 | *Bacillus thuringiensis* | no | Sparse | NRPS | NRPS | unknown | ND |
| JJ218 | *Bacillus thuringiensis* | no | Sparse | RiPP | RiPP-like | unknown | ND |
| JJ218 | *Bacillus thuringiensis* | no | Sparse | RiPP | RiPP-like | unknown | ND |
| JJ218 | *Bacillus thuringiensis* | no | Sparse | Other | betalactone | fengycin | 40 |
| JJ218 | *Bacillus thuringiensis* | no | Sparse | NRPS | NRPS | unknown | ND |
| JJ218 | *Bacillus thuringiensis* | no | Sparse | NRPS | NRPS | bacillibactin | 46 |
| JJ218 | *Bacillus thuringiensis* | no | Sparse | RiPP | lanthipeptide-class-iii | unknown | ND |
| JJ218 | *Bacillus thuringiensis* | no | Sparse | Other | siderophore | petrobactin | 100 |
| JJ218 | *Bacillus thuringiensis* | no | Sparse | RiPP | LAP | unknown | ND |
| JJ218 | *Bacillus thuringiensis* | no | Sparse | NRPS | NRPS-like | unknown | ND |
| JJ218 | *Bacillus thuringiensis* | no | Sparse | Other | furan | methylenomycin A | 14 |
| JJ218 | *Bacillus thuringiensis* | no | Sparse | PKS-NRPS | NRPS | zwittermicin A | 81 |
| JJ258 | *Bacillus velezensis* | weak | Strong | PKS | transAT-PKS-like | bacillaene | 14 |
| JJ258 | *Bacillus velezensis* | weak | Strong | NRPS | NRPS | bacillibactin | 38 |
| JJ258 | *Bacillus velezensis* | weak | Strong | Other | other | bacilysin | 85 |
| JJ258 | *Bacillus velezensis* | weak | Strong | PKS | transAT-PKS-like | difficidin | 26 |
| JJ258 | *Bacillus velezensis* | weak | Strong | NRPS | NRPS | fengycin | 33 |
| JJ258 | *Bacillus velezensis* | weak | Strong | PKS | transAT-PKS-like | macrolactin H | 44 |
| JJ258 | *Bacillus velezensis* | weak | Strong | NRPS | NRPS | unknown | ND |
| JJ258 | *Bacillus velezensis* | weak | Strong | PKS | transAT-PKS-like | unknown | ND |
| JJ258 | *Bacillus velezensis* | weak | Strong | Terpene | terpene | unknown | ND |
| JJ258 | *Bacillus velezensis* | weak | Strong | PKS | transAT-PKS-like | unknown | ND |
| JJ258 | *Bacillus velezensis* | weak | Strong | NRPS | NRPS | unknown | ND |
| JJ258 | *Bacillus velezensis* | weak | Strong | NRPS | NRPS | paenilipoheptin | 7 |
| JJ258 | *Bacillus velezensis* | weak | Strong | NRPS | NRPS | surfactin | 47 |
| JJ258 | *Bacillus velezensis* | weak | Strong | NRPS | NRPS | iturin/Bacilomycin L | 33 |
| JJ261 | *Bacillus velezensis* | no | Strong | NRPS | NRPS | bacillibactin | 100 |
| JJ261 | *Bacillus velezensis* | no | Strong | PKS | PKS-like | butirosin A | 7 |
| JJ261 | *Bacillus velezensis* | no | Strong | NRPS | NRPS | fengycin | 20 |
| JJ261 | *Bacillus velezensis* | no | Strong | PKS | transAT-PKS | macrolactin H | 30 |
| JJ261 | *Bacillus velezensis* | no | Strong | PKS | transAT-PKS-like | unknown | ND |
| JJ261 | *Bacillus velezensis* | no | Strong | NRPS | NRPS | unknown | ND |
| JJ261 | *Bacillus velezensis* | no | Strong | Other | betalactone | unknown | ND |
| JJ261 | *Bacillus velezensis* | no | Strong | Terpene | terpene | unknown | ND |
| JJ261 | *Bacillus velezensis* | no | Strong | Other | other | rhizocticin A | 12 |
| JJ261 | *Bacillus velezensis* | no | Strong | NRPS | NRPS | surfactin | 8 |
| JJ261 | *Bacillus velezensis* | no | Strong | NRPS | NRPS | iturin/Bacilomycin L | 20 |
| JJ266 | *Priestia megaterium* | no | No | Terpene | terpene | carotenoid | 50 |
| JJ266 | *Priestia megaterium* | no | No | PKS | PKS-like | macrobrevin | 53 |
| JJ266 | *Priestia megaterium* | no | No | PKS | T3PKS | unknown | ND |
| JJ266 | *Priestia megaterium* | no | No | Terpene | terpene | unknown | ND |
| JJ266 | *Priestia megaterium* | no | No | Other | siderophore | unknown | ND |
| JJ266 | *Priestia megaterium* | no | No | Other | phosphonate | unknown | ND |
| JJ266 | *Priestia megaterium* | no | No | RiPP | bacteriocin | unknown | ND |
| JJ266 | *Priestia megaterium* | no | No | PKS | transAT-PKS-like | scytophycin | 27 |
| JJ266 | *Priestia megaterium* | no | No | Terpene | terpene | surfactin | 13 |
| JJ269 | *Bacillus (other)* | no | No | NRPS | NRPS | bacillibactin | 46 |
| JJ269 | *Bacillus (other)* | no | No | RiPP | lanthipeptide | cerecidin | 58 |
| JJ269 | *Bacillus (other)* | no | No | Other | betalactone | fengycin | 40 |
| JJ269 | *Bacillus (other)* | no | No | Terpene | terpene | molybdenum cofactor | 17 |
| JJ269 | *Bacillus (other)* | no | No | NRPS | NRPS | unknown | ND |
| JJ269 | *Bacillus (other)* | no | No | RiPP | LAP | unknown | ND |
| JJ269 | *Bacillus (other)* | no | No | RiPP | bacteriocin | unknown | ND |
| JJ269 | *Bacillus (other)* | no | No | Other | ladderane | unknown | ND |
| JJ269 | *Bacillus (other)* | no | No | Other | siderophore | petrobactin | 100 |
| JJ269 | *Bacillus (other)* | no | No | NRPS | NRPS | polyoxypeptin | 5 |
| JJ271 | *Bacillus altitudinis* | strong | Sparse | NRPS | NRPS | bacillibactin | 53 |
| JJ271 | *Bacillus altitudinis* | strong | Sparse | Other | other | carotenoid | 50 |
| JJ271 | *Bacillus altitudinis* | strong | Sparse | Other | betalactone | fengycin | 53 |
| JJ271 | *Bacillus altitudinis* | strong | Sparse | NRPS | NRPS | lichenysin | 42 |
| JJ271 | *Bacillus altitudinis* | strong | Sparse | Other | betalactone | unknown | ND |
| JJ271 | *Bacillus altitudinis* | strong | Sparse | Terpene | terpene | unknown | ND |
| JJ271 | *Bacillus altitudinis* | strong | Sparse | PKS | T3PKS | unknown | ND |
| JJ271 | *Bacillus altitudinis* | strong | Sparse | NRPS | NRPS-like | unknown | ND |
| JJ271 | *Bacillus altitudinis* | strong | Sparse | NRPS | NRPS | unknown | ND |
| JJ274 | *Bacillus (other)* | no | No | Terpene | terpene | carotenoid | 50 |
| JJ274 | *Bacillus (other)* | no | No | Other | phosphonate | unknown | ND |
| JJ274 | *Bacillus (other)* | no | No | PKS | T3PKS | unknown | ND |
| JJ274 | *Bacillus (other)* | no | No | Terpene | terpene | unknown | ND |
| JJ274 | *Bacillus (other)* | no | No | Other | siderophore | unknown | ND |
| JJ274 | *Bacillus (other)* | no | No | RiPP | lanthipeptide | unknown | ND |
| JJ274 | *Bacillus (other)* | no | No | Terpene | terpene | surfactin | 13 |
| JJ279 | *Bacillus pumilus* | weak | Strong | NRPS | NRPS | bacillibactin | 53 |
| JJ279 | *Bacillus pumilus* | weak | Strong | Other | other | bacilysin | 85 |
| JJ279 | *Bacillus pumilus* | weak | Strong | Other | other | carotenoid | 50 |
| JJ279 | *Bacillus pumilus* | weak | Strong | Other | betalactone | fengycin | 53 |
| JJ279 | *Bacillus pumilus* | weak | Strong | NRPS | NRPS | lichenysin | 50 |
| JJ279 | *Bacillus pumilus* | weak | Strong | PKS | T3PKS | unknown | ND |
| JJ279 | *Bacillus pumilus* | weak | Strong | Other | betalactone | unknown | ND |
| JJ279 | *Bacillus pumilus* | weak | Strong | Terpene | terpene | unknown | ND |
| JJ279 | *Bacillus pumilus* | weak | Strong | RiPP | bacteriocin | unknown | ND |
| JJ279 | *Bacillus pumilus* | weak | Strong | PKS-NRPS | NRPS | zwittermicin A | 18 |
| JJ32 | *Bacillus velezensis* | weak | Strong | PKS-NRPS | transAT-PKS | bacillaene | 78 |
| JJ32 | *Bacillus velezensis* | weak | Strong | NRPS | NRPS | bacillibactin | 38 |
| JJ32 | *Bacillus velezensis* | weak | Strong | Other | other | bacilysin | 100 |
| JJ32 | *Bacillus velezensis* | weak | Strong | PKS | PKS-like | butirosin A | 7 |
| JJ32 | *Bacillus velezensis* | weak | Strong | PKS | transAT-PKS-like | difficidin | 53 |
| JJ32 | *Bacillus velezensis* | weak | Strong | NRPS | NRPS | fengycin | 20 |
| JJ32 | *Bacillus velezensis* | weak | Strong | PKS | transAT-PKS | macrolactin H | 100 |
| JJ32 | *Bacillus velezensis* | weak | Strong | Other | ladderane | unknown | ND |
| JJ32 | *Bacillus velezensis* | weak | Strong | PKS | T3PKS | unknown | ND |
| JJ32 | *Bacillus velezensis* | weak | Strong | Terpene | terpene | unknown | ND |
| JJ32 | *Bacillus velezensis* | weak | Strong | Terpene | terpene | unknown | ND |
| JJ32 | *Bacillus velezensis* | weak | Strong | NRPS | NRPS | surfactin | 91 |
| JJ32 | *Bacillus velezensis* | weak | Strong | NRPS | NRPS | iturin/Bacilomycin L | 20 |
| JJ326 | *Bacillus safensis* | weak | Strong | NRPS | NRPS | bacillibactin | 53 |
| JJ326 | *Bacillus safensis* | weak | Strong | Other | other | carotenoid | 50 |
| JJ326 | *Bacillus safensis* | weak | Strong | Other | betalactone | fengycin | 53 |
| JJ326 | *Bacillus safensis* | weak | Strong | NRPS | NRPS | lichenysin | 28 |
| JJ326 | *Bacillus safensis* | weak | Strong | Other | betalactone | unknown | ND |
| JJ326 | *Bacillus safensis* | weak | Strong | Terpene | terpene | unknown | ND |
| JJ326 | *Bacillus safensis* | weak | Strong | PKS | T3PKS | unknown | ND |
| JJ327 | *Priestia megaterium* | no | No | Terpene | terpene | carotenoid | 50 |
| JJ327 | *Priestia megaterium* | no | No | PKS | T3PKS | unknown | ND |
| JJ327 | *Priestia megaterium* | no | No | Other | phosphonate | unknown | ND |
| JJ327 | *Priestia megaterium* | no | No | Terpene | terpene | unknown | ND |
| JJ327 | *Priestia megaterium* | no | No | Other | siderophore | unknown | ND |
| JJ327 | *Priestia megaterium* | no | No | Terpene | terpene | surfactin | 13 |
| JJ330 | *Bacillus velezensis* | no | Strong | PKS-NRPS | transAT-PKS-like | bacillaene | 100 |
| JJ330 | *Bacillus velezensis* | no | Strong | NRPS | bacteriocin | bacillibactin | 100 |
| JJ330 | *Bacillus velezensis* | no | Strong | Other | other | bacilysin | 100 |
| JJ330 | *Bacillus velezensis* | no | Strong | PKS | PKS-like | butirosin A | 7 |
| JJ330 | *Bacillus velezensis* | no | Strong | PKS | transAT-PKS-like | difficidin | 53 |
| JJ330 | *Bacillus velezensis* | no | Strong | NRPS | NRPS | fengycin | 86 |
| JJ330 | *Bacillus velezensis* | no | Strong | PKS | transAT-PKS | macrolactin H | 100 |
| JJ330 | *Bacillus velezensis* | no | Strong | RiPP | lanthipeptide | mersacidin | 100 |
| JJ330 | *Bacillus velezensis* | no | Strong | PKS | T3PKS | unknown | ND |
| JJ330 | *Bacillus velezensis* | no | Strong | Terpene | terpene | unknown | ND |
| JJ330 | *Bacillus velezensis* | no | Strong | Terpene | terpene | unknown | ND |
| JJ330 | *Bacillus velezensis* | no | Strong | NRPS | NRPS | unknown | ND |
| JJ330 | *Bacillus velezensis* | no | Strong | NRPS | NRPS | surfactin | 47 |
| JJ330 | *Bacillus velezensis* | no | Strong | NRPS | Others | iturin/Bacilomycin L | 86 |
| JJ334 | *Bacillus velezensis* | strong | Strong | NRPS | NRPS | surfactin | 91 |
| JJ334 | *Bacillus velezensis* | strong | Strong | RiPP | LAP | plantazolicin | 91 |
| JJ334 | *Bacillus velezensis* | strong | Strong | PKS | PKS-like | butirosin A | 7 |
| JJ334 | *Bacillus velezensis* | strong | Strong | Terpene | terpene | unknown | ND |
| JJ334 | *Bacillus velezensis* | strong | Strong | PKS | transAT-PKS | macrolactin H | 100 |
| JJ334 | *Bacillus velezensis* | strong | Strong | PKS-NRPS | transAT-PKS | bacillaene | 100 |
| JJ334 | *Bacillus velezensis* | strong | Strong | NRPS | NRPS | fengycin | 100 |
| JJ334 | *Bacillus velezensis* | strong | Strong | Terpene | terpene | unknown | ND |
| JJ334 | *Bacillus velezensis* | strong | Strong | PKS | T3PKS | unknown | ND |
| JJ334 | *Bacillus velezensis* | strong | Strong | PKS | transAT-PKS | difficidin | 100 |
| JJ334 | *Bacillus velezensis* | strong | Strong | NRPS | NRPS | unknown | ND |
| JJ334 | *Bacillus velezensis* | strong | Strong | NRPS | NRPS | bacillibactin | 100 |
| JJ334 | *Bacillus velezensis* | strong | Strong | Other | other | bacilysin | 100 |
| JJ334 | *Bacillus velezensis* | strong | Strong | NRPS | Others | iturin/Bacilomycin L | 100 |
| JJ353 | *Bacillus (other)* | no | No | NRPS | NRPS | bacillibactin | 53 |
| JJ353 | *Bacillus (other)* | no | No | NRPS | NRPS | bacitracin | 33 |
| JJ353 | *Bacillus (other)* | no | No | Other | betalactone | fengycin | 46 |
| JJ353 | *Bacillus (other)* | no | No | NRPS | NRPS | lichenysin | 57 |
| JJ353 | *Bacillus (other)* | no | No | Other | siderophore | unknown | ND |
| JJ353 | *Bacillus (other)* | no | No | RiPP | bacteriocin | unknown | ND |
| JJ353 | *Bacillus (other)* | no | No | NRPS | NRPS | unknown | ND |
| JJ353 | *Bacillus (other)* | no | No | PKS | T3PKS | unknown | ND |
| JJ353 | *Bacillus (other)* | no | No | Terpene | terpene | unknown | ND |
| JJ356 | *Neobacillus drentensis* | weak | No | PKS | T3PKS | unknown | ND |
| JJ356 | *Neobacillus drentensis* | weak | No | Terpene | terpene | unknown | ND |
| JJ356 | *Neobacillus drentensis* | weak | No | RiPP | bacteriocin | unknown | ND |
| JJ356 | *Neobacillus drentensis* | weak | No | RiPP | LAP | unknown | ND |
| JJ36 | *Bacillus safensis* | weak | Strong | NRPS | NRPS | bacillibactin | 53 |
| JJ36 | *Bacillus safensis* | weak | Strong | Other | other | bacilysin | 85 |
| JJ36 | *Bacillus safensis* | weak | Strong | Terpene | terpene | carotenoid | 50 |
| JJ36 | *Bacillus safensis* | weak | Strong | Other | betalactone | fengycin | 53 |
| JJ36 | *Bacillus safensis* | weak | Strong | NRPS | NRPS | lichenysin | 50 |
| JJ36 | *Bacillus safensis* | weak | Strong | PKS | T3PKS | unknown | ND |
| JJ36 | *Bacillus safensis* | weak | Strong | Other | betalactone | unknown | ND |
| JJ36 | *Bacillus safensis* | weak | Strong | Terpene | terpene | unknown | ND |
| JJ36 | *Bacillus safensis* | weak | Strong | RiPP | bacteriocin | unknown | ND |
| JJ36 | *Bacillus safensis* | weak | Strong | RiPP | LAP | plantazolicin | 91 |
| JJ36 | *Bacillus safensis* | weak | Strong | Other | ladderane | S-layerglycan | 20 |
| JJ36 | *Bacillus safensis* | weak | Strong | RiPP | sactipeptide | sporulation killing factor | 85 |
| JJ37 | *Priestia megaterium* | no | No | Terpene | terpene | carotenoid | 50 |
| JJ37 | *Priestia megaterium* | no | No | Terpene | terpene | locillomycin | 14 |
| JJ37 | *Priestia megaterium* | no | No | PKS | T3PKS | unknown | ND |
| JJ37 | *Priestia megaterium* | no | No | Other | phosphonate | unknown | ND |
| JJ37 | *Priestia megaterium* | no | No | Terpene | terpene | unknown | ND |
| JJ37 | *Priestia megaterium* | no | No | Other | siderophore | unknown | ND |
| JJ370 | *Neobacillus vireti* | no | No | PKS | T3PKS | unknown | ND |
| JJ370 | *Neobacillus vireti* | no | No | RiPP | LAP | unknown | ND |
| JJ370 | *Neobacillus vireti* | no | No | Terpene | terpene | unknown | ND |
| JJ38 | *Bacillus velezensis* | no | Strong | RiPP | bacteriocin | amylocyclicin | 100 |
| JJ38 | *Bacillus velezensis* | no | Strong | PKS-NRPS | transAT-PKS-like | bacillaene | 35 |
| JJ38 | *Bacillus velezensis* | no | Strong | NRPS | NRPS | bacillibactin | 38 |
| JJ38 | *Bacillus velezensis* | no | Strong | Other | other | bacilysin | 100 |
| JJ38 | *Bacillus velezensis* | no | Strong | PKS | transAT-PKS | difficidin | 46 |
| JJ38 | *Bacillus velezensis* | no | Strong | Other | betalactone | fengycin | 46 |
| JJ38 | *Bacillus velezensis* | no | Strong | NRPS | transAT-PKS-like | locillomycin | 50 |
| JJ38 | *Bacillus velezensis* | no | Strong | PKS | transAT-PKS | macrolactin H | 80 |
| JJ38 | *Bacillus velezensis* | no | Strong | PKS | T3PKS | unknown | ND |
| JJ38 | *Bacillus velezensis* | no | Strong | Terpene | terpene | unknown | ND |
| JJ38 | *Bacillus velezensis* | no | Strong | Terpene | terpene | unknown | ND |
| JJ38 | *Bacillus velezensis* | no | Strong | NRPS | NRPS | unknown | ND |
| JJ38 | *Bacillus velezensis* | no | Strong | NRPS | NRPS | surfactin | 65 |
| JJ387 | *Neobacillus drentensis* | no | No | PKS | T3PKS | unknown | ND |
| JJ387 | *Neobacillus drentensis* | no | No | Terpene | terpene | unknown | ND |
| JJ387 | *Neobacillus drentensis* | no | No | RiPP | bacteriocin | unknown | ND |
| JJ387 | *Neobacillus drentensis* | no | No | RiPP | lassopeptide | paeninodin | 80 |
| JJ399 | *Bacillus altitudinis* | weak | Sparse | NRPS | NRPS | bacillibactin | 53 |
| JJ399 | *Bacillus altitudinis* | weak | Sparse | Other | other | bacilysin | 85 |
| JJ399 | *Bacillus altitudinis* | weak | Sparse | Other | other | carotenoid | 50 |
| JJ399 | *Bacillus altitudinis* | weak | Sparse | Other | betalactone | fengycin | 53 |
| JJ399 | *Bacillus altitudinis* | weak | Sparse | NRPS | NRPS | lichenysin | 50 |
| JJ399 | *Bacillus altitudinis* | weak | Sparse | PKS | T3PKS | unknown | ND |
| JJ399 | *Bacillus altitudinis* | weak | Sparse | Other | betalactone | unknown | ND |
| JJ399 | *Bacillus altitudinis* | weak | Sparse | Terpene | terpene | unknown | ND |
| JJ399 | *Bacillus altitudinis* | weak | Sparse | RiPP | bacteriocin | unknown | ND |
| JJ4 | *Bacillus safensis* | no | Strong | NRPS | NRPS | bacillibactin | 53 |
| JJ4 | *Bacillus safensis* | no | Strong | Other | other | carotenoid | 50 |
| JJ4 | *Bacillus safensis* | no | Strong | Other | betalactone | fengycin | 53 |
| JJ4 | *Bacillus safensis* | no | Strong | NRPS | NRPS | lichenysin | 71 |
| JJ4 | *Bacillus safensis* | no | Strong | PKS | T3PKS | unknown | ND |
| JJ4 | *Bacillus safensis* | no | Strong | Other | betalactone | unknown | ND |
| JJ4 | *Bacillus safensis* | no | Strong | Terpene | terpene | unknown | ND |
| JJ4 | *Bacillus safensis* | no | Strong | RiPP | bacteriocin | unknown | ND |
| JJ4 | *Bacillus safensis* | no | Strong | RiPP | LAP | plantazolicin | 50 |
| JJ405 | *Neobacillus vireti* | no | No | RiPP | thiopeptide | lankacidin C | 13 |
| JJ405 | *Neobacillus vireti* | no | No | PKS | T3PKS | unknown | ND |
| JJ405 | *Neobacillus vireti* | no | No | Terpene | terpene | unknown | ND |
| JJ405 | *Neobacillus vireti* | no | No | Terpene | terpene | unknown | ND |
| JJ405 | *Neobacillus vireti* | no | No | RiPP | lassopeptide | paeninodin | 80 |
| JJ41 | *Bacillus toyonensis* | weak | Sparse | NRPS | NRPS | bacillibactin | 46 |
| JJ41 | *Bacillus toyonensis* | weak | Sparse | Other | betalactone | fengycin | 40 |
| JJ41 | *Bacillus toyonensis* | weak | Sparse | Terpene | terpene | molybdenum cofactor | 17 |
| JJ41 | *Bacillus toyonensis* | weak | Sparse | NRPS | NRPS | unknown | ND |
| JJ41 | *Bacillus toyonensis* | weak | Sparse | NRPS | NRPS-like | unknown | ND |
| JJ41 | *Bacillus toyonensis* | weak | Sparse | NRPS | NRPS | unknown | ND |
| JJ41 | *Bacillus toyonensis* | weak | Sparse | RiPP | LAP | unknown | ND |
| JJ41 | *Bacillus toyonensis* | weak | Sparse | RiPP | bacteriocin | unknown | ND |
| JJ41 | *Bacillus toyonensis* | weak | Sparse | RiPP | lassopeptide | paeninodin | 80 |
| JJ41 | *Bacillus toyonensis* | weak | Sparse | Other | siderophore | petrobactin | 100 |
| JJ43 | *Bacillus pumilus* | strong | Strong | NRPS | NRPS | bacillibactin | 53 |
| JJ43 | *Bacillus pumilus* | strong | Strong | Other | other | bacilysin | 85 |
| JJ43 | *Bacillus pumilus* | strong | Strong | Other | other | carotenoid | 50 |
| JJ43 | *Bacillus pumilus* | strong | Strong | Other | betalactone | fengycin | 53 |
| JJ43 | *Bacillus pumilus* | strong | Strong | NRPS | NRPS | lichenysin | 85 |
| JJ43 | *Bacillus pumilus* | strong | Strong | Other | betalactone | unknown | ND |
| JJ43 | *Bacillus pumilus* | strong | Strong | PKS | T3PKS | unknown | ND |
| JJ43 | *Bacillus pumilus* | strong | Strong | Terpene | terpene | unknown | ND |
| JJ43 | *Bacillus pumilus* | strong | Strong | RiPP | head_to_tail | sporulation killing factor | 85 |
| JJ43 | *Bacillus pumilus* | strong | Strong | PKS-NRPS | NRPS | zwittermicin A | 18 |
| JJ457 | *Priestia megaterium* | no | No | Terpene | terpene | carotenoid | 50 |
| JJ457 | *Priestia megaterium* | no | No | PKS | T3PKS | unknown | ND |
| JJ457 | *Priestia megaterium* | no | No | Terpene | terpene | unknown | ND |
| JJ457 | *Priestia megaterium* | no | No | Other | siderophore | unknown | ND |
| JJ457 | *Priestia megaterium* | no | No | Other | siderophore | unknown | ND |
| JJ457 | *Priestia megaterium* | no | No | RiPP | lassopeptide | paeninodin | 60 |
| JJ457 | *Priestia megaterium* | no | No | Terpene | terpene | surfactin | 13 |
| JJ462 | *Neobacillus vireti* | no | No | PKS | T3PKS | unknown | ND |
| JJ462 | *Neobacillus vireti* | no | No | Terpene | terpene | unknown | ND |
| JJ462 | *Neobacillus vireti* | no | No | RiPP | bacteriocin | unknown | ND |
| JJ462 | *Neobacillus vireti* | no | No | Terpene | terpene | unknown | ND |
| JJ462 | *Neobacillus vireti* | no | No | RiPP | LAP | unknown | ND |
| JJ466 | *Bacillus subtilis* | no | Strong | PKS-NRPS | transAT-PKS | bacillaene | 100 |
| JJ466 | *Bacillus subtilis* | no | Strong | NRPS | NRPS | bacillibactin | 100 |
| JJ466 | *Bacillus subtilis* | no | Strong | Other | other | bacilysin | 100 |
| JJ466 | *Bacillus subtilis* | no | Strong | NRPS | NRPS | fengycin | 86 |
| JJ466 | *Bacillus subtilis* | no | Strong | PKS | T3PKS | unknown | ND |
| JJ466 | *Bacillus subtilis* | no | Strong | Terpene | terpene | unknown | ND |
| JJ466 | *Bacillus subtilis* | no | Strong | Terpene | terpene | unknown | ND |
| JJ466 | *Bacillus subtilis* | no | Strong | RiPP | lanthipeptide | subtilomycin | 100 |
| JJ466 | *Bacillus subtilis* | no | Strong | RiPP | head_to_tail | subtilosin A | 100 |
| JJ466 | *Bacillus subtilis* | no | Strong | NRPS | NRPS | surfactin | 47 |
| JJ479 | *Neobacillus vireti* | no | No | PKS | T3PKS | unknown | ND |
| JJ479 | *Neobacillus vireti* | no | No | RiPP | LAP | unknown | ND |
| JJ479 | *Neobacillus vireti* | no | No | Terpene | terpene | unknown | ND |
| JJ479 | *Neobacillus vireti* | no | No | Terpene | terpene | unknown | ND |
| JJ479 | *Neobacillus vireti* | no | No | RiPP | bacteriocin | unknown | ND |
| JJ479 | *Neobacillus vireti* | no | No | RiPP | lassopeptide | paeninodin | 100 |
| JJ480 | *Bacillus toyonensis* | no | Sparse | NRPS | NRPS | bacillibactin | 46 |
| JJ480 | *Bacillus toyonensis* | no | Sparse | RiPP | bacteriocin | bacillicin | 15 |
| JJ480 | *Bacillus toyonensis* | no | Sparse | Other | betalactone | fengycin | 40 |
| JJ480 | *Bacillus toyonensis* | no | Sparse | NRPS | NRPS | mannopeptimycin | 7 |
| JJ480 | *Bacillus toyonensis* | no | Sparse | Terpene | terpene | molybdenum cofactor | 17 |
| JJ480 | *Bacillus toyonensis* | no | Sparse | NRPS | NRPS-like | unknown | ND |
| JJ480 | *Bacillus toyonensis* | no | Sparse | RiPP | LAP | unknown | ND |
| JJ480 | *Bacillus toyonensis* | no | Sparse | NRPS | NRPS | unknown | ND |
| JJ480 | *Bacillus toyonensis* | no | Sparse | NRPS | NRPS | unknown | ND |
| JJ480 | *Bacillus toyonensis* | no | Sparse | RiPP | bacteriocin | unknown | ND |
| JJ480 | *Bacillus toyonensis* | no | Sparse | RiPP | lassopeptide | paeninodin | 80 |
| JJ480 | *Bacillus toyonensis* | no | Sparse | Other | siderophore | petrobactin | 100 |
| JJ480 | *Bacillus toyonensis* | no | Sparse | Other | ladderane | S-layerglycan | 26 |
| JJ495 | *Bacillus pumilus* | weak | Strong | NRPS | NRPS | bacillibactin | 53 |
| JJ495 | *Bacillus pumilus* | weak | Strong | Other | other | bacilysin | 85 |
| JJ495 | *Bacillus pumilus* | weak | Strong | Other | other | carotenoid | 50 |
| JJ495 | *Bacillus pumilus* | weak | Strong | Other | betalactone | fengycin | 53 |
| JJ495 | *Bacillus pumilus* | weak | Strong | NRPS | NRPS | lichenysin | 50 |
| JJ495 | *Bacillus pumilus* | weak | Strong | PKS | T3PKS | unknown | ND |
| JJ495 | *Bacillus pumilus* | weak | Strong | Other | betalactone | unknown | ND |
| JJ495 | *Bacillus pumilus* | weak | Strong | Terpene | terpene | unknown | ND |
| JJ495 | *Bacillus pumilus* | weak | Strong | PKS-NRPS | NRPS | zwittermicin A | 18 |
| JJ499 | *Bacillus thuringiensis* | no | Sparse | NRPS | NRPS | bacillibactin | 46 |
| JJ499 | *Bacillus thuringiensis* | no | Sparse | Other | betalactone | fengycin | 40 |
| JJ499 | *Bacillus thuringiensis* | no | Sparse | Terpene | terpene | molybdenum cofactor | 11 |
| JJ499 | *Bacillus thuringiensis* | no | Sparse | NRPS | NRPS-like | unknown | ND |
| JJ499 | *Bacillus thuringiensis* | no | Sparse | NRPS | NRPS | unknown | ND |
| JJ499 | *Bacillus thuringiensis* | no | Sparse | RiPP | LAP | unknown | ND |
| JJ499 | *Bacillus thuringiensis* | no | Sparse | RiPP | bacteriocin | unknown | ND |
| JJ499 | *Bacillus thuringiensis* | no | Sparse | Other | siderophore | petrobactin | 100 |
| JJ511 | *Bacillus safensis* | strong | Strong | NRPS | NRPS | bacillibactin | 53 |
| JJ511 | *Bacillus safensis* | strong | Strong | Other | other | bacilysin | 85 |
| JJ511 | *Bacillus safensis* | strong | Strong | Terpene | terpene | carotenoid | 50 |
| JJ511 | *Bacillus safensis* | strong | Strong | Other | betalactone | fengycin | 53 |
| JJ511 | *Bacillus safensis* | strong | Strong | NRPS | NRPS | lichenysin | 71 |
| JJ511 | *Bacillus safensis* | strong | Strong | PKS | T3PKS | unknown | ND |
| JJ511 | *Bacillus safensis* | strong | Strong | Other | betalactone | unknown | ND |
| JJ511 | *Bacillus safensis* | strong | Strong | Terpene | terpene | unknown | ND |
| JJ511 | *Bacillus safensis* | strong | Strong | RiPP | bacteriocin | unknown | ND |
| JJ511 | *Bacillus safensis* | strong | Strong | RiPP | LAP | plantazolicin | 91 |
| JJ519 | *Bacillus safensis* | strong | Strong | NRPS | NRPS | bacillibactin | 53 |
| JJ519 | *Bacillus safensis* | strong | Strong | Other | other | bacilysin | 85 |
| JJ519 | *Bacillus safensis* | strong | Strong | Other | other | carotenoid | 50 |
| JJ519 | *Bacillus safensis* | strong | Strong | Other | betalactone | fengycin | 53 |
| JJ519 | *Bacillus safensis* | strong | Strong | NRPS | NRPS | lichenysin | 85 |
| JJ519 | *Bacillus safensis* | strong | Strong | PKS | T3PKS | unknown | ND |
| JJ519 | *Bacillus safensis* | strong | Strong | Terpene | terpene | unknown | ND |
| JJ519 | *Bacillus safensis* | strong | Strong | RiPP | bacteriocin | unknown | ND |
| JJ519 | *Bacillus safensis* | strong | Strong | RiPP | LAP | plantazolicin | 100 |
| JJ519 | *Bacillus safensis* | strong | Strong | RiPP | sactipeptide | sporulation killing factor | 85 |
| JJ523 | *Bacillus altitudinis* | strong | Sparse | NRPS | NRPS | bacillibactin | 53 |
| JJ523 | *Bacillus altitudinis* | strong | Sparse | Other | other | bacilysin | 85 |
| JJ523 | *Bacillus altitudinis* | strong | Sparse | Terpene | terpene | carotenoid | 50 |
| JJ523 | *Bacillus altitudinis* | strong | Sparse | Other | betalactone | fengycin | 53 |
| JJ523 | *Bacillus altitudinis* | strong | Sparse | NRPS | NRPS | lichenysin | 14 |
| JJ523 | *Bacillus altitudinis* | strong | Sparse | PKS | T3PKS | unknown | ND |
| JJ523 | *Bacillus altitudinis* | strong | Sparse | Other | betalactone | unknown | ND |
| JJ523 | *Bacillus altitudinis* | strong | Sparse | Terpene | terpene | unknown | ND |
| JJ523 | *Bacillus altitudinis* | strong | Sparse | NRPS | NRPS | unknown | ND |
| JJ523 | *Bacillus altitudinis* | strong | Sparse | RiPP | bacteriocin | unknown | ND |
| JJ523 | *Bacillus altitudinis* | strong | Sparse | NRPS | NRPS-like | sporulation killing factor | 57 |
| JJ524 | *Neobacillus drentensis* | no | No | Other | betalactone | fengycin | 40 |
| JJ524 | *Neobacillus drentensis* | no | No | PKS | T3PKS | unknown | ND |
| JJ524 | *Neobacillus drentensis* | no | No | PKS | T3PKS | unknown | ND |
| JJ524 | *Neobacillus drentensis* | no | No | Terpene | terpene | unknown | ND |
| JJ524 | *Neobacillus drentensis* | no | No | RiPP | bacteriocin | unknown | ND |
| JJ524 | *Neobacillus drentensis* | no | No | RiPP | lassopeptide | paeninodin | 80 |
| JJ535 | *Priestia megaterium* | no | No | Terpene | terpene | carotenoid | 50 |
| JJ535 | *Priestia megaterium* | no | No | RiPP | lanthipeptide | cytolysin | 60 |
| JJ535 | *Priestia megaterium* | no | No | Other | phosphonate | unknown | ND |
| JJ535 | *Priestia megaterium* | no | No | PKS | T3PKS | unknown | ND |
| JJ535 | *Priestia megaterium* | no | No | Terpene | terpene | unknown | ND |
| JJ535 | *Priestia megaterium* | no | No | Other | CDPS | unknown | ND |
| JJ535 | *Priestia megaterium* | no | No | Other | siderophore | unknown | ND |
| JJ535 | *Priestia megaterium* | no | No | Terpene | terpene | surfactin | 13 |
| JJ538 | *Bacillus toyonensis* | weak | Sparse | NRPS | NRPS | bacillibactin | 46 |
| JJ538 | *Bacillus toyonensis* | weak | Sparse | NRPS | NRPS | unknown | ND |
| JJ538 | *Bacillus toyonensis* | weak | Sparse | RiPP | LAP | unknown | ND |
| JJ538 | *Bacillus toyonensis* | weak | Sparse | Terpene | terpene | unknown | ND |
| JJ538 | *Bacillus toyonensis* | weak | Sparse | Other | betalactone | unknown | ND |
| JJ538 | *Bacillus toyonensis* | weak | Sparse | RiPP | bacteriocin | unknown | ND |
| JJ538 | *Bacillus toyonensis* | weak | Sparse | RiPP | lassopeptide | paeninodin | 80 |
| JJ542 | *Bacillus safensis* | strong | Strong | NRPS | NRPS | bacillibactin | 53 |
| JJ542 | *Bacillus safensis* | strong | Strong | Other | other | bacilysin | 85 |
| JJ542 | *Bacillus safensis* | strong | Strong | Other | betalactone | bottromycin A | 6 |
| JJ542 | *Bacillus safensis* | strong | Strong | Other | other | carotenoid | 50 |
| JJ542 | *Bacillus safensis* | strong | Strong | Other | betalactone | fengycin | 53 |
| JJ542 | *Bacillus safensis* | strong | Strong | NRPS | NRPS | lichenysin | 50 |
| JJ542 | *Bacillus safensis* | strong | Strong | PKS | T3PKS | unknown | ND |
| JJ542 | *Bacillus safensis* | strong | Strong | Terpene | terpene | unknown | ND |
| JJ542 | *Bacillus safensis* | strong | Strong | RiPP | bacteriocin | unknown | ND |
| JJ542 | *Bacillus safensis* | strong | Strong | RiPP | LAP | plantazolicin | 91 |
| JJ543 | *Bacillus toyonensis* | no | Sparse | NRPS | NRPS | aunknownbaenopeptin | 100 |
| JJ543 | *Bacillus toyonensis* | no | Sparse | NRPS | NRPS | bacillibactin | 46 |
| JJ543 | *Bacillus toyonensis* | no | Sparse | Other | betalactone | fengycin | 40 |
| JJ543 | *Bacillus toyonensis* | no | Sparse | Terpene | terpene | molybdenum cofactor | 17 |
| JJ543 | *Bacillus toyonensis* | no | Sparse | NRPS | NRPS | unknown | ND |
| JJ543 | *Bacillus toyonensis* | no | Sparse | NRPS | NRPS-like | unknown | ND |
| JJ543 | *Bacillus toyonensis* | no | Sparse | RiPP | LAP | unknown | ND |
| JJ543 | *Bacillus toyonensis* | no | Sparse | NRPS | NRPS | unknown | ND |
| JJ543 | *Bacillus toyonensis* | no | Sparse | RiPP | bacteriocin | unknown | ND |
| JJ543 | *Bacillus toyonensis* | no | Sparse | RiPP | lassopeptide | paeninodin | 60 |
| JJ543 | *Bacillus toyonensis* | no | Sparse | Other | siderophore | petrobactin | 100 |
| JJ555 | *Bacillus pumilus* | strong | Strong | NRPS | NRPS | bacillibactin | 53 |
| JJ555 | *Bacillus pumilus* | strong | Strong | Other | other | bacilysin | 85 |
| JJ555 | *Bacillus pumilus* | strong | Strong | Terpene | terpene | carotenoid | 50 |
| JJ555 | *Bacillus pumilus* | strong | Strong | Other | betalactone | fengycin | 53 |
| JJ555 | *Bacillus pumilus* | strong | Strong | NRPS | NRPS | lichenysin | 50 |
| JJ555 | *Bacillus pumilus* | strong | Strong | PKS | T3PKS | unknown | ND |
| JJ555 | *Bacillus pumilus* | strong | Strong | Other | betalactone | unknown | ND |
| JJ555 | *Bacillus pumilus* | strong | Strong | Terpene | terpene | unknown | ND |
| JJ555 | *Bacillus pumilus* | strong | Strong | RiPP | bacteriocin | unknown | ND |
| JJ555 | *Bacillus pumilus* | strong | Strong | PKS-NRPS | NRPS | zwittermicin A | 18 |
| JJ603 | *Bacillus pumilus* | weak | Strong | NRPS | NRPS | bacillibactin | 53 |
| JJ603 | *Bacillus pumilus* | weak | Strong | Other | other | bacilysin | 85 |
| JJ603 | *Bacillus pumilus* | weak | Strong | Terpene | terpene | carotenoid | 50 |
| JJ603 | *Bacillus pumilus* | weak | Strong | Other | betalactone | fengycin | 33 |
| JJ603 | *Bacillus pumilus* | weak | Strong | NRPS | NRPS | lichenysin | 50 |
| JJ603 | *Bacillus pumilus* | weak | Strong | Other | betalactone | unknown | ND |
| JJ603 | *Bacillus pumilus* | weak | Strong | PKS | T3PKS | unknown | ND |
| JJ603 | *Bacillus pumilus* | weak | Strong | PKS | T3PKS | unknown | ND |
| JJ603 | *Bacillus pumilus* | weak | Strong | RiPP | head_to_tail | sporulation killing factor | 85 |
| JJ603 | *Bacillus pumilus* | weak | Strong | PKS-NRPS | T1PKS | zwittermicin A | 18 |
| JJ61 | *Bacillus altitudinis* | strong | Sparse | NRPS | NRPS-like | locillomycin | 21 |
| JJ61 | *Bacillus altitudinis* | strong | Sparse | NRPS | NRPS | lichenysin | 85 |
| JJ61 | *Bacillus altitudinis* | strong | Sparse | RiPP | RRE-containing | unknown | ND |
| JJ61 | *Bacillus altitudinis* | strong | Sparse | Terpene | terpene | carotenoid | 50 |
| JJ61 | *Bacillus altitudinis* | strong | Sparse | Other | betalactone | fengycin | 53 |
| JJ61 | *Bacillus altitudinis* | strong | Sparse | Terpene | terpene | unknown | ND |
| JJ61 | *Bacillus altitudinis* | strong | Sparse | PKS | T3PKS | unknown | ND |
| JJ61 | *Bacillus altitudinis* | strong | Sparse | RiPP | RiPP-like | unknown | ND |
| JJ61 | *Bacillus altitudinis* | strong | Sparse | Other | betalactone | unknown | ND |
| JJ61 | *Bacillus altitudinis* | strong | Sparse | Other | other | bacilysin | 85 |
| JJ61 | *Bacillus altitudinis* | strong | Sparse | NRPS | NRPS | bacillibactin | 53 |
| JJ63 | *Bacillus (other)* | no | No | Other | T3PKS | fengycin | 40 |
| JJ63 | *Bacillus (other)* | no | No | Other | siderophore | unknown | ND |
| JJ63 | *Bacillus (other)* | no | No | RiPP | lanthipeptide | unknown | ND |
| JJ63 | *Bacillus (other)* | no | No | RiPP | LAP | unknown | ND |
| JJ63 | *Bacillus (other)* | no | No | Terpene | terpene | unknown | ND |
| JJ63 | *Bacillus (other)* | no | No | RiPP | lassopeptide | paeninodin | 100 |
| JJ634 | *Bacillus (other)* | no | No | Other | betalactone | fengycin | 26 |
| JJ634 | *Bacillus (other)* | no | No | PKS | T3PKS | unknown | ND |
| JJ634 | *Bacillus (other)* | no | No | Other | furan | unknown | ND |
| JJ634 | *Bacillus (other)* | no | No | RiPP | LAP | unknown | ND |
| JJ634 | *Bacillus (other)* | no | No | Terpene | terpene | unknown | ND |
| JJ634 | *Bacillus (other)* | no | No | Terpene | terpene | unknown | ND |
| JJ634 | *Bacillus (other)* | no | No | RiPP | lassopeptide | paeninodin | 100 |
| JJ634 | *Bacillus (other)* | no | No | Other | betalactone | platensimycin | 5 |
| JJ638 | *Bacillus velezensis* | no | Strong | PKS-NRPS | transAT-PKS-like | bacillaene | 100 |
| JJ638 | *Bacillus velezensis* | no | Strong | NRPS | bacteriocin | bacillibactin | 100 |
| JJ638 | *Bacillus velezensis* | no | Strong | Other | other | bacilysin | 100 |
| JJ638 | *Bacillus velezensis* | no | Strong | PKS | PKS-like | butirosin A | 7 |
| JJ638 | *Bacillus velezensis* | no | Strong | PKS | transAT-PKS | difficidin | 73 |
| JJ638 | *Bacillus velezensis* | no | Strong | Other | betalactone | fengycin | 80 |
| JJ638 | *Bacillus velezensis* | no | Strong | PKS | transAT-PKS | macrolactin H | 100 |
| JJ638 | *Bacillus velezensis* | no | Strong | PKS | T3PKS | unknown | ND |
| JJ638 | *Bacillus velezensis* | no | Strong | NRPS | NRPS | unknown | ND |
| JJ638 | *Bacillus velezensis* | no | Strong | Terpene | terpene | unknown | ND |
| JJ638 | *Bacillus velezensis* | no | Strong | Terpene | terpene | unknown | ND |
| JJ638 | *Bacillus velezensis* | no | Strong | RiPP | LAP | plantazolicin | 91 |
| JJ638 | *Bacillus velezensis* | no | Strong | NRPS | NRPS | surfactin | 91 |
| JJ642 | *Bacillus safensis* | strong | Strong | NRPS | NRPS | bacillibactin | 53 |
| JJ642 | *Bacillus safensis* | strong | Strong | Other | other | bacilysin | 71 |
| JJ642 | *Bacillus safensis* | strong | Strong | Other | other | carotenoid | 50 |
| JJ642 | *Bacillus safensis* | strong | Strong | Other | betalactone | fengycin | 53 |
| JJ642 | *Bacillus safensis* | strong | Strong | NRPS | NRPS | lichenysin | 28 |
| JJ642 | *Bacillus safensis* | strong | Strong | Other | betalactone | unknown | ND |
| JJ642 | *Bacillus safensis* | strong | Strong | Terpene | terpene | unknown | ND |
| JJ642 | *Bacillus safensis* | strong | Strong | PKS | T3PKS | unknown | ND |
| JJ642 | *Bacillus safensis* | strong | Strong | RiPP | LAP | plantazolicin | 25 |
| JJ642 | *Bacillus safensis* | strong | Strong | RiPP | head_to_tail | sporulation killing factor | 85 |
| JJ65 | *Gottfriedia acidiceleris* | no | No | Terpene | terpene | unknown | ND |
| JJ65 | *Gottfriedia acidiceleris* | no | No | RiPP | LAP | unknown | ND |
| JJ65 | *Gottfriedia acidiceleris* | no | No | Other | siderophore | unknown | ND |
| JJ65 | *Gottfriedia acidiceleris* | no | No | PKS | T3PKS | unknown | ND |
| JJ664 | *Bacillus (other)* | no | No | Terpene | terpene | glidobactin | 10 |
| JJ664 | *Bacillus (other)* | no | No | RiPP | LAP | unknown | ND |
| JJ664 | *Bacillus (other)* | no | No | PKS | T3PKS | unknown | ND |
| JJ665 | *Bacillus (other)* | no | No | RiPP | lassopeptide | exopolysaccharide | 11 |
| JJ665 | *Bacillus (other)* | no | No | Terpene | terpene | unknown | ND |
| JJ665 | *Bacillus (other)* | no | No | PKS | T1PKS | unknown | ND |
| JJ665 | *Bacillus (other)* | no | No | RiPP | lanthipeptide | unknown | ND |
| JJ675 | *Bacillus (other)* | no | No | NRPS | NRPS | bacillibactin | 46 |
| JJ675 | *Bacillus (other)* | no | No | NRPS | NRPS | bacitracin | 66 |
| JJ675 | *Bacillus (other)* | no | No | Other | betalactone | fengycin | 53 |
| JJ675 | *Bacillus (other)* | no | No | NRPS | NRPS | lichenysin | 57 |
| JJ675 | *Bacillus (other)* | no | No | PKS | T3PKS | unknown | ND |
| JJ675 | *Bacillus (other)* | no | No | Terpene | terpene | unknown | ND |
| JJ675 | *Bacillus (other)* | no | No | Other | siderophore | unknown | ND |
| JJ675 | *Bacillus (other)* | no | No | NRPS | NRPS | unknown | ND |
| JJ675 | *Bacillus (other)* | no | No | RiPP | head_to_tail | sporulation killing factor | 71 |
| JJ680 | *Bacillus pseudomycoides* | no | No | NRPS | NRPS | aunknownbaenopeptin | 100 |
| JJ680 | *Bacillus pseudomycoides* | no | No | NRPS | NRPS | bacillibactin | 38 |
| JJ680 | *Bacillus pseudomycoides* | no | No | Other | betalactone | fengycin | 40 |
| JJ680 | *Bacillus pseudomycoides* | no | No | RiPP | LAP | unknown | ND |
| JJ680 | *Bacillus pseudomycoides* | no | No | Terpene | terpene | unknown | ND |
| JJ680 | *Bacillus pseudomycoides* | no | No | RiPP | lanthipeptide | unknown | ND |
| JJ680 | *Bacillus pseudomycoides* | no | No | NRPS | NRPS | unknown | ND |
| JJ680 | *Bacillus pseudomycoides* | no | No | RiPP | lanthipeptide | unknown | ND |
| JJ680 | *Bacillus pseudomycoides* | no | No | RiPP | lassopeptide | paeninodin | 100 |
| JJ680 | *Bacillus pseudomycoides* | no | No | NRPS | NRPS | rhizomide A | 100 |
| JJ687 | *Neobacillus niacini* | no | No | PKS | T3PKS | unknown | ND |
| JJ687 | *Neobacillus niacini* | no | No | Other | betalactone | unknown | ND |
| JJ687 | *Neobacillus niacini* | no | No | Terpene | terpene | unknown | ND |
| JJ687 | *Neobacillus niacini* | no | No | RiPP | bacteriocin | unknown | ND |
| JJ687 | *Neobacillus niacini* | no | No | RiPP | LAP | unknown | ND |
| JJ687 | *Neobacillus niacini* | no | No | RiPP | lassopeptide | paeninodin | 100 |
| JJ689 | *Bacillus (other)* | weak | No | NRPS | NRPS | bacillibactin | 46 |
| JJ689 | *Bacillus (other)* | weak | No | Other | betalactone | fengycin | 40 |
| JJ689 | *Bacillus (other)* | weak | No | Terpene | terpene | molybdenum cofactor | 17 |
| JJ689 | *Bacillus (other)* | weak | No | NRPS | NRPS | unknown | ND |
| JJ689 | *Bacillus (other)* | weak | No | RiPP | LAP | unknown | ND |
| JJ689 | *Bacillus (other)* | weak | No | RiPP | sactipeptide | unknown | ND |
| JJ689 | *Bacillus (other)* | weak | No | Other | CDPS | unknown | ND |
| JJ689 | *Bacillus (other)* | weak | No | Other | siderophore | petrobactin | 100 |
| JJ689 | *Bacillus (other)* | weak | No | NRPS | NRPS | polyoxypeptin | 5 |
| JJ689 | *Bacillus (other)* | weak | No | Other | ladderane | S-layerglycan | 26 |
| JJ689 | *Bacillus (other)* | weak | No | PKS-NRPS | NRPS | zwittermicin A | 81 |
| JJ70 | *Bacillus toyonensis* | strong | Sparse | NRPS | NRPS | bacillibactin | 46 |
| JJ70 | *Bacillus toyonensis* | strong | Sparse | NRPS | NRPS | bogorol A | 11 |
| JJ70 | *Bacillus toyonensis* | strong | Sparse | Other | betalactone | fengycin | 40 |
| JJ70 | *Bacillus toyonensis* | strong | Sparse | Terpene | terpene | molybdenum cofactor | 17 |
| JJ70 | *Bacillus toyonensis* | strong | Sparse | NRPS | NRPS | unknown | ND |
| JJ70 | *Bacillus toyonensis* | strong | Sparse | RiPP | LAP | unknown | ND |
| JJ70 | *Bacillus toyonensis* | strong | Sparse | NRPS | NRPS | unknown | ND |
| JJ70 | *Bacillus toyonensis* | strong | Sparse | RiPP | bacteriocin | unknown | ND |
| JJ70 | *Bacillus toyonensis* | strong | Sparse | RiPP | bacteriocin | unknown | ND |
| JJ70 | *Bacillus toyonensis* | strong | Sparse | RiPP | lassopeptide | paeninodin | 80 |
| JJ70 | *Bacillus toyonensis* | strong | Sparse | Other | siderophore | petrobactin | 100 |
| JJ722 | *Bacillus (other)* | no | No | Other | betalactone | fengycin | 46 |
| JJ722 | *Bacillus (other)* | no | No | Terpene | terpene | hygrocin A | 6 |
| JJ722 | *Bacillus (other)* | no | No | Terpene | terpene | unknown | ND |
| JJ722 | *Bacillus (other)* | no | No | Other | betalactone | unknown | ND |
| JJ722 | *Bacillus (other)* | no | No | Other | betalactone | unknown | ND |
| JJ722 | *Bacillus (other)* | no | No | RiPP | LAP | unknown | ND |
| JJ723 | *Priestia megaterium* | no | No | Terpene | terpene | carotenoid | 50 |
| JJ723 | *Priestia megaterium* | no | No | Other | phosphonate | unknown | ND |
| JJ723 | *Priestia megaterium* | no | No | PKS | T3PKS | unknown | ND |
| JJ723 | *Priestia megaterium* | no | No | Other | siderophore | unknown | ND |
| JJ723 | *Priestia megaterium* | no | No | Terpene | terpene | unknown | ND |
| JJ723 | *Priestia megaterium* | no | No | RiPP | lanthipeptide | unknown | ND |
| JJ723 | *Priestia megaterium* | no | No | RiPP | lassopeptide | paeninodin | 60 |
| JJ723 | *Priestia megaterium* | no | No | Terpene | terpene | surfactin | 13 |
| JJ728 | *Priestia megaterium* | no | No | Terpene | terpene | carotenoid | 50 |
| JJ728 | *Priestia megaterium* | no | No | RiPP | lanthipeptide | mersacidin | 20 |
| JJ728 | *Priestia megaterium* | no | No | Other | phosphonate | unknown | ND |
| JJ728 | *Priestia megaterium* | no | No | PKS | T3PKS | unknown | ND |
| JJ728 | *Priestia megaterium* | no | No | Terpene | terpene | unknown | ND |
| JJ728 | *Priestia megaterium* | no | No | Other | siderophore | unknown | ND |
| JJ728 | *Priestia megaterium* | no | No | Terpene | terpene | surfactin | 13 |
| JJ732 | *Bacillus (other)* | no | No | NRPS | NRPS | bacillibactin | 46 |
| JJ732 | *Bacillus (other)* | no | No | Other | betalactone | fengycin | 40 |
| JJ732 | *Bacillus (other)* | no | No | Terpene | terpene | molybdenum cofactor | 17 |
| JJ732 | *Bacillus (other)* | no | No | NRPS | NRPS | unknown | ND |
| JJ732 | *Bacillus (other)* | no | No | RiPP | LAP | unknown | ND |
| JJ732 | *Bacillus (other)* | no | No | NRPS | NRPS | unknown | ND |
| JJ732 | *Bacillus (other)* | no | No | RiPP | bacteriocin | unknown | ND |
| JJ732 | *Bacillus (other)* | no | No | RiPP | lassopeptide | paeninodin | 100 |
| JJ732 | *Bacillus (other)* | no | No | Other | siderophore | petrobactin | 100 |
| JJ747 | *Bacillus velezensis* | strong | Strong | NRPS | NRPS | rhizomide A | 22 |
| JJ747 | *Bacillus velezensis* | strong | Strong | NRPS | NRPS | surfactin | 91 |
| JJ747 | *Bacillus velezensis* | strong | Strong | Other | cyclic-lactone-autoinducer | kijanimicin | 4 |
| JJ747 | *Bacillus velezensis* | strong | Strong | PKS | PKS-like | butirosin A | 7 |
| JJ747 | *Bacillus velezensis* | strong | Strong | Terpene | terpene | unknown | ND |
| JJ747 | *Bacillus velezensis* | strong | Strong | PKS | transAT-PKS | macrolactin H | 100 |
| JJ747 | *Bacillus velezensis* | strong | Strong | PKS-NRPS | transAT-PKS | bacillaene | 100 |
| JJ747 | *Bacillus velezensis* | strong | Strong | NRPS | NRPS | fengycin | 100 |
| JJ747 | *Bacillus velezensis* | strong | Strong | Terpene | terpene | micrococcin P | 8 |
| JJ747 | *Bacillus velezensis* | strong | Strong | PKS | T3PKS | unknown | ND |
| JJ747 | *Bacillus velezensis* | strong | Strong | PKS | transAT-PKS | difficidin | 100 |
| JJ747 | *Bacillus velezensis* | strong | Strong | NRPS | NRPS | bacillibactin | 100 |
| JJ747 | *Bacillus velezensis* | strong | Strong | Other | other | bacilysin | 100 |
| JJ747 | *Bacillus velezensis* | strong | Strong | NRPS | Others | iturin/Bacilomycin L | 100 |
| JJ748 | *Bacillus velezensis* | no | Strong | PKS-NRPS | transAT-PKS-like | bacillaene | 100 |
| JJ748 | *Bacillus velezensis* | no | Strong | NRPS | NRPS | bacillibactin | 100 |
| JJ748 | *Bacillus velezensis* | no | Strong | Other | other | bacilysin | 100 |
| JJ748 | *Bacillus velezensis* | no | Strong | PKS | PKS-like | butirosin A | 7 |
| JJ748 | *Bacillus velezensis* | no | Strong | PKS | transAT-PKS-like | difficidin | 33 |
| JJ748 | *Bacillus velezensis* | no | Strong | NRPS | NRPS | fengycin | 86 |
| JJ748 | *Bacillus velezensis* | no | Strong | PKS | transAT-PKS | macrolactin H | 100 |
| JJ748 | *Bacillus velezensis* | no | Strong | PKS | T3PKS | unknown | ND |
| JJ748 | *Bacillus velezensis* | no | Strong | Terpene | terpene | unknown | ND |
| JJ748 | *Bacillus velezensis* | no | Strong | Terpene | terpene | unknown | ND |
| JJ748 | *Bacillus velezensis* | no | Strong | NRPS | NRPS | unknown | ND |
| JJ748 | *Bacillus velezensis* | no | Strong | NRPS | NRPS | surfactin | 47 |
| JJ748 | *Bacillus velezensis* | no | Strong | NRPS | Others | iturin/Bacilomycin L | 86 |
| JJ749 | *Priestia megaterium* | no | No | Terpene | terpene | carotenoid | 50 |
| JJ749 | *Priestia megaterium* | no | No | PKS | T3PKS | unknown | ND |
| JJ749 | *Priestia megaterium* | no | No | Terpene | terpene | unknown | ND |
| JJ749 | *Priestia megaterium* | no | No | Other | siderophore | unknown | ND |
| JJ749 | *Priestia megaterium* | no | No | Other | phosphonate | unknown | ND |
| JJ749 | *Priestia megaterium* | no | No | RiPP | lanthipeptide | unknown | ND |
| JJ749 | *Priestia megaterium* | no | No | Terpene | terpene | surfactin | 13 |
| JJ759 | *Priestia megaterium* | no | No | Terpene | terpene | carotenoid | 50 |
| JJ759 | *Priestia megaterium* | no | No | PKS | T3PKS | unknown | ND |
| JJ759 | *Priestia megaterium* | no | No | Terpene | terpene | unknown | ND |
| JJ759 | *Priestia megaterium* | no | No | RiPP | LAP | unknown | ND |
| JJ759 | *Priestia megaterium* | no | No | Other | siderophore | unknown | ND |
| JJ759 | *Priestia megaterium* | no | No | Terpene | terpene | unknown | ND |
| JJ768 | *Bacillus thuringiensis* | no | Sparse | NRPS | NRPS | bacillibactin | 46 |
| JJ768 | *Bacillus thuringiensis* | no | Sparse | Other | betalactone | fengycin | 40 |
| JJ768 | *Bacillus thuringiensis* | no | Sparse | Terpene | terpene | molybdenum cofactor | 17 |
| JJ768 | *Bacillus thuringiensis* | no | Sparse | NRPS | NRPS | unknown | ND |
| JJ768 | *Bacillus thuringiensis* | no | Sparse | RiPP | lanthipeptide | unknown | ND |
| JJ768 | *Bacillus thuringiensis* | no | Sparse | RiPP | bacteriocin | unknown | ND |
| JJ768 | *Bacillus thuringiensis* | no | Sparse | Other | CDPS | unknown | ND |
| JJ768 | *Bacillus thuringiensis* | no | Sparse | Other | siderophore | petrobactin | 100 |
| JJ768 | *Bacillus thuringiensis* | no | Sparse | Other | ladderane | S-layerglycan | 20 |
| JJ783 | *Bacillus (other)* | no | No | NRPS | NRPS | bacillibactin | 46 |
| JJ783 | *Bacillus (other)* | no | No | Other | betalactone | fengycin | 40 |
| JJ783 | *Bacillus (other)* | no | No | RiPP | LAP | unknown | ND |
| JJ783 | *Bacillus (other)* | no | No | Terpene | terpene | unknown | ND |
| JJ783 | *Bacillus (other)* | no | No | RiPP | bacteriocin | unknown | ND |
| JJ783 | *Bacillus (other)* | no | No | RiPP | bacteriocin | unknown | ND |
| JJ783 | *Bacillus (other)* | no | No | RiPP | lassopeptide | paeninodin | 100 |
| JJ783 | *Bacillus (other)* | no | No | Other | siderophore | petrobactin | 100 |
| JJ789 | *Bacillus (other)* | no | No | NRPS | NRPS | bacillibactin | 46 |
| JJ789 | *Bacillus (other)* | no | No | Terpene | terpene | molybdenum cofactor | 17 |
| JJ789 | *Bacillus (other)* | no | No | NRPS | NRPS | unknown | ND |
| JJ789 | *Bacillus (other)* | no | No | NRPS | NRPS | unknown | ND |
| JJ789 | *Bacillus (other)* | no | No | RiPP | LAP | unknown | ND |
| JJ789 | *Bacillus (other)* | no | No | Other | betalactone | unknown | ND |
| JJ789 | *Bacillus (other)* | no | No | RiPP | bacteriocin | unknown | ND |
| JJ789 | *Bacillus (other)* | no | No | Other | siderophore | petrobactin | 100 |
| JJ793 | *Bacillus pseudomycoides* | no | No | NRPS | NRPS-like | aunknownbaenopeptin | 100 |
| JJ793 | *Bacillus pseudomycoides* | no | No | NRPS | NRPS | bacillibactin | 38 |
| JJ793 | *Bacillus pseudomycoides* | no | No | Other | betalactone | fengycin | 40 |
| JJ793 | *Bacillus pseudomycoides* | no | No | RiPP | LAP | unknown | ND |
| JJ793 | *Bacillus pseudomycoides* | no | No | NRPS | NRPS | unknown | ND |
| JJ793 | *Bacillus pseudomycoides* | no | No | Terpene | terpene | unknown | ND |
| JJ793 | *Bacillus pseudomycoides* | no | No | RiPP | lanthipeptide | unknown | ND |
| JJ793 | *Bacillus pseudomycoides* | no | No | Other | siderophore | unknown | ND |
| JJ793 | *Bacillus pseudomycoides* | no | No | RiPP | lassopeptide | paeninodin | 100 |
| JJ793 | *Bacillus pseudomycoides* | no | No | PKS-NRPS | NRPS | zwittermicin A | 22 |
| JJ807 | *Bacillus safensis* | no | Strong | NRPS | NRPS | bacillibactin | 53 |
| JJ807 | *Bacillus safensis* | no | Strong | Other | other | bacilysin | 85 |
| JJ807 | *Bacillus safensis* | no | Strong | Terpene | terpene | carotenoid | 50 |
| JJ807 | *Bacillus safensis* | no | Strong | Other | betalactone | fengycin | 53 |
| JJ807 | *Bacillus safensis* | no | Strong | NRPS | NRPS | lichenysin | 50 |
| JJ807 | *Bacillus safensis* | no | Strong | PKS | T3PKS | unknown | ND |
| JJ807 | *Bacillus safensis* | no | Strong | Other | betalactone | unknown | ND |
| JJ807 | *Bacillus safensis* | no | Strong | Terpene | terpene | unknown | ND |
| JJ807 | *Bacillus safensis* | no | Strong | RiPP | bacteriocin | unknown | ND |
| JJ807 | *Bacillus safensis* | no | Strong | RiPP | bacteriocin | unknown | ND |
| JJ807 | *Bacillus safensis* | no | Strong | RiPP | LAP | plantazolicin | 91 |
| JJ807 | *Bacillus safensis* | no | Strong | Other | ladderane | S-layerglycan | 20 |
| JJ807 | *Bacillus safensis* | no | Strong | RiPP | head_to_tail | sporulation killing factor | 85 |
| JJ814 | *Neobacillus drentensis* | no | No | Other | betalactone | fengycin | 40 |
| JJ814 | *Neobacillus drentensis* | no | No | PKS | T3PKS | unknown | ND |
| JJ814 | *Neobacillus drentensis* | no | No | PKS | T3PKS | unknown | ND |
| JJ814 | *Neobacillus drentensis* | no | No | Terpene | terpene | unknown | ND |
| JJ814 | *Neobacillus drentensis* | no | No | RiPP | bacteriocin | unknown | ND |
| JJ814 | *Neobacillus drentensis* | no | No | RiPP | lassopeptide | paeninodin | 80 |
| JJ833 | *Cytobacillus firmus* | no | Sparse | PKS | T1PKS | C-1027 | 4 |
| JJ833 | *Cytobacillus firmus* | no | Sparse | Other | ladderane | unknown | ND |
| JJ833 | *Cytobacillus firmus* | no | Sparse | PKS | T3PKS | unknown | ND |
| JJ833 | *Cytobacillus firmus* | no | Sparse | RiPP | LAP | unknown | ND |
| JJ833 | *Cytobacillus firmus* | no | Sparse | Other | siderophore | unknown | ND |
| JJ834 | *Bacillus safensis* | strong | Strong | NRPS | NRPS | bacillibactin | 53 |
| JJ834 | *Bacillus safensis* | strong | Strong | Other | other | bacilysin | 85 |
| JJ834 | *Bacillus safensis* | strong | Strong | Terpene | terpene | carotenoid | 50 |
| JJ834 | *Bacillus safensis* | strong | Strong | Other | betalactone | fengycin | 46 |
| JJ834 | *Bacillus safensis* | strong | Strong | NRPS | NRPS | lichenysin | 71 |
| JJ834 | *Bacillus safensis* | strong | Strong | Other | betalactone | unknown | ND |
| JJ834 | *Bacillus safensis* | strong | Strong | PKS | T3PKS | unknown | ND |
| JJ834 | *Bacillus safensis* | strong | Strong | Terpene | terpene | unknown | ND |
| JJ834 | *Bacillus safensis* | strong | Strong | RiPP | bacteriocin | unknown | ND |
| JJ834 | *Bacillus safensis* | strong | Strong | RiPP | LAP | plantazolicin | 50 |
| JJ846 | *Bacillus velezensis* | no | Strong | PKS-NRPS | transAT-PKS | bacillaene | 100 |
| JJ846 | *Bacillus velezensis* | no | Strong | NRPS | bacteriocin | bacillibactin | 100 |
| JJ846 | *Bacillus velezensis* | no | Strong | Other | other | bacilysin | 100 |
| JJ846 | *Bacillus velezensis* | no | Strong | PKS | PKS-like | butirosin A | 7 |
| JJ846 | *Bacillus velezensis* | no | Strong | PKS | transAT-PKS-like | difficidin | 100 |
| JJ846 | *Bacillus velezensis* | no | Strong | NRPS | NRPS | fengycin | 93 |
| JJ846 | *Bacillus velezensis* | no | Strong | PKS | transAT-PKS | macrolactin H | 90 |
| JJ846 | *Bacillus velezensis* | no | Strong | PKS | T3PKS | unknown | ND |
| JJ846 | *Bacillus velezensis* | no | Strong | Terpene | terpene | unknown | ND |
| JJ846 | *Bacillus velezensis* | no | Strong | Terpene | terpene | unknown | ND |
| JJ846 | *Bacillus velezensis* | no | Strong | NRPS | NRPS | surfactin | 82 |
| JJ846 | *Bacillus velezensis* | no | Strong | NRPS | Others | iturin/Bacilomycin L | 93 |
| JJ853 | *Neobacillus drentensis* | no | No | Other | betalactone | fengycin | 40 |
| JJ853 | *Neobacillus drentensis* | no | No | PKS | T3PKS | unknown | ND |
| JJ853 | *Neobacillus drentensis* | no | No | Terpene | terpene | unknown | ND |
| JJ853 | *Neobacillus drentensis* | no | No | RiPP | bacteriocin | unknown | ND |
| JJ858 | *Bacillus pseudomycoides* | no | No | NRPS | NRPS | aunknownbaenopeptin | 100 |
| JJ858 | *Bacillus pseudomycoides* | no | No | RiPP | sactipeptide | unknown | ND |
| JJ858 | *Bacillus pseudomycoides* | no | No | NRPS | NRPS | unknown | ND |
| JJ858 | *Bacillus pseudomycoides* | no | No | Terpene | terpene | unknown | ND |
| JJ858 | *Bacillus pseudomycoides* | no | No | NRPS | NRPS | unknown | ND |
| JJ858 | *Bacillus pseudomycoides* | no | No | RiPP | lassopeptide | paeninodin | 100 |
| JJ858 | *Bacillus pseudomycoides* | no | No | NRPS | NRPS | WAP-8294A2 | 30 |
| JJ860 | *Priestia megaterium* | no | No | Terpene | terpene | carotenoid | 50 |
| JJ860 | *Priestia megaterium* | no | No | PKS | T3PKS | unknown | ND |
| JJ860 | *Priestia megaterium* | no | No | Terpene | terpene | unknown | ND |
| JJ860 | *Priestia megaterium* | no | No | Other | siderophore | unknown | ND |
| JJ860 | *Priestia megaterium* | no | No | RiPP | lassopeptide | paeninodin | 100 |
| JJ860 | *Priestia megaterium* | no | No | Terpene | terpene | surfactin | 13 |
| JJ861 | *Bacillus pumilus* | strong | Strong | NRPS | NRPS | bacillibactin | 53 |
| JJ861 | *Bacillus pumilus* | strong | Strong | Other | other | bacilysin | 85 |
| JJ861 | *Bacillus pumilus* | strong | Strong | Terpene | terpene | carotenoid | 50 |
| JJ861 | *Bacillus pumilus* | strong | Strong | Other | betalactone | fengycin | 53 |
| JJ861 | *Bacillus pumilus* | strong | Strong | NRPS | NRPS | lichenysin | 50 |
| JJ861 | *Bacillus pumilus* | strong | Strong | Other | betalactone | unknown | ND |
| JJ861 | *Bacillus pumilus* | strong | Strong | NRPS | NRPS | unknown | ND |
| JJ861 | *Bacillus pumilus* | strong | Strong | PKS | T3PKS | unknown | ND |
| JJ861 | *Bacillus pumilus* | strong | Strong | Terpene | terpene | unknown | ND |
| JJ861 | *Bacillus pumilus* | strong | Strong | NRPS | NRPS | unknown | ND |
| JJ861 | *Bacillus pumilus* | strong | Strong | RiPP | head_to_tail | sporulation killing factor | 85 |
| JJ861 | *Bacillus pumilus* | strong | Strong | PKS-NRPS | NRPS | zwittermicin A | 18 |
| JJ864 | *Bacillus (other)* | no | No | Other | betalactone | fengycin | 33 |
| JJ864 | *Bacillus (other)* | no | No | Other | furan | methylenomycin A | 14 |
| JJ864 | *Bacillus (other)* | no | No | RiPP | LAP | unknown | ND |
| JJ864 | *Bacillus (other)* | no | No | Other | siderophore | unknown | ND |
| JJ864 | *Bacillus (other)* | no | No | Terpene | terpene | unknown | ND |
| JJ864 | *Bacillus (other)* | no | No | RiPP | bacteriocin | unknown | ND |
| JJ864 | *Bacillus (other)* | no | No | RiPP | bacteriocin | unknown | ND |
| JJ868 | *Bacillus (other)* | no | No | NRPS | NRPS | bacillibactin | 46 |
| JJ868 | *Bacillus (other)* | no | No | Other | betalactone | fengycin | 40 |
| JJ868 | *Bacillus (other)* | no | No | RiPP | LAP | unknown | ND |
| JJ868 | *Bacillus (other)* | no | No | Terpene | terpene | unknown | ND |
| JJ868 | *Bacillus (other)* | no | No | RiPP | lanthipeptide | unknown | ND |
| JJ868 | *Bacillus (other)* | no | No | RiPP | bacteriocin | unknown | ND |
| JJ868 | *Bacillus (other)* | no | No | RiPP | lassopeptide | paeninodin | 80 |
| JJ868 | *Bacillus (other)* | no | No | Other | siderophore | petrobactin | 100 |
| JJ883 | *Priestia megaterium* | no | No | Terpene | terpene | carotenoid | 50 |
| JJ883 | *Priestia megaterium* | no | No | Other | phosphonate | unknown | ND |
| JJ883 | *Priestia megaterium* | no | No | Other | siderophore | unknown | ND |
| JJ883 | *Priestia megaterium* | no | No | PKS | T3PKS | unknown | ND |
| JJ883 | *Priestia megaterium* | no | No | Terpene | terpene | unknown | ND |
| JJ883 | *Priestia megaterium* | no | No | Terpene | terpene | unknown | ND |
| JJ885 | *Bacillus pumilus* | weak | Strong | NRPS | NRPS | bacillibactin | 53 |
| JJ885 | *Bacillus pumilus* | weak | Strong | Other | other | bacilysin | 85 |
| JJ885 | *Bacillus pumilus* | weak | Strong | Other | other | carotenoid | 50 |
| JJ885 | *Bacillus pumilus* | weak | Strong | Other | betalactone | fengycin | 53 |
| JJ885 | *Bacillus pumilus* | weak | Strong | NRPS | NRPS | lichenysin | 50 |
| JJ885 | *Bacillus pumilus* | weak | Strong | Other | betalactone | unknown | ND |
| JJ885 | *Bacillus pumilus* | weak | Strong | PKS | T3PKS | unknown | ND |
| JJ885 | *Bacillus pumilus* | weak | Strong | Terpene | terpene | unknown | ND |
| JJ885 | *Bacillus pumilus* | weak | Strong | PKS-NRPS | NRPS | zwittermicin A | 18 |
| JJ887 | *Priestia megaterium* | no | No | Terpene | terpene | carotenoid | 50 |
| JJ887 | *Priestia megaterium* | no | No | Other | phosphonate | unknown | ND |
| JJ887 | *Priestia megaterium* | no | No | PKS | T3PKS | unknown | ND |
| JJ887 | *Priestia megaterium* | no | No | Terpene | terpene | unknown | ND |
| JJ887 | *Priestia megaterium* | no | No | Other | siderophore | unknown | ND |
| JJ887 | *Priestia megaterium* | no | No | Other | siderophore | unknown | ND |
| JJ887 | *Priestia megaterium* | no | No | RiPP | lassopeptide | paeninodin | 60 |
| JJ887 | *Priestia megaterium* | no | No | Terpene | terpene | surfactin | 13 |
| JJ889 | *Bacillus pseudomycoides* | no | No | Other | betalactone | fengycin | 20 |
| JJ889 | *Bacillus pseudomycoides* | no | No | Terpene | terpene | unknown | ND |
| JJ889 | *Bacillus pseudomycoides* | no | No | Other | siderophore | unknown | ND |
| JJ889 | *Bacillus pseudomycoides* | no | No | RiPP | LAP | unknown | ND |
| JJ889 | *Bacillus pseudomycoides* | no | No | RiPP | lassopeptide | paeninodin | 80 |
| JJ894 | *Bacillus pumilus* | strong | Strong | NRPS | NRPS | bacillibactin | 53 |
| JJ894 | *Bacillus pumilus* | strong | Strong | Other | other | bacilysin | 85 |
| JJ894 | *Bacillus pumilus* | strong | Strong | Terpene | terpene | carotenoid | 50 |
| JJ894 | *Bacillus pumilus* | strong | Strong | Other | betalactone | fengycin | 53 |
| JJ894 | *Bacillus pumilus* | strong | Strong | NRPS | NRPS | lichenysin | 28 |
| JJ894 | *Bacillus pumilus* | strong | Strong | NRPS | NRPS | unknown | ND |
| JJ894 | *Bacillus pumilus* | strong | Strong | PKS | T3PKS | unknown | ND |
| JJ894 | *Bacillus pumilus* | strong | Strong | RiPP | bacteriocin | unknown | ND |
| JJ894 | *Bacillus pumilus* | strong | Strong | Terpene | terpene | unknown | ND |
| JJ894 | *Bacillus pumilus* | strong | Strong | PKS-NRPS | NRPS | zwittermicin A | 14 |
| JJ902 | *Bacillus pumilus* | strong | Strong | NRPS | NRPS | bacillibactin | 53 |
| JJ902 | *Bacillus pumilus* | strong | Strong | Other | other | bacilysin | 85 |
| JJ902 | *Bacillus pumilus* | strong | Strong | Other | other | carotenoid | 50 |
| JJ902 | *Bacillus pumilus* | strong | Strong | Other | betalactone | fengycin | 53 |
| JJ902 | *Bacillus pumilus* | strong | Strong | NRPS | NRPS | lichenysin | 85 |
| JJ902 | *Bacillus pumilus* | strong | Strong | PKS | T3PKS | unknown | ND |
| JJ902 | *Bacillus pumilus* | strong | Strong | Other | betalactone | unknown | ND |
| JJ902 | *Bacillus pumilus* | strong | Strong | Terpene | terpene | unknown | ND |
| JJ902 | *Bacillus pumilus* | strong | Strong | RiPP | sactipeptide | sporulation killing factor | 85 |
| JJ902 | *Bacillus pumilus* | strong | Strong | PKS-NRPS | T1PKS | zwittermicin A | 18 |
| JJ904 | *Bacillus toyonensis* | no | Sparse | NRPS | NRPS | bacillibactin | 46 |
| JJ904 | *Bacillus toyonensis* | no | Sparse | Other | betalactone | fengycin | 40 |
| JJ904 | *Bacillus toyonensis* | no | Sparse | Terpene | terpene | molybdenum cofactor | 17 |
| JJ904 | *Bacillus toyonensis* | no | Sparse | NRPS | NRPS | unknown | ND |
| JJ904 | *Bacillus toyonensis* | no | Sparse | RiPP | LAP | unknown | ND |
| JJ904 | *Bacillus toyonensis* | no | Sparse | RiPP | lanthipeptide | unknown | ND |
| JJ904 | *Bacillus toyonensis* | no | Sparse | RiPP | bacteriocin | unknown | ND |
| JJ904 | *Bacillus toyonensis* | no | Sparse | RiPP | lassopeptide | paeninodin | 80 |
| JJ904 | *Bacillus toyonensis* | no | Sparse | Other | siderophore | petrobactin | 100 |
| JJ905 | *Bacillus pumilus* | strong | Strong | NRPS | NRPS | bacillibactin | 53 |
| JJ905 | *Bacillus pumilus* | strong | Strong | Other | other | bacilysin | 85 |
| JJ905 | *Bacillus pumilus* | strong | Strong | Terpene | terpene | carotenoid | 50 |
| JJ905 | *Bacillus pumilus* | strong | Strong | Other | betalactone | fengycin | 53 |
| JJ905 | *Bacillus pumilus* | strong | Strong | NRPS | NRPS | lichenysin | 50 |
| JJ905 | *Bacillus pumilus* | strong | Strong | NRPS | NRPS-like | locillomycin | 21 |
| JJ905 | *Bacillus pumilus* | strong | Strong | PKS | T3PKS | unknown | ND |
| JJ905 | *Bacillus pumilus* | strong | Strong | Other | betalactone | unknown | ND |
| JJ905 | *Bacillus pumilus* | strong | Strong | Terpene | terpene | unknown | ND |
| JJ905 | *Bacillus pumilus* | strong | Strong | RiPP | bacteriocin | unknown | ND |
| JJ905 | *Bacillus pumilus* | strong | Strong | PKS-NRPS | NRPS | zwittermicin A | 18 |
| JJ909 | *Priestia megaterium* | no | No | Terpene | terpene | carotenoid | 50 |
| JJ909 | *Priestia megaterium* | no | No | Other | siderophore | unknown | ND |
| JJ909 | *Priestia megaterium* | no | No | Terpene | terpene | unknown | ND |
| JJ909 | *Priestia megaterium* | no | No | Other | phosphonate | unknown | ND |
| JJ909 | *Priestia megaterium* | no | No | PKS | T3PKS | unknown | ND |
| JJ909 | *Priestia megaterium* | no | No | RiPP | lassopeptide | paeninodin | 60 |
| JJ911 | *Bacillus pumilus* | strong | Strong | NRPS | NRPS | bacillibactin | 53 |
| JJ911 | *Bacillus pumilus* | strong | Strong | Other | other | bacilysin | 85 |
| JJ911 | *Bacillus pumilus* | strong | Strong | Terpene | terpene | carotenoid | 50 |
| JJ911 | *Bacillus pumilus* | strong | Strong | Other | betalactone | fengycin | 53 |
| JJ911 | *Bacillus pumilus* | strong | Strong | NRPS | NRPS | lichenysin | 50 |
| JJ911 | *Bacillus pumilus* | strong | Strong | PKS | T3PKS | unknown | ND |
| JJ911 | *Bacillus pumilus* | strong | Strong | Other | betalactone | unknown | ND |
| JJ911 | *Bacillus pumilus* | strong | Strong | Terpene | terpene | unknown | ND |
| JJ911 | *Bacillus pumilus* | strong | Strong | RiPP | bacteriocin | unknown | ND |
| JJ911 | *Bacillus pumilus* | strong | Strong | RiPP | head_to_tail | sporulation killing factor | 100 |
| JJ911 | *Bacillus pumilus* | strong | Strong | PKS-NRPS | NRPS | zwittermicin A | 18 |
| JJ914 | *Bacillus velezensis* | no | Strong | PKS-NRPS | transAT-PKS-like | bacillaene | 100 |
| JJ914 | *Bacillus velezensis* | no | Strong | NRPS | bacteriocin | bacillibactin | 100 |
| JJ914 | *Bacillus velezensis* | no | Strong | Other | other | bacilysin | 100 |
| JJ914 | *Bacillus velezensis* | no | Strong | PKS | PKS-like | butirosin A | 7 |
| JJ914 | *Bacillus velezensis* | no | Strong | PKS | transAT-PKS-like | difficidin | 66 |
| JJ914 | *Bacillus velezensis* | no | Strong | Other | betalactone | fengycin | 80 |
| JJ914 | *Bacillus velezensis* | no | Strong | PKS | transAT-PKS | macrolactin H | 100 |
| JJ914 | *Bacillus velezensis* | no | Strong | PKS | T3PKS | unknown | ND |
| JJ914 | *Bacillus velezensis* | no | Strong | RiPP | lanthipeptide | unknown | ND |
| JJ914 | *Bacillus velezensis* | no | Strong | Terpene | terpene | unknown | ND |
| JJ914 | *Bacillus velezensis* | no | Strong | Terpene | terpene | unknown | ND |
| JJ914 | *Bacillus velezensis* | no | Strong | NRPS | NRPS | surfactin | 82 |
| JJ917 | *Bacillus safensis* | weak | Strong | NRPS | NRPS | bacillibactin | 53 |
| JJ917 | *Bacillus safensis* | weak | Strong | Other | other | bacilysin | 85 |
| JJ917 | *Bacillus safensis* | weak | Strong | Other | other | carotenoid | 50 |
| JJ917 | *Bacillus safensis* | weak | Strong | Other | betalactone | fengycin | 53 |
| JJ917 | *Bacillus safensis* | weak | Strong | NRPS | NRPS | lichenysin | 85 |
| JJ917 | *Bacillus safensis* | weak | Strong | Other | betalactone | unknown | ND |
| JJ917 | *Bacillus safensis* | weak | Strong | Terpene | terpene | unknown | ND |
| JJ917 | *Bacillus safensis* | weak | Strong | PKS | T3PKS | unknown | ND |
| JJ917 | *Bacillus safensis* | weak | Strong | RiPP | bacteriocin | unknown | ND |
| JJ917 | *Bacillus safensis* | weak | Strong | RiPP | LAP | plantazolicin | 25 |
| JJ917 | *Bacillus safensis* | weak | Strong | RiPP | head_to_tail | sporulation killing factor | 85 |
| JJ919 | *Neobacillus drentensis* | no | No | Other | betalactone | fengycin | 40 |
| JJ919 | *Neobacillus drentensis* | no | No | PKS | T3PKS | unknown | ND |
| JJ919 | *Neobacillus drentensis* | no | No | PKS | T3PKS | unknown | ND |
| JJ919 | *Neobacillus drentensis* | no | No | Terpene | terpene | unknown | ND |
| JJ919 | *Neobacillus drentensis* | no | No | RiPP | bacteriocin | unknown | ND |
| JJ919 | *Neobacillus drentensis* | no | No | RiPP | lassopeptide | paeninodin | 60 |
| JJ920 | *Priestia megaterium* | no | No | Terpene | terpene | carotenoid | 50 |
| JJ920 | *Priestia megaterium* | no | No | Other | phosphonate | unknown | ND |
| JJ920 | *Priestia megaterium* | no | No | PKS | T3PKS | unknown | ND |
| JJ920 | *Priestia megaterium* | no | No | Terpene | terpene | unknown | ND |
| JJ920 | *Priestia megaterium* | no | No | Other | CDPS | unknown | ND |
| JJ920 | *Priestia megaterium* | no | No | Other | siderophore | unknown | ND |
| JJ920 | *Priestia megaterium* | no | No | Terpene | terpene | surfactin | 13 |
| JJ921 | *Priestia megaterium* | no | No | Terpene | terpene | carotenoid | 50 |
| JJ921 | *Priestia megaterium* | no | No | PKS | T3PKS | unknown | ND |
| JJ921 | *Priestia megaterium* | no | No | Other | phosphonate | unknown | ND |
| JJ921 | *Priestia megaterium* | no | No | Terpene | terpene | unknown | ND |
| JJ921 | *Priestia megaterium* | no | No | Other | siderophore | unknown | ND |
| JJ921 | *Priestia megaterium* | no | No | RiPP | lassopeptide | paeninodin | 60 |
| JJ921 | *Priestia megaterium* | no | No | Terpene | terpene | surfactin | 13 |
| JJ923 | *Priestia megaterium* | no | No | Terpene | terpene | carotenoid | 50 |
| JJ923 | *Priestia megaterium* | no | No | Terpene | terpene | locillomycin | 14 |
| JJ923 | *Priestia megaterium* | no | No | PKS | T3PKS | unknown | ND |
| JJ923 | *Priestia megaterium* | no | No | Terpene | terpene | unknown | ND |
| JJ923 | *Priestia megaterium* | no | No | Other | siderophore | unknown | ND |
| JJ923 | *Priestia megaterium* | no | No | Other | phosphonate | unknown | ND |
| JJ923 | *Priestia megaterium* | no | No | RiPP | lanthipeptide | unknown | ND |
| JJ923 | *Priestia megaterium* | no | No | RiPP | lassopeptide | paeninodin | 60 |
| JJ927 | *Bacillus (other)* | no | No | NRPS | NRPS | bacillibactin | 46 |
| JJ927 | *Bacillus (other)* | no | No | Other | betalactone | fengycin | 40 |
| JJ927 | *Bacillus (other)* | no | No | NRPS | NRPS-like | unknown | ND |
| JJ927 | *Bacillus (other)* | no | No | NRPS | NRPS | unknown | ND |
| JJ927 | *Bacillus (other)* | no | No | RiPP | LAP | unknown | ND |
| JJ927 | *Bacillus (other)* | no | No | Terpene | terpene | unknown | ND |
| JJ927 | *Bacillus (other)* | no | No | RiPP | bacteriocin | unknown | ND |
| JJ927 | *Bacillus (other)* | no | No | RiPP | lassopeptide | paeninodin | 100 |
| JJ927 | *Bacillus (other)* | no | No | RiPP | lassopeptide | paeninodin | 100 |
| JJ927 | *Bacillus (other)* | no | No | Other | siderophore | petrobactin | 100 |
| JJ928 | *Bacillus velezensis* | no | Strong | PKS-NRPS | transAT-PKS-like | bacillaene | 100 |
| JJ928 | *Bacillus velezensis* | no | Strong | NRPS | NRPS | bacillibactin | 100 |
| JJ928 | *Bacillus velezensis* | no | Strong | Other | other | bacilysin | 100 |
| JJ928 | *Bacillus velezensis* | no | Strong | PKS | PKS-like | butirosin A | 7 |
| JJ928 | *Bacillus velezensis* | no | Strong | PKS | transAT-PKS-like | difficidin | 53 |
| JJ928 | *Bacillus velezensis* | no | Strong | NRPS | NRPS | fengycin | 86 |
| JJ928 | *Bacillus velezensis* | no | Strong | PKS | transAT-PKS | macrolactin H | 100 |
| JJ928 | *Bacillus velezensis* | no | Strong | PKS | T3PKS | unknown | ND |
| JJ928 | *Bacillus velezensis* | no | Strong | RiPP | LAP | unknown | ND |
| JJ928 | *Bacillus velezensis* | no | Strong | Terpene | terpene | unknown | ND |
| JJ928 | *Bacillus velezensis* | no | Strong | Terpene | terpene | unknown | ND |
| JJ928 | *Bacillus velezensis* | no | Strong | NRPS | NRPS | surfactin | 47 |
| JJ928 | *Bacillus velezensis* | no | Strong | NRPS | Others | iturin/Bacilomycin L | 86 |
| JJ929 | *Bacillus velezensis* | weak | Strong | PKS-NRPS | transAT-PKS-like | bacillaene | 100 |
| JJ929 | *Bacillus velezensis* | weak | Strong | NRPS | bacteriocin | bacillibactin | 100 |
| JJ929 | *Bacillus velezensis* | weak | Strong | Other | other | bacilysin | 100 |
| JJ929 | *Bacillus velezensis* | weak | Strong | PKS | PKS-like | butirosin A | 7 |
| JJ929 | *Bacillus velezensis* | weak | Strong | PKS | transAT-PKS-like | difficidin | 53 |
| JJ929 | *Bacillus velezensis* | weak | Strong | NRPS | NRPS | fengycin | 86 |
| JJ929 | *Bacillus velezensis* | weak | Strong | PKS | transAT-PKS | macrolactin H | 100 |
| JJ929 | *Bacillus velezensis* | weak | Strong | PKS | T3PKS | unknown | ND |
| JJ929 | *Bacillus velezensis* | weak | Strong | RiPP | thiopeptide | unknown | ND |
| JJ929 | *Bacillus velezensis* | weak | Strong | Terpene | terpene | unknown | ND |
| JJ929 | *Bacillus velezensis* | weak | Strong | Terpene | terpene | unknown | ND |
| JJ929 | *Bacillus velezensis* | weak | Strong | NRPS | NRPS | surfactin | 47 |
| JJ929 | *Bacillus velezensis* | weak | Strong | NRPS | Others | iturin/Bacilomycin L | 86 |
| JJ935 | *Bacillus altitudinis* | no | Sparse | Other | other | bacilysin | 85 |
| JJ935 | *Bacillus altitudinis* | no | Sparse | Other | other | carotenoid | 50 |
| JJ935 | *Bacillus altitudinis* | no | Sparse | Other | betalactone | fengycin | 53 |
| JJ935 | *Bacillus altitudinis* | no | Sparse | NRPS | NRPS | lichenysin | 85 |
| JJ935 | *Bacillus altitudinis* | no | Sparse | Other | betalactone | unknown | ND |
| JJ935 | *Bacillus altitudinis* | no | Sparse | Terpene | terpene | unknown | ND |
| JJ935 | *Bacillus altitudinis* | no | Sparse | PKS | T3PKS | unknown | ND |
| JJ935 | *Bacillus altitudinis* | no | Sparse | RiPP | bacteriocin | unknown | ND |
| JJ937 | *Bacillus safensis* | no | Strong | NRPS | NRPS | bacillibactin | 53 |
| JJ937 | *Bacillus safensis* | no | Strong | Other | other | bacilysin | 71 |
| JJ937 | *Bacillus safensis* | no | Strong | Other | other | carotenoid | 50 |
| JJ937 | *Bacillus safensis* | no | Strong | Other | betalactone | fengycin | 53 |
| JJ937 | *Bacillus safensis* | no | Strong | NRPS | NRPS | lichenysin | 50 |
| JJ937 | *Bacillus safensis* | no | Strong | PKS | T3PKS | unknown | ND |
| JJ937 | *Bacillus safensis* | no | Strong | Other | betalactone | unknown | ND |
| JJ937 | *Bacillus safensis* | no | Strong | Terpene | terpene | unknown | ND |
| JJ937 | *Bacillus safensis* | no | Strong | RiPP | bacteriocin | unknown | ND |
| JJ937 | *Bacillus safensis* | no | Strong | Other | ladderane | unknown | ND |
| JJ937 | *Bacillus safensis* | no | Strong | RiPP | LAP | plantazolicin | 50 |
| JJ937 | *Bacillus safensis* | no | Strong | RiPP | head_to_tail | sporulation killing factor | 85 |
| JJ939 | *Priestia megaterium* | no | No | Terpene | terpene | carotenoid | 50 |
| JJ939 | *Priestia megaterium* | no | No | Terpene | terpene | locillomycin | 14 |
| JJ939 | *Priestia megaterium* | no | No | PKS | T3PKS | unknown | ND |
| JJ939 | *Priestia megaterium* | no | No | Other | phosphonate | unknown | ND |
| JJ939 | *Priestia megaterium* | no | No | Terpene | terpene | unknown | ND |
| JJ939 | *Priestia megaterium* | no | No | Other | siderophore | unknown | ND |
| JJ939 | *Priestia megaterium* | no | No | RiPP | lassopeptide | paeninodin | 60 |
| JJ941 | *Bacillus velezensis* | no | Strong | PKS-NRPS | transAT-PKS-like | bacillaene | 100 |
| JJ941 | *Bacillus velezensis* | no | Strong | NRPS | bacteriocin | bacillibactin | 84 |
| JJ941 | *Bacillus velezensis* | no | Strong | Other | other | bacilysin | 100 |
| JJ941 | *Bacillus velezensis* | no | Strong | PKS | PKS-like | butirosin A | 7 |
| JJ941 | *Bacillus velezensis* | no | Strong | PKS | transAT-PKS | difficidin | 46 |
| JJ941 | *Bacillus velezensis* | no | Strong | Other | betalactone | fengycin | 80 |
| JJ941 | *Bacillus velezensis* | no | Strong | PKS | transAT-PKS | macrolactin H | 100 |
| JJ941 | *Bacillus velezensis* | no | Strong | PKS | T3PKS | unknown | ND |
| JJ941 | *Bacillus velezensis* | no | Strong | Terpene | terpene | unknown | ND |
| JJ941 | *Bacillus velezensis* | no | Strong | Terpene | terpene | unknown | ND |
| JJ941 | *Bacillus velezensis* | no | Strong | NRPS | NRPS | surfactin | 47 |
| JJ943 | *Bacillus velezensis* | no | Strong | PKS-NRPS | transAT-PKS | bacillaene | 100 |
| JJ943 | *Bacillus velezensis* | no | Strong | NRPS | bacteriocin | bacillibactin | 100 |
| JJ943 | *Bacillus velezensis* | no | Strong | Other | other | bacilysin | 100 |
| JJ943 | *Bacillus velezensis* | no | Strong | PKS | PKS-like | butirosin A | 7 |
| JJ943 | *Bacillus velezensis* | no | Strong | PKS | transAT-PKS-like | difficidin | 53 |
| JJ943 | *Bacillus velezensis* | no | Strong | NRPS | NRPS | fengycin | 86 |
| JJ943 | *Bacillus velezensis* | no | Strong | PKS | transAT-PKS | macrolactin H | 100 |
| JJ943 | *Bacillus velezensis* | no | Strong | PKS | T3PKS | unknown | ND |
| JJ943 | *Bacillus velezensis* | no | Strong | RiPP | thiopeptide | unknown | ND |
| JJ943 | *Bacillus velezensis* | no | Strong | Terpene | terpene | unknown | ND |
| JJ943 | *Bacillus velezensis* | no | Strong | Terpene | terpene | unknown | ND |
| JJ943 | *Bacillus velezensis* | no | Strong | NRPS | NRPS | surfactin | 91 |
| JJ943 | *Bacillus velezensis* | no | Strong | NRPS | Others | iturin/Bacilomycin L | 86 |
| JJ944 | *Bacillus subtilis* | strong | Strong | PKS-NRPS | transAT-PKS | bacillaene | 100 |
| JJ944 | *Bacillus subtilis* | strong | Strong | NRPS | NRPS | bacillibactin | 100 |
| JJ944 | *Bacillus subtilis* | strong | Strong | Other | other | bacilysin | 100 |
| JJ944 | *Bacillus subtilis* | strong | Strong | NRPS | NRPS | fengycin | 80 |
| JJ944 | *Bacillus subtilis* | strong | Strong | PKS | T3PKS | unknown | ND |
| JJ944 | *Bacillus subtilis* | strong | Strong | Terpene | terpene | unknown | ND |
| JJ944 | *Bacillus subtilis* | strong | Strong | Terpene | terpene | unknown | ND |
| JJ944 | *Bacillus subtilis* | strong | Strong | RiPP | sactipeptide | subtilosin A | 100 |
| JJ944 | *Bacillus subtilis* | strong | Strong | NRPS | NRPS | surfactin | 43 |
| JJ945 | *Bacillus safensis* | no | Strong | NRPS | NRPS | bacillibactin | 53 |
| JJ945 | *Bacillus safensis* | no | Strong | Other | other | bacilysin | 85 |
| JJ945 | *Bacillus safensis* | no | Strong | Terpene | terpene | carotenoid | 50 |
| JJ945 | *Bacillus safensis* | no | Strong | Other | betalactone | fengycin | 53 |
| JJ945 | *Bacillus safensis* | no | Strong | NRPS | NRPS | lichenysin | 50 |
| JJ945 | *Bacillus safensis* | no | Strong | Other | betalactone | unknown | ND |
| JJ945 | *Bacillus safensis* | no | Strong | Terpene | terpene | unknown | ND |
| JJ945 | *Bacillus safensis* | no | Strong | PKS | T3PKS | unknown | ND |
| JJ945 | *Bacillus safensis* | no | Strong | RiPP | bacteriocin | unknown | ND |
| JJ945 | *Bacillus safensis* | no | Strong | RiPP | LAP | plantazolicin | 50 |
| JJ945 | *Bacillus safensis* | no | Strong | Other | ladderane | S-layerglycan | 13 |
| JJ945 | *Bacillus safensis* | no | Strong | RiPP | sactipeptide | sporulation killing factor | 85 |
| JJ946 | *Bacillus velezensis* | no | Strong | PKS-NRPS | transAT-PKS-like | bacillaene | 100 |
| JJ946 | *Bacillus velezensis* | no | Strong | NRPS | bacteriocin | bacillibactin | 100 |
| JJ946 | *Bacillus velezensis* | no | Strong | Other | other | bacilysin | 100 |
| JJ946 | *Bacillus velezensis* | no | Strong | PKS | PKS-like | butirosin A | 7 |
| JJ946 | *Bacillus velezensis* | no | Strong | PKS | transAT-PKS-like | difficidin | 66 |
| JJ946 | *Bacillus velezensis* | no | Strong | NRPS | NRPS | fengycin | 73 |
| JJ946 | *Bacillus velezensis* | no | Strong | PKS | transAT-PKS | macrolactin H | 100 |
| JJ946 | *Bacillus velezensis* | no | Strong | PKS | T3PKS | unknown | ND |
| JJ946 | *Bacillus velezensis* | no | Strong | RiPP | lanthipeptide | unknown | ND |
| JJ946 | *Bacillus velezensis* | no | Strong | Terpene | terpene | unknown | ND |
| JJ946 | *Bacillus velezensis* | no | Strong | Terpene | terpene | unknown | ND |
| JJ946 | *Bacillus velezensis* | no | Strong | NRPS | NRPS | surfactin | 82 |
| JJ946 | *Bacillus velezensis* | no | Strong | NRPS | Others | iturin/Bacilomycin L | 73 |
| JJ947 | *Bacillus velezensis* | strong | Strong | RiPP | lanthipeptide-class-ii | unknown | ND |
| JJ947 | *Bacillus velezensis* | strong | Strong | NRPS | NRPS | surfactin | 91 |
| JJ947 | *Bacillus velezensis* | strong | Strong | PKS | PKS-like | butirosin A | 7 |
| JJ947 | *Bacillus velezensis* | strong | Strong | Terpene | terpene | unknown | ND |
| JJ947 | *Bacillus velezensis* | strong | Strong | PKS | transAT-PKS | macrolactin H | 100 |
| JJ947 | *Bacillus velezensis* | strong | Strong | PKS-NRPS | transAT-PKS | bacillaene | 100 |
| JJ947 | *Bacillus velezensis* | strong | Strong | NRPS | NRPS | fengycin | 100 |
| JJ947 | *Bacillus velezensis* | strong | Strong | Terpene | terpene | unknown | ND |
| JJ947 | *Bacillus velezensis* | strong | Strong | PKS | T3PKS | unknown | ND |
| JJ947 | *Bacillus velezensis* | strong | Strong | PKS | transAT-PKS | difficidin | 100 |
| JJ947 | *Bacillus velezensis* | strong | Strong | NRPS | NRPS | bacillibactin | 100 |
| JJ947 | *Bacillus velezensis* | strong | Strong | Other | other | bacilysin | 100 |
| JJ947 | *Bacillus velezensis* | strong | Strong | NRPS | Others | iturin/Bacilomycin L | 100 |
| JJ950 | *Bacillus pumilus* | strong | Strong | RiPP | sactipeptide | sporulation killing factor | 85 |
| JJ950 | *Bacillus pumilus* | strong | Strong | NRPS | NRPS | lichenysin | 85 |
| JJ950 | *Bacillus pumilus* | strong | Strong | PKS-NRPS | NRPS | zwittermicin A | 18 |
| JJ950 | *Bacillus pumilus* | strong | Strong | RiPP | RRE-containing | unknown | ND |
| JJ950 | *Bacillus pumilus* | strong | Strong | Terpene | terpene | carotenoid | 50 |
| JJ950 | *Bacillus pumilus* | strong | Strong | Other | betalactone | fengycin | 53 |
| JJ950 | *Bacillus pumilus* | strong | Strong | Terpene | terpene | unknown | ND |
| JJ950 | *Bacillus pumilus* | strong | Strong | PKS | T3PKS | unknown | ND |
| JJ950 | *Bacillus pumilus* | strong | Strong | Other | ladderane | lipopolysaccharide | 11 |
| JJ950 | *Bacillus pumilus* | strong | Strong | Other | betalactone | unknown | ND |
| JJ950 | *Bacillus pumilus* | strong | Strong | RiPP | RiPP-like | unknown | ND |
| JJ950 | *Bacillus pumilus* | strong | Strong | Other | other | bacilysin | 85 |
| JJ950 | *Bacillus pumilus* | strong | Strong | NRPS | NRPS | bacillibactin | 53 |
| JJ951 | *Bacillus velezensis* | strong | Strong | NRPS | NRPS | surfactin | 82 |
| JJ951 | *Bacillus velezensis* | strong | Strong | PKS | PKS-like | butirosin A | 7 |
| JJ951 | *Bacillus velezensis* | strong | Strong | Terpene | terpene | unknown | ND |
| JJ951 | *Bacillus velezensis* | strong | Strong | RiPP | lanthipeptide-class-ii | unknown | ND |
| JJ951 | *Bacillus velezensis* | strong | Strong | PKS | transAT-PKS | macrolactin H | 100 |
| JJ951 | *Bacillus velezensis* | strong | Strong | PKS-NRPS | transAT-PKS | bacillaene | 100 |
| JJ951 | *Bacillus velezensis* | strong | Strong | NRPS | NRPS | fengycin | 100 |
| JJ951 | *Bacillus velezensis* | strong | Strong | Terpene | terpene | unknown | ND |
| JJ951 | *Bacillus velezensis* | strong | Strong | PKS | T3PKS | unknown | ND |
| JJ951 | *Bacillus velezensis* | strong | Strong | PKS | transAT-PKS | difficidin | 100 |
| JJ951 | *Bacillus velezensis* | strong | Strong | NRPS | NRPS | bacillibactin | 100 |
| JJ951 | *Bacillus velezensis* | strong | Strong | Other | other | bacilysin | 100 |
| JJ951 | *Bacillus velezensis* | strong | Strong | NRPS | Others | iturin/Bacilomycin L | 100 |
| JJ952 | *Bacillus altitudinis* | no | Sparse | Other | other | bacilysin | 85 |
| JJ952 | *Bacillus altitudinis* | no | Sparse | Other | other | carotenoid | 50 |
| JJ952 | *Bacillus altitudinis* | no | Sparse | Other | betalactone | fengycin | 53 |
| JJ952 | *Bacillus altitudinis* | no | Sparse | NRPS | NRPS | lichenysin | 85 |
| JJ952 | *Bacillus altitudinis* | no | Sparse | PKS | T3PKS | unknown | ND |
| JJ952 | *Bacillus altitudinis* | no | Sparse | Other | betalactone | unknown | ND |
| JJ952 | *Bacillus altitudinis* | no | Sparse | Terpene | terpene | unknown | ND |
| JJ952 | *Bacillus altitudinis* | no | Sparse | RiPP | bacteriocin | unknown | ND |
| JJ953 | *Bacillus safensis* | weak | Strong | NRPS | NRPS | bacillibactin | 53 |
| JJ953 | *Bacillus safensis* | weak | Strong | Other | other | bacilysin | 85 |
| JJ953 | *Bacillus safensis* | weak | Strong | Other | betalactone | bottromycin A | 6 |
| JJ953 | *Bacillus safensis* | weak | Strong | Other | other | carotenoid | 50 |
| JJ953 | *Bacillus safensis* | weak | Strong | Other | betalactone | fengycin | 53 |
| JJ953 | *Bacillus safensis* | weak | Strong | NRPS | NRPS | lichenysin | 85 |
| JJ953 | *Bacillus safensis* | weak | Strong | PKS | T3PKS | unknown | ND |
| JJ953 | *Bacillus safensis* | weak | Strong | Terpene | terpene | unknown | ND |
| JJ953 | *Bacillus safensis* | weak | Strong | RiPP | bacteriocin | unknown | ND |
| JJ953 | *Bacillus safensis* | weak | Strong | RiPP | LAP | plantazolicin | 50 |
| JJ953 | *Bacillus safensis* | weak | Strong | PKS-NRPS | NRPS | zwittermicin A | 18 |
| JJ956 | *Bacillus velezensis* | weak | Strong | PKS-NRPS | transAT-PKS-like | bacillaene | 100 |
| JJ956 | *Bacillus velezensis* | weak | Strong | NRPS | NRPS | bacillibactin | 100 |
| JJ956 | *Bacillus velezensis* | weak | Strong | Other | other | bacilysin | 100 |
| JJ956 | *Bacillus velezensis* | weak | Strong | PKS | PKS-like | butirosin A | 7 |
| JJ956 | *Bacillus velezensis* | weak | Strong | PKS | transAT-PKS-like | difficidin | 53 |
| JJ956 | *Bacillus velezensis* | weak | Strong | NRPS | NRPS | fengycin | 86 |
| JJ956 | *Bacillus velezensis* | weak | Strong | PKS | transAT-PKS | macrolactin H | 100 |
| JJ956 | *Bacillus velezensis* | weak | Strong | PKS | T3PKS | unknown | ND |
| JJ956 | *Bacillus velezensis* | weak | Strong | RiPP | LAP | unknown | ND |
| JJ956 | *Bacillus velezensis* | weak | Strong | Terpene | terpene | unknown | ND |
| JJ956 | *Bacillus velezensis* | weak | Strong | Terpene | terpene | unknown | ND |
| JJ956 | *Bacillus velezensis* | weak | Strong | NRPS | NRPS | surfactin | 47 |
| JJ956 | *Bacillus velezensis* | weak | Strong | NRPS | Others | iturin/Bacilomycin L | 86 |
| JJ959 | *Bacillus velezensis* | no | Strong | PKS-NRPS | transAT-PKS | bacillaene | 100 |
| JJ959 | *Bacillus velezensis* | no | Strong | NRPS | bacteriocin | bacillibactin | 84 |
| JJ959 | *Bacillus velezensis* | no | Strong | Other | other | bacilysin | 100 |
| JJ959 | *Bacillus velezensis* | no | Strong | PKS | PKS-like | butirosin A | 7 |
| JJ959 | *Bacillus velezensis* | no | Strong | PKS | transAT-PKS-like | difficidin | 66 |
| JJ959 | *Bacillus velezensis* | no | Strong | NRPS | NRPS | fengycin | 80 |
| JJ959 | *Bacillus velezensis* | no | Strong | PKS | transAT-PKS | macrolactin H | 100 |
| JJ959 | *Bacillus velezensis* | no | Strong | PKS | T3PKS | unknown | ND |
| JJ959 | *Bacillus velezensis* | no | Strong | Terpene | terpene | unknown | ND |
| JJ959 | *Bacillus velezensis* | no | Strong | Terpene | terpene | unknown | ND |
| JJ959 | *Bacillus velezensis* | no | Strong | RiPP | lanthipeptide | unknown | ND |
| JJ959 | *Bacillus velezensis* | no | Strong | NRPS | NRPS | unknown | ND |
| JJ959 | *Bacillus velezensis* | no | Strong | NRPS | NRPS | surfactin | 47 |
| JJ959 | *Bacillus velezensis* | no | Strong | NRPS | Others | iturin/Bacilomycin L | 80 |
| JJ962 | *Bacillus velezensis* | weak | Strong | PKS-NRPS | transAT-PKS | bacillaene | 100 |
| JJ962 | *Bacillus velezensis* | weak | Strong | NRPS | bacteriocin | bacillibactin | 100 |
| JJ962 | *Bacillus velezensis* | weak | Strong | Other | other | bacilysin | 100 |
| JJ962 | *Bacillus velezensis* | weak | Strong | PKS | PKS-like | butirosin A | 7 |
| JJ962 | *Bacillus velezensis* | weak | Strong | PKS | transAT-PKS-like | difficidin | 53 |
| JJ962 | *Bacillus velezensis* | weak | Strong | NRPS | NRPS | fengycin | 86 |
| JJ962 | *Bacillus velezensis* | weak | Strong | PKS | transAT-PKS | macrolactin H | 100 |
| JJ962 | *Bacillus velezensis* | weak | Strong | PKS | T3PKS | unknown | ND |
| JJ962 | *Bacillus velezensis* | weak | Strong | RiPP | LAP | unknown | ND |
| JJ962 | *Bacillus velezensis* | weak | Strong | Terpene | terpene | unknown | ND |
| JJ962 | *Bacillus velezensis* | weak | Strong | Terpene | terpene | unknown | ND |
| JJ962 | *Bacillus velezensis* | weak | Strong | NRPS | NRPS | surfactin | 52 |
| JJ962 | *Bacillus velezensis* | weak | Strong | NRPS | Others | iturin/Bacilomycin L | 86 |
| JJ967 | *Priestia megaterium* | weak | No | Terpene | terpene | carotenoid | 50 |
| JJ967 | *Priestia megaterium* | weak | No | PKS | T3PKS | unknown | ND |
| JJ967 | *Priestia megaterium* | weak | No | Terpene | terpene | unknown | ND |
| JJ967 | *Priestia megaterium* | weak | No | Other | siderophore | unknown | ND |
| JJ967 | *Priestia megaterium* | weak | No | Other | phosphonate | unknown | ND |
| JJ967 | *Priestia megaterium* | weak | No | Terpene | terpene | surfactin | 13 |
| JJ969 | *Bacillus (other)* | no | No | Terpene | terpene | carotenoid | 50 |
| JJ969 | *Bacillus (other)* | no | No | Other | phosphonate | unknown | ND |
| JJ969 | *Bacillus (other)* | no | No | Terpene | terpene | unknown | ND |
| JJ969 | *Bacillus (other)* | no | No | Other | siderophore | unknown | ND |
| JJ969 | *Bacillus (other)* | no | No | PKS | T3PKS | unknown | ND |
| JJ969 | *Bacillus (other)* | no | No | Terpene | terpene | surfactin | 13 |
| JJ973 | *Bacillus pumilus* | weak | Strong | NRPS | NRPS | bacillibactin | 53 |
| JJ973 | *Bacillus pumilus* | weak | Strong | Other | other | bacilysin | 85 |
| JJ973 | *Bacillus pumilus* | weak | Strong | Terpene | terpene | carotenoid | 50 |
| JJ973 | *Bacillus pumilus* | weak | Strong | Other | betalactone | fengycin | 53 |
| JJ973 | *Bacillus pumilus* | weak | Strong | NRPS | NRPS | lichenysin | 50 |
| JJ973 | *Bacillus pumilus* | weak | Strong | PKS | T3PKS | unknown | ND |
| JJ973 | *Bacillus pumilus* | weak | Strong | Other | betalactone | unknown | ND |
| JJ973 | *Bacillus pumilus* | weak | Strong | Terpene | terpene | unknown | ND |
| JJ973 | *Bacillus pumilus* | weak | Strong | RiPP | bacteriocin | unknown | ND |
| JJ973 | *Bacillus pumilus* | weak | Strong | RiPP | head_to_tail | sporulation killing factor | 100 |
| JJ973 | *Bacillus pumilus* | weak | Strong | PKS-NRPS | NRPS | zwittermicin A | 18 |
| JJ975 | *Bacillus subtilis* | strong | Strong | PKS-NRPS | transAT-PKS-like | bacillaene | 100 |
| JJ975 | *Bacillus subtilis* | strong | Strong | NRPS | NRPS | bacillibactin | 100 |
| JJ975 | *Bacillus subtilis* | strong | Strong | Other | other | bacilysin | 100 |
| JJ975 | *Bacillus subtilis* | strong | Strong | NRPS | NRPS | fengycin | 80 |
| JJ975 | *Bacillus subtilis* | strong | Strong | PKS | T3PKS | unknown | ND |
| JJ975 | *Bacillus subtilis* | strong | Strong | Terpene | terpene | unknown | ND |
| JJ975 | *Bacillus subtilis* | strong | Strong | Terpene | terpene | unknown | ND |
| JJ975 | *Bacillus subtilis* | strong | Strong | RiPP | head_to_tail | subtilosin A | 100 |
| JJ975 | *Bacillus subtilis* | strong | Strong | NRPS | NRPS | surfactin | 82 |
| JJ977 | *Bacillus pseudomycoides* | no | No | NRPS | NRPS | cyanopeptin | 75 |
| JJ977 | *Bacillus pseudomycoides* | no | No | Other | betalactone | fengycin | 40 |
| JJ977 | *Bacillus pseudomycoides* | no | No | Terpene | terpene | unknown | ND |
| JJ977 | *Bacillus pseudomycoides* | no | No | NRPS | NRPS | unknown | ND |
| JJ977 | *Bacillus pseudomycoides* | no | No | RiPP | LAP | unknown | ND |
| JJ977 | *Bacillus pseudomycoides* | no | No | RiPP | bacteriocin | unknown | ND |
| JJ977 | *Bacillus pseudomycoides* | no | No | RiPP | lanthipeptide | unknown | ND |
| JJ977 | *Bacillus pseudomycoides* | no | No | RiPP | lassopeptide | paeninodin | 100 |
| JJ978 | *Bacillus velezensis* | weak | Strong | PKS-NRPS | transAT-PKS-like | bacillaene | 100 |
| JJ978 | *Bacillus velezensis* | weak | Strong | NRPS | NRPS | bacillibactin | 100 |
| JJ978 | *Bacillus velezensis* | weak | Strong | Other | other | bacilysin | 100 |
| JJ978 | *Bacillus velezensis* | weak | Strong | PKS | PKS-like | butirosin A | 7 |
| JJ978 | *Bacillus velezensis* | weak | Strong | PKS | transAT-PKS-like | difficidin | 53 |
| JJ978 | *Bacillus velezensis* | weak | Strong | Other | betalactone | fengycin | 80 |
| JJ978 | *Bacillus velezensis* | weak | Strong | PKS | transAT-PKS | macrolactin H | 100 |
| JJ978 | *Bacillus velezensis* | weak | Strong | PKS | T3PKS | unknown | ND |
| JJ978 | *Bacillus velezensis* | weak | Strong | RiPP | thiopeptide | unknown | ND |
| JJ978 | *Bacillus velezensis* | weak | Strong | Terpene | terpene | unknown | ND |
| JJ978 | *Bacillus velezensis* | weak | Strong | Terpene | terpene | unknown | ND |
| JJ978 | *Bacillus velezensis* | weak | Strong | NRPS | NRPS | surfactin | 47 |
| JJ982 | *Bacillus safensis* | no | Strong | NRPS | NRPS | bacillibactin | 53 |
| JJ982 | *Bacillus safensis* | no | Strong | Other | other | bacilysin | 85 |
| JJ982 | *Bacillus safensis* | no | Strong | Other | other | carotenoid | 50 |
| JJ982 | *Bacillus safensis* | no | Strong | Other | betalactone | fengycin | 53 |
| JJ982 | *Bacillus safensis* | no | Strong | NRPS | NRPS | lichenysin | 85 |
| JJ982 | *Bacillus safensis* | no | Strong | PKS | T3PKS | unknown | ND |
| JJ982 | *Bacillus safensis* | no | Strong | Terpene | terpene | unknown | ND |
| JJ982 | *Bacillus safensis* | no | Strong | RiPP | LAP | plantazolicin | 91 |
| JJ987 | *Priestia megaterium* | no | No | Terpene | terpene | carotenoid | 50 |
| JJ987 | *Priestia megaterium* | no | No | PKS | T3PKS | unknown | ND |
| JJ987 | *Priestia megaterium* | no | No | Terpene | terpene | unknown | ND |
| JJ987 | *Priestia megaterium* | no | No | Other | siderophore | unknown | ND |
| JJ987 | *Priestia megaterium* | no | No | Other | siderophore | unknown | ND |
| JJ987 | *Priestia megaterium* | no | No | RiPP | lassopeptide | paeninodin | 60 |
| JJ987 | *Priestia megaterium* | no | No | Terpene | terpene | surfactin | 13 |
| JJ988 | *Bacillus altitudinis* | no | Sparse | Other | other | bacilysin | 85 |
| JJ988 | *Bacillus altitudinis* | no | Sparse | Terpene | terpene | carotenoid | 50 |
| JJ988 | *Bacillus altitudinis* | no | Sparse | Other | betalactone | fengycin | 53 |
| JJ988 | *Bacillus altitudinis* | no | Sparse | NRPS | NRPS | lichenysin | 50 |
| JJ988 | *Bacillus altitudinis* | no | Sparse | Other | betalactone | unknown | ND |
| JJ988 | *Bacillus altitudinis* | no | Sparse | PKS | T3PKS | unknown | ND |
| JJ988 | *Bacillus altitudinis* | no | Sparse | Terpene | terpene | unknown | ND |
| JJ988 | *Bacillus altitudinis* | no | Sparse | Other | siderophore | unknown | ND |
| JJ988 | *Bacillus altitudinis* | no | Sparse | RiPP | bacteriocin | unknown | ND |
| JJ991 | *Bacillus velezensis* | weak | Strong | PKS-NRPS | transAT-PKS | bacillaene | 71 |
| JJ991 | *Bacillus velezensis* | weak | Strong | NRPS | NRPS | bacillibactin | 100 |
| JJ991 | *Bacillus velezensis* | weak | Strong | Other | other | bacilysin | 100 |
| JJ991 | *Bacillus velezensis* | weak | Strong | PKS | PKS-like | butirosin A | 7 |
| JJ991 | *Bacillus velezensis* | weak | Strong | PKS | transAT-PKS | difficidin | 40 |
| JJ991 | *Bacillus velezensis* | weak | Strong | NRPS | NRPS | fengycin | 100 |
| JJ991 | *Bacillus velezensis* | weak | Strong | PKS | transAT-PKS | macrolactin H | 100 |
| JJ991 | *Bacillus velezensis* | weak | Strong | PKS | T3PKS | unknown | ND |
| JJ991 | *Bacillus velezensis* | weak | Strong | RiPP | lanthipeptide | unknown | ND |
| JJ991 | *Bacillus velezensis* | weak | Strong | Terpene | terpene | unknown | ND |
| JJ991 | *Bacillus velezensis* | weak | Strong | Terpene | terpene | unknown | ND |
| JJ991 | *Bacillus velezensis* | weak | Strong | NRPS | NRPS | surfactin | 82 |
| JJ991 | *Bacillus velezensis* | weak | Strong | NRPS | Others | iturin/Bacilomycin L | 100 |
| JM199 | *Bacillus velezensis* | weak | Strong | RiPP | lanthipeptide-class-ii | unknown | ND |
| JM199 | *Bacillus velezensis* | weak | Strong | NRPS | NRPS | rhizomide A | 22 |
| JM199 | *Bacillus velezensis* | weak | Strong | NRPS | NRPS | surfactin | 91 |
| JM199 | *Bacillus velezensis* | weak | Strong | PKS | PKS-like | butirosin A | 7 |
| JM199 | *Bacillus velezensis* | weak | Strong | Terpene | terpene | unknown | ND |
| JM199 | *Bacillus velezensis* | weak | Strong | PKS | transAT-PKS | macrolactin H | 100 |
| JM199 | *Bacillus velezensis* | weak | Strong | PKS-NRPS | transAT-PKS | bacillaene | 100 |
| JM199 | *Bacillus velezensis* | weak | Strong | NRPS | NRPS | fengycin | 100 |
| JM199 | *Bacillus velezensis* | weak | Strong | Terpene | terpene | unknown | ND |
| JM199 | *Bacillus velezensis* | weak | Strong | PKS | T3PKS | unknown | ND |
| JM199 | *Bacillus velezensis* | weak | Strong | PKS | transAT-PKS | difficidin | 100 |
| JM199 | *Bacillus velezensis* | weak | Strong | NRPS | NRPS | bacillibactin | 100 |
| JM199 | *Bacillus velezensis* | weak | Strong | RiPP | lanthipeptide-class-i | subtilin | 100 |
| JM199 | *Bacillus velezensis* | weak | Strong | Other | other | bacilysin | 100 |
| JM199 | *Bacillus velezensis* | weak | Strong | NRPS | Others | iturin/Bacilomycin L | 100 |
| JM204 | *Bacillus velezensis* | strong | Strong | RiPP | thiopeptide | unknown | ND |
| JM204 | *Bacillus velezensis* | strong | Strong | NRPS | NRPS | surfactin | 91 |
| JM204 | *Bacillus velezensis* | strong | Strong | PKS | PKS-like | butirosin A | 7 |
| JM204 | *Bacillus velezensis* | strong | Strong | Terpene | terpene | unknown | ND |
| JM204 | *Bacillus velezensis* | strong | Strong | PKS | transAT-PKS | macrolactin H | 100 |
| JM204 | *Bacillus velezensis* | strong | Strong | PKS-NRPS | transAT-PKS | bacillaene | 100 |
| JM204 | *Bacillus velezensis* | strong | Strong | NRPS | NRPS | fengycin | 100 |
| JM204 | *Bacillus velezensis* | strong | Strong | Terpene | terpene | unknown | ND |
| JM204 | *Bacillus velezensis* | strong | Strong | PKS | T3PKS | unknown | ND |
| JM204 | *Bacillus velezensis* | strong | Strong | PKS | transAT-PKS | difficidin | 100 |
| JM204 | *Bacillus velezensis* | strong | Strong | NRPS | NRPS | bacillibactin | 100 |
| JM204 | *Bacillus velezensis* | strong | Strong | Other | other | bacilysin | 100 |
| JM204 | *Bacillus velezensis* | strong | Strong | NRPS | Others | iturin/Bacilomycin L | 100 |
| JM236 | *Bacillus velezensis* | strong | Strong | NRPS | NRPS | surfactin | 91 |
| JM236 | *Bacillus velezensis* | strong | Strong | RiPP | RRE-containing | plantazolicin | 91 |
| JM236 | *Bacillus velezensis* | strong | Strong | PKS | PKS-like | butirosin A | 7 |
| JM236 | *Bacillus velezensis* | strong | Strong | Terpene | terpene | unknown | ND |
| JM236 | *Bacillus velezensis* | strong | Strong | PKS | transAT-PKS | macrolactin H | 100 |
| JM236 | *Bacillus velezensis* | strong | Strong | PKS-NRPS | transAT-PKS | bacillaene | 100 |
| JM236 | *Bacillus velezensis* | strong | Strong | NRPS | NRPS | fengycin | 100 |
| JM236 | *Bacillus velezensis* | strong | Strong | Terpene | terpene | unknown | ND |
| JM236 | *Bacillus velezensis* | strong | Strong | PKS | T3PKS | unknown | ND |
| JM236 | *Bacillus velezensis* | strong | Strong | PKS | transAT-PKS | difficidin | 100 |
| JM236 | *Bacillus velezensis* | strong | Strong | NRPS | NRPS | bacillibactin | 100 |
| JM236 | *Bacillus velezensis* | strong | Strong | Other | other | bacilysin | 100 |
| JM236 | *Bacillus velezensis* | strong | Strong | RiPP | lanthipeptide-class-ii | mersacidin | 100 |
| JM236 | *Bacillus velezensis* | strong | Strong | NRPS | Others | iturin/Bacilomycin L | 100 |
| JM553 | *Bacillus subtilis* | strong | Strong | NRPS | NRPS | surfactin | 78 |
| JM553 | *Bacillus subtilis* | strong | Strong | Terpene | terpene | unknown | ND |
| JM553 | *Bacillus subtilis* | strong | Strong | PKS-NRPS | transAT-PKS | bacillaene | 100 |
| JM553 | *Bacillus subtilis* | strong | Strong | NRPS | NRPS | fengycin | 100 |
| JM553 | *Bacillus subtilis* | strong | Strong | Terpene | terpene | unknown | ND |
| JM553 | *Bacillus subtilis* | strong | Strong | PKS | T3PKS | unknown | ND |
| JM553 | *Bacillus subtilis* | strong | Strong | NRPS | NRPS | bacillibactin | 100 |
| JM553 | *Bacillus subtilis* | strong | Strong | RiPP | sactipeptide | subtilosin A | 100 |
| JM553 | *Bacillus subtilis* | strong | Strong | Other | other | bacilysin | 100 |
| JM553 | *Bacillus subtilis* | strong | Strong | Other | epipeptide | thailanstatin A | 10 |
| JM907 | *Bacillus subtilis* | strong | Strong | RiPP | thiopeptide | unknown | ND |
| JM907 | *Bacillus subtilis* | strong | Strong | NRPS | NRPS | surfactin | 91 |
| JM907 | *Bacillus subtilis* | strong | Strong | PKS | PKS-like | butirosin A | 7 |
| JM907 | *Bacillus subtilis* | strong | Strong | Terpene | terpene | unknown | ND |
| JM907 | *Bacillus subtilis* | strong | Strong | PKS | transAT-PKS | macrolactin H | 100 |
| JM907 | *Bacillus subtilis* | strong | Strong | PKS-NRPS | transAT-PKS | bacillaene | 100 |
| JM907 | *Bacillus subtilis* | strong | Strong | NRPS | NRPS | fengycin | 100 |
| JM907 | *Bacillus subtilis* | strong | Strong | Terpene | terpene | unknown | ND |
| JM907 | *Bacillus subtilis* | strong | Strong | PKS | T3PKS | unknown | ND |
| JM907 | *Bacillus subtilis* | strong | Strong | PKS | transAT-PKS | difficidin | 100 |
| JM907 | *Bacillus subtilis* | strong | Strong | NRPS | NRPS | bacillibactin | 100 |
| JM907 | *Bacillus subtilis* | strong | Strong | Other | other | bacilysin | 100 |
| JM907 | *Bacillus velezensis* | strong | Strong | NRPS | Others | iturin/Bacilomycin L | 100 |
