# Supplementary material for: Broad antibiosis activity of Bacillus velezensis and Bacillus subtilis is accounted for by a conserved capacity for lipopeptide biosynthesis
Source: Front Microbiol. 2025 Aug 29;16:1636481. doi: 10.3389/fmicb.2025.1636481 (PMC12426035; doi:10.3389/fmicb.2025.1636481)
Supplement: Supplementary file 4 [file Table_4.docx]

Supplementary Material

**Broad Antibiosis Activity of *Bacillus velezensis* and *Bacillus subtilis* is Accounted for by a Conserved Capacity for Lipopeptide Biosynthesis**

**Jahangir Alam,^1,4^ Oluwakemisola E. Olofintila,^2^ Francesco S. Moen,^3^ Zachary A. Noel,^2^ Mark R. Liles,^3^ Douglas C. Goodwin^1,*^**

^1^Department of Chemistry and Biochemistry, Auburn University, Auburn, AL 36849

^2^Department of Entomology and Plant Pathology, Auburn University, Auburn, AL 36849

^3^Department of Biological Sciences, Auburn University, Auburn, AL 36849

^4^Current address: Organon & Co., 727 Norristown Road, Building 4, Lower Gwynedd, PA, 19002

*** Correspondence:** Douglas C. Goodwin: goodwdc@auburn.edu

***Supplementary Material Contents***

***2-Supplementary Material Table S1 (Separate File)***

Table S1. Bacillaceae PGPR strains evaluated for biocontrol ability.

***3-Supplementary Material Figures S1 - S6 (Separate File)***

Figure S1. A representative assay plate showing antibiosis against *P. nicotianae*.

Figure S2. Correlations of bioactivity index and the number of BGCs from each of six major classes.

Figure S3. Clustering analysis of all 2,446 BGCs identified from the genomes of 284 Bacillaceae strains.

Figure S4. Plate-based evaluation of antibiosis exerted by total extracts of *B. velezensis* JJ334 against *P. nicotianae* and fungal pathogens.

Figure S5. Separation and spectral properties of total extracts from representative bioactive *Bacillus* species.

Figure S6. Plate-based evaluation of antibiosis exerted by isolated lipopeptides against *P. nicotianae* and fungal pathogens.

***4-Supplementary Material Table S2 (Separate File)***

Table S2. Detailed information on BGCs/secondary metabolites from Bacillaceae strains: Strain ID, BGCs, secondary metabolites, and inhibition against *Phytophthora nicotianae.*

***5-Supplementary Material Mass Spec (This File)***

Table S3 and Figure S7. Assignment of fengycin derivatives based on representative fragment ions generated by MS^2^ analyses.

Table S4 and Figure S8. Assignment of surfactin derivatives based on representative fragment ions generated by MS^2^ analyses.

Table S5 and Figure S9. Assignment of bacillomycin L derivatives based on representative fragment ions generated by MS^2^ analyses.

**Table S3**. Fengycin ions generated by MS and MS^2^ were used for unequivocal assignment of fengycin derivatives.

| **Precursor ion (MH^+^)** | **Diagnostic**  **ion (A/B)** | **Diagnostic Ion (C)** | **Fengycin**  **Derivative** | **aa position 6** | **aa position 10** |
| --- | --- | --- | --- | --- | --- |
| 1435.7730 | 966.4499/1080.5294 | 356.2407 | C14 fengycin A | Ala | Ile |
| 1449.7878 | 966.4497/1080.5299 | 370.2564 | C15 fengycin A | Ala | Ile |
|  | 952.4338/1066.5136 | 384.2717 | C16 fengycin A2 | Ala | Val |
| 1463.8028 | 966.4498/1080.5306 | 384.2700 | C16 fengycin A | Ala | Ile |
|  | 952.4378/1066.5146 | 398.2852 | C17 fengycin A2 | Ala | Val |
| 1477.8185 | 966.4502/1080.5254 | 398.2860 | C17 fengycin A | Ala | Ile |
| 1491.8332 | 994.4809/1108.5593 | 384.2726 | C16 fengycin B | Val | Ile |
|  | 980.4677/1094.5444 | 398.2726 | C17 fengycin B2 | Val | Val |
| 1505.8471 | 994.4833/1108.5606 | 398.2873 | C17 fengycin B | Val | Ile |
| 1519.8666 | 994.4780/1108.5611 | 412.3039 | C18 fengycin B | Val | Ile |
| 1533.8819 | 980.4987/1094.5770 | ND^1^ | C20 fengycin B2 | Val | Val |
| 1547.8966 | 980.4988/1094.5783 | ND^1^ | C21 fengycin B2 | Val | Val |
| 1561.9106 | ND^1^ | | | | |

^1^ND=Not Detected.


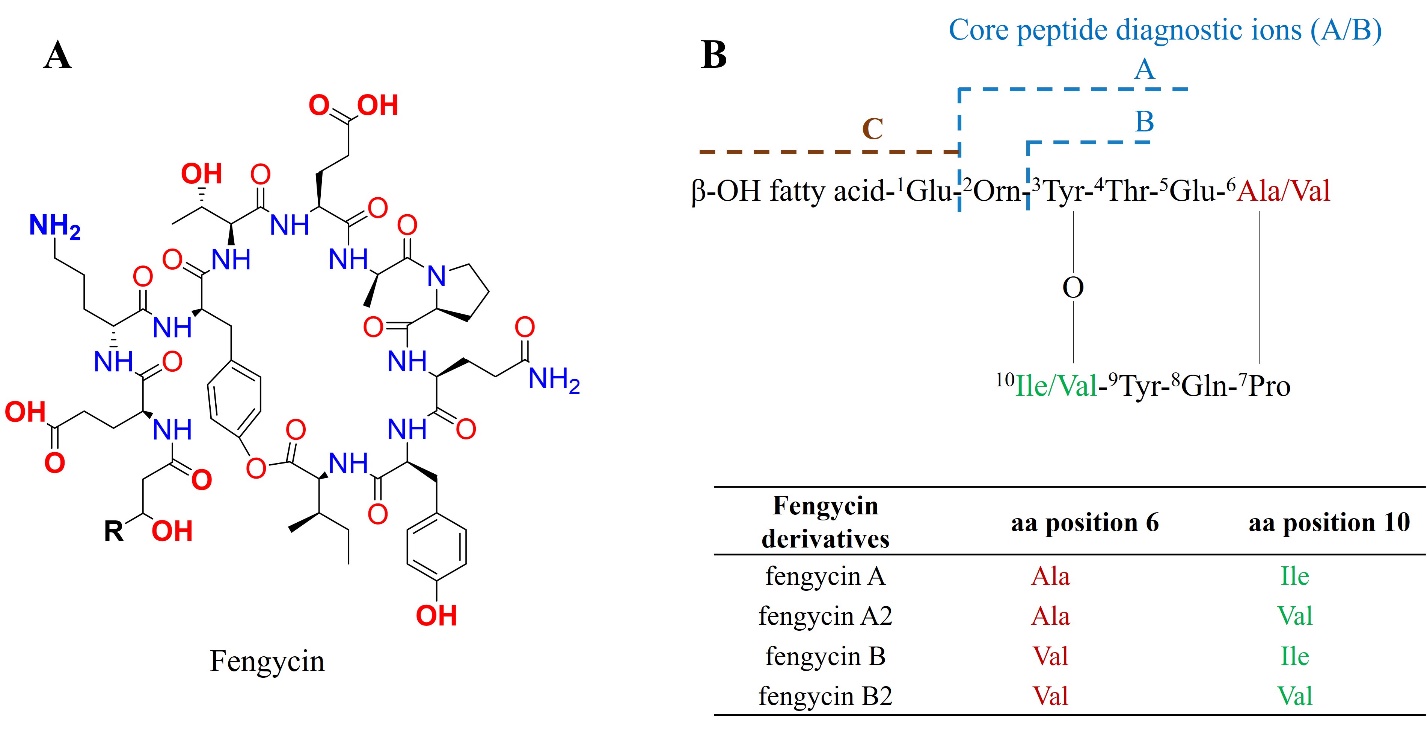


**C**

**MS^2^ of C14 fengycin A ([M+ H]^+^ = 1435.7730) (*B. velezensis* JJ334)**


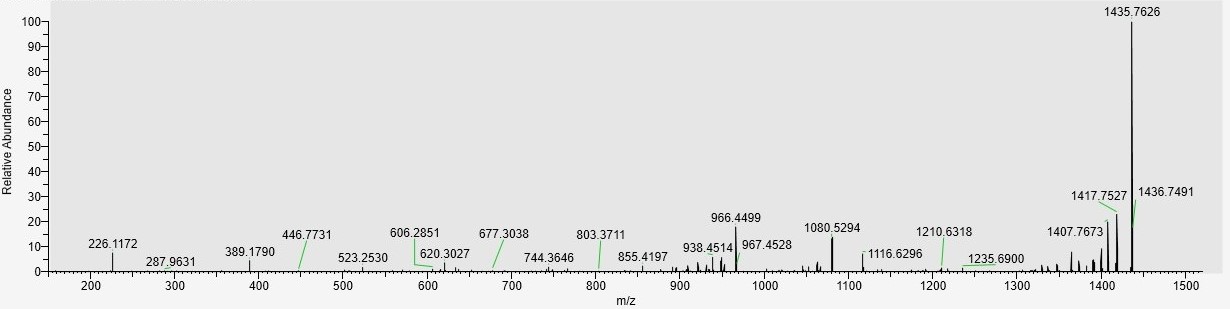

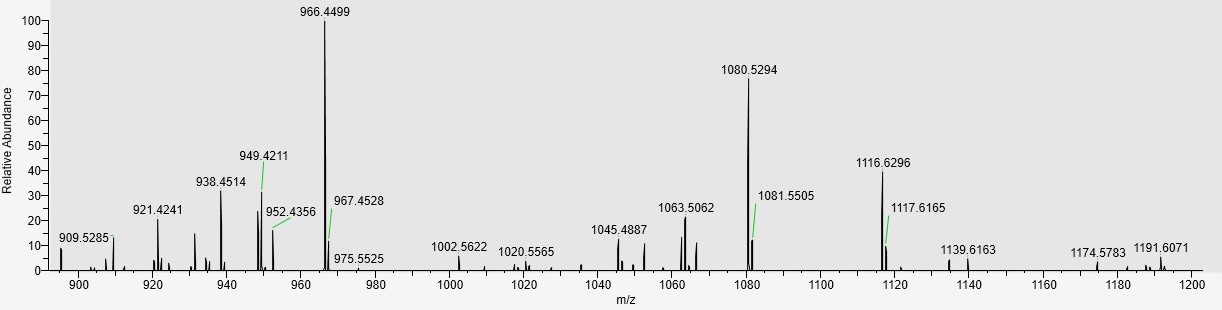


**MS^2^ of C15 fengycin A/ C16 of fengycin A2 ([M+ H]^+^ = 1449.5539) (*B. velezensis* JJ334)**


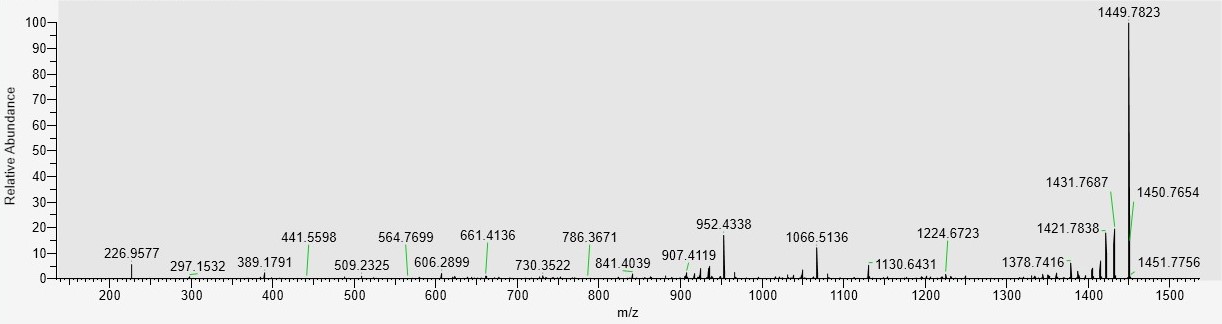

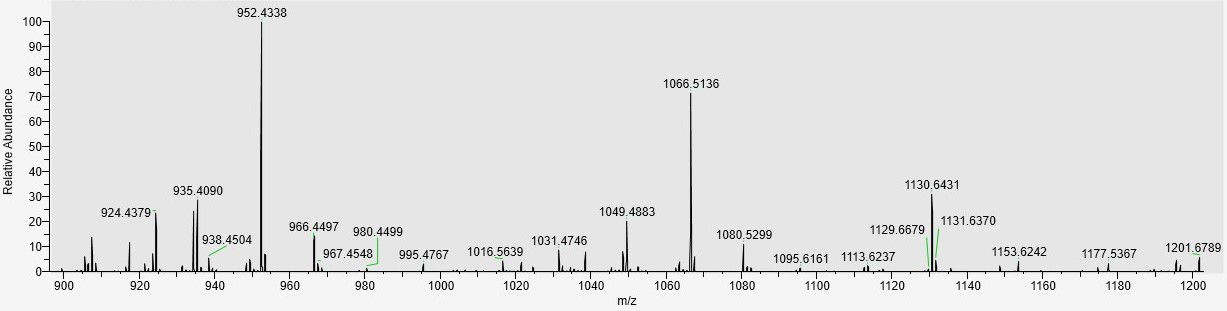


**MS^2^ of C16 fengycin A/ C17 of fengycin A2 ([M+ H]^+^ = 1463.8028) (*B. velezensis* JJ334)**


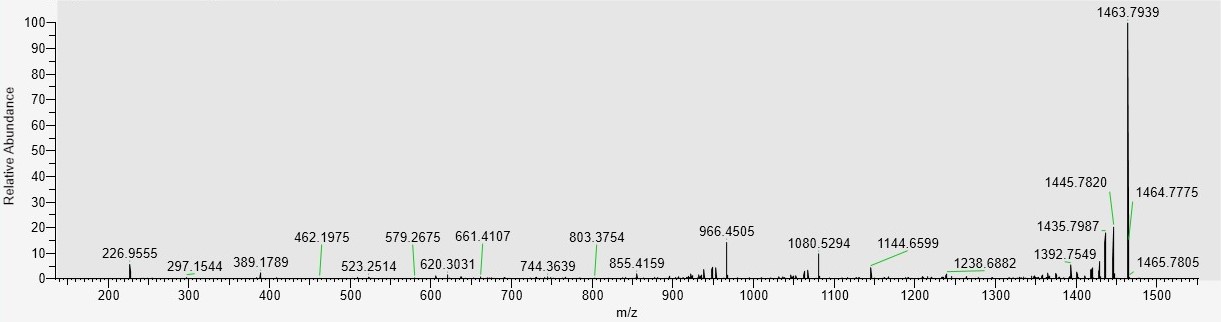

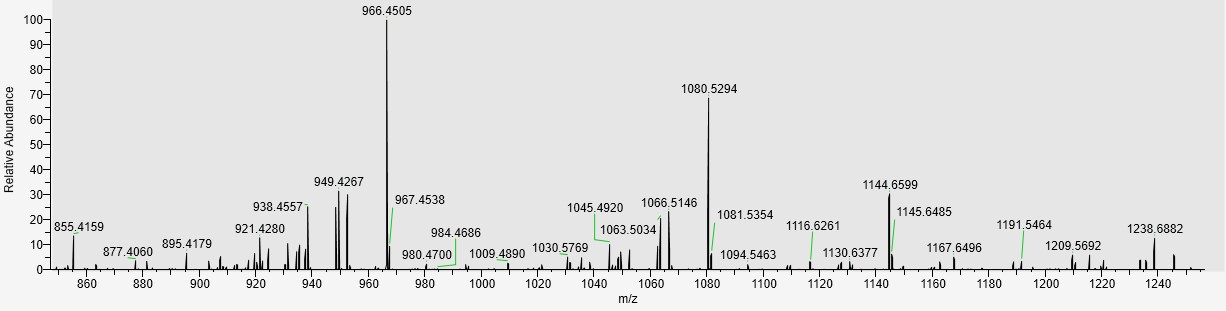


952.4378

**MS^2^ of C17 fengycin A ([M+ H]^+^ = 1477.8185) (*B. velezensis* JJ334)**


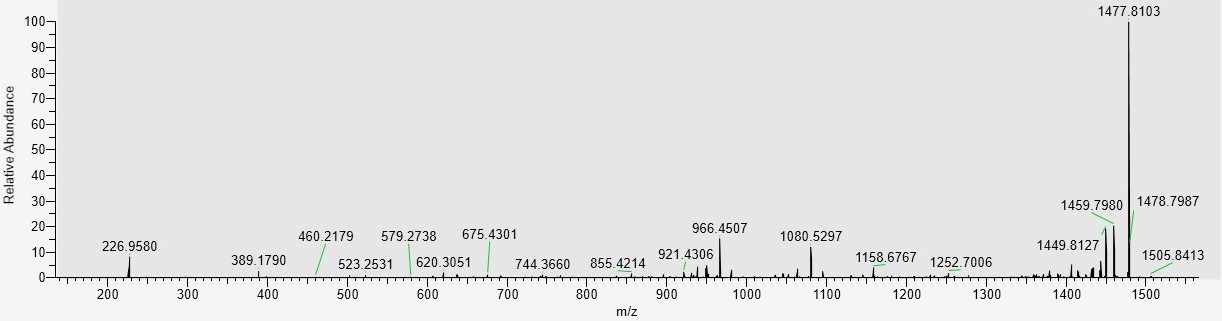

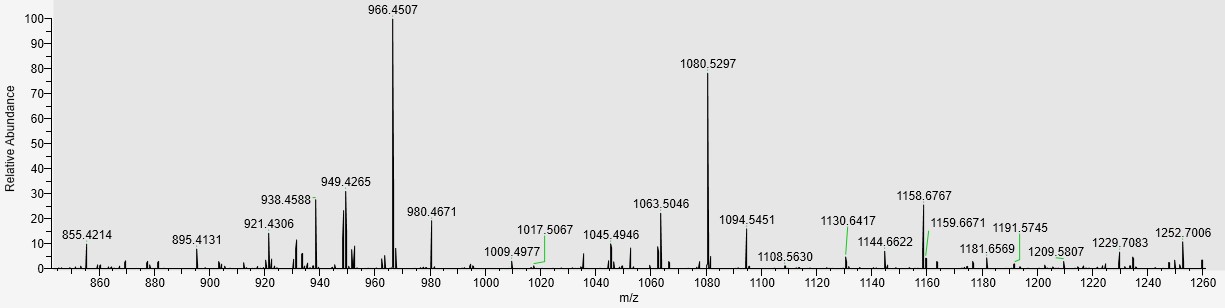


**MS^2^ of C16 fengycin B/ C17 of fengycin B2 ([M+ H]^+^ = 1491.8332) (*B. velezensis* JJ334)**


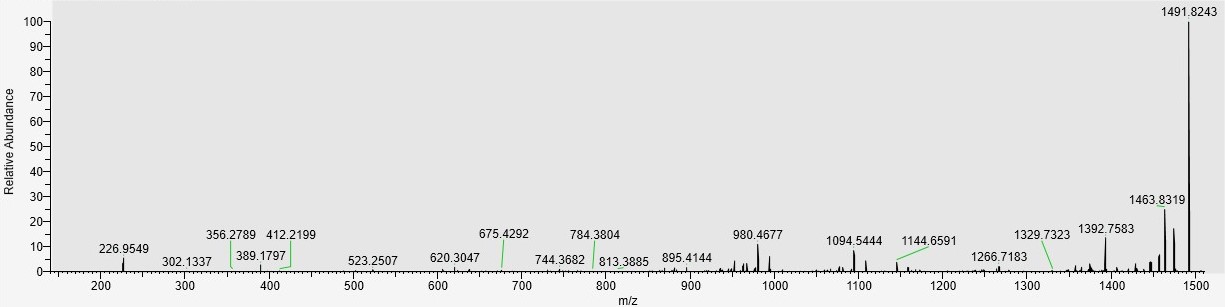

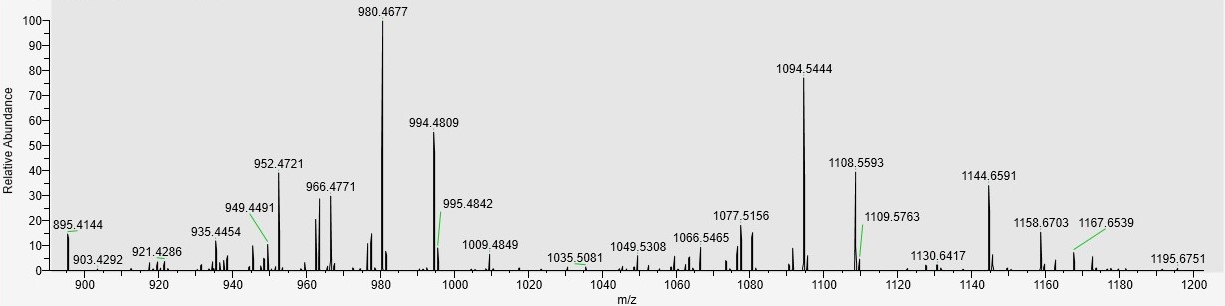


**MS^2^ of C17 fengycin B ([M+ H]^+^ = 1505.8471) (*B. velezensis* JJ334)**


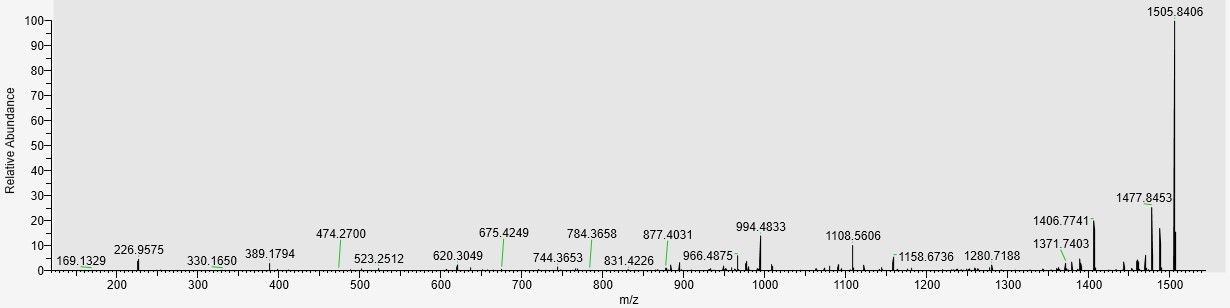

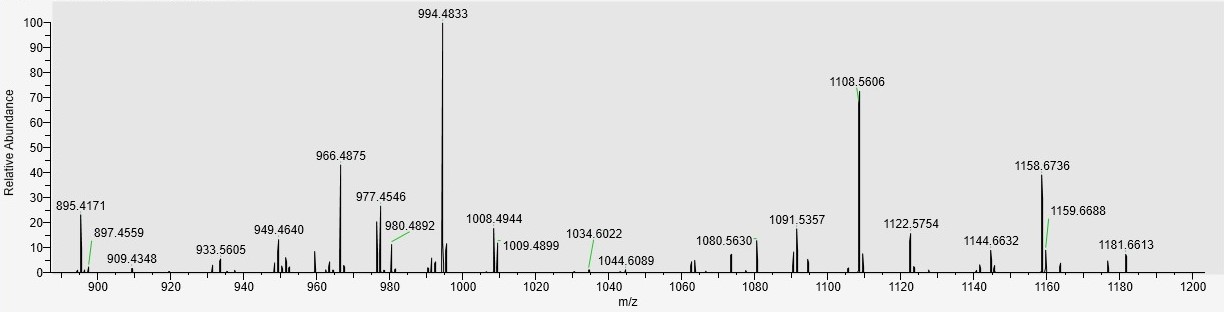


**MS^2^ of fengycin B C18 ([M+ H]^+^ = 1519.8666) (*B. velezensis* JJ334)**


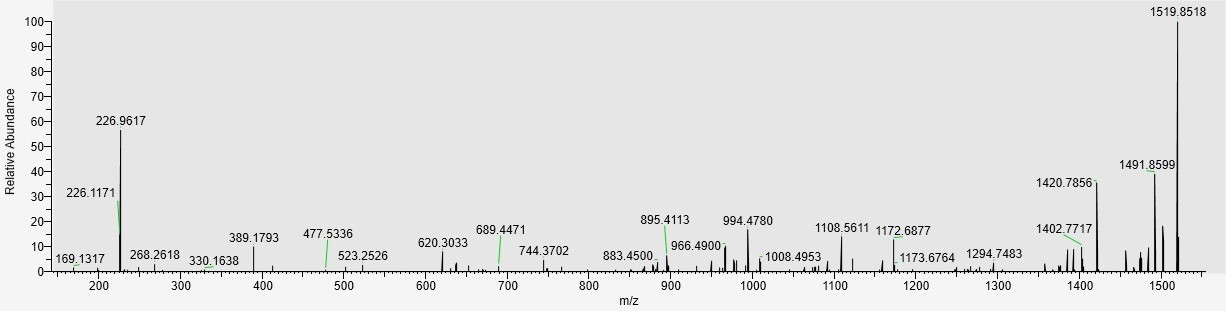

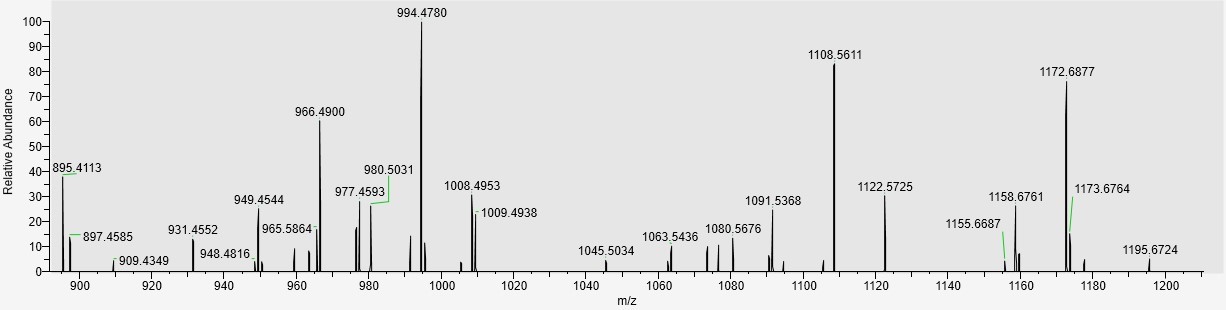


**MS^2^ of C20 fengycin B2 ([M+ H]^+^ = 1533.8819) (*B. velezensis* JJ334)**


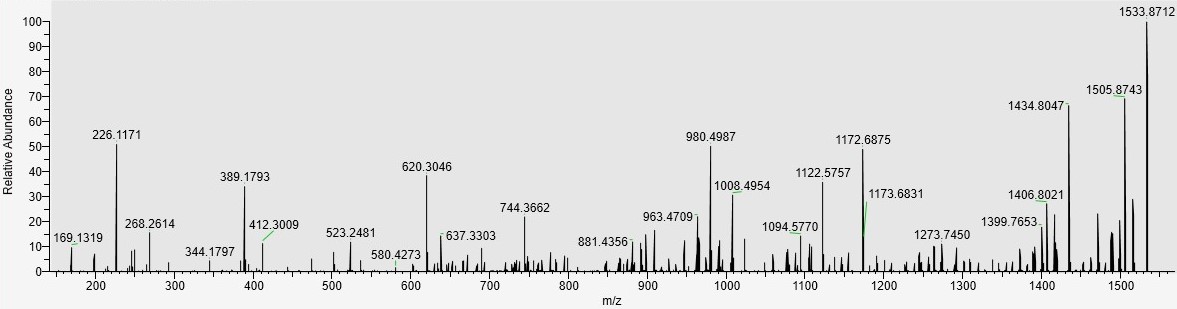

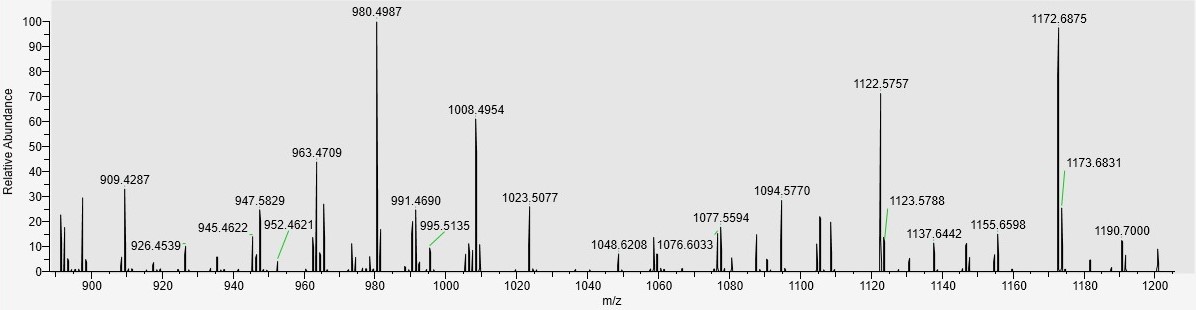


**MS^2^ of C21 ([M+ H]^+^ = 1547.8966) fengycin B2 (*B. velezensis* JJ334)**


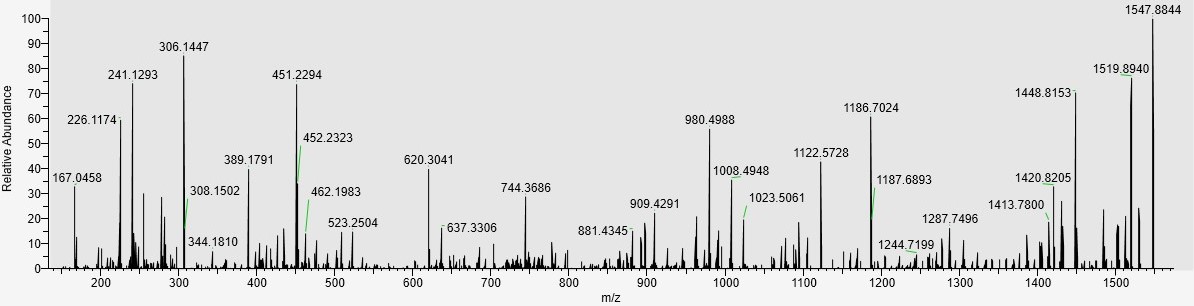

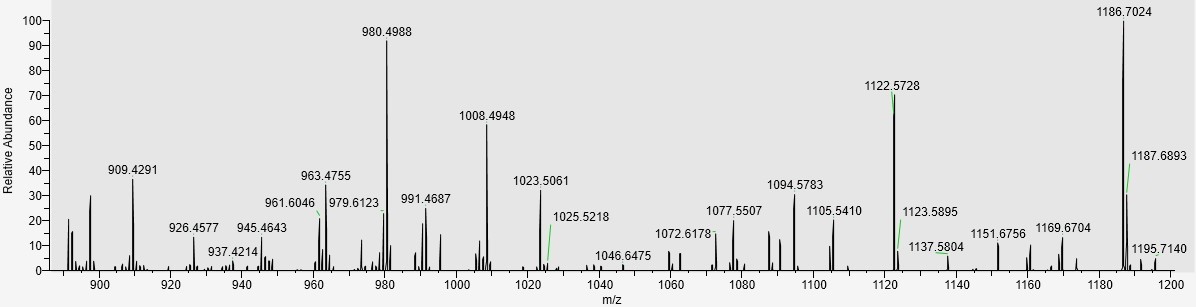


**Figure S7.** The structure of fengycin (A), ions generated from diagnostic fragmentation of the core peptide (B), and MS^2^ spectra corresponding to identified fengycin derivatives (C). MS^2^ spectra of nine prominent fengycin derivatives are shown. These were obtained from total extracts of *B. velezensis* JJ334. These spectra were generated by a multi-stage MS^n^ analyzer from respective precursor ions (positive ionization mode). Note that MS^2^ is not available for the fengycin derivative 1561.9106. Assignment of fengycin derivatives based on change of aa at position 6^th^ and 10^th^ in the core peptide. Commonly observed diagnostic ions generated by MS^2^ of precursor ion were used for assigning the fengycin derivatives. The table shows the assignment of fengycin derivatives based on the variation of aa in the core peptide.

**Table S4**. Ions from MS and MS^2^ spectra used for assignment of surfactin derivatives.

| **Precursor ion (MH^+^)** | **Fragment**  **ion (I)** | **Fragment**  **Ion (II)** | **Surfactin derivative** |
| --- | --- | --- | --- |
| 994.6428 | 685.4447 | 310.1996 | C12 |
| 1008.6587 | 685.4458 | 324.2162 | C13 |
| 1022.6740 | 685.4451 | 338.2312 | C14 |
| 1036.6898 | 685.4443 | 352.2463 | C15 |
| 1050.7053 | 685.4447 | 366.2615 | C16 |
| 1064.7199 | 685.4449 | 380.2765 | C17 |
| 1078.7360 | 685.4509 | 394.2923 | C18 |
| 1092.7519 | ND^1^ | ND^1^ | C19 |

^1^ND=Not Detected.


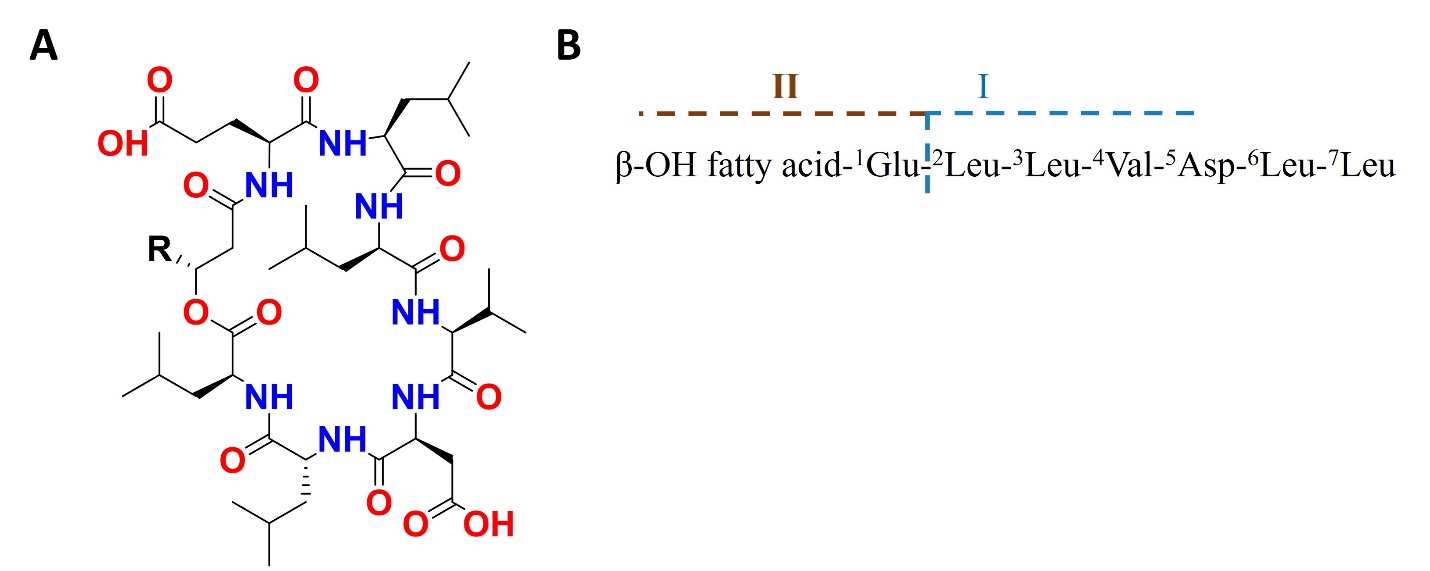


**C**

**MS^2^ of C12 surfactin ([M+ H]^+^ = 994.6428) (*B. velezensis* JJ334)**


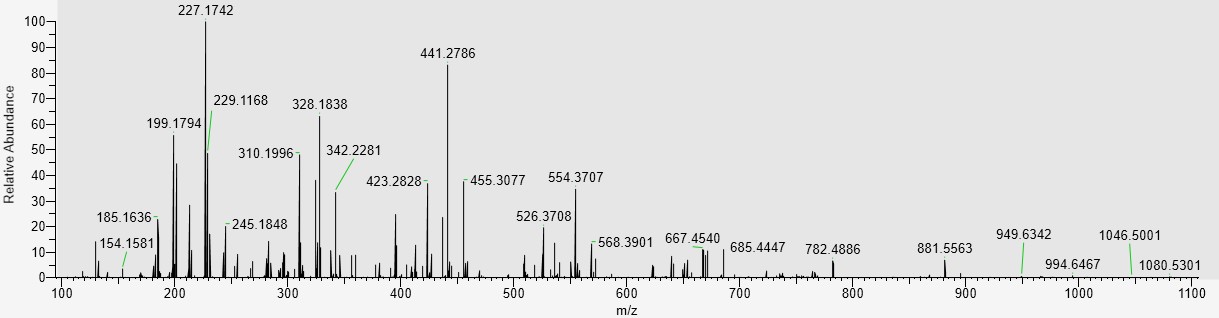

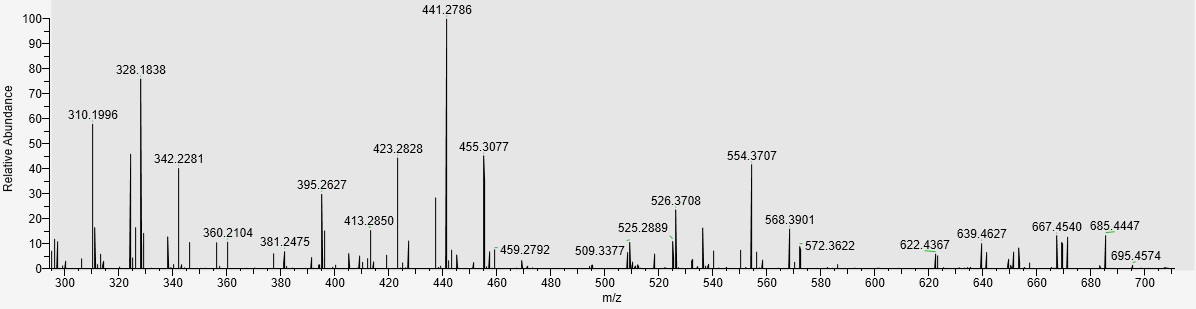


**MS^2^ of C13 surfactin ([M+ H]^+^ = 1008.6587) (*B. velezensis* JJ334)**


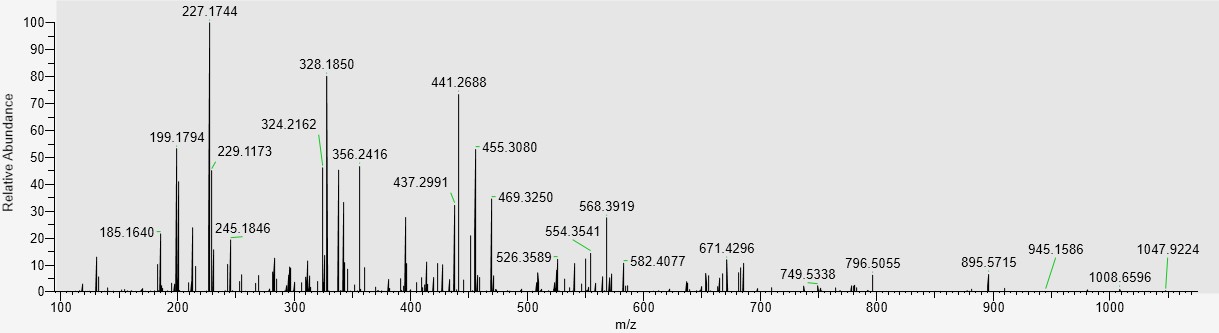

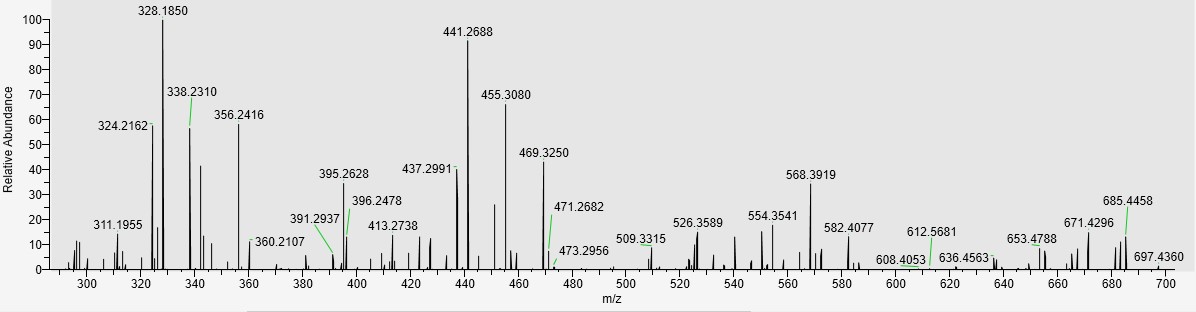


**MS^2^ of C14 surfactin ([M+ H]^+^ = 1022.6740) (*B. velezensis* JJ334)**


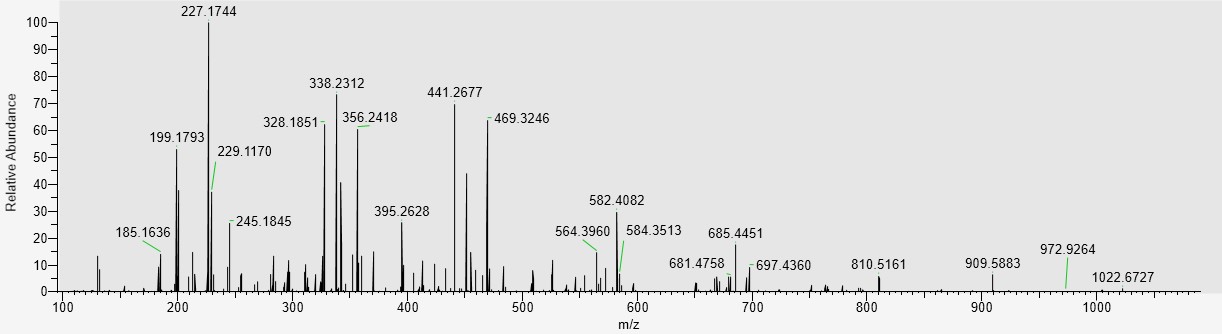

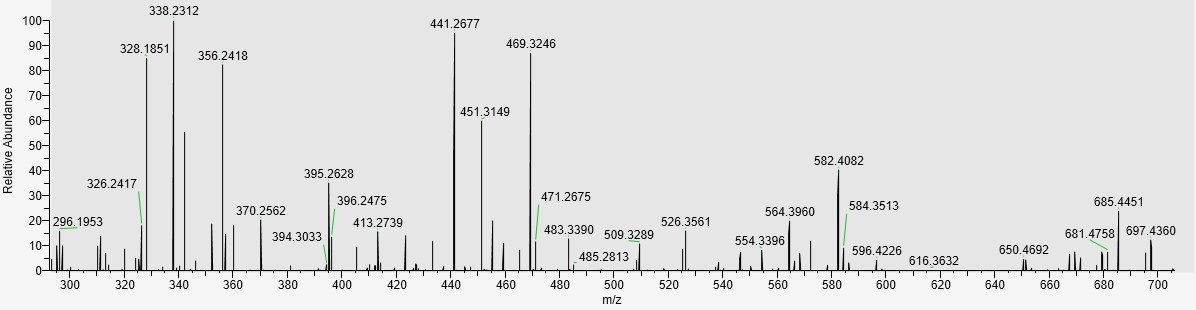


**MS^2^ of C15 surfactin ([M+ H]^+^ = 1036.6898) (*B. velezensis* JJ334)**


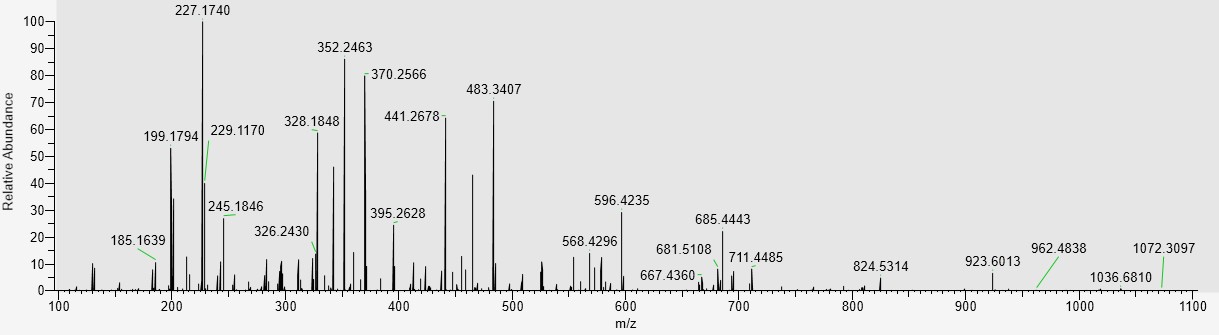

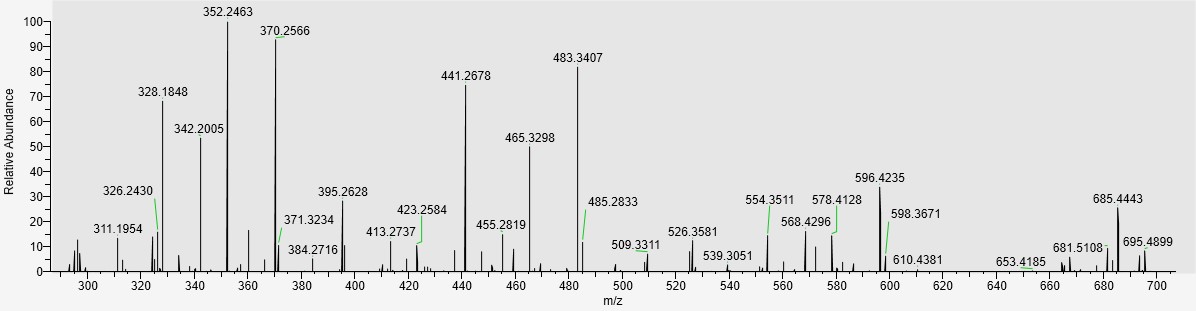


**MS^2^ of C16 surfactin ([M+ H]^+^ = 1050.7053) (*B. velezensis* JJ334)**


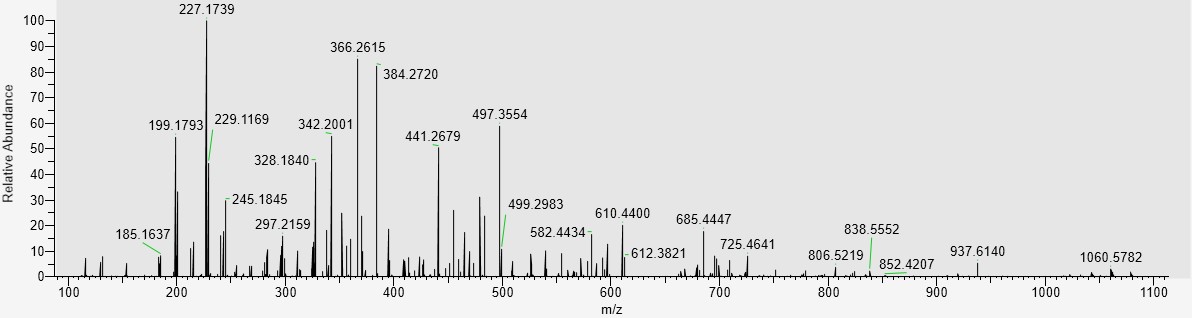

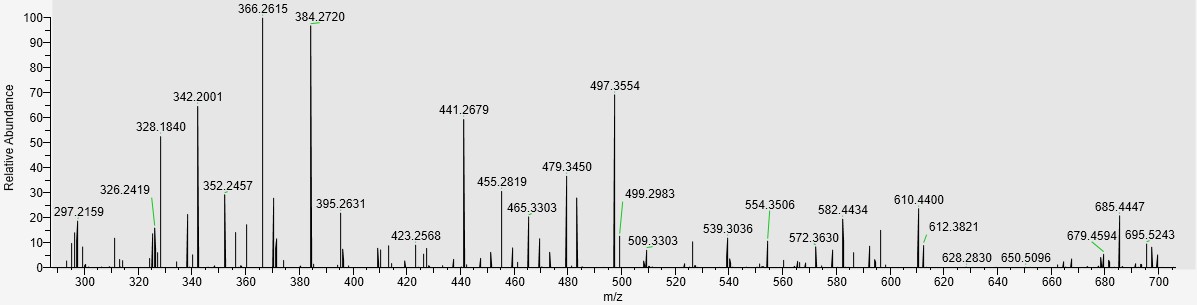


**MS^2^ of C17 surfactin ([M+ H]^+^ = 1064.7199) (*B. velezensis* JJ334)**


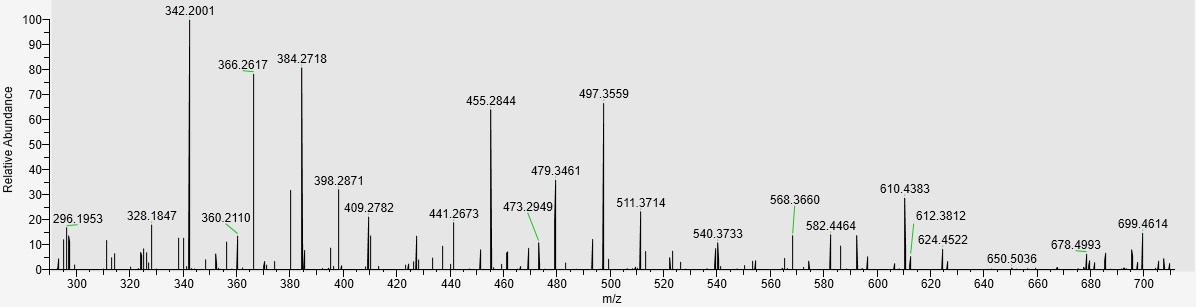

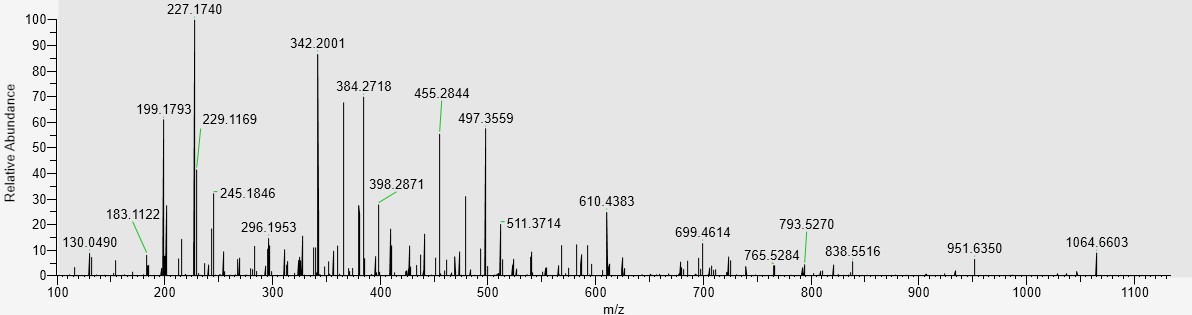


380.2765

685.4449

**MS^2^ of C18 surfactin ([M+ H]^+^ = 1078.7360) (*B. velezensis* JJ334)**


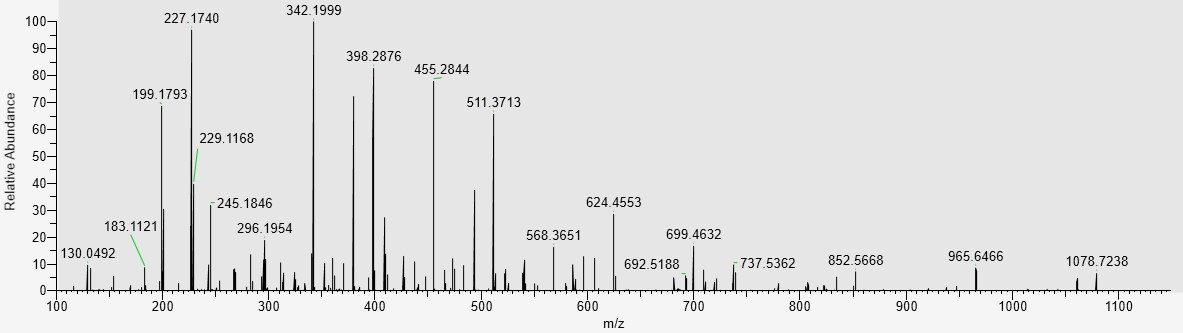

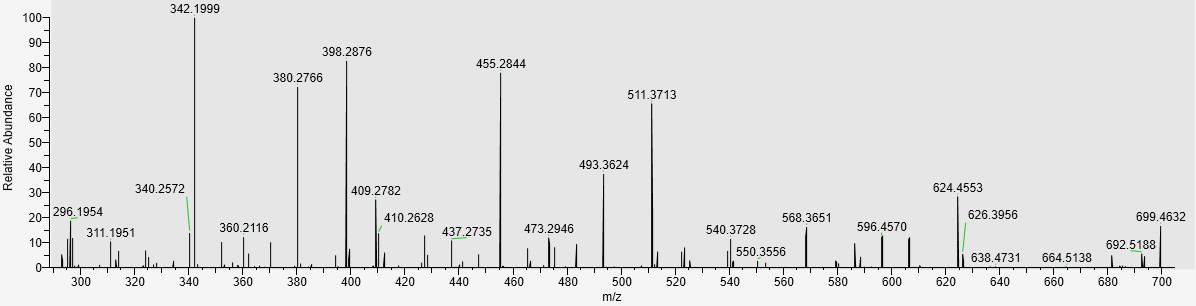


394.2923

685.4449

**Figure S8.** The structure of surfactin (A), ions generated from diagnostic fragmentation of the core peptide (B), and MS^2^ spectra corresponding to identified surfactin derivatives (C). MS^2^ spectra of seven prominent surfactin derivatives were identified from the total extract of *B. velezensis* JJ334. Assignment of surfactin derivatives was based on the cleavage of the Glu1-(Leu/Ile)2 bond, generating the bulk of the core peptide (Leu/Ile2 – Leu7) and the remaining Glu1-FA tail. These spectra were generated by a multi-stage MS^n^ analyzer from respective precursor ions (positive ionization mode). Note that MS^2^ data are not available for the surfactin derivative 1092.9519.

**Table S5**. Ions from MS and MS^2^ spectra used for assignment of bacillomycin L derivatives.

| **Precursor ion (MH^+^)** | **Fragment ion (I)** | **Fragment ion (II)** | **Fragment ion (III)** | **Fragment ion (IV)** | **Bacillomycin Derivative** |
| --- | --- | --- | --- | --- | --- |
| 1007.5044 | 392.1530 | 278.1116 | 170.1891 | 313.2468 | C13 |
| 1021.5198 | 392.1542 | 278.1115 | 184.2046 | 327.2621 | C14 |
| 1035.5354 | 392.1525 | 278.1119 | 198.2205 | 341.2778 | C15 |
| 1049.5539 | 392.1549 | 278.1120 | 212.2361 | 355.2938 | C16 |
| 1063.5666 | 392.1544 | 278.1117 | 226.2516 | 369.3085 | C17 |
| 1077.5820 | ND^1^ | | | | |

^1^ND=Not Detected.


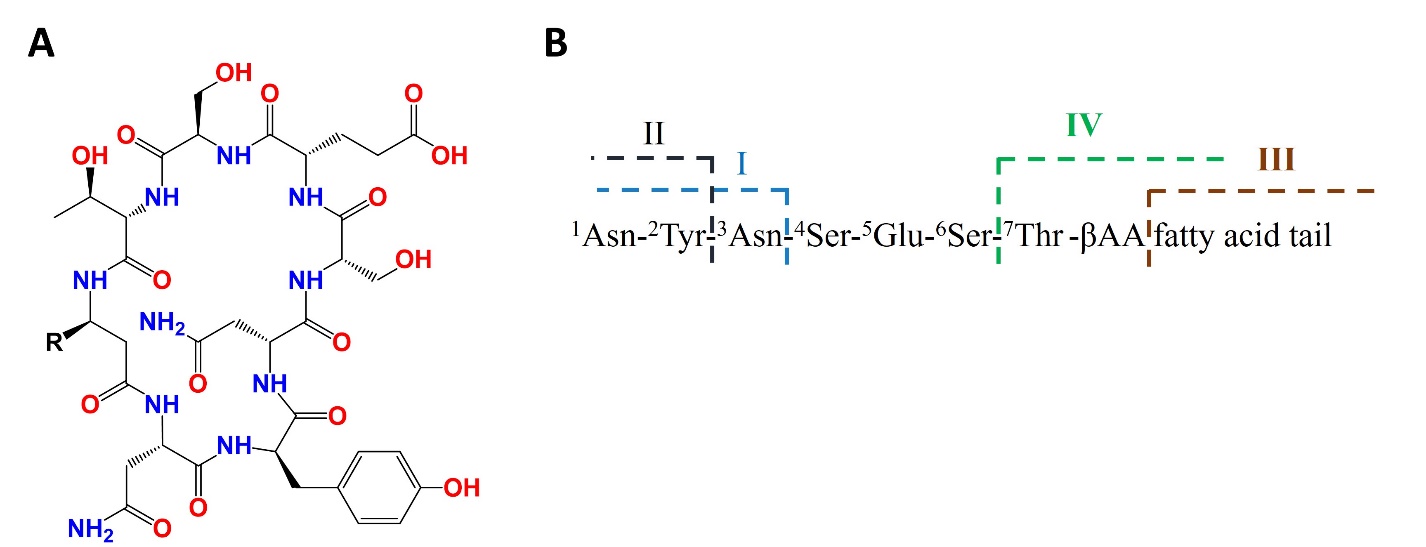


**C**

**MS^2^ of C13 bacillomycin L ([M+ H]^+^ = 1007.5044) (*B. velezensis* JJ334)**


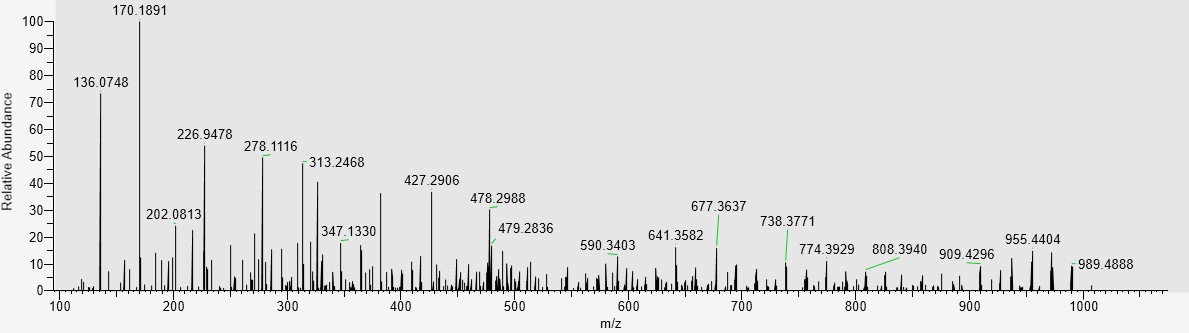

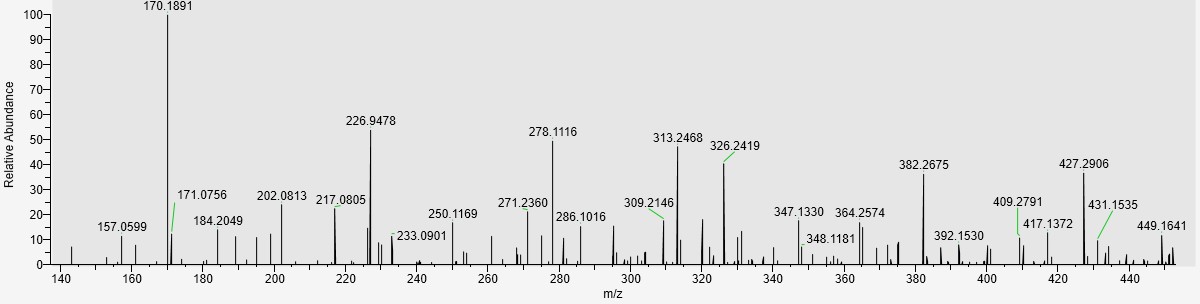


**MS^2^ of C14 bacillomycin L ([M+ H]^+^ = 1021.5188) (*B. velezensis* JJ334)**


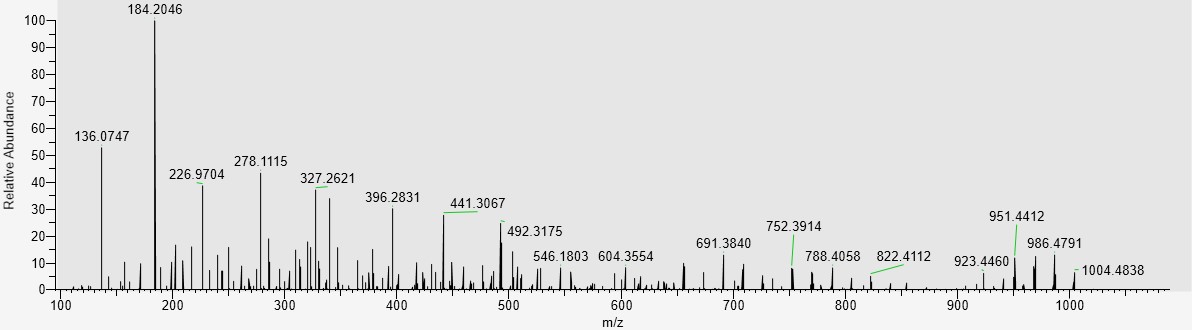

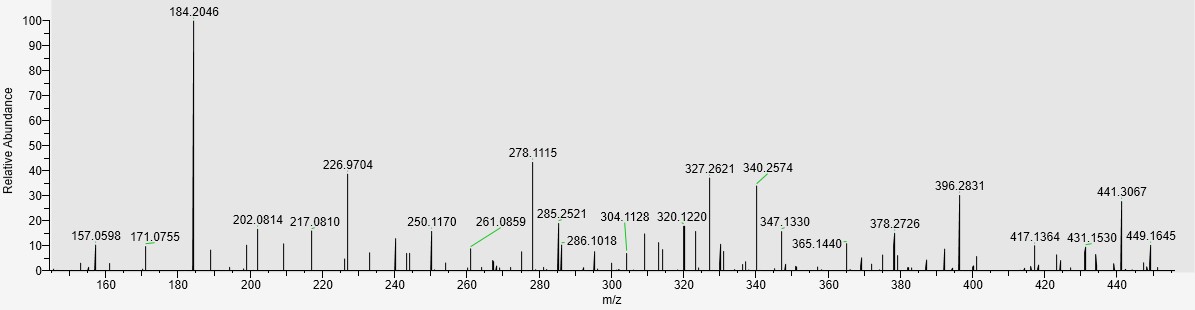


**MS^2^ of C15 bacillomycin L ([M+ H]^+^ = 1035.7730) (*B. velezensis* JJ334)**


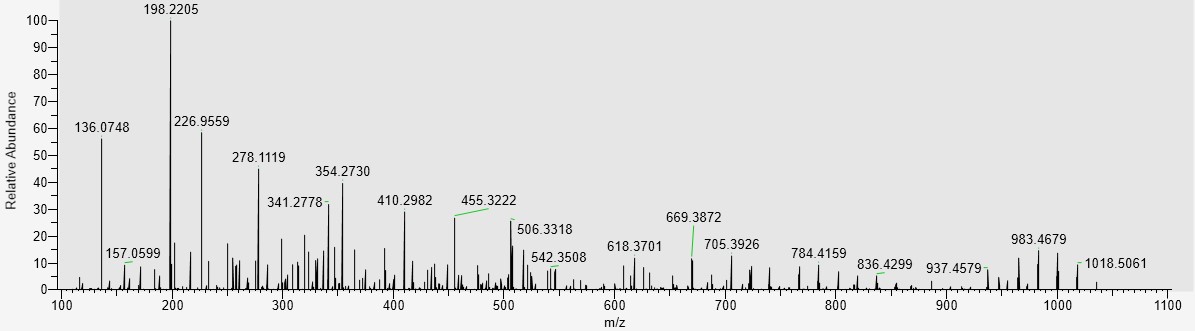

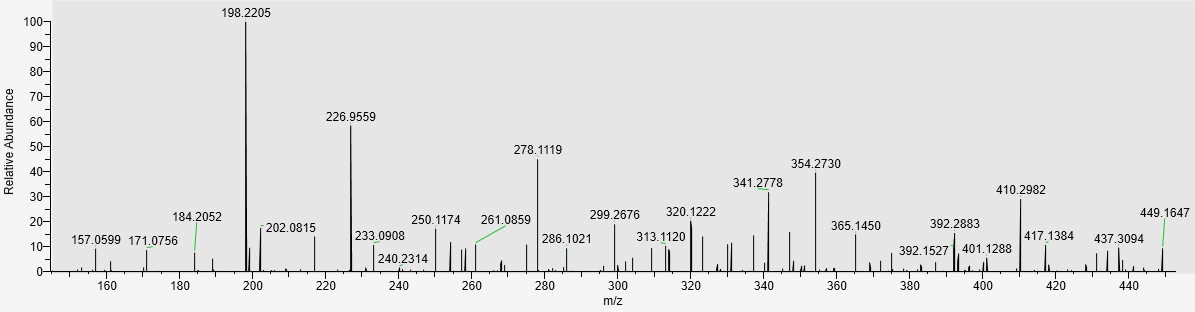


**MS^2^ of C16 bacillomycin L ([M+ H]^+^ = 1049.5539) (*B. velezensis* JJ334)**


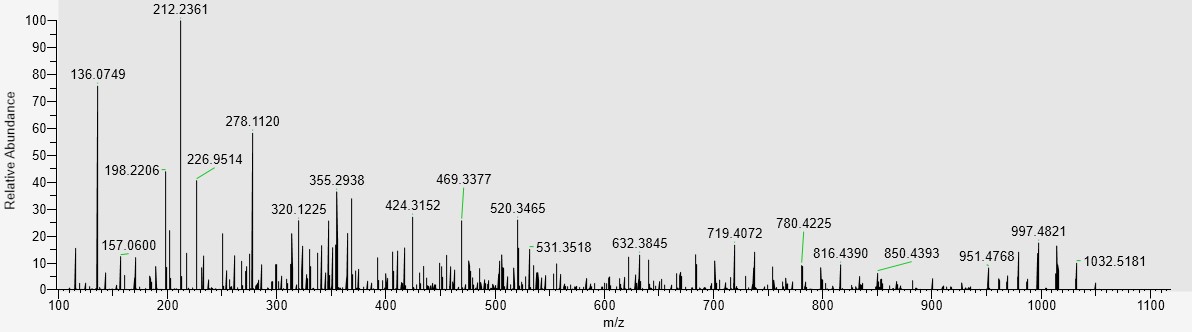

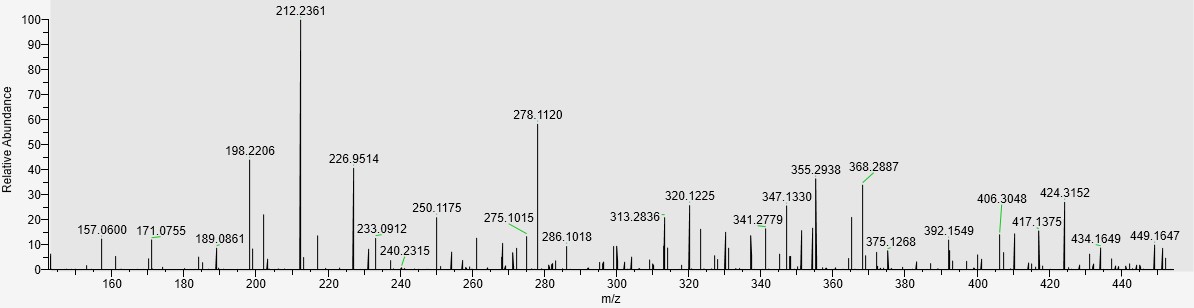


**MS^2^ of C17 bacillomycin L ([M+ H]^+^ = 1063.5666) (*B. velezensis* JJ334)**


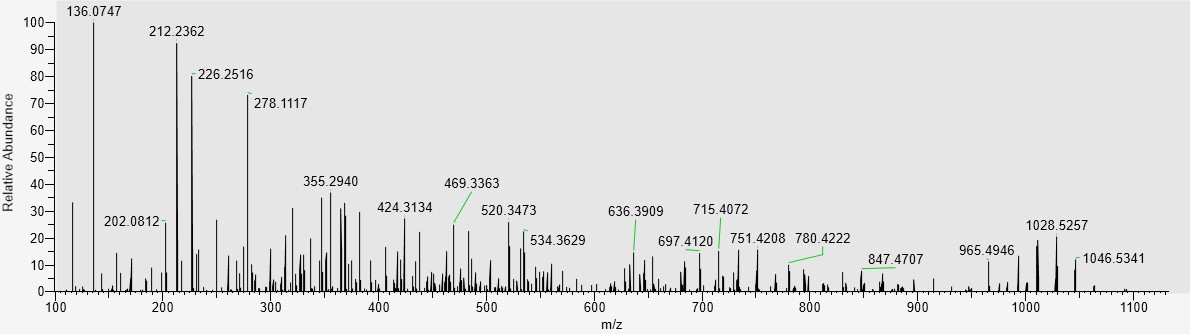

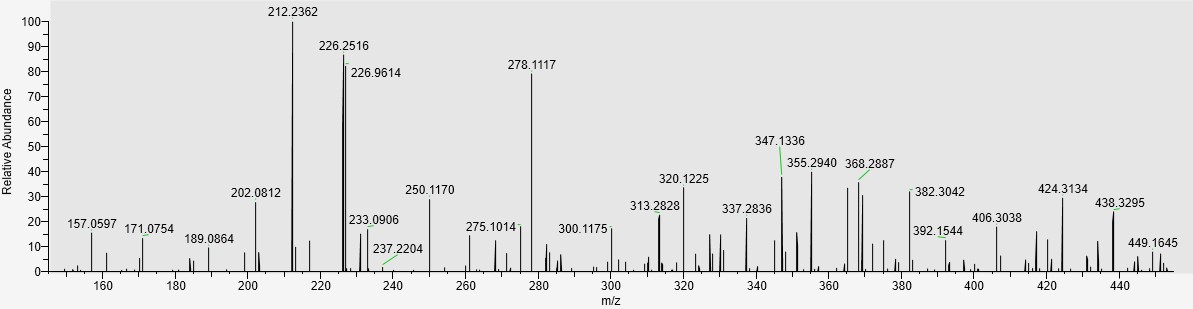


369.3085

**Figure S9.** The structure of bacillomycin L (A), ions generated from diagnostic fragmentation of the core peptide (B), and MS^2^ spectra corresponding to identify bacillomycin L derivatives (C). MS^2^ spectra of six prominent bacillomycin L derivatives were identified from total extracts of *B. velezensis* JJ334. Assignment of bacillomycin L derivatives was based on the fragmentation of the core peptide to generate Asn-Tyr-Asn (I), Asn-Tyr (II) ions as well as the cleavage of the β-hydroxy fatty acid tail. Note that MS^2^ is not available for the bacillomycin L derivative with a *m/z* of 1077.5820.
